# Supplementary material for: Interactions between BRD4S, LOXL2, and MED1 drive cell cycle transcription in triple‐negative breast cancer
Source: EMBO Mol Med. 2023 Nov 8;15(12):e18459. doi: 10.15252/emmm.202318459 (PMC10701626; doi:10.15252/emmm.202318459)
Supplement: Supplementary file 1 — Appendix [file EMMM-15-e18459-s001.pdf]

Appendix

Table of contents

- **Appendix Figure S1: LOXL2 protein levels predict response to BETi** …… 1
- **Appendix Figure S2: Modulation of LOXL2 transcription does not induce changes of BRD4 protein levels** ……………………………………….…… 2
- **Appendix Figure S3: LOXL2 and BRD4 levels are not inversely correlated across tumors** ……………………………………………………….. 5
- **Appendix Figure S4: BRD4 and MED1 foci intensity does not change upon treatment** …..…………………………………………………..………. 6
- **Appendix Figure S5: Characterization of MDA-MB-231 and PDX-127 excised tumors** ……………………………………………..………...7

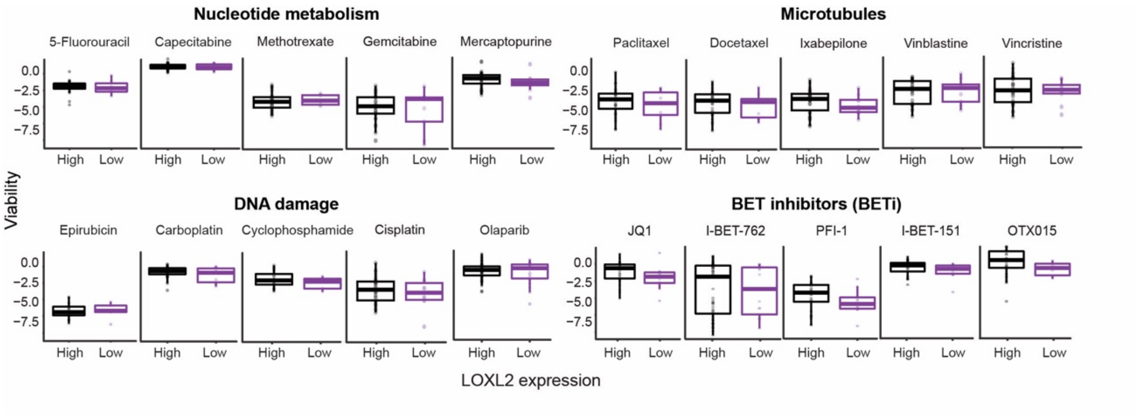

Appendix Figure S1. LOXL2 protein levels predict response to BETi.

Cell viability of high and low LOXL2-expressing CCLE cell lines (protein levels) treated with different chemotherapeutic agents and BETi small molecules at the highest concentration (i.e., 10µM). *N*=80 cell lines. The bottom and top fractions in the boxes represent the first and third quartiles, and the line, the median. Whiskers denote the interval between 1.5 times the interquartile range (IQR) and the median. Data beyond the end of the whiskers are plotted as outliers.

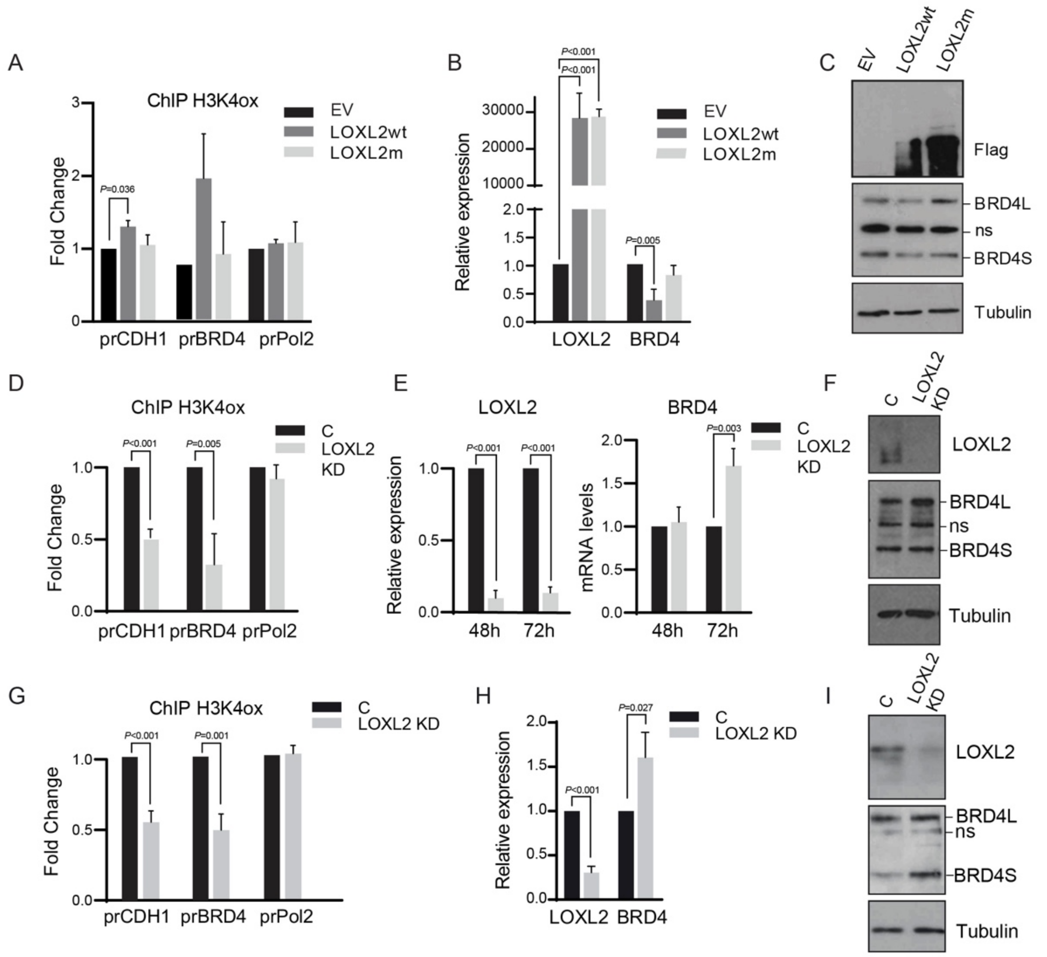

Appendix Figure S2. Modulation of LOXL2 transcription does not induce changes of BRD4 protein levels

A. H3K4ox ChIP-qPCR of the BRD4 promoter (prBRD4) in MDA-MB-468 cells overexpressing either an empty vector (EV), LOXL2-Flag wild-type (LOXL2wt), or the catalytically dead form of LOXL2-Flag (LOXL2m). E-cadherin promoter (prCDH1) and RNA Pol 2 promoter (prPol2) were used as positive and negative controls, respectively. Data from qPCR were normalized to the input and represented as the fold-change relative to the EV condition, which was set as 1. Data are shown as the mean of three independent biological replicates. Standard deviation is shown as error bars. Significance was determined using a one-way ANOVA multiple comparisons with Tukey's correction test.

B. Real-time quantitative PCR (qPCR) showing the changes in mRNA expression of LOXL2 and BRD4 in MDA-MB-468 cells overexpressing EV, LOXL2wt, or LOXL2m. Gene expression was normalized against an endogenous control (Pumilio homolog 1) and represented as the expression relative to the EV condition, which was set as 1. Data are

shown as the mean of three independent biological replicates. Standard deviation is shown as error bars. Significance was determined using a one-way ANOVA multiple comparisons with Tukey's correction test.

C. Representative Western blot of MDA-MB-468 cells overexpressing EV, LOXL2wt, or LOXL2m showing BRD4 and LOXL2 protein levels. Tubulin was used as a loading control, and Flag as a transfection control. ns, non-specific. Three biological replicates were performed.

D. H3K4ox ChIP-qPCR of the BRD4 promoter (prBRD4) in MDA-MB-231 cells infected with C or LOXL2 KD. E-cadherin promoter (prCDH1) and RNA Pol 2 promoter (prPol2) were used as positive and negative controls, respectively. Data from qPCR were normalized to the input and represented as the fold-change relative to the C condition, which was set as 1. Data are shown as the mean of three independent biological replicates. Standard deviation is shown as error bars.

E. Real-time quantitative RT-PCR (qRT-PCR) showing the changes in mRNA expression of LOXL2 and BRD4 in C or LOXL2 KD MDA-MB-231 cells. Gene expression was normalized against an endogenous control (Pumilio homolog 1) and is represented as the expression relative to the EV condition, which was set as 1. Data are shown as the mean of three independent biological replicates. Standard deviation is shown as error bars.

F. Representative Western blot of C and LOXL2 KD MDA-MB-231 cells showing BRD4 and LOXL2 protein levels. Tubulin was used as a loading control. ns, non-specific. Three biological replicates were performed.

G. H3K4ox ChIP-qPCR of the BRD4 promoter (prBRD4) in BT-549 cells infected with C or LOXL2 KD. E-cadherin promoter (prCDH1) and RNA Pol 2 promoter (prPol2) were used as positive and negative controls, respectively. Data from qPCR were normalized to the input and represented as the fold-change relative to the C condition, which was set as 1. Data are shown as the mean of three independent biological replicates. Standard deviation is shown as error bars.

H. Real-time quantitative RT-PCR (qRT-PCR) showing the changes in mRNA expression of LOXL2 and BRD4 in C and LOXL2 KD BT-549 cells. Gene expression was normalized to an endogenous control (Pumilio homolog 1) and represented as the expression relative to EV condition, which was set as 1. Data are shown as the mean of three independent biological replicates. Standard deviation is shown as error bars.

I. Representative Western blot of C and LOXL2 KD MDA-MB-231 cells showing the differences in BRD4 and LOXL2 protein levels. Tubulin was used as a loading control. ns, non-specific. Three biological replicates were performed.

For D, E, G and H, significance was determined by unpaired Student's *t*-test.

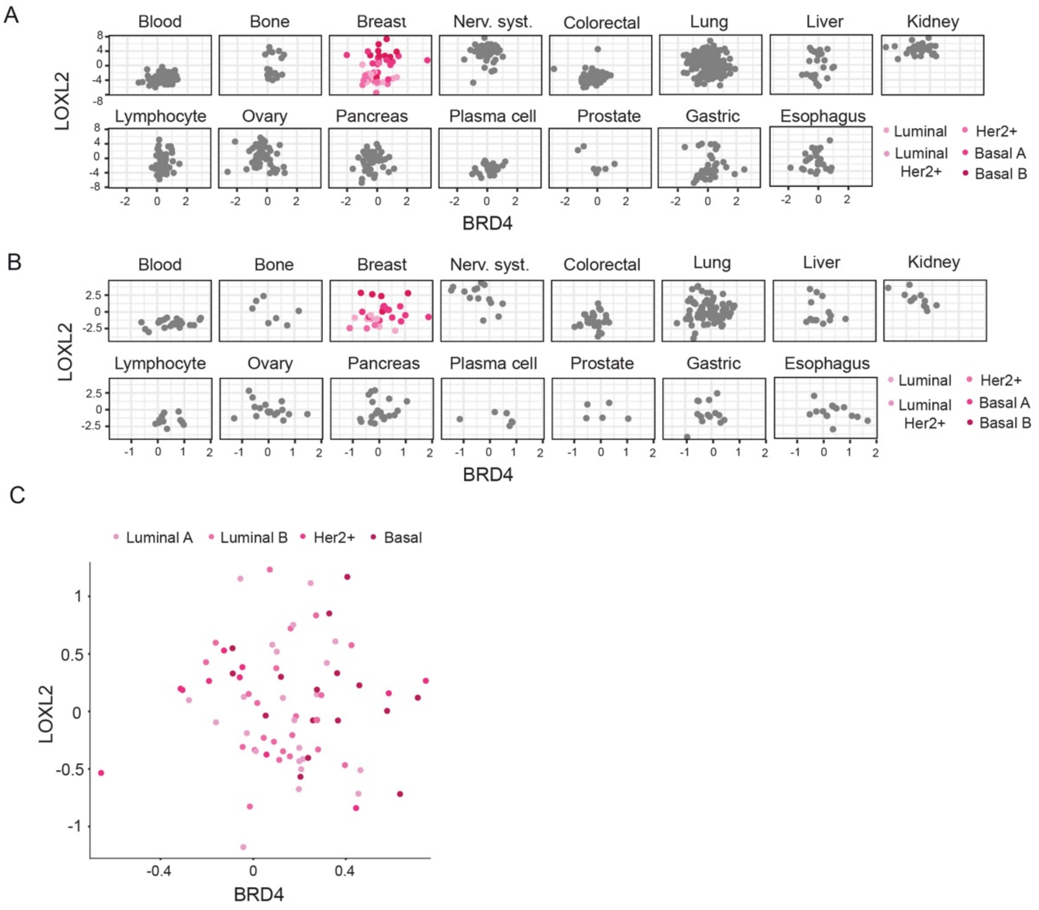

Appendix Figure S3. LOXL2 and BRD4 levels are not inversely correlated across tumors

Correlation between LOXL2 and BRD4 normalized intensities for transcript amounts (**A**) and protein levels (**B**) across CCLE cancer lineages. For breast cancer, different colors indicate different breast cancer subtypes, as indicated in the panel legend.

C. LOXL2-BRD4 protein correlation in TCGA-BRCA mass spectrometry study. Different colors indicate different breast cancer subtypes, as indicated in the panel legend.

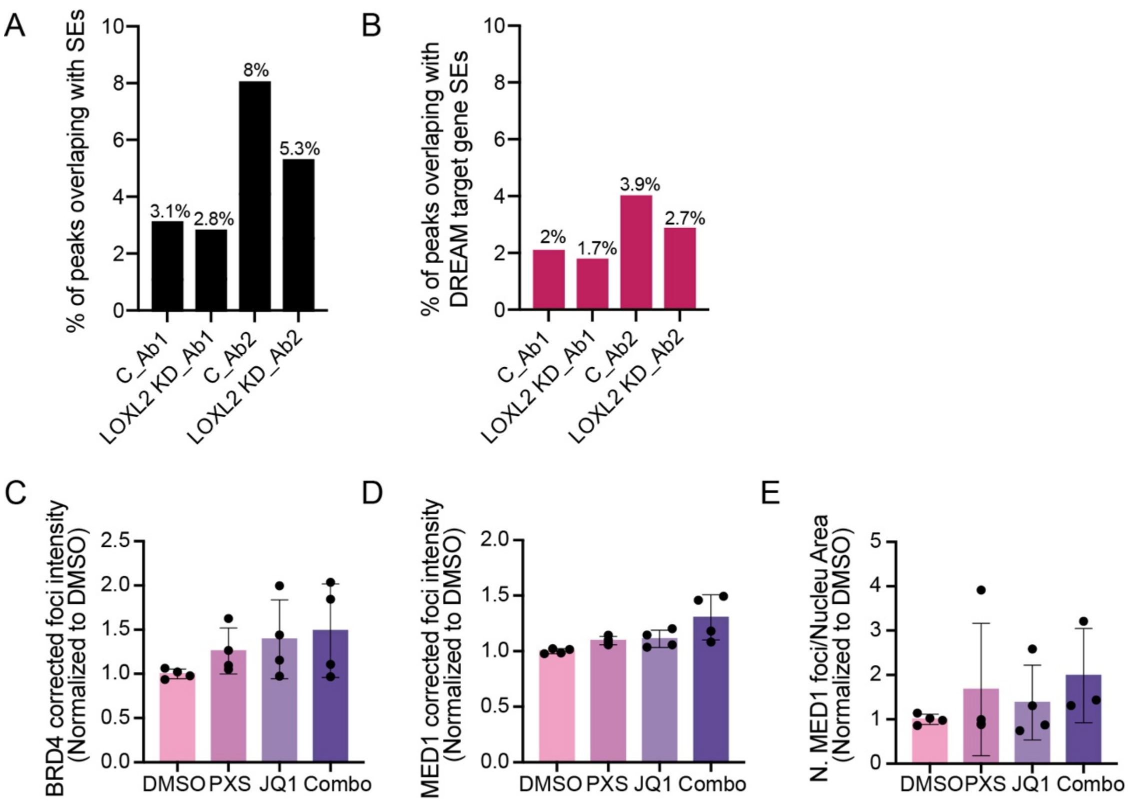

Appendix Figure S4. BRD4 and MED1 foci intensity does not change upon treatment

A. Percentage of Ab1 or Ab2 associated peaks in C or LOXL2 KD conditions overlapping with Super-Enhancers (SE).

B. Percentage of Ab1 or Ab2 associated peaks in C or LOXL2 KD conditions overlapping with Super-Enhancers (SE) located <1Mb of DREAM target genes.

C. Quantification of BRD4 foci intensity from Fig. 5g corrected by the background signal. Results are normalized to DMSO. Data are shown as the mean of four independent biological replicates. Standard deviation is shown as error bars.

D. Quantification of MED1 foci intensity from Fig. 5g corrected by the background signal. Results are normalized to DMSO. Data are shown as the mean of four independent biological replicates. Standard deviation is shown as error bars.

E. Quantification of the number of MED1 foci from Fig. 5g corrected by the nucleus area. Results are normalized to DMSO. Four biological replicates were performed using at least 4000 nuclei/replicate for the analysis. Data are shown as the mean of four independent biological replicates. Standard deviation is shown as error bars. Significance was calculated using a one-way ANOVA multiple comparisons with Tukey's correction test.

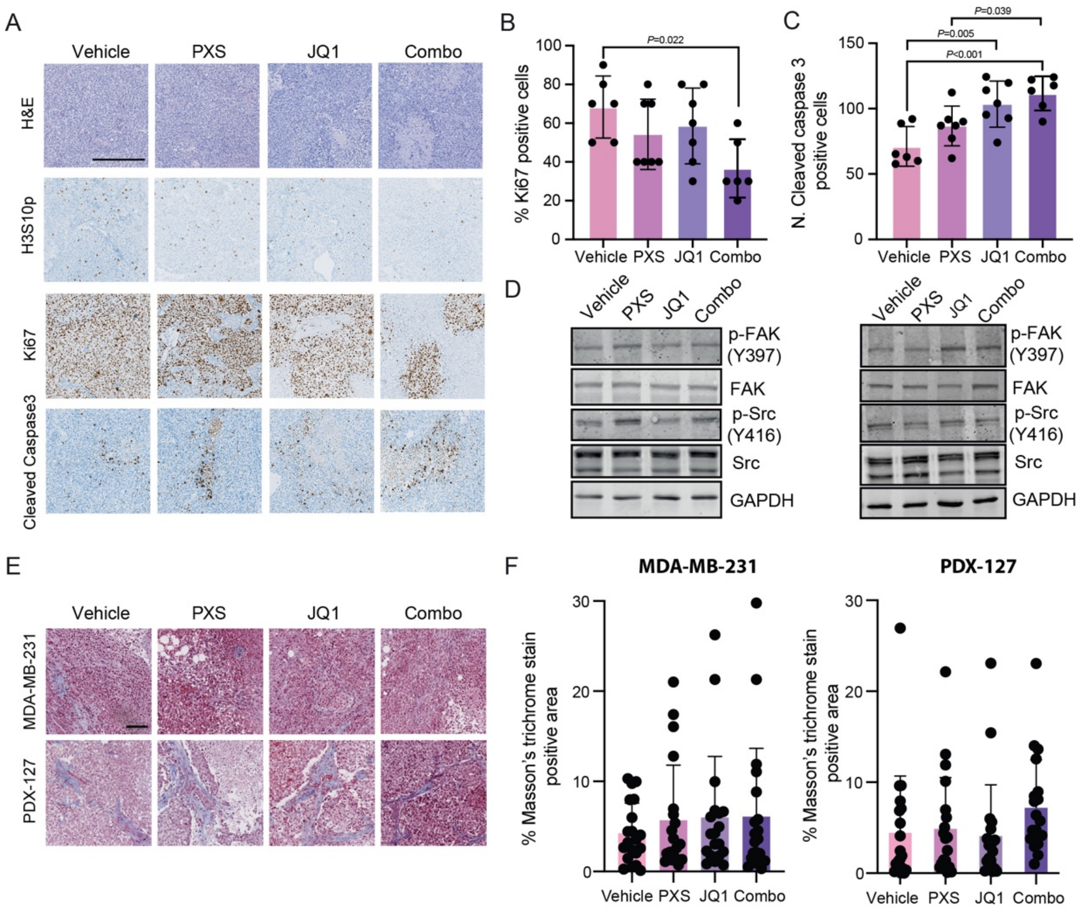

Appendix Figure S5. Characterization of MDA-MB-231 and PDX-127 excised tumors

A. Representative images of hematoxylin-eosin (H&E), H3S10p, Ki67 and cleaved caspase 3 immunohistochemistry of PDX-127 excised tumors at the endpoint (day 15). Scale bar; 100 μm.

B. Quantification of the percentage of Ki67 positive cells in each condition. Six tumors per group were analyzed. Data are shown as the mean of the six tumors. Standard deviations are shown as error bars. Significance was calculated using a one-way ANOVA multiple comparisons with Tukey's correction test.

C. Quantification of the number of cleaved caspase 3 positive cells in each condition. Six tumors per group were analyzed. Data are shown as the mean of the six tumors. Standard deviations are shown as error bars. Significance was calculated using a one-way ANOVA multiple comparisons with Tukey's correction test.

D. Western blots showing phospho FAK (p-FAK), phospho Src (p-Src), as well as FAK and Src total protein levels of PDX-127 excised tumors at the endpoint (day 15). Two

biological replicates were performed and showed (left and right). GAPDH is shown as a loading control.

E. Representative images of Masson's trichrome staining of MDA-MB-231 (top) and PDX-127 (bottom) excised tumors at the endpoint (day 26 and day 15, respectively). Scale bar; 100 μm.

F. Quantification of the percentage of Masson's trichrome stain positive area in each condition from MDA-MB-231 (left) and PDX-127 (right) excised tumors. Ten pictures per tumor and a total of two tumors per group were analyzed. Data are shown as the mean of the replicates. Standard deviations are shown as error bars.
