# Supplementary material for: Interactions between BRD4S, LOXL2, and MED1 drive cell cycle transcription in triple‐negative breast cancer
Source: EMBO Mol Med. 2023 Nov 8;15(12):e18459. doi: 10.15252/emmm.202318459 (PMC10701626; doi:10.15252/emmm.202318459)
Supplement: Supplementary file 2 — Expanded View Figures PDF [file EMMM-15-e18459-s005.pdf]

Expanded View Figures

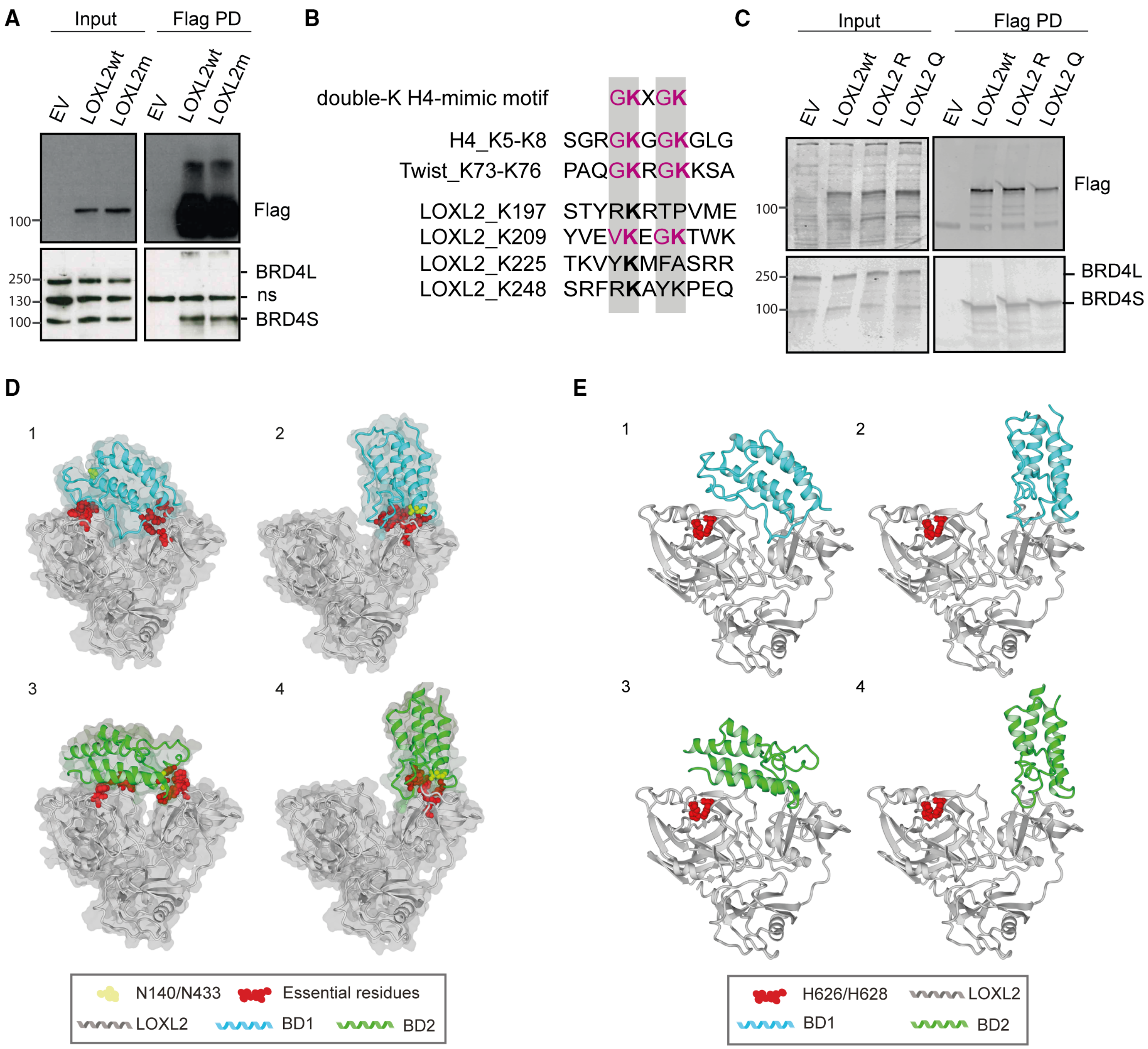

Figure EV1. LOXL2-BRD4S interaction does not involve the activity of the bromodomains.

A Flag pulldown in HEK293T cells overexpressing the empty vector (EV), LOXL2-Flag wild-type (LOXL2wt), or the catalytically dead form of LOXL2-Flag (LOXL2m). Precipitates were analyzed by Western blot with the indicated antibodies. Three biological replicates were performed. ns: non-specific.

B Schematic representation of the double-K H4-mimic motif partially shared between H4, Twist, and LOXL2.

C Flag pulldown of MDA-MB-231 cells overexpressing EV, LOXL2wt, LOXL2 R (the K209 residue mutated to R), or LOXL2 Q (the K209/K212 residues mutated to Q). Precipitates were analyzed by Western blot with the indicated antibodies. Three biological replicates were performed.

D Selected docking models, two for each of the BDs. The docking poses 1 and 2 show the BD1 structure docked on LOXL2, corresponding to models 4uyd_zdock_10 and 4uyd_zdock_3, respectively (Tables EV2 and EV3), while 3 and 4 show the BD2 structures docked into LOXL2, corresponding to models 2ouo_zdock_4 and 2ouo_zdock_5, respectively (Tables EV2 and EV3). Red indicates the atomic representation of residues predicted to be fundamental for the modeled interaction (Table EV3), while yellow indicates the asparagines (N) N140 (BD1), and N433 (BD2). The molecular volumes of the four models are shown in light gray.

E View of the four selected models highlighting LOXL2 histidines H626 and H628 whose mutations to glutamine did not affect the LOXL2 binding to BRD4. Panels 1 to 4 correspond to docking models 4uyd_complex_10, 4uyd_complex_3, 2ouo_complex_4, and 2ouo_complex_5, respectively (Table EV3).

 © 2023 The Authors

Figure EV2. ChIP-seq, RNA-seq, and ATAC-seq analyses of LOXL2 downregulation in MDA-MB-231 TNBC cells.

A Representative Western blot analysis of MDA-MB-231 cells infected with C or LOXL2 KD showing LOXL2 levels. Tubulin was used as a loading control. Three biological replicates were performed.

B ATAC-seq normalized coverage in MDA-MB-231 cells transduced with either C or LOXL2 KD, represented as the distance from the center of all peaks (bp = base pair).

C Volcano Plot representation of the differential expression of genes between C and KD conditions. Significance was calculated using the Wald test with Benjamini–Hochberg correction used for multiple testing. Genes with adjusted *P*-values <0.05 and abs (FC) > 1.5 were considered significant.

D RNA-seq logFC for genes associated with ATAC-seq peaks in C and KD conditions which fall in promoter regions. The bottom and top fractions in the boxes represent the first and third quartiles, and the line, the median. Whiskers denote the interval between 1.5 times the interquartile range (IQR) and the median. Data beyond the end of the whiskers are plotted as outliers.

E Gene Set Enrichment Analysis (GSEA) of the genes upregulated upon LOXL2 KD in the RNA-seq dataset. Significance was calculated using a permutation test with Benjamini-Hochberg correction used for multiple testing.

F Number of BRD4 ChIP-seq total or promoter peaks using Ab1 or Ab2 antibodies identified with MACS2.

G Top-10 GO-terms identified either with Ab1 or Ab2 when analyzing promoter peaks with the mSigDB. Gene ratios and adjusted *P*-values are reported on the left side of each GS. GSs shared among the top 10 of Ab1 and Ab2 are indicated in bold.

H Representative Western blot analysis showing BRD4 levels in MDA-MB-231 cells infected with shControl), shBRD4 Long (BRD4L KD), and shBRD4 Short (BRD4S KD) isoforms. Tubulin was used as a loading control. Three biological replicates were performed. ns: non-specific.

I Heatmap of ChIP-seq normalized signal (reads per genomic content) in all peaks in LOXL2 KD or control cells for the antibodies Ab1 and Ab2. The normalized signal is calculated for a region of −1 to 1 kb from the center of the peaks.

J Normalized ATAC-seq signal (reads per genomic content) in the ChIP-seq peaks for Ab1 and Ab2 in LOXL2 KD or control cells. Significance was calculated using a two-sample Kolmogorov–Smirnov test. The bottom and top fractions in the boxes represent the first and third quartiles, and the line, the median. Whiskers denote the interval between 1.5 times the interquartile range (IQR) and the median. Data beyond the end of the whiskers are plotted as outliers.

K RNA-seq logFC for genes associated with the peaks of Ab1 and Ab2, which fall in promoter regions in control or LOXL2 KD cells, respectively. Significance was calculated using a two-sample Kolmogorov–Smirnov test. The bottom and top fractions in the boxes represent the first and third quartiles, and the line, the median. Whiskers denote the interval between 1.5 times the interquartile range (IQR) and the median. Data beyond the end of the whiskers are plotted as outliers.

L Venn diagram showing the number of promoter genes identified with the ChIP-seq with either Ab1 or Ab2 antibodies in control (left) or LOXL2 KD (right) cells. DREAM target genes identified in each condition are depicted in colored dots. The numbers on top of the dots represent the total number of promoters retrieved for each condition. The numbers below the dots are respectively the (upper) total number of DREAM target gene promoters retrieved in each condition and (lower) the condition-relative percentage of DREAM target gene promoters identified (DREAM target gene promoters relative to all promoters).

M Venn diagram showing the overlap between the total number of peaks detected with the ChIP-seq with either Ab1 or Ab2 in control (left) or LOXL2 KD (right) conditions. The overlap of Ab1 and Ab2 peaks is shown as a percentage (intersection relative to Ab1 peaks).

© 2023 The Authors

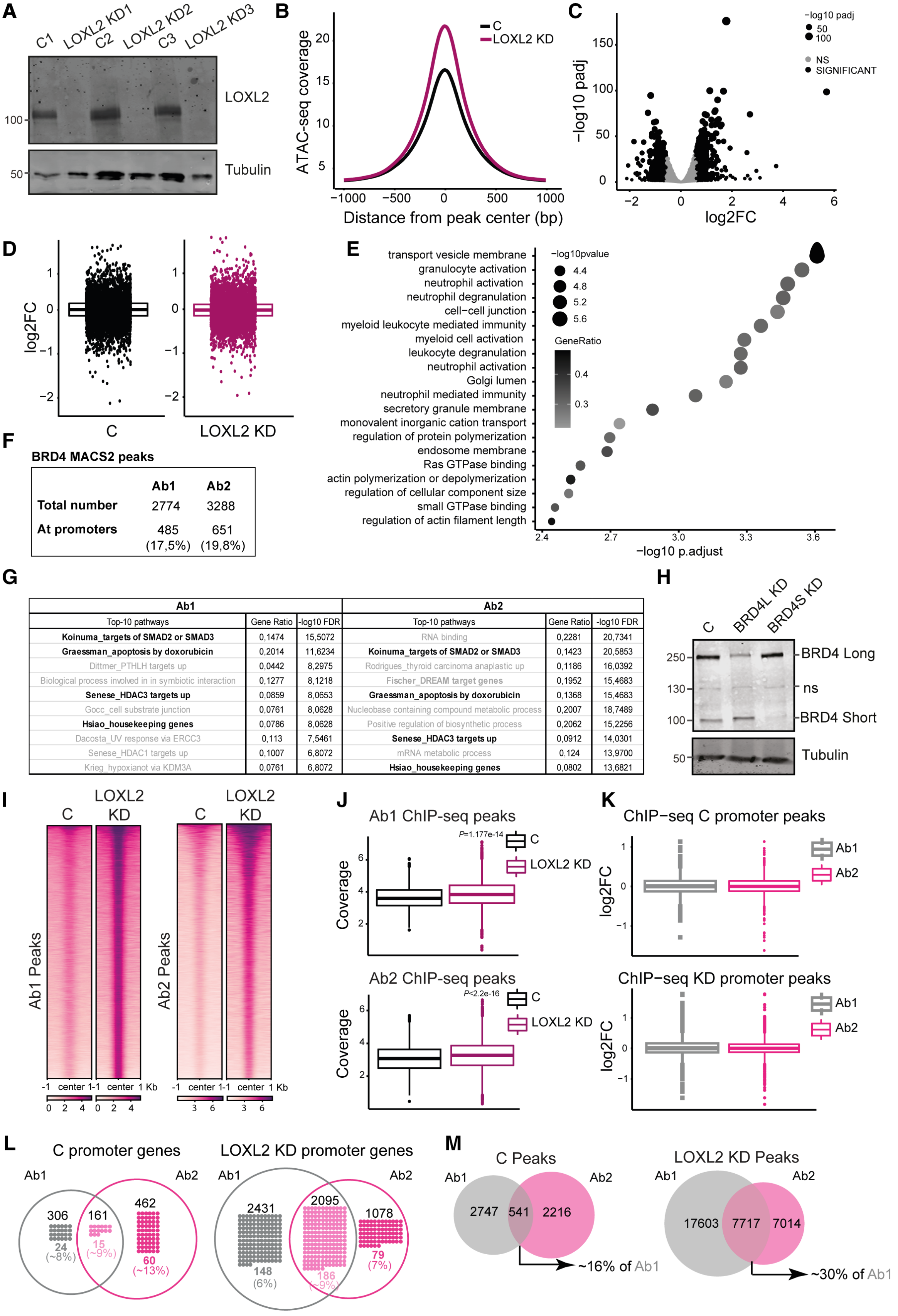

F BRD4 MACS2 peaks

	Ab1	Ab2
**Total number**	2774	3288
**At promoters**	485 (17,5%)	651 (19,8%)

G

Ab1			Ab2		
Top-10 pathways	Gene Ratio	-log10 FDR	Top-10 pathways	Gene Ratio	-log10 FDR
**Koinuma_targets of SMAD2 or SMAD3**	0,1474	15,5072	RNA binding	0,2281	20,7341
**Graessman_apoptosis by doxorubicin**	0,2014	11,6234	**Koinuma_targets of SMAD2 or SMAD3**	0,1423	20,5853
Dittmer_PTHLH targets up	0,0442	8,2975	Rodrigues_thyroid carcinoma anaplastic up	0,1186	16,0392
Biological process involved in in symbiotic interaction	0,1277	8,1218	**Fischer_DREAM target genes**	0,1952	15,4683
**Senese_HDAC3 targets up**	0,0859	8,0653	**Graessman_apoptosis by doxorubicin**	0,1368	15,4683
Gocc_cell substrate junction	0,0761	8,0628	Nucleobase containing compound metabolic process	0,2007	18,7489
**Hsiao_housekeeping genes**	0,0786	8,0628	Positive regulation of biosynthetic process	0,2062	15,2256
Dacosta_UV response via ERCC3	0,113	7,5461	**Senese_HDAC3 targets up**	0,0912	14,0301
Senese_HDAC1 targets up	0,1007	6,8072	mRNA metabolic process	0,124	13,9700
Krieg_hypoxianot via KDM3A	0,0761	6,8072	**Hsiao_housekeeping genes**	0,0802	13,6821

Figure EV2.

 © 2023 The Authors

Figure EV3. LOXL2 repression affects the transcriptional regulation of DREAM target genes.

A Real-time quantitative PCR (qPCR) showing the changes in mRNA expression of four selected DREAM target genes (*EZH2*, *HMGB2*, *AURKB*, and *PLK4*) in C or LOXL2 KD MDA-MB-231 cells. Gene expression was normalized against an endogenous control (Pumilio homolog 1) and represented as the expression relative to the C condition, which was set as 1. Data are shown as the mean of three independent biological replicates. The standard deviation is shown as error bars. Significance was determined by unpaired Student's *t*-test.

B Representative images of H3S10P high-throughput immunofluorescence in LOXL2 KD or C MDA-MB-231 cells. Scale bar, 100 μm.

C Representative Western blot analysis of H3K4ox in MDA-MB-231 cells treated with DMSO or PXS for 96 h at the indicated concentrations. H3 was used as a loading control. Two biological replicates were performed.

D qPCR showing the changes in mRNA expression of four selected DREAM target genes (*EZH2*, *HMGB2*, *AURKB*, and *PLK4*) in MDA-MB-231 cells treated with DMSO or PXS. Gene expression was normalized against an endogenous control (Pumilio homolog 1) and is represented as the expression relative to the DMSO condition, which was set as 1. Data are shown as the mean of three independent biological replicates. The standard deviation is shown as error bars. Significance was determined by unpaired Student's *t*-test.

E MDA-MB-231 cells expressing SLBP-mTurquoise2 and H1-Maroon1 treated with DMSO or PXS for 96 h. Representative images of the quantification in Fig 4D are shown. Images of big panels show SLBP-mTurquoise2 (top) and H1-Maroon1 (bottom); inset panels show their overlap with brightfield. Scale bar, 100 μm.

F Gene-specific differential gene essentiality between high and low LOXL2-expressing cell lines (CCLE) as calculated by analyzing the Achilles' dataset. $N = 80$ cell lines. Significance was determined using the Student's *t*-test with BH multiple hypothesis correction. The bottom and top fractions in the boxes represent the first and third quartiles, and the line, the median. Whiskers denote the interval between 1.5 times the interquartile range (IQR) and the median. Data beyond the end of the whiskers are plotted as outliers.

© 2023 The Authors

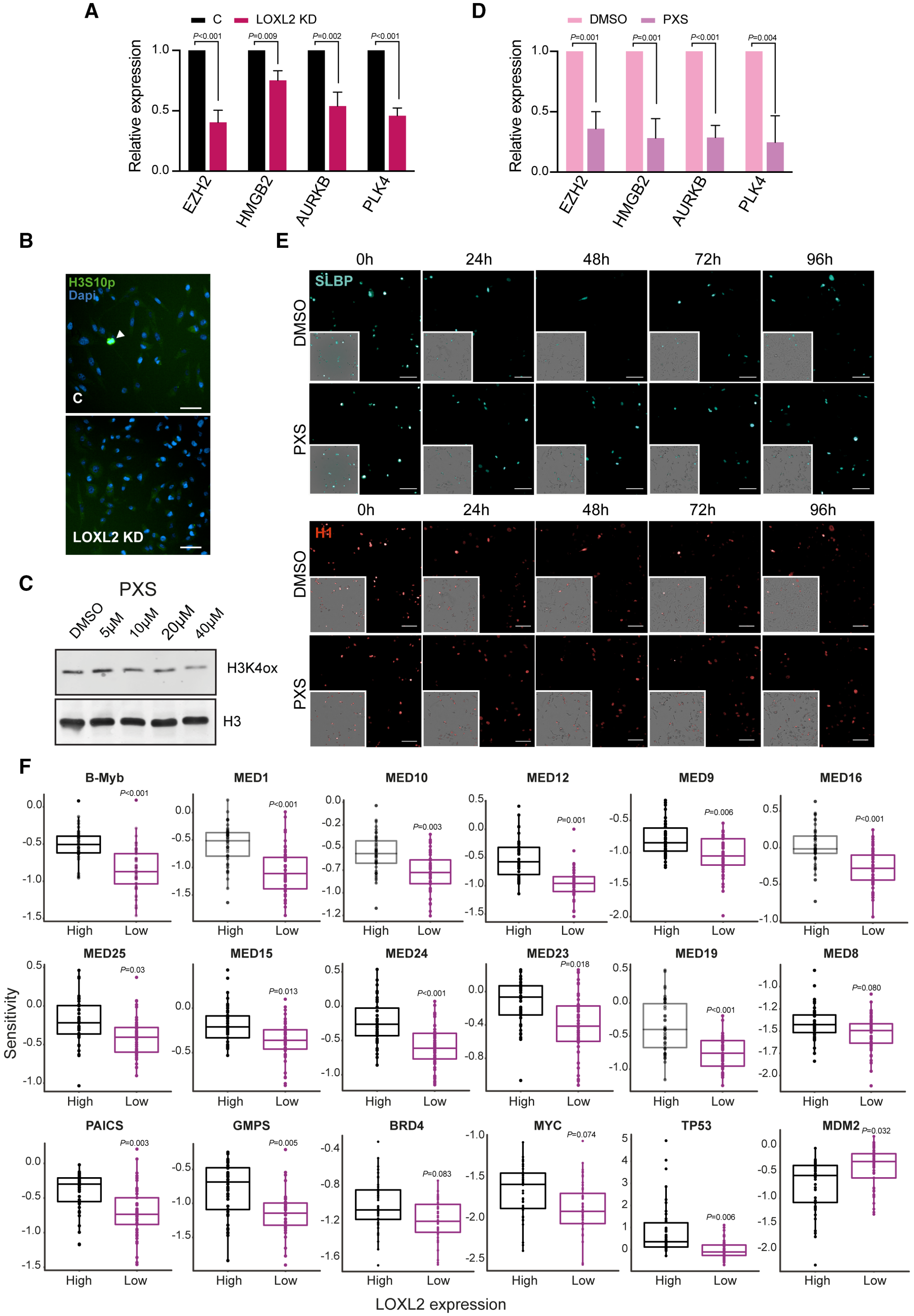

Figure EV3.

 © 2023 The Authors

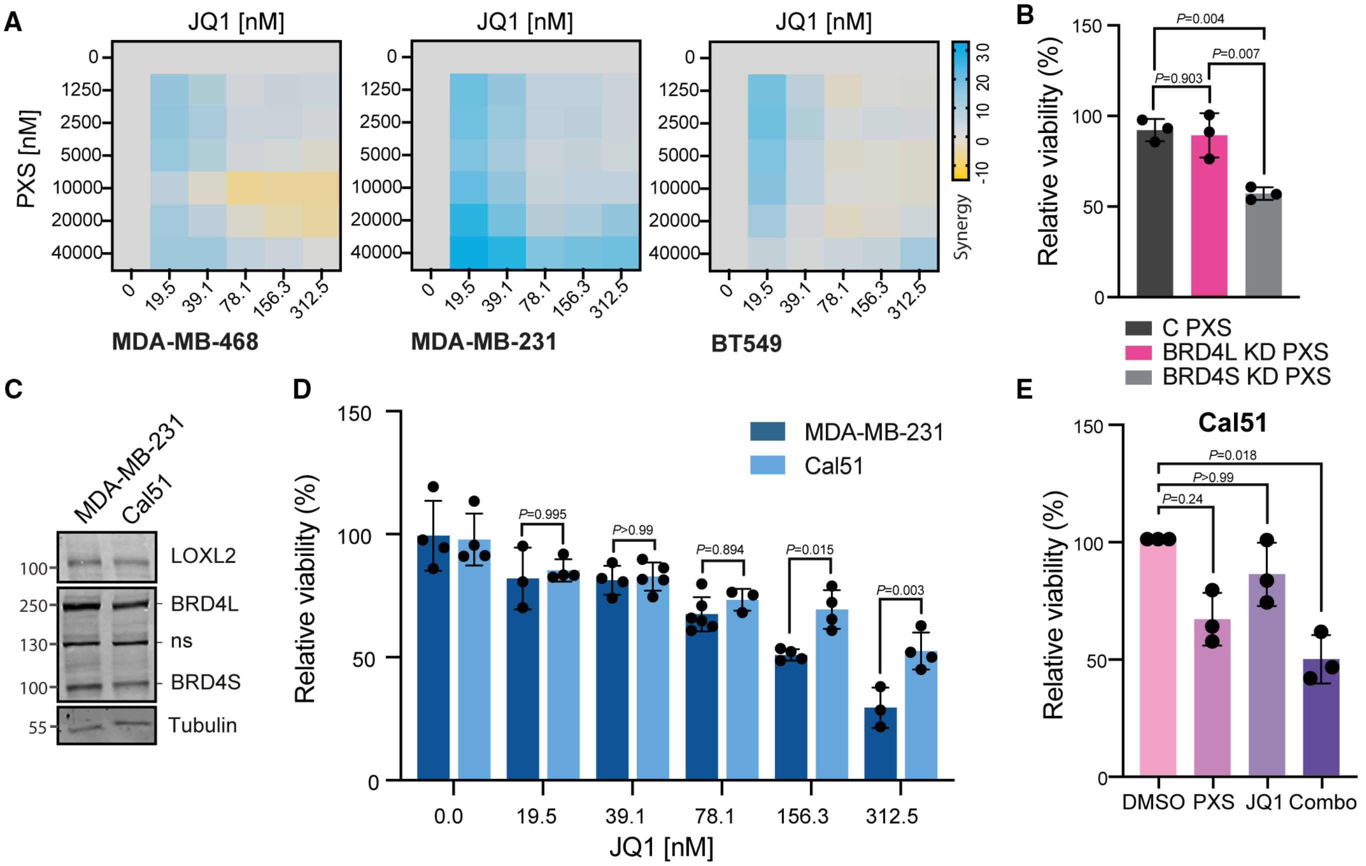

Figure EV4. Effect of LOXL2 and BRD4 inhibition on different TNBC cell lines.

A Representative matrixes showing the synergy score calculated with the cell viability data illustrated in Fig 6A.

B Cell viability assay of MDA-MB-231 cells infected with shControl (C), shBRD4L (BRD4L KD), or shBRD4S (BRD4S KD) and treated with either DMSO or 20 μM of PXS for 96 h. Data were analyzed by DAPI count using the Operetta High Content Screening System and normalized to DMSO. Data are shown as the mean of three independent biological replicates. The standard deviation is shown as error bars. Significance was determined using a one-way ANOVA multiple comparisons with Tukey's correction test.

C Representative Western blot showing BRD4 and LOXL2 levels in MDA-MB-231 and Cal51 cell lines. Tubulin is shown as a loading control. Two biological replicates were performed.

D Cell viability assay of MDA-MB-231 and Cal51 cells treated with the indicated concentrations of JQ1 for 96 h. Data were analyzed with MTT assay and normalized to DMSO. Data are shown as the mean of four independent biological replicates. The standard deviation is shown as error bars. Significance was determined using an unpaired Student's *t*-test.

E Cell viability assay of Cal51 cells treated with either DMSO, 40 μM of PXS, 312.5 nM of JQ1, or the combination of both (Combo) for 96 h. Data were analyzed with MTT assay and normalized to DMSO. Data are shown as the mean of three independent biological replicates. The standard deviation is shown as error bars. Significance was determined using one-way ANOVA multiple comparisons with Dunn's multiple comparisons test.

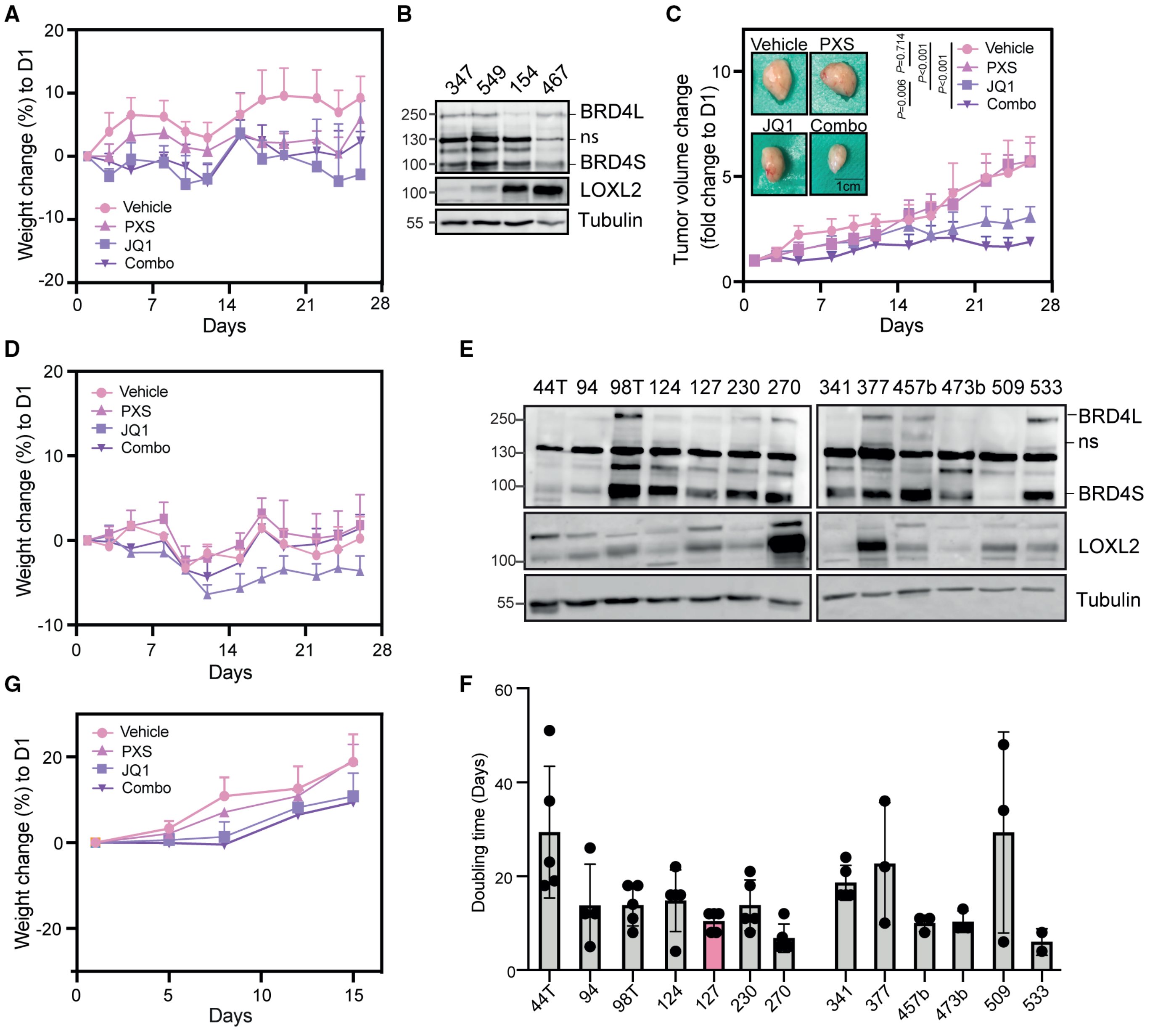

Figure EV5.

 © 2023 The Authors

◀ **Figure EV5. Effect of LOXL2 and BRD4 inhibition on *in vivo* TNBC proliferation.**

A Mouse body weight changes from the MDA-MB-231 xenograft mice treated as in Fig 6B at the endpoint (day 26). Weight changes are represented as the percentage with respect to day 1, and standard deviations are shown as error bars. Body weight changes of less than 10% are considered tolerable. A minimum of six tumors per group (with three mice per group, with one tumor on each side) are shown as average mouse body weight. Statistical analysis was performed using two-way ANOVA multiple comparisons with Tukey's correction test for the whole experiment.

B Representative Western blot showing LOXL2 and BRD4 protein levels of four different PDXs. Tubulin is shown as a loading control; ns: non-specific. Two biological replicates were performed.

C Tumor volumes represented as fold change to day 1 (D1) from PDX-549 mice treated five times per week with 715 mg/kg JQ1 and/or 2 mg per pump PXS for 26 days. A minimum of six tumors per group (with 3 mice per group, with one tumor on each side) are shown as the average tumor volume. Standard deviations are shown as error bars. Significance was determined at the endpoint (day 26) using a two-way ANOVA multiple comparisons with Tukey's correction test (graph). Images of the excised tumors at the end of the experiment (day 26) (pictures).

D Mouse body weight changes from the PDX-549 xenograft mice treated as in C at the endpoint (day 26). Weight changes are represented as the percentage with respect to day 1. Standard deviations are shown as error bars. Body weight changes of less than 10% are considered tolerable. A minimum of six tumors per group (with three mice per group, with one tumor on each side) are shown as average mouse body weight. Statistical analysis was performed using two-way ANOVA multiple comparisons with Tukey's correction test.

E Representative Western blot showing LOXL2 and BRD4 protein levels of 13 different PDXs. Tubulin is shown as a loading control; ns: non-specific. Two biological replicates were performed.

F Quantification of tumor doubling time measured as the day it reaches a volume of 2 as compared to day 1. For each PDX, the different number of tumors are shown (a minimum of three different tumors were analyzed). Data are shown as the mean of the replicates. Standard deviations are shown as error bars.

G Mouse body weight changes from the PDX-127 xenograft mice treated as in Fig 6D at the endpoint (day 15). Weight changes are represented as the percentage with respect to day 1. Standard deviations are shown as error bars. Body weight changes of less than 10% are considered tolerable. A minimum of six tumors per group (with three mice per group, with one tumor on each side) are shown as average mouse body weight. Statistical analysis was performed using two-way ANOVA multiple comparisons with Tukey's correction.

© 2023 The Authors
