# Supplementary material for: Interactions between BRD4S, LOXL2, and MED1 drive cell cycle transcription in triple‐negative breast cancer
Source: EMBO Mol Med. 2023 Nov 8;15(12):e18459. doi: 10.15252/emmm.202318459 (PMC10701626; doi:10.15252/emmm.202318459)
Supplement: Supplementary file 6 — Movie EV1 [file EMMM-15-e18459-s004.zip › Movie_EV1_figure_legend.docx]

**Movie EV1.** **Time-lapse of mTurquoise-SLBP–expressing cells.** Representative time-lapse movie of MDA-MB-231 cells expressing mTurquoise2-SLBP and H1-Maroon1, treated with DMSO or PXS for 96 hours. Images were acquired every 15 min.
