# Supplementary material for: Interactions between BRD4S, LOXL2, and MED1 drive cell cycle transcription in triple‐negative breast cancer
Source: EMBO Mol Med. 2023 Nov 8;15(12):e18459. doi: 10.15252/emmm.202318459 (PMC10701626; doi:10.15252/emmm.202318459)
Supplement: Supplementary file 9 — PDF+ [file EMMM-15-e18459-s011.pdf]

Article

SOURCE DATA | TRANSPARENT PROCESS | OPEN ACCESS

EMBO Molecular Medicine

Interactions between BRD4S, LOXL2, and MED1 drive cell cycle transcription in triple-negative breast cancer

Laura Pascual-Reguant[1], Queralt Serra-Camprubí[2], Debayan Datta[1], Damiano Cianferoni[1], Savvas Kourtis[1], Antoni Gañez-Zapater[1], Chiara Cannatá[1], Lorena Espinar[1], Jessica Querol[2], Laura García-López[1], Sara Musa-Afaneh[1], Maria Guirola[1], Anestis Gkanogiannis[1], Andrea Miró Canturri[2,3], Marta Guzman[2], Olga Rodríguez[2], Andrea Herencia-Ropero[2], Joaquin Arribas[2,3,4,5,6], Violeta Serra[2], Luis Serrano[1], Tian V Tian[2], Sandra Peiró[2,*] & Sara Sdelci[1,**]

Abstract

Triple-negative breast cancer (TNBC) often develops resistance to single-agent treatment, which can be circumvented using targeted combinatorial approaches. Here, we demonstrate that the simultaneous inhibition of LOXL2 and BRD4 synergistically limits TNBC proliferation *in vitro* and *in vivo*. Mechanistically, LOXL2 interacts in the nucleus with the short isoform of BRD4 (BRD4S), MED1, and the cell cycle transcriptional regulator B-MyB. These interactions sustain the formation of BRD4 and MED1 nuclear transcriptional foci and control cell cycle progression at the gene expression level. The pharmacological co-inhibition of LOXL2 and BRD4 reduces BRD4 nuclear foci, BRD4-MED1 colocalization, and the transcription of cell cycle genes, thus suppressing TNBC cell proliferation. Targeting the interaction between BRD4S and LOXL2 could be a starting point for the development of new anticancer strategies for the treatment of TNBC.

Keywords cell cycle; combinatorial therapy; gene expression; triple-negative breast cancer
Subject Categories Cancer; Cell Cycle
DOI 10.15252/emmm.202318459 | Received 3 August 2023 | Revised 16 October 2023 | Accepted 17 October 2023 | Published online 8 November 2023
EMBO Mol Med (2023) 15: e18459

Introduction

Breast cancer is the most diagnosed cancer type and the fourth most common cause of cancer-related death in women, with more than 2 million cases worldwide and 685,000 deaths in 2020 (Lei *et al*, 2021). Breast cancer is commonly classified based on the expression of the estrogen receptor (ER), progesterone receptor (PR), and human epidermal growth factor receptor 2 (HER2), each of which drives cancer proliferation via the activation of precise downstream signaling cascades (Eliyatkın *et al*, 2015). However, 15% of breast cancers do not express any of these receptors (i.e., ER-/PR-/HER2-) and are thus classified as triple-negative breast cancer (TNBC). TNBC is highly metastatic, prone to developing drug resistance, and characterized by high molecular heterogeneity. Indeed, none of the current treatment regimens are effective for treating TNBC, resulting in a dismal prognosis. Efficient and targeted combinatorial treatments could be successful in tackling the heterogeneous and drug-resistant phenotype of TNBC. Thus, discovering molecular factors controlling the proliferation of TNBC is crucial for developing such strategies.

Bromodomain-containing protein 4 (BRD4) is an epigenetic reader known to play a role in the regulation of super-enhancer assembly (Lovén *et al*, 2013; Sengupta & George, 2017) and oncogene transcriptional activation (Delmore *et al*, 2011; Filippakopoulos *et al*, 2012; Muhar *et al*, 2018). Eleven different transcripts exist for *BRD4*, six of which originate different protein isoforms, being the long (BRD4L; ENSP00000470481) and the short (BRD4S; ENSP00000471240) the most prominent ones. Despite the huge similarity in the N-terminal domains which contain the two bromodomains that serve as histones acetyl readers, the short isoform is characterized by the absence of an unstructured proline-rich C-terminal domain. Although the role of BRD4 as a transcriptional activator has been mainly attributed to the long isoform (BRD4L)

1 Centre for Genomic Regulation (CRG), The Barcelona Institute of Science and Technology, Barcelona, Spain
2 Vall d'Hebron Institute of Oncology (VHIO), Barcelona, Spain
3 IMIM (Hospital del Mar Medical Research Institute), Barcelona, Spain
4 Centro de Investigación Biomédica en Red de Cáncer, Monforte de Lemos, Madrid, Spain
5 Department of Biochemistry and Molecular Biology, Universitat Autónoma de Barcelona, Bellaterra, Spain
6 Institució Catalana de Recerca i Estudis Avançats (ICREA), Barcelona, Spain
*Corresponding author. Tel: +34 932543450; E-mail: speiro@vhio.net
**Corresponding author. Tel: +34 933160149; E-mail: sara.sdelci@crg.eu

© 2023 The Authors. Published under the terms of the CC BY 4.0 license

(Drumond-Bock & Bieniasz, 2021), recent evidence demonstrated that BRD4S promotes the formation of phase-separated transcriptional foci, which sustain gene expression and cancer cell proliferation (Han *et al*, 2020). Moreover, in TNBC, BRD4S has oncogenic properties while BRD4L plays a role as a tumor suppressor (Wu *et al*, 2020), suggesting an opposing function of the two isoforms in tumor biology. Several BRD4 inhibitors, known as Bromo- and Extra-Terminal domain (BET) inhibitors (BETi), have been tested in multiple cancer models, and 30 clinical trials are currently ongoing to evaluate their anticancer efficacy (Shorstova *et al*, 2021). In breast cancer, the inhibition of BRD4 has shown promising preclinical results, sparking enthusiasm for TNBC treatment (Andrikopoulou *et al*, 2020). However, due to its heterogeneous and aggressive nature, TNBC develops resistance to single-agent-approaches (Marra *et al*, 2020), including BETi (Shu *et al*, 2016).

Lysyl oxidase-like 2 (LOXL2) is a member of the lysyl oxidase family of copper-dependent amine oxidases (Jung *et al*, 2003), which catalyzes the oxidative deamination of peptidyl lysine residues. In the extracellular matrix, LOXL2 activity promotes collagen, elastin (Añazco *et al*, 2016), and tropoelastin (Schmelzer *et al*, 2019) crosslinks, a phenomenon that is associated with the accumulation of the extracellular matrix, fibrosis, and inflammation, which are all typical hallmarks of cancer (Pickup *et al*, 2014; Chandler *et al*, 2019; Hanahan, 2022). Intracellularly, LOXL2 localizes to the nucleus, where it promotes the oxidation of nuclear proteins such as TAF10 (Iturbide *et al*, 2015b) and Histone 3 (Iturbide *et al*, 2015a; Herranz *et al*, 2016), leading to transcriptional repression and heterochromatinization, respectively. Recently, LOXL2 has been shown to play a pivotal role in different solid cancers, including liver, pancreas, lung, and breast (Ahn *et al*, 2013; Salvador *et al*, 2017; Dinca *et al*, 2021). Strikingly, LOXL2 repression efficiently reduces TNBC cell proliferation (Chang *et al*, 2017), and inhibits the formation of TNBC distal metastasis (Salvador *et al*, 2017).

Here, we show that LOXL2 expression can predict the outcome of BETi treatment in cancer cells, suggesting a functional interaction between LOXL2 and BRD4. We explored this functional interaction in the context of TNBC and discovered that it is mediated by the physical interaction of LOXL2 with the short isoform of BRD4 (BRD4S) in the nucleus of TNBC cells. Chip-seq and transcriptomics analysis highlighted that the interaction between LOXL2 and BRD4S promotes the expression of cell cycle genes, thereby controlling TNBC proliferation. Essentiality analysis revealed that cells expressing low levels of LOXL2 are overly sensitive to the loss of the cell cycle transcription factor B-Myb and BRD4 functional partners, including several subunits of the Mediator complex (being MED1 one of the most essential). Furthermore, BRD4S, LOXL2, and MED1 interact with B-Myb and with each other. By simultaneously inhibiting LOXL2 and BRD4, the LOXL2-BRD4-MED1 interactions are dismantled, impacting BRD4 transcriptional foci formation, BRD4-MED1 colocalization, and cell cycle gene expression. The phenotypic consequence arising from the combinatorial treatment is a clear synergistic effect in suppressing TNBC proliferation, which we tested *in vitro* and in three independent *in vivo* models. Our results describe a completely new molecular pathway controlling TNBC proliferation and pave the way for further research into co-targeting BRD4S and LOXL2 as novel TNBC therapy.

Results

LOXL2 expression levels predict BRD4 inhibition sensitivity in cancer cells

LOXL2 is highly expressed in aggressive tumor types (Fong *et al*, 2007) and plays a key role in promoting breast cancer metastasis (Salvador *et al*, 2017). Given the fact that it has been associated with cancer progression and invasiveness (Wu & Zhu, 2015), we wondered whether the expression of LOXL2 could be used to predict tumor response to standard chemotherapeutic drugs. By analyzing the Cancer Cell Line Encyclopedia (CCLE)-associated chemotherapeutics sensitivity (Corsello *et al*, 2020), we observed that high levels of LOXL2 expression make cells significantly more sensitive to Vinca alkaloid compounds. However, none of the chemotherapeutic groups showed increased efficacy in cells with low LOXL2 expression, a condition that could be mimicked in the clinic by using LOXL2 inhibitors (Fig 1A). As more than 30 clinical trials are currently investigating BETi treatments for solid and hematological malignancies (www.clinicaltrials.gov), we checked for a functional interaction between LOXL2 expression and BRD4 inhibition. When comparing the CCLE-associated BETi sensitivity, we observed that LOXL2 high-expressing cell lines were less sensitive to BETi compounds than LOXL2 low-expressing cell lines (Fig 1A). A similar behavior was observed when cell lines were classified based on LOXL2 protein levels (Appendix Fig S1). These data indicate that LOXL2 expression in

Figure 1. LOXL2 low expression levels sensitize cells to BRD4 inhibition.

A Cell viability of high and low LOXL2-expressing CCLE cell lines (mRNA levels) treated with different chemotherapeutic agents and BETi small molecules at the highest concentration (i.e., 10 μM). Color gradient indicates cell viability, with 8 being the highest and 0 the lowest. Significance was determined using an unpaired Student's *t*-test with BH correction.

B Analysis of the CPTAC proteomics dataset showing the protein abundance of BRD4 and LOXL2 in different breast cancer subtypes from tumor samples classified by subtype using vimentin (VIM), human epidermal growth factor receptor 2 (HER2), progesterone receptor (PGR), and estrogen receptor 1 (ESR1) protein abundance. $N = 96$ tumor samples. The significance of each cancer subtype against adjacent tissue was calculated using one-way ANOVA with Tukey's *post hoc* test. The bottom and top fractions in the boxes represent the first and third quartiles, and the line, the median. Whiskers denote the interval between 1.5 times the interquartile range (IQR) and the median. Data beyond the end of the whiskers are plotted as outliers.

C Representative Western blot analysis showing BRD4 and LOXL2 protein levels in three different TNBC cell lines. Tubulin is the loading control (ns: non-specific). Three biological replicates were performed.

D Cell viability assay of MDA-MB-231 cells infected with C or LOXL2 KD and treated with either DMSO or 2.5 μM of JQ1 for 24 and 48 h. Data were analyzed with MTT assay and normalized to condition C treated with DMSO. Data are shown as the mean of three independent biological replicates. The standard deviation is shown as error bars. Significance was determined using one-way ANOVA multiple comparisons with Tukey's correction test.

Source data are available online for this figure.

© 2023 The Authors

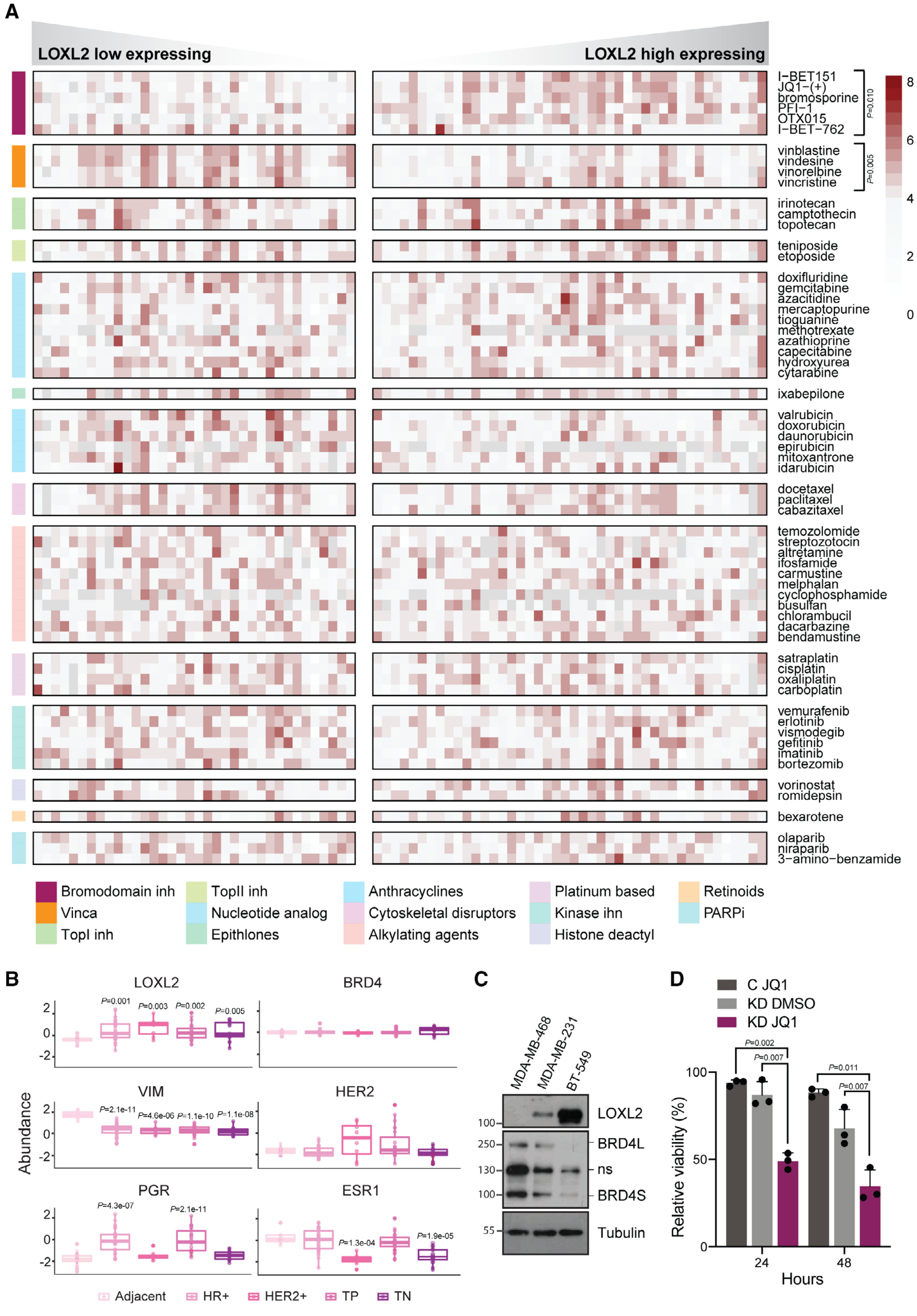
A
LOXL2 low expressing
LOXL2 high expressing
I−BET151
JQ1−(+)
bromosporine
PFI−1
OTX015
I−BET−762
P=0.010
vinblastine
vindesine
vinorelbine
vincristine
P=0.005
irinotecan
camptothecin
topotecan
teniposide
etoposide
doxifluridine
gemcitabine
azacitidine
mercaptopurine
tioguanine
methotrexate
azathioprine
capecitabine
hydroxyurea
cytarabine
ixabepilone
valrubicin
doxorubicin
daunorubicin
epirubicin
mitoxantrone
idarubicin
docetaxel
paclitaxel
cabazitaxel
temozolomide
streptozotocin
altretamine
ifosfamide
carmustine
melphalan
cyclophosphamide
busulfan
chlorambucil
dacarbazine
bendamustine
satraplatin
cisplatin
oxaliplatin
carboplatin
vemurafenib
erlotinib
vismodegib
gefitinib
imatinib
bortezomib
vorinostat
romidepsin
bexarotene
olaparib
niraparib
3−amino−benzamide
8
6
4
2
0
Bromodomain inh
Vinca
TopI inh
TopII inh
Nucleotide analog
Epithlones
Anthracyclines
Cytoskeletal disruptors
Alkylating agents
Platinum based
Kinase ihn
Histone deactyl
Retinoids
PARPi
B
LOXL2
BRD4
VIM
HER2
PGR
ESR1
Abundance
P=0.001
P=0.003
P=0.002
P=0.005
P=2.1e-11
P=4.6e-06
P=1.1e-10
P=1.1e-08
P=4.3e-07
P=2.1e-11
P=1.3e-04
P=1.9e-05
Adjacent
HR+
HER2+
TP
TN
C
MDA-MB-468
MDA-MB-231
BT-549
LOXL2
BRD4L
ns
BRD4S
Tubulin
100
250
130
100
55
D
C JQ1
KD DMSO
KD JQ1
P=0.002
P=0.007
P=0.011
P=0.007
Relative viability (%)
100
50
0
24
48
Hours

Figure 1.

tumor cells can be used to stratify patients into potential BETi responders or refractors.

Recent studies have shown that inhibiting LOXL2 (Chang *et al*, 2017) or BRD4 (Shu *et al*, 2016) can delay the growth of TNBC, which remains an unmet medical need. However, TNBC heterogeneity promotes the development of resistance to single-agent therapies, which may be circumvented by combinatorial treatments with synergistic potential. Therefore, we focused our study on TNBC to understand the molecular basis of the observed functional interaction between LOXL2 and BRD4, and whether their simultaneous inhibition could be exploited as a therapeutic possibility.

By taking advantage of the CPTAC proteomics dataset (Krug *et al*, 2020), we stratified breast cancer samples into different breast cancer subtypes and observed that LOXL2 protein levels were significantly increased in each of them, whereas BRD4 only showed a mild increase in TNBC (Fig 1B). We then performed western blot analysis of three TNBC cell lines expressing distinct levels of LOXL2 (Cebrià-Costa *et al*, 2020) (MDA-MB-468, MDA-MB-231, and BT-549), and we observed that BRD4 protein levels showed an opposite pattern (Fig 1C). LOXL2 induces chromatin compaction via H3K4 oxidation (Cebrià-Costa *et al*, 2020), whereas BRD4 acts as a transcriptional activator. Therefore, we investigated whether the balance between BRD4 and LOXL2 protein levels was the result of transcriptional co-regulation. To test this, we overexpressed C-terminal Flag-tagged LOXL2 wild-type (herein, LOXL2wt) in the MDA-MB-468 cell line that expresses almost undetectable levels of endogenous LOXL2 (Fig 1C). H3K4 oxidation increased at the *BRD4* promoter followed by a mild reduction in *BRD4* gene expression. As expected, the same effect was not achieved by overexpressing the FLAG-tagged catalytically dead form of LOXL2 (LOXL2m) (Appendix Fig S2A and B). However, LOXL2wt overexpression resulted in minimal changes in BRD4 protein level (Appendix Fig S2C). Similarly, when LOXL2 was downregulated in both the MDA-MB-231 and BT-549 TNBC cell lines (both of which normally express medium to high levels of LOXL2) (Fig 1C), we observed decreased H3K4 oxidation at the *BRD4* promoter, followed only by mildly increased *BRD4* gene expression and almost no changes in BRD4 protein levels (Appendix Fig S2D–I). Next, we analyzed a broader cancer cell panel to determine whether there was a consistent inverse correlation between BRD4 and LOXL2 expression. The CCLE transcriptomics and proteomics datasets (Ghandi *et al*, 2019) did not show any correlation between *LOXL2* and *BRD4* mRNA (Appendix Fig S3A) or protein (Appendix Fig S3B) levels across lineages. Similarly, no correlation was observed when analyzing the TCGA proteomics data of human breast tumor samples (Gao *et al*, 2013) (Appendix Fig S3C).

Despite the absence of a correlation between BRD4 and LOXL2 expression levels, we could recapitulate that low expression of LOXL2 sensitize cells to BRD4 inhibition. In particular, we transduced MDA-MB-231 cells with shControl (C) or shLOXL2 (Knock-Down, LOXL2 KD) and treated them with either DMSO or the BETi (*S*)-JQ1 (hereafter, JQ1) (Filippakopoulos *et al*, 2010). Notably, LOXL2 KD in combination with JQ1 treatment had the highest impact on cellular viability (Fig 1D). These results confirm that LOXL2 expression is also a predictor of response to BETi in TNBC cells and suggest that simultaneous inhibition of LOXL2 and BRD4 could be explored as a possible treatment for TNBC.

Taken together, these data suggest the presence of a functional interaction between BRD4 and LOXL2, which may underlie future therapeutic interventions for cancer treatment, and which we therefore further investigated at the molecular, cellular, and tumor levels.

LOXL2 interacts with the short isoform of BRD4 via its bromodomains in an acetylation-independent manner

We therefore asked whether the functional interaction between BRD4 and LOXL2 implied a physical interaction. To test this hypothesis, we performed nuclear BRD4 pulldown in MDA-MB-231 cells and observed an interaction with LOXL2 (Fig 2A). As there are no efficient LOXL2 antibodies to perform endogenous LOXL2 pulldown, we carried out a complementary experiment by transiently overexpressing LOXL2wt in MDA-MB-231 cells and performing Flag pulldown instead. Surprisingly, the results showed that nuclear LOXL2 selectively interacted with the short isoform of BRD4 (BRD4S) (Fig 2B). This interaction was also retained when LOXL2m was overexpressed, suggesting that the catalytic activity of LOXL2 is dispensable to such interaction (Fig 2B). Comparable results were obtained when transfecting LOXL2wt or LOXL2m into HEK-293-T cells, in which endogenous LOXL2 expression was undetectable (Fig EV1A).

BRD4 binds to acetylated proteins (Huang *et al*, 2009; Shi *et al*, 2014; Behera *et al*, 2019; Sdelci *et al*, 2019) via the lysine acetylation (AcK) binding pocket of its bromodomains, BD1 and BD2. Thus, we aimed to identify possible LOXL2 acetylated residues that would explain its interaction with BRD4S. The Phosphosite database (Hornbeck *et al*, 2015) indicates that four different LOXL2 lysine (K) residues have been previously described as acetylated (K197, K209, K225 (Zhao *et al*, 2010), and K248). The residue K209 is positioned in a double-K, H4-mimic–similar motif (K209-K212) (Fig EV1B). H4-mimic motifs are known to promote BRD4 binding to acetylated proteins, including the acetylated histone H4 (Morinière *et al*, 2009; Filippakopoulos *et al*, 2012) and the transcription factor Twist (Shi *et al*, 2014). However, we mutated either the K209 residue to abrogate its acetylation (K → R) or the K209-K212 to mimic it (K → Q) and we did not observe any significant changes in the LOXL2-BRD4S interaction (Fig EV1C), indicating that acetylation of these residues is most likely not required. To further investigate the BRD4S-LOXL2 interaction module, we performed Flag-pulldown in HEK293 cells that overexpressed LOXL2wt together with a GFP-tagged version of: (i) BRD4S (1); (ii) BRD4S-N140F/N433F, which has inactivating mutations in the BD1 and BD2 AcK-binding pockets (2); (iii) BRD4_BD1 (3); (iv) BRD4_BD1/BD2 (4); (v) BRD4_BD2 (5); (vi) BRD4S-specific C-terminal domain (6); or (vii) BRD4 long isoform (BRD4L) (7) (Fig 2C). Flag-pulldown confirmed that LOXL2 interacted with BRD4S as well as with all constructs (ii to v), but not with BRD4L (Fig 2D). These experiments confirmed the specific interaction between LOXL2 and BRD4S, indicated that the bromodomains are contributing to the interaction independently of their functional activity, thus suggesting that the interaction is LOXL2 acetylation independent. Finally, the fact that the C-terminal domain of BRD4S alone (6) can interact with LOXL2 may partially explain why BRD4L cannot engage in this interaction, which could also be prevented by the unstructured C-terminal domain of BRD4L.

To propose binding models between BRD4 bromodomains 1 and 2 (BD1 and BD2) and LOXL2, we performed a docking analysis of

 © 2023 The Authors

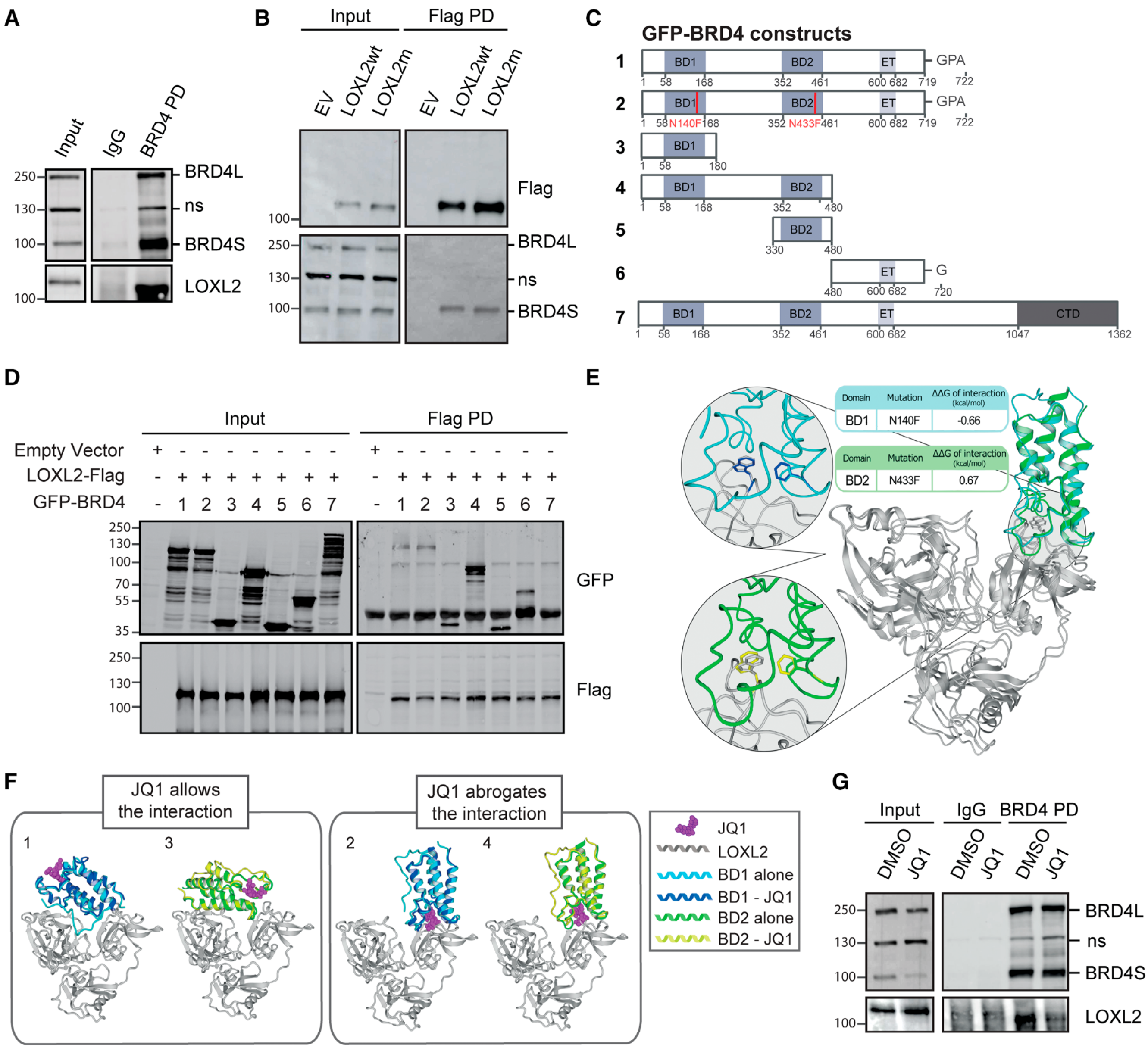

Figure 2. LOXL2 interacts specifically with the short isoform of BRD4.

A BRD4 pulldown in MDA-MB-231 cells using irrelevant IgG as a negative control. Precipitates were analyzed by Western blot with the indicated antibodies. Three biological replicates were performed; ns: non-specific.

B Flag pulldown performed in MDA-MB-231 cells overexpressing either empty vector (EV), Flag-tagged LOXL2 wild-type (LOXL2wt), or the catalytically dead form of LOXL2 (LOXL2m). Precipitates were analyzed by Western blot with the indicated antibodies. Three biological replicates were performed. ns: non-specific.

C Schematic representation of BRD4-GFP constructs used in (D).

D Flag pulldown of HEK293T cells overexpressing either empty vector (EV) or a combination of the indicated constructs. Precipitates were analyzed by Western blot with the indicated antibodies. Three biological replicates were performed.

E Details of docking models 4uyd_complex_3 (BD1), 2ouo_complex_5 (BD2), and their Asp→Phe mutant versions. The panel shows the superposition of LOXL2 (gray) docked to BD1 (cyan) and BD2 (green), with asparagines N140 and N433 mutated to phenylalanine (blue and yellow, respectively), both of which face the buried tryptophan W493 from LOXL2. On the left, zoomed visions of the mutants are shown superposed over their wild-type structures.

F Superimposition of selected docking models of BD1 (PDB: 3MXF) or BD2 (PDB: 3ONI) captured as crystallographic structures binding JQ1. Superimposition on 3MXF of the docking model of BD1 (4uyd_zdock_10; panel 1) or of BD2 (2ouo_zdock_4, panel 3) shows the compatibility of binding despite the presence of JQ1 in the AcK binding pocket. In contrast, superimposition on 3ONI of the docking model of BD1 (4uyd_zdock_3; panel 2) or of BD2 (2ouo_zdock_5, panel 4) shows the incompatibility of binding due to binding site competition of JQ1 and LOXL2.

G BRD4 pulldown in MDA-MB-231 cells treated either with DMSO or with 5 μM of JQ1 for 24 h. IgGs were used as a negative control, and the precipitates were analyzed by Western blot with the indicated antibodies. Three biological replicates were performed. ns: non-specific.

Source data are available online for this figure.

BRD4_BD1/LOXL2 and BRD4_BD2/LOXL2. We used a collection of structures (Table EV1) from the Protein Data Bank (Berman *et al*, 2000) (PDB) and Interactome3d (Mosca *et al*, 2013), and three independent software programs, ZDOCK (Pierce *et al*, 2014), Autodock VINA (Trott & Olson, 2010), and ProteinFishing (Cianferoni *et al*, 2020). The results were energetically minimized and ranked based on the buried surface, FoldX (Delgado *et al*, 2019) interaction energy, and FoldX (Delgado *et al*, 2019) stability. An initial filtering step reduced the number of reliable docks to seven (Table EV2). For these, we then performed computational mutagenesis to exclude all models that were incompatible with our experimental data showing LOXL2 binding, which further reduced the candidate models to four (two for BRD4_BD1/LOXL2 and two for BRD4_BD2/LOXL2, Fig EV1D). In all four proposed models, we observed that histidines H626 and H628 of LOXL2, which are required for its catalytic activity (Cuevas *et al*, 2014; Herranz *et al*, 2016), did not participate in the interaction with BRD4 BD1 or BD2 (Fig EV1E). These data corroborated our pulldown results, showing that LOXL2m could still interact with BRD4S (Fig 2B). Two of the four proposed binding models were remarkably similar for BD1 and BD2, and implicated the interaction of LOXL2 with BD1/BD2 AcK binding pockets (models 2 and 4; Fig EV1D) independently of their AcK reader activity (Fig 2E). On the contrary, for models 1 and 3, the interactions between LOXL2 and BD1/BD2 did not involve at all the AcK binding pockets. As expected, adding JQ1 to the docking analysis invalidated models 2 and 4 but did not perturb models 1 and 3 (Fig 2F). To experimentally verify which of the models were correct, we treated MDA-MB-231 cells with JQ1 and performed a BRD4 pulldown experiment. JQ1 treatment strongly reduced BRD4S-LOXL2 interaction (Fig 2G), thus confirming models 2 and 4 as correct.

Overall, these results show that LOXL2 interacts with the short isoform of BRD4 and that the interaction involves the bromodomains' AcK binding pockets but it does not require their AcK reading activity.

LOXL2 and BRD4S control the expression of DREAM target genes

To investigate whether the nuclear BRD4S-LOXL2 interaction plays a role in transcription regulation, we performed ATAC-seq, RNA-seq, and BRD4-ChIP-seq, comparing shControl and shLOXL2 transduced MDA-MB-231 cells (Fig EV2A). The ATAC-seq experiment indicated that LOXL2 KD led to more relaxed chromatin, as expected due to its role in maintaining chromatin compaction (Fig EV2B). Therefore, we initially expected that LOXL2 KD would induce upregulation of gene expression. However, expression changes were equally distributed between up- and downregulated genes (Fig EV2C). Furthermore, despite the increased accessibility, LOXL2 KD did not lead to overall transcriptional activation (Fig EV2D).

We then characterized the functional effects of LOXL2 KD on gene expression. Gene set enrichment analysis (GSEA) revealed that LOXL2 KD induced upregulation of processes involved in cell morphology, secretion, membrane trafficking, and cell differentiation, with *cell–cell junction* being one of the most significantly affected pathways (Fig EV2E). These results agree with the role of LOXL2 in regulating epithelial-to-mesenchymal transition (Millanes-Romero *et al*, 2013; Cuevas *et al*, 2017; Park *et al*, 2017), corroborating the high quality of our dataset. Genes downregulated following LOXL2 KD were instead enriched in the cell cycle signature, specifically DNA duplication (S-phase) and mitotic completion (M-phase) (Fig 3A), suggesting an unexplored role of LOXL2 in controlling cell cycle progression.

We next investigated the differential binding of BRD4S and BRD4L across the genome in shControl and shLOXL2 transduced MDA-MB-231 cells. Given that there is no specific commercial antibody for BRD4S, we adopted a dual ChIP-seq strategy. We performed ChIP-seq using two antibodies: Ab1, which is specific for BRD4L, and Ab2, which recognizes both isoforms. Genomic regions marked by Ab2, but not by Ab1, should be preferentially bound by BRD4S (Fig 3B). We retrieved a total of 2,774 peaks for Ab1 and 3,288 peaks for Ab2, with approximately 20% of the peaks located at promoter regions (Fig EV2F). With these identified promoters, we examined overlaps with gene sets (GSs) in the Molecular Signatures Database (MSigDB) (Liberzon *et al*, 2015) to compare promoter regions differentially bound by the two antibodies. We observed a strong functional overlap in the top 10 GSs identified for each antibody (as expected, given that both antibodies recognize BRD4L) (Fig EV2G). Nevertheless, we identified GSs for Ab2 with a greater gene ratio and a lower adjusted *P*-value (Fig 3C), suggesting that those promoters were preferentially bound by BRD4S. GSs with the greatest adjusted *P*-values and gene ratio differences included *Fisher_DREAM targets*, *RNA binding*, and *Rodrigues_thyroid carcinoma anaplastic up*. Notably, the *Fisher_DREAM targets* GS comprises cell cycle genes that are silenced by the DREAM complex (DREAM: dimerization partner, RB-like, E2F, and multi-vulval class B

Figure 3. LOXL2 and BRD4S regulate DREAM target gene expression.

A Gene Set Enrichment Analysis (GSEA) of the genes downregulated upon LOXL2 KD. The top 20 categories are shown, with the size of the points proportional to the adjusted *P*-values and the distance from the center proportional to the gene ratio.

B Schematic representation of BRD4L and BRD4S illustrating the Ab1 and Ab2 binding sites (top). Schematic representation of the ChIP-seq strategy used to identify BRD4S preferentially bound sites (bottom).

C Overlap of promoter target GSs of Ab1 and Ab2 identified with the MsigDB collections. The size of the points is proportional to the adjusted *P*-values and the distance from the center is proportional to the gene ratio. The adjusted *P*-values are calculated independently for each overlap comparison (Ab1 and Ab2).

D RNA-seq logFC for genes associated with the ChIP-seq peaks of Ab2 in C which fall in promoter regions. The logFC of the subset of these genes which are DREAM targets are plotted in pink (top) and statistical significance is determined by permutation test (bottom).

E BRD4 ChIP-qPCR of DREAM target gene promoters in MDA-MB-231 cells infected with shControl (C), shBRD4 Long (BRD4L KD), and shBRD4 Short (BRD4S KD) isoforms. Data from qPCR were normalized to the input and represented as the fold-change relative to the C condition, which was set as 1. Data are shown as the mean of three independent biological replicates. The standard deviation is shown as error bars. Significance was determined using a one-way ANOVA multiple comparisons with Tukey's correction.

F Genome Browser tracks of four different DREAM target genes containing the following information (from top to bottom): Ab1 ChIP-seq profile, Ab2 ChIP-seq profile either in C or LOXL2 KD conditions, and RNA-seq signal in C and LOXL2 KD conditions.

▶

© 2023 The Authors

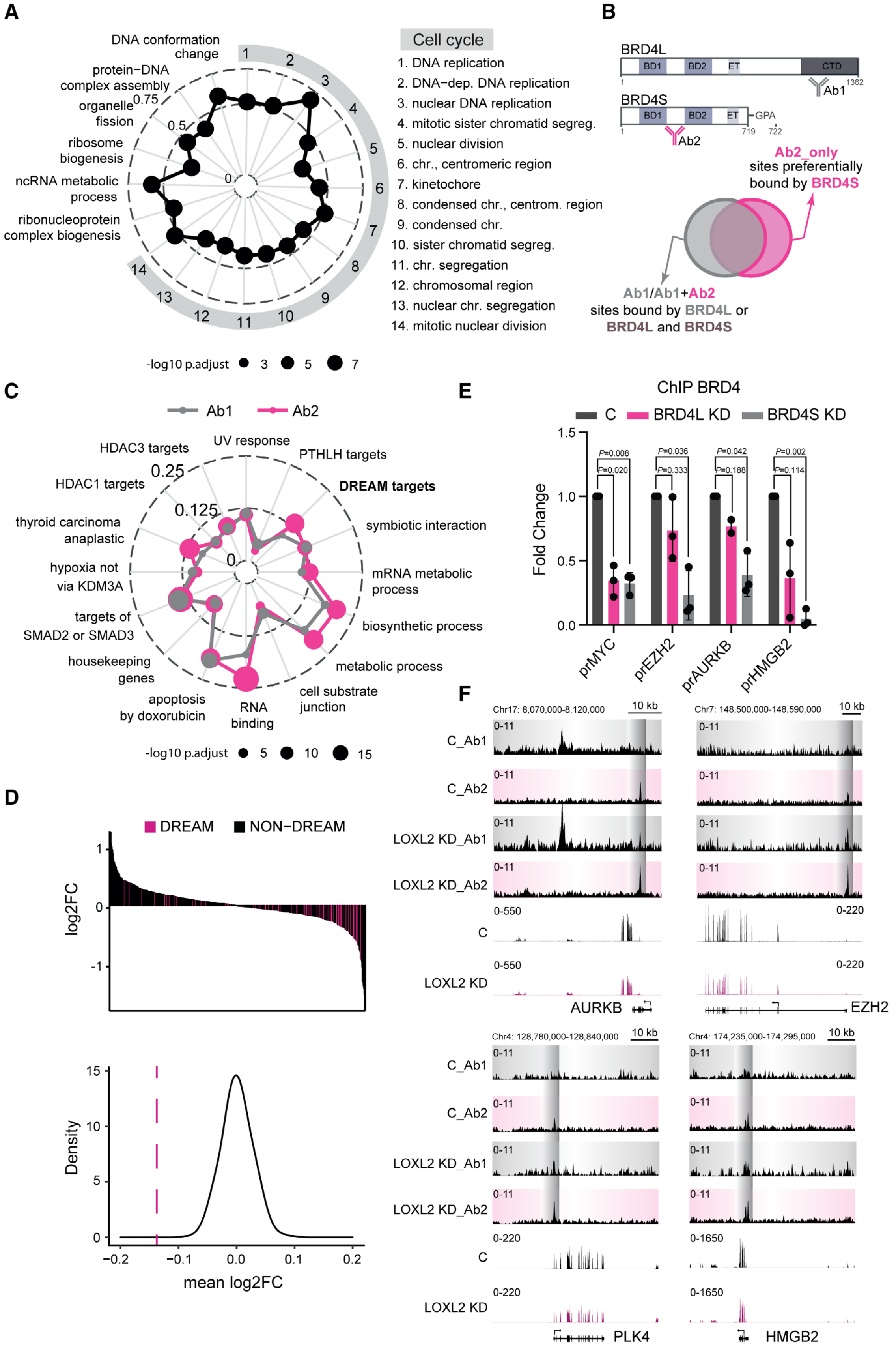
A
Cell cycle
1. DNA replication
2. DNA−dep. DNA replication
3. nuclear DNA replication
4. mitotic sister chromatid segreg.
5. nuclear division
6. chr., centromeric region
7. kinetochore
8. condensed chr., centrom. region
9. condensed chr.
10. sister chromatid segreg.
11. chr. segregation
12. chromosomal region
13. nuclear chr. segregation
14. mitotic nuclear division
DNA conformation change
protein−DNA complex assembly
organelle fission
ribosome biogenesis
ncRNA metabolic process
ribonucleoprotein complex biogenesis
-log10 p.adjust 3 5 7
B
BRD4L
BD1 BD2 ET CTD
Ab1
BRD4S
BD1 BD2 ET GPA
Ab2
Ab2_only sites preferentially bound by BRD4S
Ab1/Ab1+Ab2 sites bound by BRD4L or BRD4L and BRD4S
C
Ab1 Ab2
UV response
PTHLH targets
DREAM targets
symbiotic interaction
mRNA metabolic process
biosynthetic process
metabolic process
cell substrate junction
RNA binding
apoptosis by doxorubicin
housekeeping genes
targets of SMAD2 or SMAD3
hypoxia not via KDM3A
thyroid carcinoma anaplastic
HDAC1 targets
HDAC3 targets
-log10 p.adjust 5 10 15
D
DREAM NON−DREAM
log2FC
Density
mean log2FC
E
ChIP BRD4
C BRD4L KD BRD4S KD
Fold Change
prMYC prEZH2 prAURKB prHMGB2
F
C_Ab1
C_Ab2
LOXL2 KD_Ab1
LOXL2 KD_Ab2
C
LOXL2 KD
AURKB EZH2 PLK4 HMGB2

Figure 3.

complex) (Fischer & Müller, 2017). According to our RNA-seq analysis, LOXL2 downregulation deeply affected the expression of cell cycle genes (Fig 3A). Consequently, the majority of the DREAM target genes retrieved in our Ab2-ChIP-seq were significantly downregulated in the LOXL2 KD condition (Fig 3D), suggesting a functional interaction between BRD4S and LOXL2 to transcriptionally control cell cycle progression. To validate our ChIP-seq strategy, we specifically downregulated either BRD4L or BRD4S (Fig EV2H) and performed ChIP-qPCR on selected DREAM target genes promoter regions using the BRD4 antibody that recognizes both isoforms (Ab2). Notably, the downregulation of BRD4S, but not BRD4L, significantly reduced BRD4 binding to DREAM target gene promoters (Fig 3E). We next checked whether BRD4S and BRD4L differently relocalize on chromatin when LOXL2 is downregulated. We observed a large increase in BRD4 (S/L) binding following LOXL2 KD (Fig EV2I), in line with the increase in chromatin accessibility (Fig EV2J). However, increased BRD4 binding did not significantly correlate with gene expression upregulation (Fig EV2K), suggesting that the increased binding might be a non-functional consequence of the increased chromatin accessibility. We also observed that in the absence of LOXL2, the promoters of DREAM target genes were no longer predominately captured by Ab2 but with both antibodies, indicating either BRD4S-BRD4L co-binding or exclusive BRD4L binding (Figs 3F and EV2L). We hypothesize that the increased signal observed with Ab1 and Ab2 in the LOXL2 KD condition may principally depend on BRD4L binding nonspecifically wherever chromatin becomes more accessible following LOXL2 KD. Indeed, the overlap between the chromatin loci identified with the two antibodies increased in the LOXL2 KD condition (Fig EV2M).

Overall, our transcriptomics and the ChIP-seq analyses suggest that LOXL2 and BRD4S may control the expression of DREAM target genes to transcriptionally regulate cell cycle progression.

LOXL2, BRD4S, and MED1 interact with Lin9 and B-MyB

Next, we investigated the role of LOXL2 on cell cycle progression. Real-time quantitative PCR (qPCR) analysis showed that LOXL2 downregulation reduced the expression of selected DREAM target genes in MDA-MB-231 cells (Fig EV3A), confirming the results of the transcriptomic analysis (Fig 3D). LOXL2 KD significantly reduced the number of cells in the G2-M phase of the cell cycle, indicating a cell cycle progression defect (Fig 4A). High-throughput immunofluorescence (HT-IF) of H3 serine 10 phosphorylation (H3S10p), a typical marker of mitotic entry, confirmed such a defect showing an important reduction of mitotic cells following LOXL2 KD (Figs 4B and EV3B).

We next addressed whether the role of LOXL2 in the regulation of cell cycle progression was dependent on its catalytic activity. The selective LOXL2 inhibitor PXS-5382 (hereafter, PXS) efficiently reduced the levels of oxidized histone H3 (H3K4ox) in MDA-MB-231 cells at 40 μM (Fig EV3C), indicating an efficient inhibition of LOXL2 catalytic activity in the nucleus. When MDA-MB-231 cells were treated with PXS, we observed that the expression of DREAM target genes decreased as in the LOXL2 KD condition (Fig EV3D), suggesting that the catalytic activity of LOXL2 is required for cell cycle transcriptional control. Cell cycle analysis confirmed that PXS treatment of MDA-MB-231 cells significantly decreased the G2-M population, thereby impairing cell cycle progression (Fig 4C). To corroborate the cell cycle role of LOXL2, we performed a time-lapse experiment transducing MDA-MB-231 cells with vectors encoding the mTurquoise2 fluorophore-labeled Stem-Loop Binding Protein (SLBP), a cell cycle-regulated protein that accumulates in the nucleus during G1 and starts to be degraded in G2 (Whitfield *et al*, 2000), and the Maroon1 fluorophore-labeled Histone H1 (required for nuclear identification), and treated treating them either with DMSO or PXS. As expected, DMSO-treated MDA-MB-231 cells progressed into the cell cycle, first acquiring and then progressively losing the accumulation of nuclear SLBP-mTurquoise2. In contrast, PXS-treated cells showed a clear retention of mTurquoise2 nuclear fluorescence (from SLBP), confirming that the treatment impairs cell cycle progression (Figs 4D and EV3E; Movie EV1).

Cell cycle progression relies on the finely tuned transcriptional control of DREAM target genes. The DREAM complex comprises multiple subunits, including the MuvB complex (LIN9, LIN37, LIN52, LIN54, and RBBP), the Rb-like proteins p130 and E2F4, and DP1, and acts as a transcriptional repressor of cell cycle genes. When cells start cycling the DREAM complex disassembles, leaving only the MuvB complex bound to the promoters of cell cycle genes. MuvB coordinates gene expression in the G2/M phases via interacting with the Proto-Oncogene Like 2 (B-Myb) and the Forkhead Box M1 (FOXM1) transcription factors (Sadasivam *et al*, 2012). Interestingly, when performing an unbiased and orthogonal analysis exploring the Achilles dataset (preprint: Dempster *et al*, 2019), we observed that B-Myb scored as differentially top-essential in LOXL2 low-expressing cells as compared to LOXL2 high-expressing cells. The same behavior was observed for several subunits of the Mediator complex (MED1, MED12, MED19, MED24, MED16, MED10, MED9, MED15, MED23, and MED25), which is a well-known BRD4 transcriptional partner. In addition, other BRD4 functionally related genes were differentially essential in LOXL2 low-expressing cells, such as the *de novo* purine synthesis genes PAICS and GMPS (Li *et al*, 2021). Finally, BRD4 itself, the BRD4 target oncogene MYC (Delmore *et al*, 2011; Zuber *et al*, 2011; Xu & Vakoc, 2017; Muhar *et al*, 2018), and the remaining subunits of the mediator complex followed the trend (Fig 4E). These results support the hypothesis that LOXL2 and BRD4 regulate together cell cycle gene expression.

To confirm that LOXL2 and BRD4S control the transcription of cell cycle genes, we performed pulldown experiments for Lin9 (a MuvB subunit member), B-Myb, and FOXM1 using wild-type MDA-MB-231 cells. BRD4S and LOXL2 were pulled down with all three factors. A greater association of BRD4 and LOXL2 with Lin9 and B-Myb was observed compared with FOXM1 (Fig 4F). Only minimal amounts of BRD4L were pulled down with Lin9, B-Myb, or FOXM1 (Fig 4F), corroborating our ChIP-seq results where we observed that DREAM target gene promoters were preferentially bound by BRD4S (Fig 3C and D). Given that several subunits of the Mediator complex scored as highly essential in LOXL2 low-expressing cells (Fig 4E), we investigated whether Mediator may be involved in the regulation of cell cycle gene expression together with BRD4S and LOXL2. By using a MED1 antibody as a proxy for the Mediator complex, we showed that, as observed for LOXL2 and BRD4S, MED1 preferentially interacted with Lin9 and B-Myb. These results overall suggest that BRD4S, LOXL2, and MED1 interact with the MuvB complex and B-Myb to promote the transcription of cell cycle genes.

 © 2023 The Authors

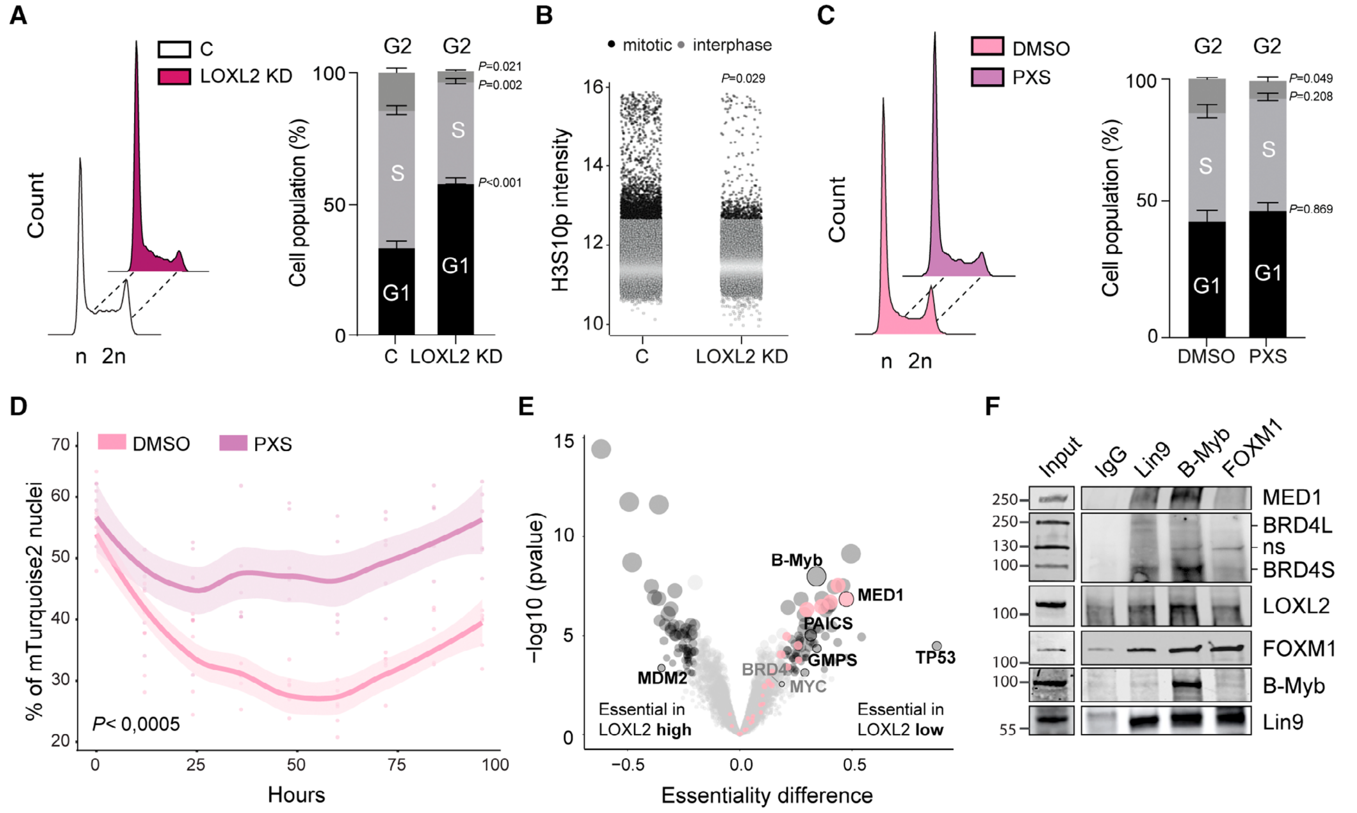

Figure 4. LOXL2 repression leads to G1-S delay.

A Representative cell cycle profile of MDA-MB-231 cells infected either with C or LOXL2 KD. DNA content was analyzed by FACS following propidium iodide (PI) staining (left). The percentage of cells in each phase of the cell cycle was quantified using the FlowJo Software (right). Data are shown as the mean of three independent biological replicates. The standard deviation is shown as error bars. Significance was determined by unpaired Student's *t*-test.

B High-throughput immunofluorescence of H3S10p mitotic marker in C or LOXL2 KD MDA-MB-231 cells. Mitotic cells (black dots) showed on average a higher H3S10p signal than the population median + 3S.D. Interphase cells are represented with gray dots. H3S10p intensity is represented as the normalized median. Significance was calculated using an unpaired Student's *t*-test and is based on the mitotic index of the two populations.

C Representative cell cycle profile of MDA-MB-231 cells treated either with DMSO or 40 μM of PXS for 96 h. DNA content was analyzed by FACS following propidium iodide (PI) staining (left). The percentage of cells in each phase of the cell cycle was quantified using the FlowJo Software (right). Data are shown as the mean of three independent biological replicates. The standard deviation is shown as error bars. Significance was determined by unpaired Student's *t*-test.

D MDA-MB-231 cells expressing SLBP-mTurquoise2 and H1-Maroon1 were treated with DMSO or 40 μM of PXS for 96 h. The percentage of mTurquoise2 nuclei in each well is shown, representing cells in G1-S. The difference between the PXS and DMSO-treated cells was significant ($P < 0.005$) under a linear model comparing the percentage of mTurquoise2-positive cells, across time, in the two conditions. The estimated increase following treatment with PXS of the area-under-the-curve (AUC) is 13.3 au. Significance was calculated by the Student's *t*-test on AUC. Quantification was performed every 12 h. $N = 200$ cells/replicate, with six biological replicates.

E Differential gene essentiality between high and low LOXL2-expressing cell lines (CCLE) as calculated by analyzing the Achilles dataset. Cell lines with low LOXL2 expression are significantly more sensitive to the depletion of genes represented in the right part of the X-axis as compared to cell lines with high LOXL2 expression, which are more sensitive to the depletion of genes represented in the left part of the X-axis. $N = 80$ cell lines. Significance was determined using the Student's *t*-test with BH multiple hypothesis correction. Significant threshold is based on adjusted P-value < 0.05; black dots represent significant essentialities and pink dots represent different mediator subunits. The dot size is proportional to the adjusted P-value of each gene.

F Pulldown of endogenous Lin9, B-Myb, or FOXM1 in MDA-MB-231 cells. Precipitates were analyzed by Western blot with the indicated antibodies. Irrelevant IgGs were used as a negative control; ns: non-specific. Three biological replicates were performed.

Source data are available online for this figure.

The stability of BRD4-MED1 transcriptional foci requires BRD4 and LOXL2 catalytic activity and regulates cell cycle transcriptional control

To further characterize the relationship between LOXL2, BRD4S, and MED1, we queried whether MED1 also interacted with LOXL2, if this interaction required the catalytic activity of LOXL2, and whether LOXL2 inhibition impaired the interaction between BRD4 and MED1. When MDA-MB-231 cells were treated with either DMSO or PXS, we observed that MED1 interacted with LOXL2 in a catalytic-dependent manner (Fig 5A and C). However, the PXS treatment did not perturb the MED1-BRD4 interaction (S and L), nor the BRD4S-LOXL2 interaction (Fig 5B and C), as expected based on the docking results (Fig EV1E). We previously showed that JQ1 abrogated the BRD4S-LOXL2 interaction (Fig 2G), as predicted by the docking poses 2 and 4 (Fig 2F). We, therefore, tested if JQ1

treatment impaired the MED1-LOXL2 or the MED1-BRD4 interactions. We performed a MED1 pulldown in cells treated with either DMSO or JQ1 and showed that while the MED1-LOXL2 interaction was not affected, BRD4S-MED1 interaction decreased, leaving unperturbed the BRD4L-MED1 interaction (Fig 5D and F). BRD4 pulldown confirmed the loss of BRD4S-LOXL2 interaction in the presence of JQ1 (Fig 5E and F).

It is known that BRD4 interacts with MED1 (Jang *et al*, 2005; Lambert *et al*, 2019) and that the BRD4-MED1 interaction underpins the formation of nuclear transcriptional foci (Sabari *et al*, 2018) that decorate super-enhancers and boost the expression of downstream target genes (Lovén *et al*, 2013; Quevedo *et al*, 2019). Recently, it has been shown that BRD4S is crucial for the formation of such transcriptional foci (Han *et al*, 2020). Thus, we asked whether LOXL2 KD in our ChIP-seq dataset caused a decrease in the binding of BRD4S to super-enhancers associated with DREAM target genes. After calling super-enhancers with Rank Ordering of Super-Enhancers (ROSE) (Lovén *et al*, 2013; Whyte *et al*, 2013), we determined that of the peaks retrieved with the antibody specific for BRD4L (Ab1), only 3.1% was associated to super-enhancers. In contrast, 8% of the peaks retrieved with the antibody recognizing both BRD4 isoforms (S/L; Ab2) were located at super-enhancers (Appendix Fig S4A), suggesting a prominent role for BRD4S in super-enhancer formation. Additionally, the percentage of Ab1-bound super-enhancers associated with DREAM target genes only mildly decreased in the LOXL2 KD condition (2 vs. 1.7%), while the percentage of Ab2-bound super-enhancers associated with DREAM target genes considerably dropped (3.9 vs. 2.7%), confirming a predominant role of BRD4S controlling the formation of these cell cycle-related super-enhancers (Appendix Fig S4B). We, therefore, hypothesized that the downregulation of cell cycle genes observed with LOXL2 repression may be due to a partial destabilization of BRD4S-MED1 transcriptional foci, which are a visual proxy for super-enhancer formation. Given the fact that BRD4S, LOXL2, and MED1 interact with each other and that the inhibition of either BRD4 or LOXL2 partially affects these interactions, we reasoned that combining the treatments (PXS and JQ1) would strongly destabilize the formation of this newly described transcriptional complex, leading to a strong reduction of BRD4-MED1 transcriptional foci and consequential repression of cell cycle gene expression. MDA-MB-231 cells treated either with DMSO, PXS, JQ1, or the combination of both inhibitors (combo) were used for HT-IF to analyze changes in BRD4 and MED1 nuclear foci. The intensity of BRD4 and MED1 foci did not change with any treatment (Appendix Fig S4C and D). However, each treatment showed a decrease in BRD4 foci number, being the combo significantly stronger than single treatments (Fig 5G and H). Additionally, even though the number of MED1 foci was not altered with any treatment (Appendix Fig S4E), we observed a reduction of BRD4-MED1 colocalization in the combo treatment (Fig 5G and I). We, therefore, tested whether the combo-driven reduction of BRD4 nuclear foci and BRD4-MED1 colocalization would compromise the transcription of cell cycle genes to a greater extent than either PXS or JQ1 alone. For this, we performed qPCR analysis of selected DREAM target genes comparing PXS, JQ1, and the combo. As previously observed (Fig EV3D), the PXS treatment decreased the expression of such genes, and the JQ1 treatment showed even a higher effect. The combo treatment, however, almost completely shut down their expression clearly displaying a superior effect (Fig 5J).

These results indicate that the transcriptional complex containing BRD4S, LOXL2, and MED1 is only partially affected by inhibiting either BRD4 or LOXL2, while the combo treatment can dismantle it. Additionally, these data reveal a novel mechanism by which the interactions between BRD4S and LOXL2 are required for the

Figure 5. LOXL2 and BRD4 inhibition induces BRD4-MED1 transcriptional foci disintegration.

- A MED1 pulldown in MDA-MB-231 cells treated with DMSO or 40 μM of PXS for 96 h. Precipitates were analyzed by Western blot with the indicated antibodies. Irrelevant IgGs were used as a negative control; ns: non-specific. Three biological replicates were performed.
- B BRD4 pulldown in MDA-MB-231 cells treated with DMSO or 40 μM of PXS for 96 h. Precipitates were analyzed by Western blot with the indicated antibodies. Irrelevant IgGs were used as a negative control; ns: non-specific. Three biological replicates were performed.
- C Schematic representation of the interactions between BRD4S, LOXL2, and MED1 after LOXL2 inhibition with PXS.
- D MED1 pulldown in MDA-MB-231 cells treated with DMSO or 5 μM of JQ1 for 24 h. Precipitates were analyzed by Western blot with the indicated antibodies. Irrelevant IgGs were used as a negative control; ns: non-specific. Three biological replicates were performed.
- E BRD4 pulldown in MDA-MB-231 cells treated with DMSO or 5 μM of JQ1 for 24 h. Precipitates were analyzed by Western blot with the indicated antibodies. Irrelevant IgGs were used as a negative control; ns: non-specific. Three biological replicates were performed.
- F Schematic representation of the interactions between BRD4S, LOXL2, and MED1 after BRD4 inhibition with JQ1.
- G Representative images of BRD4 (green) and MED1 (red) high-throughput immunofluorescence of MDA-MB-231 cells treated for 96 h with DMSO, 40 μM of PXS, 5 μM of JQ1 or the combination of both inhibitors (Combo). DAPI (blue) was used as a nuclear marker. Scale bar; 5 μm. Magnifications of representative foci are also depicted.
- H Quantification of the number of BRD4 foci from (G) corrected by the nucleus area. Results are normalized to DMSO. Four biological replicates were performed using at least 4,000 nuclei/replicate for the analysis. Data are shown as the mean of four independent biological replicates. The standard deviation is shown as error bars. Significance was calculated using a one-way ANOVA multiple comparisons with Tukey's correction test.
- I Quantification of the percentage of MED1 overlapping with BRD4 occupied area from (G). Four biological replicates were performed using at least 4,000 nuclei/replicate for the analysis. Data are shown as the mean of four independent biological replicates. The standard deviation is shown as error bars. Significance was calculated using a one-way ANOVA multiple comparisons with Tukey's correction test.
- J Real-time quantitative PCR (qPCR) showing the changes in mRNA expression of four selected DREAM target genes (*EZH2*, *HMGB2*, *AURKB*, and *PLK4*) in MDA-MB-231 cells treated with DMSO, 40 μM of PXS, 5 μM of JQ1 or the combination of both inhibitors (Combo) for 96 h. Gene expression was normalized against an endogenous control (Pumilio homolog 1) and represented as the expression relative to the DMSO condition, which was set as 1. Data are shown as the mean of three independent biological replicates. The standard deviation is shown as error bars. Significance was calculated using a one-way ANOVA multiple comparisons with Tukey's correction test.
- K Schematic representation of the proposed molecular mechanism.

Source data are available online for this figure.

 © 2023 The Authors

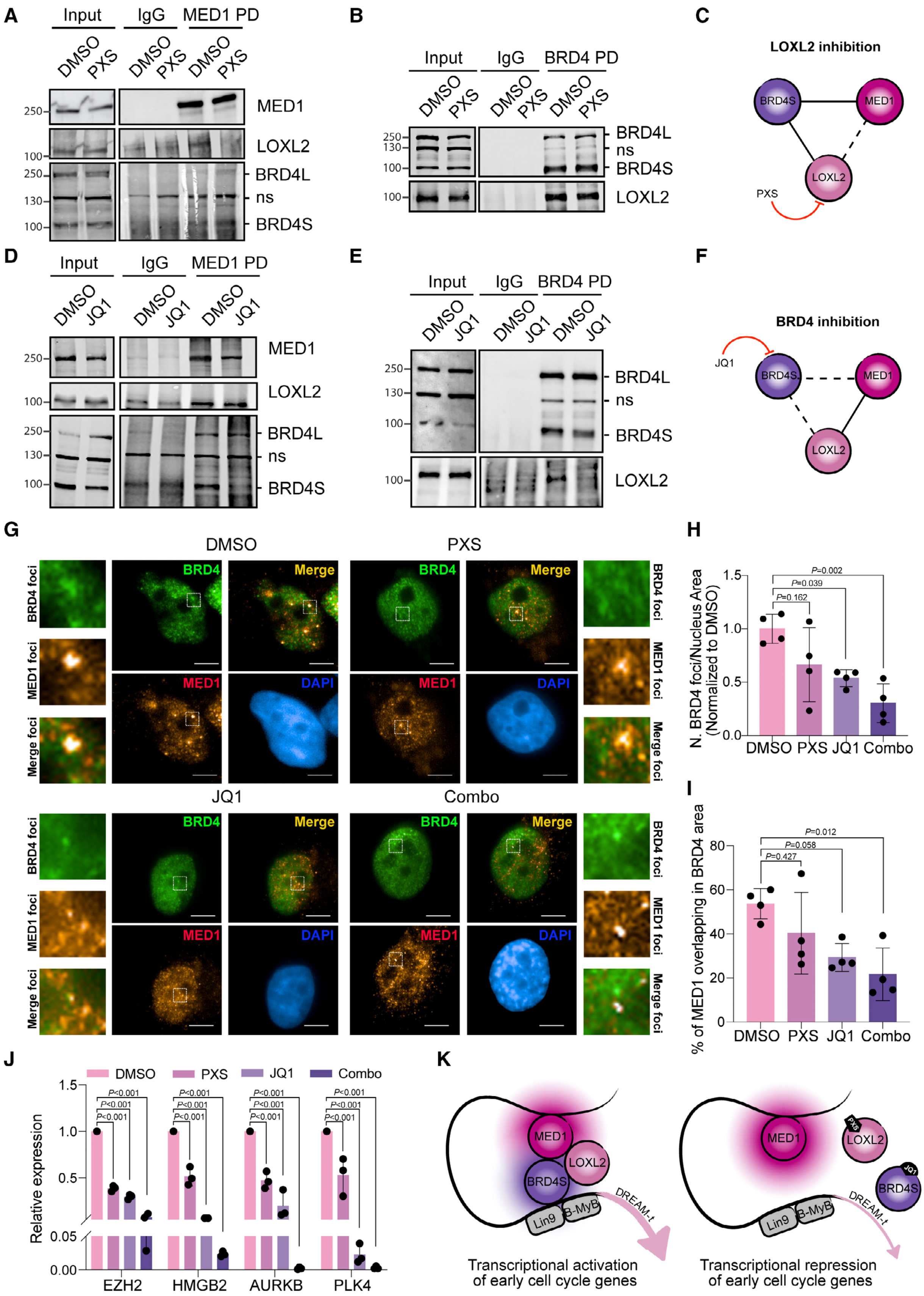
A
Input IgG MED1 PD
DMSO PXS DMSO PXS DMSO PXS
MED1
LOXL2
BRD4L
ns
BRD4S
B
Input IgG BRD4 PD
BRD4L
ns
BRD4S
LOXL2
C
LOXL2 inhibition
BRD4S MED1 LOXL2 PXS
D
Input IgG MED1 PD
DMSO JQ1 DMSO JQ1 DMSO JQ1
E
Input IgG BRD4 PD
F
BRD4 inhibition
JQ1 BRD4S MED1 LOXL2
G
DMSO PXS JQ1 Combo
BRD4 Merge MED1 DAPI
BRD4 foci MED1 foci Merge foci
H
N. BRD4 foci/Nucleus Area (Normalized to DMSO)
P=0.002 P=0.039 P=0.162
DMSO PXS JQ1 Combo
I
% of MED1 overlapping in BRD4 area
P=0.012 P=0.058 P=0.427
DMSO PXS JQ1 Combo
J
DMSO PXS JQ1 Combo
Relative expression
P<0.001
EZH2 HMGB2 AURKB PLK4
K
MED1 LOXL2 BRD4S Lin9 B-MyB DREAM-t
Transcriptional activation of early cell cycle genes
PXS JQ1
Transcriptional repression of early cell cycle genes

Figure 5.

formation of BRD4-MED1 transcriptional foci and the gene expression regulation of cell cycle genes (Fig 5K).

The simultaneous inhibition of BRD4S and LOXL2 compromises TNBC proliferation

Given the strong effect of the combo treatment on the expression of cell cycle genes, we wondered whether we could use it as a strategy to efficiently suppress the proliferation of TNBC. We first investigated *in vitro* whether inhibiting LOXL2 and BRD4 either alone or together would have a differential effect on TNBC cell growth. Three different TNBC cell lines were treated for 96 h either with DMSO or increasing concentration of PXS and JQ1, either alone or in combination. PXS and JQ1 reduced cell proliferation in all the cell lines tested. In the cell lines MDA-MB-468 and BT-549, which have very low levels of LOXL2 or BRD4, respectively (Fig 1C), we observed that the combo treatment showed only an additive effect, as expected due to partial lack of either target. In contrast, in the cell line MDA-MB-231, which has high levels of BRD4 and LOXL2 (Fig 1C), the combo treatment synergistically suppressed cell proliferation (Figs 6A and EV4A). In order to further demonstrate the critical role of the short isoform of BRD4 in controlling TNBC cell proliferation together with LOXL2, we transduced MDA-MB-231 cells with isoform-specific shBRD4s (BRD4S KD and BRD4L KD). Only BRD4S KD provoked a significant reduction of cell viability following PXS treatment (Fig EV4B), confirming our hypothesis. Additionally, we tested the combinatorial treatment in a Cal51, a TNBC cell line expressing high levels of LOXL2 and BRD4 (Fig EV4C) and displaying a superior resistance to JQ1 treatment than MDA-MB-231 (Fig EV4D). Also, in Cal51 we observed that the combo treatment impacted cell viability to a greater extent than the single agent treatments (Fig EV4E), reinforcing the role of the BRD4S-LOXL2 interaction in controlling TNBC proliferation.

We further examined whether the combo treatment would have a synergistic effect *in vivo* by orthotopically implanting MDA-MB-231 cells into the mammary glands of immunodeficient mice (NOD-SCID). After tumor formation (at approximately 100 mm^3 tumor volume), mice were treated with JQ1, PXS, combo, or vehicle. The PXS and JQ1 single treatments delayed tumor growth *in vivo* (by approximately 33 and 23%, respectively, at the endpoint); however, the combo treatment was much more effective, reaching a 78% reduction at the endpoint (Fig 6B). The MDA-MB-231 cell line has high metastatic capacity when orthotopically implanted (Price *et al*, 1990), and histopathological analyses of lung sections from vehicle-treated mice showed multiple tumor metastatic foci. In contrast, no metastatic foci were observed in the PXS-treated or combo-treated mice (Fig 6C), confirming the potential antimetastatic effects of LOXL2 inhibition (Salvador *et al*, 2017). JQ1 treatment was also highly anti-metastatic (one metastatic nodule was observed in the analyzed samples) (Fig 6C). Importantly, none of the treatments (including the combo treatment) had significant toxicity in mice (Fig EV5A).

Next, we tested whether the combination of PXS and JQ1 was effective in a previously established TNBC PDX model (PDX-549) from EuroPDX (www.europdx.eu), in which we detected moderate protein levels of both BRD4 and LOXL2 (Fig EV5B). Similar to what we observed in the MDA-MB-231 orthotopic xenograft model, the combination of PXS and JQ1 in the TNBC PDX model also showed a superior antitumor effect compared to single-agent treatments (50% with JQ1, no effect with PXS and 70% with the combo) (Fig EV5C). Again, no significant toxicity was observed in TNBC tumor-bearing mice treated with either PXS or JQ1, alone or in combination (Fig EV5D).

We, therefore, checked the expression of LOXL2 and BRD4 in 13 additional PDXs and observed that LOXL2 was clearly expressed in 11 out of 13 (PDX-44T, 94, 98T, 124, 127, 230, 270, 377, 457b, 509, and 533). While BRD4S was also detected in 12 PDXs (PDX-44T, 94, 98T, 124, 127, 230, 270, 341, 377, 457b, 473b, and 533), BRD4L was barely expressed in all the PDXs and could be clearly detected only in 6 (PDX-98T, 230, 270, 377, 457b, and 533) (Fig EV5E). Interestingly, the majority of the analyzed PDX showed co-expression of BRD4S and LOXL2 (10 out of 13), suggesting that co-targeting BRD4S and LOXL2 could be a valid therapeutic approach for the majority of TNBC. We therefore selected a second PDX (127) based on low doubling time (Fig EV5F), which is indicative of high proliferation capacity, and lack of BRD4L expression (Fig EV5E), which made the JQ1 treatment specific for BRD4S. We further challenged our model by reducing by half the dose of JQ1, since in the PDX-549 we observed that the tested concentration showed already a significant reduction of tumor growth when given alone (Fig EV5C). Even in this utmost situation, the combo treatment showed a stronger effect (reaching a 50% reduction at the 15-day end time point) than the single treatments alone, which in this case had only minimal effect (no effect in PXS and only 20% reduction in JQ1 treatment) (Fig 6D). As expected, no significant toxicity was observed in mice (Fig EV5G). We wondered whether the observed reduction in tumor volume was the result of a cytostatic and/or cytotoxic effect. We performed immunohistochemistry for KI67, and H3S10p, which mark respectively actively replicating cells and mitotic cells, and observed that the combo treatment was significantly reducing both stains at superior levels than the single treatments (Fig 6E and Appendix Fig S5A). Further, we analyzed Caspase3 immunostaining and observed that the combo treatment also increased apoptosis significantly higher than each single treatment (Appendix Fig S5A and C).

Targeting lysyl oxidase (LOX) proteins inhibits the FAK/Src signaling cascade and re-sensitizes cells to chemotherapy treatment in TNBC-resistant models (Saatci *et al*, 2020). Since the FAK/Src cascade promotes the G1-S transition (Pickup *et al*, 2014), we wanted to exclude that the observed tumor growth defects in the combo treatment were arising from FAK or Src direct inhibition. Western blot analysis of excised tumors did not show a reduction in activated FAK or Src upon any treatment (Appendix Fig S5D), thus discarding this possibility. Besides, in the extracellular matrix, LOXL2 plays a role in crosslinking collagen fibers, which promotes tumor fibrosis and metastatic potential (Pickup *et al*, 2014; Añazco *et al*, 2016). Collagen staining of tumor samples from MDA-MB-231 and PDX-127 xenografted mice did not show changes following any treatment, again discarding that the observed antiproliferative effect was due to alterations of the collagen in the extracellular matrix (Appendix Fig S5E and F).

Overall, these data indicate that the simultaneous inhibition of BRD4S and LOXL2 successfully reduces tumor growth by suppressing TNBC cell proliferation and inducing cell death, holding exciting potential for the development of future clinical applications.

© 2023 The Authors

Discussion

Approximately 15% of breast cancers are classified as TNBC, which are highly heterogeneous, show an aggressive phenotype, and have an unfavorable prognosis. Given that there is no targeted therapy to treat them, the standard regimen for TNBC treatment relies on the use of conventional chemotherapeutic agents, yet this strategy in most cases fails to arrest TNBC proliferation. In this study, we report a newly discovered mechanism for controlling TNBC proliferation, which can be exploited to develop successful TNBC treatment. Recently, LOXL2 has been shown to be implicated in the proliferation of several solid tumors (Ahn *et al*, 2013; Tanaka *et al*, 2018; Cao *et al*, 2020), including TNBC (Cebrià-Costa *et al*, 2020). Therefore, we sought to explore the possibility of combining LOXL2

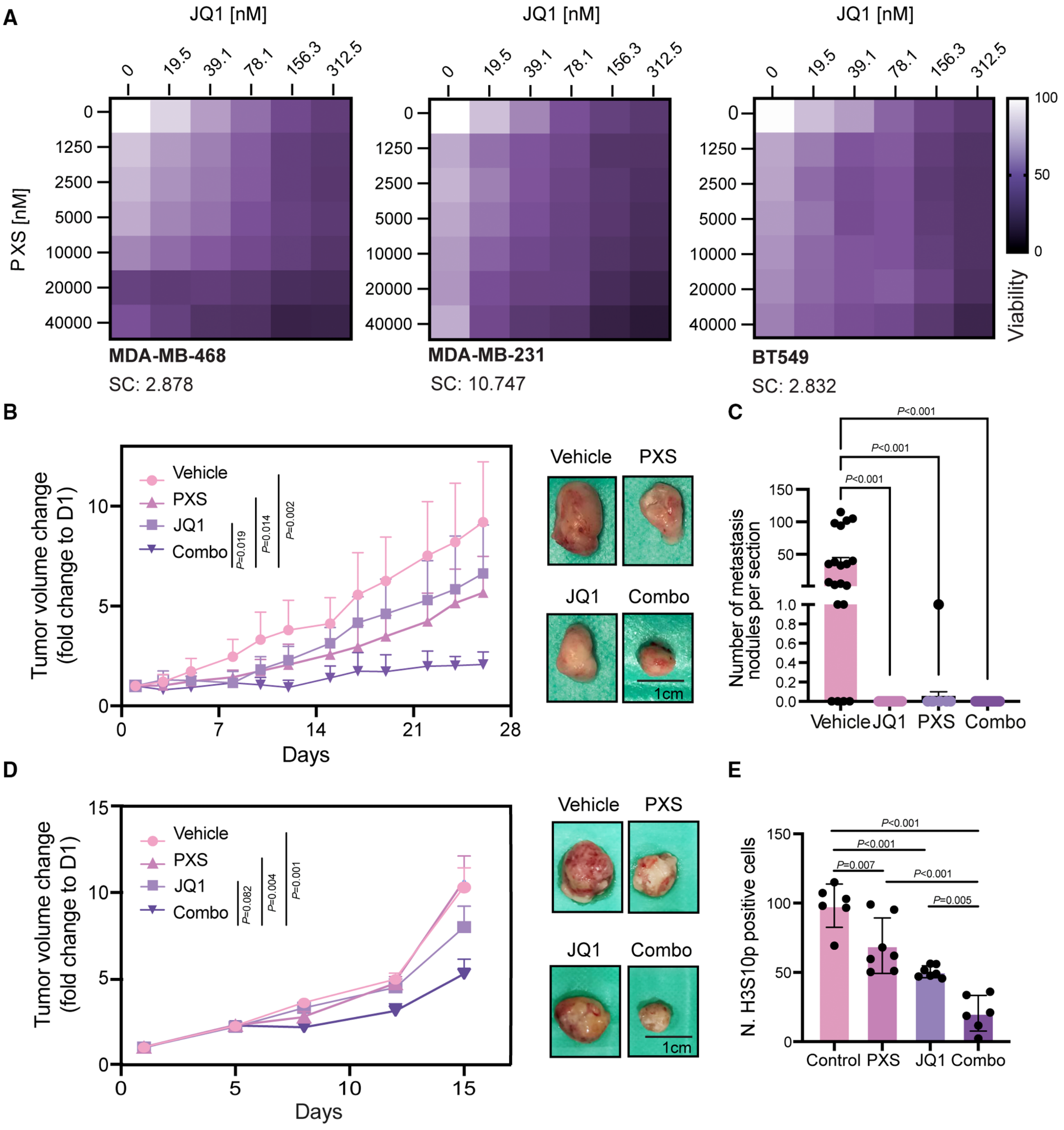

Figure 6.

Figure 6. BRD4 and LOXL2 simultaneous inhibition impairs *in vivo* TNBC progression.

A Representative synergy matrixes showing cell viability measured with the MTT assay. The three TNBC cell lines used were treated with either PXS or JQ1 alone or with their combination at the indicated concentration for 96 h. SC indicates the synergy score for each cell line; a synergy score lower than 5 indicates the additive effect of the treatments, while a synergy score higher than 5 indicates synergism (Love *et al*, 2014). Three biological replicates were analyzed for each cell line.

B Tumor volumes represented as fold change to day 1 (D1) from the MDA-MB-231 xenograft mice treated five times per week with 15 mg/kg JQ1 and/or 2 mg per pump PXS during 26 days. A minimum of six tumors per group (3 mice/both sides) are shown as average tumor volume and standard deviations are shown as error bars. The significance was determined at the endpoint (day 26) using a two-way ANOVA multiple comparisons with Tukey's correction test (left). Images of the excised tumors at the end of the experiment (day 26) (right).

C Quantification of the number of metastasis nodules per mouse lung section analyzed. Data are shown as the mean of at least 15 lung sections analyzed per group. The standard deviation is shown as error bars. Significance was calculated using a one-way ANOVA multiple comparisons with Dunnett's correction test.

D Tumor volumes represented as fold change to day 1 (D1) from PDX-127 mice treated five times per week with 7.5 mg/kg JQ1 and/or 2 mg per pump PXS for 15 days. A minimum of six tumors per group (3 mice/both sides) are shown as average tumor volume. Standard deviations are shown as error bars. Significance was determined at the endpoint (day 15) using a two-way ANOVA multiple comparisons with Tukey's correction test (left). Images of the excised tumors at the end of the experiment (day 15) (right).

E Quantification of the number of H3S10p positive cells stained by immunohistochemistry in each of the excised tumors. A minimum of six tumors were analyzed per group and three different regions per tumor were quantified. The standard deviation is shown as error bars. Significance was calculated using a one-way ANOVA multiple comparisons with Tukey's correction test.

Source data are available online for this figure.

inhibition with conventional chemotherapy. However, LOXL2 expression fails to predict the response of TNBC to common chemotherapeutic treatments. BETi are tested in over 30 clinical trials, five of them centered in TNBC. Excitingly, LOXL2 expression could very well predict BETi treatment outcome, being LOXL2 low expressing cells more sensitive to the treatment (Fig 1A). Further, we found that BRD4 and LOXL2 are physical and functional interactors. In the nucleus, LOXL2 binds specifically to the short isoform of BRD4 (BRD4S) (Fig 2B) that in TNBC has been recently describe as oncogenic, but not to the long one (BRD4L), which has a tumor suppressive role (Wu *et al*, 2020). Genetic or pharmacological repression of LOXL2 downregulates the expression of cell cycle genes (Figs 3A and D, and EV3A and D), whose promoter is bound by BRD4S (Fig 3C). LOXL2 was previously reported as a transcriptional repressor (Iturbide *et al*, 2015a). Here, we report for the first time that in TNBC, LOXL2 acts as a transcriptional activator of cell cycle genes.

When scouting for vulnerabilities associated with LOXL2 low expression, we found that LOXL2 low expressing cells are much more sensitive to the loss of B-Myb, which controls the expression of cell cycle genes, and BRD4 functional partners, including the Mediator complex (Figs 4E and EV3F). We showed that MED1 interacts with LOXL2 and BRD4S (Fig 5A and D). Such trimeric complex can be dismantled only by inhibiting BRD4 and LOXL2 simultaneously, which molecularly induces loss of BRD4 transcriptional foci, BRD4-MED1 colocalization, and a remarkable downregulation of cell cycle genes (Fig 5G–J). Interestingly, while LOXL2 has never been associated with cell cycle transcriptional control, BRD4 has been previously linked to the regulation of cell cycle gene expression (Dey *et al*, 2003; Yang *et al*, 2008).

At the phenotypic level, we showed that the combinatorial inhibition of BRD4 and LOXL2 is synergistic *in vitro* and *in vivo* in suppressing TNBC proliferation (Fig 6). Importantly, the three *in vivo* models that we tested (one cell line implantation, and two PDXs models with different aggressiveness) all showed comparable results, indicating strong consistency. The vast majority of the PDXs assessed in our study showed moderate to high levels of BRD4S and LOXL2, while BRD4L was barely expressed (Fig EV5B and E). This evidence not only confirms the pro-oncogenic function of BRD4S (Wu *et al*, 2020) but also indicates that the simultaneous inhibition of LOXL2 and BRD4 could be explored as a strategy for the treatment of TNBC.

BRD4 inhibition has been found to increase the activity of the oncosuppressor TP53 (Latif *et al*, 2021). The observed antiproliferative effect in MDA-MB-231cells, however, cannot be attributed to the direct activation of TP53 given its mutated state in this cell line, but rather to the activation of alternative cytotoxic or cytostatic routes (Webber *et al*, 2019). Interestingly, the essentiality analysis that we conducted revealed that LOXL2-low expressing cells are very sensitive to the loss of TP53, and conversely, they survive better if MDM2 (which is required to induce TP53 proteasomal degradation) is absent (Figs 4E and EV3F). This evidence is in line with previous results indicating that the loss of LOXL2 in TNBC enhances DNA damage, and may indicate that cells with low levels of LOXL2 require a resilient mechanism to guarantee genome integrity and avoid apoptosis. Excitingly, BRD4 has also been associated multiple times with the DNA damage response (Pongas *et al*, 2017; Zhang *et al*, 2018). Mechanistically, it has been suggested to be responsible for insulating chromatin at DNA-damaged sites, thereby allowing repair (Floyd *et al*, 2013), or preventing the accumulation of R-loops and thereby protecting against transcription–replication collision (Lam *et al*, 2020). The role of LOXL2 and BRD4 in DNA damage and their cooperation in controlling the transcriptional regulation of cell cycle progression may suggest that their simultaneous inhibition in tumor cells could act as a double-edged sword. In line with this, combining LOXL2 and BRD4 inhibition with DNA-damaging agents could further improve the outcome of TNBC treatment.

The potential of LOXL2 as a biomarker is noteworthy. In this manuscript, we show that low LOXL2 levels sensitize cells to BETi treatments. This represents a great advantage in the clinical field because we can mimic low LOXL2 levels by treating with inhibitors, and effectively stop tumor growth by co-treating with BETi. However, BETi are not yet approved in the clinic, although there are clinical trials even for TNBC. On the other hand, Vinca alkaloid compounds are used as standard therapy for many types of cancer, including TNBC. In this manuscript, we also show that LOXL2 expression levels can predict the outcome of Vinca alkaloid treatments (Fig 1A), thus reinforcing the role of LOXL2 as a biomarker that could be readily implemented in the clinic. Finally, although we

 © 2023 The Authors

have focused our study on TNBC, it would be interesting to extrapolate our findings to other cancer types to find out whether the same strategy would be effective in counteracting their growth.

Materials and Methods

Cell culture

HEK293T cells (ATCC; #CRL-3216), MDA-MB-231 cells (ATCC; #HTB-26), MDA-MB-468 (ATCC; #HTB-132), and BT-549 (ATCC; #HTB-122) were cultured in Dulbecco's modified Eagle's medium (DMEM; Biowest; #L0106-500) supplemented with 10% FBS (Gibco; #10270106) at 37°C in 5% CO_2. To verify that no mycoplasma contamination was present, cultures were tested every month. Pre-authenticated cell lines used in this study were obtained from the CRG cell line collection.

Plasmids

To obtain the human LOXL2 (hLOXL2) plasmid with point mutations in either the K209 or K209-K212 (Table 1), a GeneBlock (IDT) was designed and cloned with K209R and K209Q/K212Q mutations. For this, pcDNA3-hLOXL2 (2 µg) was digested with EcoRI and HindIII (New England Biolabs) in Cutsmart buffer for 2 h, and the digested plasmid was gel purified. Each GeneBlock containing the point mutations was cloned using the Gibson reaction approach for 1 h at 50°C. DH5α *E. coli* cells were transformed with 3 µl of Gibson reaction solution, and single colonies were grown in LB medium with ampicillin for further analysis. Sanger sequencing was performed to select positive clones.

Table 1. List of plasmids used in the study.

Plasmids	Source	Purchase no.
pcDNA3-hLOXL2wt-Flag	Herranz *et al* (2016)	N/A
pcDNA3-hLOXL2m-Flag	Herranz *et al* (2016)	N/A
Empty-pcDNA3	Herranz *et al* (2016)	N/A
pcDNA3-hLOXL2R-Flag	This manuscript	N/A
pcDNA3-hLOXL2Q-Flag	This manuscript	N/A
BRD4L-GFP	Sdelci *et al* (2019)	N/A
BRD4S-GFP	Sdelci *et al* (2019)	N/A
BRD4S-N140F/N433F-GFP	Sdelci *et al* (2019)	N/A
BRD4_BD1-GFP	Sdelci *et al* (2019)	N/A
BRD4_BD1/BD2-GFP	Sdelci *et al* (2019)	N/A
BRD4_BD2-GFP	Sdelci *et al* (2019)	N/A
BRD4S-specific C-terminal-GFP	Sdelci *et al* (2019)	N/A
BRD4 Long shRNA	This manuscript	N/A
BRD4 Short shRNA	This manuscript	N/A
pLL3.7 m-mTurquoise2-SLBP(18-126)-IRES-H1-mMaroon1	Addgene	#83842
CT shRNA	Sigma-Aldrich	#SHC002V
LOXL2 shRNA	Sigma-Aldrich	#TRCN0000046196

In order to knockdown the long and short BRD4 isoforms (BRD4L KD and BRD4S KD, respectively) (Table 1), two targeting shRNAs were cloned in plKO plasmid. The vector (1.5 µg) was digested with EcorI-HF (NEB, R3101) and AgeI-HF (NEB, R3552) in rCutsmart Buffer and the resulting product was gel purified with the QIAquick Gel Extraction Kit (Qiagen, 28706). To obtain the shRNA, each oligo (IDT) (Table 2) was resuspended to 20 µM in molecular biology water and 5 µl each were annealed in NEBuffer 2 (B7002S) for 4 min at 95°C and slowly cool down to room temperature for several hours. The vector and both inserts were ligated with 1 µl of NEB T4 DNA ligase (NEB, M0202) and 2 µl of 10× NEB T4 DNA ligase buffer in molecular biology water at 16°C overnight. The resulting ligation product was transformed in 25 µl of DH5a Competent Cells (Thermo Fisher Scientific, 18265017) and selected in 100 µg/ml in house ampicillin plates.

The sequences of the primers used were obtained from Broad Institute GPP Web Portal (https://portals.broadinstitute.org/gpp/public/) and are listed in the Table 2.

Transfection

For overexpression assays, MDA-MB-231, MDA-MB-468, or HEK293T cells were seeded for 24 h and transiently transfected with the indicated vectors using either polyethyleneimine polymer (Polysciences Inc; #23966-1) or TransIT-X2 Dynamic Delivery System (Mirus Bio; #MIR6004), following the manufacturer's instructions.

Lentiviral infection

HEK293T cells were used to produce lentiviral particles. Cells were grown to 70% confluence (day 0) and transfected by adding dropwise a mixture of 150 mM NaCl, DNA (50% of either control shRNA [SHC002V] or LOXL2 shRNA [TRCN0000046196], BRD4L shRNA, BRD4S shRNA vectors, 10% pCMV-VSVG, 30% pMDLg/pRRE, and 10% pRSV rev), and polyethyleneimine polymer (Polysciences Inc; 23966-1), which was pre-incubated for 15 min at room temperature. Transfection medium was replaced with fresh medium after 24 h (day 1). On days 2 and 3, the cell-conditioned medium was filtered with a 0.45 µm filter unit (Merck Millipore; 051338) and stored at 4°C. Viral particles were concentrated using a Lenti-X Concentrator (Clonetech; 631232) following the manufacturer's instructions, and virus aliquots were stored at −80°C until use.

MDA-MB-231 and BT-549 cells were infected by adding concentrated viral particles to their culture media. After 18 h, the medium was replaced with fresh medium containing 2 µg/ml of puromycin (Sigma-Aldrich; P8833). At 48 h after puromycin selection, cells were used for the experiments.

RNA extraction and qPCR

RNA was extracted using the PureLink RNA mini kit (Invitrogen) and converted into cDNA using the High-Capacity RNA-to-DNA kit (Applied Biosystems), following the manufacturer's instructions. qPCR was performed using the Power SYBR Green PCR Master Mix (Applied Biosystems) in a 7900HT thermocycler (Applied Biosystems). The primers used for amplification are listed in Table 2.

© 2023 The Authors

Table 2. List of primers used in the study.

Primer	Direction	Sequence	Application
PUM1	Forward	5′-TTCCTTCAGACCAGCAGGTAAT-3′	qPCR
PUM1	Reverse	5′-GGATAAGGCAAATACCTGTCCC-3′	qPCR
HMGB2	Forward	5′-CTTGGCACGATATGCAGCAA-3′	qPCR
HMGB2	Reverse	5′-CAGCCAAAGATAAACAACCATATGA-3′	qPCR
EZH2	Forward	5′-GACCTCTGTCTTACTTGTGGAGC-3′	qPCR
EZH2	Reverse	5′-CGTCAGATGGTGCCAGCAATAG-3′	qPCR
AURKB	Forward	5′-CAGAGAGATCGAAATCCAGGC-3′	qPCR
AURKB	Reverse	5′-CCTTGAGCCCTAAGAGCAGAT-3′	qPCR
PLK4	Forward	5′-GACTGCGTGAAGGAAGCTAATC-3′	qPCR
PLK4	Reverse	5′-TCTCTGTACCATTCCTGCTTTG-3′	qPCR
prCDH1	Forward	5′-AACCCTCAGCCAATCAGCGG-3′	ChIP-qPCR
prCDH1	Reverse	5′-GTTCCGACGCCACTGAGAGG-3′	ChIP-qPCR
prPol2	Forward	5′-CTGAGTCCGGATGAACTGGT-3′	ChIP-qPCR
prPol2	Reverse	5′-ACCCATAAGCAGCGAGAAAG-3′	ChIP-qPCR
prBRD4	Forward	5′-TTTCCTGGCCTCCTGACTGC-3′	ChIP-qPCR
prBRD4	Reverse	5′-GACCCTGCAACTTGCCTTGG-3′	ChIP-qPCR
prEZH2	Forward	5′-CTGGTTCAAACTTGGCTTCCA-3′	ChIP-qPCR
prEZH2	Reverse	5′-TTCTTTCGCTGAACACACGG-3′	ChIP-qPCR
prHMGB2	Forward	5′-CTGTAGTCGCTCTGCTCTGT-3′	ChIP-qPCR
prHMGB2	Reverse	5′-CCTTGACTTCCCCGAGTTCT-3′	ChIP-qPCR
prAURKB	Forward	5′-ACCTGATCATCTGCCCACTC-3′	ChIP-qPCR
prAURKB	Reverse	5′-CATTCCGCCTCTTCCATTGG-3′	ChIP-qPCR
prPLK4	Forward	5′-TTAGAGAGCCGAGCCTGATG-3′	ChIP-qPCR
prPLK4	Reverse	5′-TCCCACAATTACTCCCACCC-3′	ChIP-qPCR
Ad1_noMX	Forward	5′-AATGATACGGCGACCACCGAGATCTACA<br>CTCGTCGGCAGCGTCAGATGTG-3′	ATAC-seq
Ad2.1_TAAGGCGA	Reverse	5′-CAAGCAGAAGACGGCATACGAGAT<br>TCGCCTTAGTCTCGTGGGCTCGGAGATGT-3′	ATAC-seq
Ad2.2_CGTACTAG	Reverse	5′-CAAGCAGAAGACGGCATACGAGAT<br>CTAGTACGGTCTCGTGGGCTCGGAGATGT-3′	ATAC-seq
Ad2.3_AGGCAGAA	Reverse	5′-CAAGCAGAAGACGGCATACGAGAT<br>TTCTGCCTGTCTCGTGGGCTCGGAGATGT-3′	ATAC-seq
Ad2.4_TCCTGAGC	Reverse	5′-CAAGCAGAAGACGGCATACGAGAT<br>GCTCAGGAGTCTCGTGGGCTCGGAGATGT-3′	ATAC-seq
Ad2.5_GGACTCCT	Reverse	5′-CAAGCAGAAGACGGCATACGAGAT<br>AGGAGTCCGTCTCGTGGGCTCGGAGATGT-3′	ATAC-seq
Ad2.6_TAGGCATG	Reverse	5′-CAAGCAGAAGACGGCATACGAGAT<br>CATGCCTAGTCTCGTGGGCTCGGAGATGT-3′	ATAC-seq
pcDNA3	Forward	5′-TACGACTCACTATAGGGAGACCCAAG-3′	Sanger
shBRD4 Short (TRCN0000349782)	Forward	5′-CCGGATTGGACACGGACTCTTAATACTCGAGTATTAAGAGTCCGTGTCCAATTTTTTG-3′	Cloning
shBRD4 Short (TRCN0000349782)	Reverse	5′-AATTCAAAAAATTGGACACGGACTCTTAATACTCGAGTATTAAGAGTCCGTGTCCAAT-3′	Cloning
shBRD4 Long (TRCN0000021427)	Forward	5′-CCGGCCTGGAGATGACATAGTCTTACTCGAGTAAGACTATGTCATCTCCAGGTTTTTG-3′	Cloning
shBRD4 Long (TRCN0000021427)	Reverse	5′-AATTCAAAAACCTGGAGATGACATAGTCTTACTCGAGTAAGACTATGTCATCTCCAGG-3′	Cloning

© 2023 The Authors

Protein–protein docking analysis

Two structures covering BD1 and BD2 of BRD4 were retrieved from the Protein Data Bank (PDB) (Berman *et al*, 2000), and 45 models related to six observed protein–protein interactions involving the BRD4 bromodomains were extracted from Interactome3d (Mosca *et al*, 2013) (Table EV1). The first group of structures, together with the only available PDB model for LOXL2 (5ZE3), was used to run docking on the ZDOCK server (Pierce *et al*, 2014) and Autodock VINA (Trott & Olson, 2010). Models capturing BRD4 in interactions were used as input for ProteinFishing (Cianferoni *et al*, 2020) together with the LOXL2 structure. The obtained models were later minimized using the YASARA structure minimization routine, followed by the FoldX *RepairPDB*. A reliability ranking based on FoldX (Delgado *et al*, 2019) interaction energy, stability, and buried surface was generated (Table EV1) using the FoldX *AnalyseComplex* and *Stability* commands, and the buried surface was computed using the YASARA structure. The top 10 models for each BD were selected using the ranking mentioned above, and models incompatible with the LOXL2 AlphaFold (Jumper *et al*, 2021) model AF-Q9Y4K0-F1 were excluded. Such a model predicts the formation and packing of a domain implying residues 1–318, unsolved in the LOXL2 PDB structure. Models with similar poses were grouped into clusters and further pruned by considering the top-ranking model as representative of each cluster. For each of the six final models, the residues involved in the interaction were determined using the FoldX *AnalyseComplex* command. The FoldX *Pssm* command was used to predict the ΔΔG for mutating each of the interaction residues to alanine. Mutations predicting a ΔΔG of interaction greater than 2.0 kcal/mol characterize the relative residue position as fundamental for the interaction. In the same way, ΔΔGs smaller than −2.0 kcal/mol should be considered invalidating for the relative model (Table EV2). Nevertheless, model *4uyd_zdock_3* was spared, given the fact that the unsatisfactory residue D144 possibly forms a salt bridge with R447 of LOXL2. In contrast, ProteinFishing model *O60885-O60885-EXP-5khm_5ze3_1* was excluded because of its lack of strong interactions to support the proposed binding pose (Table EV2). Asparagines 140 and 433 for BD1 and BD2 were mutated to phenylalanine and, in accordance with the experimental data, models implying a loss of binding upon mutation were excluded (Table EV3).

Cell cycle analysis

A total of 1×10^6 cells MDA-MB-231 cells were trypsinized and washed with PBS. Cells were fixed using 1 ml of 70% cold ethanol (diluted with PBS) and added dropwise to the cell pellet while vortexing (in order to avoid cell clumping). After at least 30 min on ice, fixed cells were carefully washed three times with PBS. The washed pellet was resuspended in 1 ml of Propidium Iodide (PI) staining buffer (100 μg/ml RNAse A and 50 μg/ml PI (Sigma-Aldrich; #P4864) in PBS) and incubated for at least 30 min at 4°C in the dark. The stained cells were analyzed by FACS using LSRFortessa and FlowJo V10 (BD Biosciences).

Small molecule treatment and synergism analysis

Triple-negative breast cancer cell lines were seeded on day 1 in 96-well plates in triplicate: MDA-MB-468 (4,000 cells/well), MDA-MB-231 (3,000 cells/well), and BT-549 (3,000 cells/well). On the second day (day 2), cells were treated with the indicated concentrations of JQ1 or PXS and their combinations. After 96 h (day 6), the MTT (3-(4,5-dimethylthiazol-2-yl)-2,5-diphenyltetrazolium bromide) assay was performed by adding 0.5 mg of MTT (Cat A2231, Panreac AppliChem) per ml of Dulbecco's Modified Eagle Medium (DMEM) without fetal bovine serum (FBS) for 3 h at 37°C to assess cell viability. The synergy score was calculated using the Synergy Finder 2.0 software (Ianevski *et al*, 2020).

High-throughput immunofluorescence

High-throughput immunofluorescence analysis was performed using cells seeded on clear flat-bottom 96-well plates (Perkin Elmer), treated as described in the manuscript, and fixed with 4% paraformaldehyde for 10 min. Permeabilization and blocking were performed using PBS/3% bovine serum albumin (BSA)/0.1% Triton for 30 min. The cells were incubated first with primary antibodies for 1 h at room temperature (H3S10P #06-570; 1:2,000, BRD4 #ab128874; 1:500, Med1 #LS-C290523; 1:200) and then with secondary antibodies (Alexa Fluor 488 goat anti-rabbit; 1:1,000 and Alexa Fluor 555 donkey anti-mouse; 1:1,000, Thermo Fisher Scientific) for 30 min at room temperature in the dark. Finally, cells were washed and incubated with DAPI (4,6-diamidino-2-phenylindole, #MBD0015; 1:1,000) for 5 min at room temperature in the dark. The samples were washed thrice with PBS to remove excess antibodies and DAPI. Images were taken with the Operetta High Content Screening System (PerkinElmer) using a 63× or 40× objective and non-confocal mode. Images were quantified using the Harmony software, first by identifying nuclei and then quantifying their properties (of H3S10P intensity, BRD4 and MED1 foci, and BRD4/MED1 foci colocalization). For cell viability assays using DAPI count, MDA-MB-231 cells were also seeded on clear flat-bottom 96-well plates (Perkin Elmer), treated as described in the result section and fixed with 4% paraformaldehyde for 10 min. Cells were washed thrice with PBS and incubated with DAPI (4,6-diamidino-2-phenylindole, #MBD0015; 1:1,000) for 5 min at room temperature in the dark. Images were taken with the Operetta High Content Screening System (PerkinElmer) using a 10× objective and non-confocal mode. Images were quantified using the Harmony software.

Live-cell imaging

MDA-MB-231 cells expressing mTurquoise2-SLBP (18–126) and H1-Maroon1 were seeded in clear, flat-bottom 96-well plates (Perkin Elmer), treated with DMSO or PXS (40 μM), and tracked for 96 h using the Operetta High Content Screening System (PerkinElmer), 20× objective, and non-confocal mode. Images were acquired every 15 min, and mTurquoise2 and Maroon1 were quantified using the Harmony software.

Western blot and pulldown experiments

Whole-cell extracts were obtained using an SDS lysis buffer (2% SDS, 50 mM Tris–HCl, and 10% glycerol). Tumor samples were placed in a 5 ml round bottom polystyrene falcon (Corning; 352052) containing SDS lysis buffer. Samples were homogenized using

© 2023 The Authors

Dispersor ULTRA-TURRAX T10 Basic (IKA). The samples were quantified and mixed with 4× Laemmli sample buffer (Bio-Rad) and boiled at 95°C for 5 min. Proteins were separated by SDS–polyacrylamide gel electrophoresis and detected with the following antibodies: LOXL2 (Cell Signaling; #99680S; 1:1,000), BRD4 (Abcam; #ab128874; 1:1,000), Tubulin (Sigma-Aldrich; #T6557; 1:10,000), and Flag (Sigma-Aldrich; #F7425; 1:10,000), p-FAK (Cell Signaling; #3283S; 1:1,000), FAK (Cell Signaling; #3285S; 1:1,000), p-Src (Cell Signaling; #2101S; 1:1,000), Src (Cell Signaling; #2108S; 1:1,000), GAPDH (Cell Signaling; #97166S; 1:5,000).

For the histone isolation experiment, the cells were pelleted by centrifugation and washed with cold PBS. Pellets were resuspended by vortexing with lysis buffer (10 mM Tris, pH 6.5, 50 mM sodium bisulfite, 1% Triton X-100, 10 mM $MgCl_2$, 8.6% sucrose, and 10 mM sodium butyrate) and centrifuged twice at full speed for 15 s. The same procedure was repeated once with wash buffer (10 mM Tris [pH 7.4] and 13 mM EDTA). Pellets were resuspended in 0.4 N H_2SO_4 and left for 1 h at 4°C with occasional gentle mixing. After centrifugation at full speed for 5 min, the supernatants were transferred to a new tube, and acetone was added (1:9). The mixture was left overnight at –20°C and then centrifuged at full speed for 10 min. The pellets were air-dried for 5 min and resuspended in 30–100 μl of distilled water. Proteins were quantified using Bradford Assay (Bio-Rad; 5000006) and separated by SDS–polyacrylamide gel electrophoresis. The proteins were detected with anti-H3K4ox (previously generated (Cebrià-Costa *et al*, 2020); 1:1,000) or anti-H3 (Abcam; #ab1791; 1:5,000).

For the Flag pulldown experiment, MDA-MB-231 or HEK293T cells were transfected with pcDNA3-hLOXL2wt-Flag, pcDNA3-hLOXL2m-Flag, or an empty pcDNA3, and used after 48 h for pulldown experiments. For pulldown, cells were washed twice with cold PBS, lysed in high-salt lysis buffer (20 mM HEPES pH 7.4, 10% glycerol, 350 mM NaCl, 1 mM $MgCl_2$, and 0.5% Triton X-100) supplemented with protease inhibitors, and incubated for 30 min on ice for lysis. Lysate samples were centrifuged at 15,200 *g* at 4°C for 10 min. Balance buffer (20 mM HEPES [pH 7.4], 1 mM $MgCl_2$, and 10 mM KCl) was added to the resulting supernatant to reach a final NaCl concentration of 150 mM. Cell extracts were quantified using the Pierce BCA Protein Assay Kit (Thermo Scientific; PIER23225), and 1 mg was incubated with 40 μl of Flag-M2 Affinity Agarose Gel (Sigma-Aldrich; A2220) for 4 h at 4°C and washed three times with wash buffer (20 mM HEPES pH 7.4, 1 mM $MgCl_2$, 150 mM NaCl, 10% glycerol, 0.5% Triton X-100). The precipitated complexes were eluted with 2× Laemmli buffer.

For endogenous pulldown experiments, cells were washed twice with cold PBS and lysed in soft-salt lysis buffer (50 mM Tris–HCl pH 8.0, 10 mM EDTA, and 0.1% NP-40) supplemented with protease inhibitors for nuclear enrichment. After centrifugation at 800 *g* for 15 min, nuclei were pelleted and lysed in high-salt lysis buffer supplemented with protease inhibitors and then incubated for 30 min on ice for nuclei lysis. Lysate samples were centrifuged at 15,200 *g* at 4°C for 10 min. Balance buffer was added to the resulting supernatant to reach a final NaCl concentration of 150 mM. Nuclear extracts were quantified using Pierce BCA Protein Assay Kit (Thermo Scientific; #PIER23225) and 1 mg of nuclear extract was incubated overnight with a primary antibody, using: 2.5 μg anti-BRD4 (Abcam; #ab128874), 5 μg anti-MED1 (Bethyl Laboratories; A300-793A), 4 μg anti-Lin9 (Proteintech; #17882-1-AP), 4 μg anti-B-Myb (Proteintech; 18896-1-AP), or 4 μg anti-FOXM1 (Proteintech; #13147-1-AP). The samples were then incubated with Protein A Dynabeads (Thermo Scientific; #10002D) for 1 h at 4°C. The complexes were washed three times with wash buffer and eluted with 2× Laemmli buffer.

For pulldown analysis, proteins were separated by SDS–polyacrylamide gel electrophoresis and detected with the appropriate antibodies: anti-Flag (Sigma-Aldrich; #F7425; 1:10,000), anti-GFP (Abcam; #ab1218; 1:1,000), anti-BRD4 (Abcam; #ab128874; 1:1,000); Lin9 (Santa Cruz; #sc-130571; 1:500), FOXM1 (Santa Cruz; #sc-376471; 1:1,000), B-Myb (Santa Cruz; #sc-81192; 1:1,000), MED1 (Bethyl Laboratories; #A300-793A; 1:1,000), and LOXL2 (Cell Signaling; #99680S; 1:1,000).

ATAC-seq sample preparation

Three biological replicates of ATAC-seq samples were prepared as described previously (Buenrostro *et al*, 2015). Briefly, 50,000 MDA-MB-231 cells infected with shControl or shLOXL2 (KD) were collected and treated with transposase Tn5 (Nextera Tn5 Transposase; Illumina Cat #FC-121-1030). DNA was purified using AMPure XP beads to remove large fragments (0.5 × beads; > 1 kb) and small fragments (1.5 × beads; < 100 bp). Samples were then amplified using NEBNexthigh-Fidelity 2× PCR Master Mix (New England Labs Cat #M0541) with primers containing a barcode to generate the libraries, as previously described (Buenrostro *et al*, 2013). Each replicate was amplified using a combination of the forward primer and one of the reverse primers containing the adaptors (Table 2). The number of cycles of library amplification was calculated as previously described (Buenrostro *et al*, 2015). DNA was purified using a MinElute PCR Purification Kit (Qiagen), and samples were sequenced using an Illumina HiSeq 2500.

ATAC-seq analysis

Paired-end 50 bp reads were adaptor-trimmed using TrimGalore (version 0.6.5). Trimmed reads were aligned to the hg19 genome (UCSC) using BowTie 2 Aligner (version 2.4.2) (Langmead & Salzberg, 2012) with the following parameters: very-sensitive–2000. Aligned reads were filtered using SAM tools (version 1.11) (Li *et al*, 2009) to retain proper pairs with a MapQ value ≥ 30. Read pairs aligned with ChrM were discarded. Duplicate read pairs were removed using the Picard (version 2.23.8). The read alignment was offset as previously described (Buenrostro *et al*, 2013). Peaks were called using MACS2 (version 2.2.7.1) (Feng *et al*, 2012) with a false discovery rate (FDR) < 0.01. Differentially accessible regions were determined using the DiffBind package (version 3.0.7) in R (version 4.0.2) with false discovery rate (FDR) < 0.005. Genomic annotation of differential regions was performed using HOMER (version 4.11) (Heinz *et al*, 2010). Normalized read coverage values were obtained using deepTools (version 3.5.0) (Ramírez *et al*, 2016). Coverage density heatmaps were generated using deepTools with the option reference point and considering flanking regions 1 kb upstream and downstream from the center of the peaks.

ChIP sample preparation

MDA-MB-231 cells (10×10^6) infected with C or LOXL2 KD and were crosslinked in suspension using 1% formaldehyde for 10 min

 © 2023 The Authors

at 37°C. Crosslinking was stopped by adding glycine at a final concentration of 0.125 M for 5 min at room temperature. The cells were collected by centrifugation at 300 *g* for 5 min at 4°C. Cell pellets were resuspended in SDS sonication lysis buffer (1 M Tris–HCl pH 8, 10% SDS, 0.5 M EDTA) that had been supplemented with protease inhibitors. Extracts were sonicated to generate 200–600 bp DNA fragments, diluted 1:1.5 with equilibration buffer (10 mM Tris, 233 mM NaCl, 1.66% TritonX-100, 0.166% Na-deoxycholate (DOC), 1 mM EDTA) supplemented with protease inhibitors, and centrifuged at 14,000 *g* for 10 min at 4°C to pellet insoluble material. The primary antibodies used were 5 μg Ab2 (Abcam; #ab128874), 15 μg Ab1 (Bethyl Laboratories; #A301-985A100), or an irrelevant antibody (IgG). After adding the appropriate antibody to each sample, the mixtures were incubated overnight with rotation at 4°C; note that 10% of each sample was reserved as input prior to adding the antibodies. Antibody-bound chromatin was immunoprecipitated using 50 μl Protein A Dynabeads (Thermo Scientific; #10002D) for 2 h with rotation at 4°C. Precipitated samples were washed twice with RIPA-LS (10 mM Tris–HCl pH 8.0, 140 mM NaCl, 1 mM EDTA, 0.1% SDS, 0.1% DOX, 1% TritonX-100), twice with RIPA-HS (10 mM Tris–HCl pH 8.0, 0.5 M EDTA, 5 M NaCl, 10% TritonX-100, 10% SDS, 10% DOX), and twice with RIPA-LiCl (10 mM Tris–HCl pH 8.0, 1 mM EDTA, 250 mM LiCl, 0.5% NP-40, 0.5% DOX). Beads were resuspended in 48 μl ChIP elution buffer (10 mM Tris–HCl pH 8.0, 5 mM EDTA, 300 mM NaCl, 0.4% SDS) and 2 μl proteinase K, and incubated at 55°C for 1 h and then at 65°C overnight for de-crosslinking (inputs were also incubated in parallel during the de-crosslinking step).

DNA was purified using a MinElute PCR Purification Kit (Qiagen) and eluted in nuclease-free water. The NEBNext Ultra DNA Library Prep Kit for Illumina was used to prepare the libraries, and the samples were sequenced using the Illumina HiSeq 2500 system.

For ChIP-qPCR experiments, MDA-MB-468 cells were transfected with Empty-pcDNA3, pcDNA3-hLOXL2wt-Flag or pcDNA3-hLOXL2m-Flag (Appendix Fig S2A). For Appendix Fig S2D and G, MDA-MB-231 and BT-449 cells were infected with shContrl (C) and shLOXL2 (LOXL2 KD), respectively. MDA-MB-231 cells were also infected with shColol (C), shBRD4 Long (BRD4L KD) or shBRD4 Short (BRD4S KD) (Fig 3D). The experiments were performed as previously described using 5 μg of either H3K4ox (Cebrià-Costa *et al*, 2020) or Ab2 (#ab128874). Genomic regions were detected by qPCR using the Power SYBR Green PCR Master Mix (Applied Biosystems) in a 7900HT thermocycler (Applied Biosystems). The primers used for amplification are listed in Table 2. Results were analyzed relative to the input and to the amount of irrelevant IgG immunoprecipitated in each condition.

ChIP-seq analysis

Single-end 50 bp reads were adaptor-trimmed using TrimGalore (version 0.6.5). Trimmed reads were aligned to the hg19 genome (UCSC) using BowTie 2 Aligner (version 2.4.2) (Langmead & Salzberg, 2012). The aligned reads were filtered using SAMtools (version 1.11) (Li *et al*, 2009) to retain reads with a MapQ value ≥ 30. Duplicate read pairs were removed using the Picard (version 2.23.8). Peaks were called using MACS2 (version 2.2.7.1) (Feng *et al*, 2012) with a false discovery rate (FDR) < 0.01 (Dataset EV2). Genomic annotation of differential peaks was performed using HOMER (version 4.11) (Heinz *et al*, 2010). Normalized read coverage values were obtained using deepTools (version 3.5.0) (Ramírez *et al*, 2016). Motif enrichment analysis was performed using HOMER (version 4.11) (Heinz *et al*, 2010). Gene ontology was performed using the clusterProfiler package (version 3.18.0) (Yu *et al*, 2012) in R (version 4.0.2). Gene overlaps with the MSigDB collections (version 7.4) were performed using http://www.gsea-msigdb.org/gsea/msigdb/annotate.jsp. Overlaps were calculated with C2 (curated gene sets), C5 (ontology gene sets), and C6 (oncogenic signature gene sets). The statistical significance of the distributions of ATAC-seq signals in ChIP-seq peaks was determined using a two-sample Kolmogorov–Smirnov test.

RNA-seq sample preparation

Three biological replicates of RNA samples were prepared from 1×10^6 MDA-MB-231 cells infected with shControl RNA (C) or shLOXL2 RNA (KD). RNA was extracted using a PureLink RNA mini kit (Invitrogen) and sequenced on an Illumina HiSeq 2500 system.

RNA-seq analysis

Single-end, 50-bp-long reads were aligned to the GRCh37.p13 Homo Sapiens reference genome using the STAR Aligner (version 2.7.6a) (Dobin *et al*, 2013). Gene level counts were obtained by STA–using the --quantMode GeneCounts option, using gene annotations downloaded from Gencode (Release 19 GRCh37.p13). Differential expression analysis was performed in R (version 4.0.2) using the DESeq2 package (version 1.30.0) (Love *et al*, 2014). Genes with adjusted *P*-value < 0.05 were considered differentially expressed (Dataset EV1). The lfcShrink function from DESeq2 was used for visualization purposes. Gene Set Enrichment Analysis (GSEA) was performed using the clusterProfiler package (version 3.18.0) (Yu *et al*, 2012) in R (version 4.0.2).

Mouse xenograft studies

Mouse xenograft studies were performed using 6-week-old NOD.CB17PrkdcSCID/J (NOD/SCID) female mice purchased from Janvier Labs (RRID:MGI:3760616). Animals were housed in air-filtered flow cabinets with a 12:12 light/dark cycle, and food and water were provided *ad libitum*. To generate MDA-MB-231 orthotopic xenografts, 1 million low passage cells were diluted in Matrigel/PBS (v/v 1:1) and implanted into the number four fat pad of the mouse. In the case of patient-derived xenograft, low passage PDX-549 (passage 5) or PDX-127 were expanded, and one 3-mm diameter fragment was implanted into the mouse number four fat pad. After injection of cells and implantation of PDX-549 or PDX-127 into the fat pads, mice were sutured and kept in a clean cage with drinking water supplemented with Enrofloxacin (1.2 mg/kg) for 2 weeks. Tumor xenografts were measured with calipers three times per week, and the tumor volumes were determined using the formula: (length × width^2)/2.

When the majority of tumor volumes reached 100 mm^3, mice were randomized into four groups: vehicle, JQ1 ((*S*)-JQ1; MedChemExpress, #HY-13030), PXS (PXS-5382; provided by

Dr. Wolfgang Jarlimek, Pharmaxis, Australia), and the PXS/JQ1 combo ((*S*)-JQ1 + PXS-5382). For mice administration, JQ1 was first resuspended in 10% DMSO and then supplemented with 40% PEG300, 5% Tween-80, and 45% sterilized saline buffer. In case of xenografting MDA-MD-231 cells or the PDX-549, JQ1 was administered to mice at 15 mg/kg by i.p. injection five times per week (for one cycle) over 4 weeks. In case of xenografting the PDX-127, JQ1 was administered to mice at 7.5 mg/kg by i.p. injection five times per week over 2 weeks. PXS was resuspended in PBS buffer at 20 mg/ml. The PXS solution (100 μl) was then injected into an Alzet micro/osmotic pump (model 1004) according to the manufacturer's instructions (2 mg/pump) and placed subcutaneously into the mice. Vehicle group was administered a solution of 5% DMSO, 40% PEG300, 5% Tween-80 and 45% sterilized saline buffer.

After four (MDA-MB-231/PDX-549) or 2 (PDX-127) cycles of JQ1 treatment, mice were euthanized, and tumors were excised and measured. For metastasis analyses, lungs of each mouse were dissected and fixed with 4% paraformaldehyde, embedded into paraffin, serially sectioned to 5-μm thickness, and stained with hematoxylin–eosin (H&E). Five slides from each lung section (specifically, the 3rd, 5th, 15th, 25th, and 35th) were used to count metastatic nodules.

Immunohistochemistry and Masson's trichrome staining

For immunohistochemistry analyses, the third part of tumors from each mouse was dissected and fixed with 4% paraformaldehyde, embedded into paraffin, serially sectioned to 5-μm thickness, and stained with hematoxylin–eosin (H&E), H3S10p (Cell Signaling; #9701; 1:100), Ki67 (Roche Diagnostics; #05278384001), and cleaved caspase 3 (Cell Signaling; #9661; 1:100) antibodies. Briefly, slides were heated at 75°C for 8 min and deparaffinized with EZ prep solution (Ventana Medical System; 950-102 2 l). Antigen retrieval was performed at 95°C for 64 min using the Cell Conditioning Buffer 1 (Ventana Medical System; 950-124 2 l. For peroxidase blockade, samples were incubated or 8 min with CM inhibitor (ChromoMap DAB kit). The secondary antibody used was UltraMap anti-Rabbit antibody (Roche Diagnostics; 05269717001). As a detection system, CM ChromoMap DAB kit (Roche Diagnostics; 760-159) was used according to the manufacturer's instructions, followed by counterstaining with hematoxylin II (Ventana Medical System; 760-2021) for 8–12 min and bluing reagent (Ventana Medical System; 760-2037) for 4 min, dehydration, and mounting processes. Slides were scanned in the NanoZoomer 2.0-HT slide scanner and visualized in the NDP.view2 software (Hamamatsu Photonics).

Masson's trichrome staining (Bioquochem; KH07007) was performed following the manufacturer's instructions using paraffin-embedded tumors prepared as explained before. Briefly, slides were incubated O/N at 60°C. Samples were deparaffinized and hydrated with ddH2O. After staining using the manufacturer's buffers, samples were dehydrated and mounted. Slides were scanned in the NanoZoomer 2.0-HT slide scanner and quantified using the Qupath software.

Super-enhancers detection

Super-enhancers were obtained with the ROSE package (Lovén *et al*, 2013; Whyte *et al*, 2013), using the default settings. H3K27ac peaks obtained from GSE49651 were used as constituent enhancers, and a total of 180 Super-Enhancers were determined. Overlaps between Ab1 peaks and Ab2 peaks with Super-Enhancers within 1 Mb of DREAM target genes were calculated using bedtools (version 2.29.2).

The paper explained

Problem
Among the different subtypes of breast cancer, triple-negative breast cancer (TNBC) accounts for 15% of all diagnosed cases. Due to the lack of effective treatment options and to the highly heterogeneous nature of this cancer subtype, patients with TNBC have a poor prognosis and are at a higher risk of developing drug resistance. Therefore, there is an urgent need to develop new therapeutic approaches to address this unmet medical need.

Results
We found that LOXL2 expression can predict the response to BET inhibitors (BETi) in cancer cells. In TNBC, LOXL2 interacts with the short isoform of BRD4 (BRD4S), the functional partner MED1, and the transcriptional regulator B-Myb in the nucleus. These interactions play a crucial role in the formation of BRD4-MED1 transcriptional foci, which regulate the transcription of cell cycle genes, and ultimately, TNBC proliferation. When LOXL2 and BRD4 are inhibited simultaneously, LOXL2-BRD4S-MED1 interactions are dismantled, leading to the suppression of TNBC progression both in vitro and in vivo through synergistic cooperation.

Impact
Inhibiting both BRD4 and LOXL2 shows promise as a novel therapeutic approach for the treatment of TNBC.

Ethics

The authors declare that animal use was in accordance with the institutional guidelines of the Vall d'Hebron Institute of Oncology (VHIO), where all *in vivo* experiments were performed.

Statistics

The statistical tests used are described in each figure legend. No data were excluded. Randomization was not required because of the size of the datasets used. The sample size (specified in each figure legend) varies depending on the experiment and was decided based on the expected variability. High-throughput immunofluorescence was performed in blinded mode using an automated screening microscope.

Data availability

Sequencing samples (raw data and processed files) are available at NCBI's Gene Expression Omnibus under the accession number GSE198647 (https://www.ncbi.nlm.nih.gov/geo/query/acc.cgi?acc=GSE198647). Differential essentiality analysis is available on GitHub (https://github.com/Skourtis/LOXL2-BRD4).

Expanded View for this article is available online.

© 2023 The Authors

Acknowledgments

We thank the CRG genomics unit, the CRG-UPF flow cytometry unit, and the VHIO mouse facility for their contribution. We thank Pharmaxis for the supply of PXS LOXL2 inhibitors. SS is supported by the *Plan Estatal* de I + D + I (COMBAT PID2019-110598GA-I00), and the ERC Starting Grant (ERC-StG-852343-EPICAMENTE). LP-R is supported by the Juan de la Cierva-*Formación* fellowship (FJC2019-040598-I) and Fundación Franscico Cobos fellowship. TVT is supported by *Plan Estatal* de I + D + I (PID2019-108008RJ-I00), AECC (INVES20036TIAN), and a Ramón y Cajal investigator contract (RYC2020-029098-I). DC is supported by the *la Caixa* Foundation PhD fellowship (ID 100010434; fellowship code LCF/BQ/DI19/11730061).

Author contributions

Laura Pascual-Reguant: Conceptualization; formal analysis; supervision; funding acquisition; validation; investigation; visualization; methodology; writing – original draft; writing – review and editing. **Queralt Serra-Camprubí:** Formal analysis; investigation; methodology. **Debayan Datta:** Data curation; software; formal analysis. **Damiano Cianferoni:** Data curation; software; formal analysis. **Savvas Kourtis:** Data curation; software; formal analysis. **Antoni Gañez-Zapater:** Investigation; methodology. **Chiara Cannatá:** Investigation; methodology. **Lorena Espinar:** Investigation; methodology. **Jessica Querol:** Investigation; methodology. **Laura García-López:** Investigation; methodology. **Sara Musa-Afaneh:** Investigation; methodology. **Maria Guirola:** Investigation; methodology. **Anestis Gkanogiannis:** Data curation; formal analysis. **Andrea Miró Canturri:** Resources. **Marta Guzman:** Resources. **Olga Rodríguez:** Resources. **Andrea Herencia-Ropero:** Formal analysis. **Joaquin Arribas:** Resources. **Violeta Serra:** Resources. **Luis Serrano:** Data curation; formal analysis; supervision. **Tian V Tian:** Formal analysis; supervision; funding acquisition; investigation; methodology. **Sandra Peiró:** Conceptualization; supervision; funding acquisition. **Sara Sdelci:** Conceptualization; supervision; funding acquisition; writing – original draft; writing – review and editing.

Disclosure and competing interests statement

TVT and SP received funding from Pharmaxis.

References

Ahn SG, Dong SM, Oshima A, Kim WH, Lee HM, Lee SA, Kwon SH, Lee JH, Lee JM, Jeong J *et al* (2013) LOXL2 expression is associated with invasiveness and negatively influences survival in breast cancer patients. *Breast Cancer Res Treat* 141: 89 – 99

Añazco C, López-Jiménez AJ, Rafi M, Vega-Montoto L, Zhang MZ, Hudson BG, Vanacore RM (2016) Lysyl oxidase-like-2 cross-links collagen IV of glomerular basement membrane. *J Biol Chem* 291: 25999 – 26012

Andrikopoulou A, Liontos M, Koutsoukos K, Dimopoulos MA, Zagouri F (2020) The emerging role of BET inhibitors in breast cancer. *Breast* 53: 152 – 163

Behera V, Stonestrom AJ, Hamagami N, Hsiung CC, Keller CA, Giardine B, Sidoli S, Yuan ZF, Bhanu NV, Werner MT *et al* (2019) Interrogating histone acetylation and BRD4 as mitotic bookmarks of transcription. *Cell Rep* 27: 400 – 415

Berman HM, Westbrook J, Feng Z, Gilliland G, Bhat TN, Weissig H, Shindyalov IN, Bourne PE (2000) The protein data bank. *Nucleic Acids Res* 28: 235 – 242

Buenrostro JD, Giresi PG, Zaba LC, Chang HY, Greenleaf WJ (2013) Transposition of native chromatin for fast and sensitive epigenomic profiling of open chromatin, DNA-binding proteins and nucleosome position. *Nat Methods* 10: 1213 – 1218

Buenrostro JD, Wu B, Chang HY, Greenleaf WJ (2015) ATAC-seq: a method for assaying chromatin accessibility genome-wide. *Curr Protoc Mol Biol* 109: 21.29.21 – 21.29.29

Cao C, Lin S, Zhi W, Lazare C, Meng Y, Wu P, Gao P, Wei J, Wu P (2020) LOXL2 expression status is correlated with molecular characterizations of cervical carcinoma and associated with poor cancer survival via epithelial-mesenchymal transition (EMT) phenotype. *Front Oncol* 10: 284

Cebrià-Costa JP, Pascual-Reguant L, Gonzalez-Perez A, Serra-Bardenys G, Querol J, Cosín M, Verde G, Cigliano RA, Sanseverino W, Segura-Bayona S *et al* (2020) LOXL2-mediated H3K4 oxidation reduces chromatin accessibility in triple-negative breast cancer cells. *Oncogene* 39: 79 – 121

Chandler C, Liu T, Buckanovich R, Coffman LG (2019) The double edge sword of fibrosis in cancer. *Transl Res* 209: 55 – 67

Chang J, Lucas MC, Leonte LE, Garcia-Montolio M, Singh LB, Findlay AD, Deodhar M, Foot JS, Jarolimek W, Timpson P *et al* (2017) Pre-clinical evaluation of small molecule LOXL2 inhibitors in breast cancer. *Oncotarget* 8: 26066 – 26078

Cianferoni D, Radusky LG, Head SA, Serrano L, Delgado J (2020) ProteinFishing: a protein complex generator within the ModelX toolsuite. *Bioinformatics* 36: 4208 – 4210

Corsello SM, Nagari RT, Spangler RD, Rossen J, Kocak M, Bryan JG, Humeidi R, Peck D, Wu X, Tang AA *et al* (2020) Discovering the anti-cancer potential of non-oncology drugs by systematic viability profiling. *Nat Cancer* 1: 235 – 248

Cuevas EP, Moreno-Bueno G, Canesin G, Santos V, Portillo F, Cano A (2014) LOXL2 catalytically inactive mutants mediate epithelial-to-mesenchymal transition. *Biol Open* 3: 129 – 137

Cuevas EP, Eraso P, Mazón MJ, Santos V, Moreno-Bueno G, Cano A, Portillo F (2017) LOXL2 drives epithelial-mesenchymal transition via activation of IRE1-XBP1 signalling pathway. *Sci Rep* 7: 44988

Delgado J, Radusky LG, Cianferoni D, Serrano L (2019) FoldX 5.0: working with RNA, small molecules and a new graphical interface. *Bioinformatics* 35: 4168 – 4169

Delmore JE, Issa GC, Lemieux ME, Rahl PB, Shi J, Jacobs HM, Kastritis E, Gilpatrick T, Paranal RM, Qi J *et al* (2011) BET bromodomain inhibition as a therapeutic strategy to target c-Myc. *Cell* 146: 904 – 917

Dempster JM, Rossen J, Kazachkova M, Pan J, Kugener G, Root DE, Tsherniak A (2019) Extracting biological insights from the project achilles genome-scale CRISPR screens in cancer cell lines. *bioRxiv* https://doi.org/10.1101/720243 [PREPRINT]

Dey A, Chitsaz F, Abbasi A, Misteli T, Ozato K (2003) The double bromodomain protein Brd4 binds to acetylated chromatin during interphase and mitosis. *Proc Natl Acad Sci U S A* 100: 8758 – 8763

Dinca SC, Greiner D, Weidenfeld K, Bond L, Barkan D, Jorcyk CL (2021) Novel mechanism for OSM-promoted extracellular matrix remodeling in breast cancer: LOXL2 upregulation and subsequent ECM alignment. *Breast Cancer Res* 23: 56

Dobin A, Davis CA, Schlesinger F, Drenkow J, Zaleski C, Jha S, Batut P, Chaisson M, Gingeras TR (2013) STAR: ultrafast universal RNA-seq aligner. *Bioinformatics* 29: 15 – 21

Drumond-Bock AL, Bieniasz M (2021) The role of distinct BRD4 isoforms and their contribution to high-grade serous ovarian carcinoma pathogenesis. *Mol Cancer* 20: 145

Eliyatkın N, Yalçın E, Zengel B, Aktaş S, Vardar E (2015) Molecular classification of breast carcinoma: from traditional, old-fashioned way to a new age, and a new way. *J Breast Health* 11: 59 – 66

Feng J, Liu T, Qin B, Zhang Y, Liu XS (2012) Identifying ChIP-seq enrichment using MACS. *Nat Protoc* 7: 1728 – 1740

© 2023 The Authors

Filippakopoulos P, Qi J, Picaud S, Shen Y, Smith WB, Fedorov O, Morse EM, Keates T, Hickman TT, Felletar I *et al* (2010) Selective inhibition of BET bromodomains. *Nature* 468: 1067 – 1073

Filippakopoulos P, Picaud S, Mangos M, Keates T, Lambert JP, Barsyte-Lovejoy D, Felletar I, Volkmer R, Müller S, Pawson T *et al* (2012) Histone recognition and large-scale structural analysis of the human bromodomain family. *Cell* 149: 214 – 231

Fischer M, Müller GA (2017) Cell cycle transcription control: DREAM/MuvB and RB-E2F complexes. *Crit Rev Biochem Mol Biol* 52: 638 – 662

Floyd SR, Pacold ME, Huang Q, Clarke SM, Lam FC, Cannell IG, Bryson BD, Rameseder J, Lee MJ, Blake EJ *et al* (2013) The bromodomain protein Brd4 insulates chromatin from DNA damage signalling. *Nature* 498: 246 – 250

Fong SF, Dietzsch E, Fong KS, Hollosi P, Asuncion L, He Q, Parker MI, Csiszar K (2007) Lysyl oxidase-like 2 expression is increased in colon and esophageal tumors and associated with less differentiated colon tumors. *Genes Chromosomes Cancer* 46: 644 – 655

Gao J, Aksoy BA, Dogrusoz U, Dresdner G, Gross B, Sumer SO, Sun Y, Jacobsen A, Sinha R, Larsson E *et al* (2013) Integrative analysis of complex cancer genomics and clinical profiles using the cBioPortal. *Sci Signal* 6: pl1

Ghandi M, Huang FW, Jané-Valbuena J, Kryukov GV, Lo CC, McDonald ER 3rd, Barretina J, Gelfand ET, Bielski CM, Li H *et al* (2019) Next-generation characterization of the cancer cell line encyclopedia. *Nature* 569: 503 – 508

Han X, Yu D, Gu R, Jia Y, Wang Q, Jaganathan A, Yang X, Yu M, Babault N, Zhao C *et al* (2020) Roles of the BRD4 short isoform in phase separation and active gene transcription. *Nat Struct Mol Biol* 27: 333 – 341

Hanahan D (2022) Hallmarks of cancer: new dimensions. *Cancer Discov* 12: 31 – 46

Heinz S, Benner C, Spann N, Bertolino E, Lin YC, Laslo P, Cheng JX, Murre C, Singh H, Glass CK (2010) Simple combinations of lineage-determining transcription factors prime cis-regulatory elements required for macrophage and B cell identities. *Mol Cell* 38: 576 – 589

Herranz N, Dave N, Millanes-Romero A, Pascual-Reguant L, Morey L, Díaz VM, Lórenz-Fonfría V, Gutierrez-Gallego R, Jerónimo C, Iturbide A *et al* (2016) Lysyl oxidase-like 2 (LOXL2) oxidizes trimethylated lysine 4 in histone H3. *FEBS J* 283: 4263 – 4273

Hornbeck PV, Zhang B, Murray B, Kornhauser JM, Latham V, Skrzypek E (2015) PhosphoSitePlus, 2014: mutations, PTMs and recalibrations. *Nucleic Acids Res* 43: D512 – D520

Huang B, Yang XD, Zhou MM, Ozato K, Chen LF (2009) Brd4 coactivates transcriptional activation of NF-kappaB via specific binding to acetylated RelA. *Mol Cell Biol* 29: 1375 – 1387

Ianevski A, Giri AK, Aittokallio T (2020) SynergyFinder 2.0: visual analytics of multi-drug combination synergies. *Nucleic Acids Res* 48: W488 – W493

Iturbide A, García de Herreros A, Peiró S (2015a) A new role for LOX and LOXL2 proteins in transcription regulation. *FEBS J* 282: 1768 – 1773

Iturbide A, Pascual-Reguant L, Fargas L, Cebrià JP, Alsina B, García de Herreros A, Peiró S (2015b) LOXL2 oxidizes methylated TAF10 and controls TFIID-dependent genes during neural progenitor differentiation. *Mol Cell* 58: 755 – 766

Jang MK, Mochizuki K, Zhou M, Jeong HS, Brady JN, Ozato K (2005) The bromodomain protein Brd4 is a positive regulatory component of P-TEFb and stimulates RNA polymerase II-dependent transcription. *Mol Cell* 19: 523 – 534

Jumper J, Evans R, Pritzel A, Green T, Figurnov M, Ronneberger O, Tunyasuvunakool K, Bates R, Žídek A, Potapenko A *et al* (2021) Highly accurate protein structure prediction with AlphaFold. *Nature* 596: 583 – 589

Jung ST, Kim MS, Seo JY, Kim HC, Kim Y (2003) Purification of enzymatically active human lysyl oxidase and lysyl oxidase-like protein from *Escherichia coli* inclusion bodies. *Protein Expr Purif* 31: 240 – 246

Krug K, Jaehnig EJ, Satpathy S, Blumenberg L, Karpova A, Anurag M, Miles G, Mertins P, Geffen Y, Tang LC *et al* (2020) Proteogenomic landscape of breast cancer tumorigenesis and targeted therapy. *Cell* 183: 1436 – 1456

Lam FC, Kong YW, Huang Q, Vu Han TL, Maffa AD, Kasper EM, Yaffe MB (2020) BRD4 prevents the accumulation of R-loops and protects against transcription-replication collision events and DNA damage. *Nat Commun* 11: 4083

Lambert JP, Picaud S, Fujisawa T, Hou H, Savitsky P, Uusküla-Reimand L, Gupta GD, Abdouni H, Lin ZY, Tucholska M *et al* (2019) Interactome rewiring following pharmacological targeting of BET bromodomains. *Mol Cell* 73: 621 – 638

Langmead B, Salzberg SL (2012) Fast gapped-read alignment with Bowtie 2. *Nat Methods* 9: 357 – 359

Latif AL, Newcombe A, Li S, Gilroy K, Robertson NA, Lei X, Stewart HJS, Cole J, Terradas MT, Rishi L *et al* (2021) BRD4-mediated repression of p53 is a target for combination therapy in AML. *Nat Commun* 12: 241

Lei S, Zheng R, Zhang S, Wang S, Chen R, Sun K, Zeng H, Zhou J, Wei W (2021) Global patterns of breast cancer incidence and mortality: a population-based cancer registry data analysis from 2000 to 2020. *Cancer Commun (Lond)* 41: 1183 – 1194

Li H, Handsaker B, Wysoker A, Fennell T, Ruan J, Homer N, Marth G, Abecasis G, Durbin R (2009) The Sequence Alignment/Map format and SAMtools. *Bioinformatics* 25: 2078 – 2079

Li KC, Girardi E, Kartnig F, Grosche S, Pemovska T, Bigenzahn JW, Goldmann U, Sedlyarov V, Bensimon A, Schick S *et al* (2021) Cell-surface SLC nucleoside transporters and purine levels modulate BRD4-dependent chromatin states. *Nat Metab* 3: 651 – 664

Liberzon A, Birger C, Thorvaldsdóttir H, Ghandi M, Mesirov JP, Tamayo P (2015) The Molecular Signatures Database (MSigDB) hallmark gene set collection. *Cell Syst* 1: 417 – 425

Love MI, Huber W, Anders S (2014) Moderated estimation of fold change and dispersion for RNA-seq data with DESeq2. *Genome Biol* 15: 550

Lovén J, Hoke HA, Lin CY, Lau A, Orlando DA, Vakoc CR, Bradner JE, Lee TI, Young RA (2013) Selective inhibition of tumor oncogenes by disruption of super-enhancers. *Cell* 153: 320 – 334

Marra A, Trapani D, Viale G, Criscitiello C, Curigliano G (2020) Practical classification of triple-negative breast cancer: intratumoral heterogeneity, mechanisms of drug resistance, and novel therapies. *NPJ Breast Cancer* 6: 54

Millanes-Romero A, Herranz N, Perrera V, Iturbide A, Loubat-Casanovas J, Gil J, Jenuwein T, García de Herreros A, Peiró S (2013) Regulation of heterochromatin transcription by Snail1/LOXL2 during epithelial-to-mesenchymal transition. *Mol Cell* 52: 746 – 757

Morinière J, Rousseaux S, Steuerwald U, Soler-López M, Curtet S, Vitte AL, Govin J, Gaucher J, Sadoul K, Hart DJ *et al* (2009) Cooperative binding of two acetylation marks on a histone tail by a single bromodomain. *Nature* 461: 664 – 668

Mosca R, Céol A, Aloy P (2013) Interactome3D: adding structural details to protein networks. *Nat Methods* 10: 47 – 53

Muhar M, Ebert A, Neumann T, Umkehrer C, Jude J, Wieshofer C, Rescheneder P, Lipp JJ, Herzog VA, Reichholf B *et al* (2018) SLAM-seq defines direct gene-regulatory functions of the BRD4-MYC axis. *Science* 360: 800 – 805

Park PG, Jo SJ, Kim MJ, Kim HJ, Lee JH, Park CK, Kim H, Lee KY, Kim H, Park JH *et al* (2017) Role of LOXL2 in the epithelial-mesenchymal transition and colorectal cancer metastasis. *Oncotarget* 8: 80325 – 80335

 © 2023 The Authors

Pickup MW, Mouw JK, Weaver VM (2014) The extracellular matrix modulates the hallmarks of cancer. *EMBO Rep* 15: 1243 – 1253

Pierce BG, Wiehe K, Hwang H, Kim BH, Vreven T, Weng Z (2014) ZDOCK server: interactive docking prediction of protein-protein complexes and symmetric multimers. *Bioinformatics* 30: 1771 – 1773

Pongas G, Kim MK, Min DJ, House CD, Jordan E, Caplen N, Chakka S, Ohiri J, Kruhlak MJ, Annunziata CM (2017) BRD4 facilitates DNA damage response and represses CBX5/Heterochromatin protein 1 (HP1). *Oncotarget* 8: 51402 – 51415

Price JE, Polyzos A, Zhang RD, Daniels LM (1990) Tumorigenicity and metastasis of human breast carcinoma cell lines in nude mice. *Cancer Res* 50: 717 – 721

Quevedo M, Meert L, Dekker MR, Dekkers DHW, Brandsma JH, van den Berg DLC, Ozgür Z, van IJcken WFJ, Demmers J, Fornerod M *et al* (2019) Mediator complex interaction partners organize the transcriptional network that defines neural stem cells. *Nat Commun* 10: 2669

Ramírez F, Ryan DP, Grüning B, Bhardwaj V, Kilpert F, Richter AS, Heyne S, Dündar F, Manke T (2016) deepTools2: a next generation web server for deep-sequencing data analysis. *Nucleic Acids Res* 44: W160 – W165

Saatci O, Kaymak A, Raza U, Ersan PG, Akbulut O, Banister CE, Sikirzhytski V, Tokat UM, Aykut G, Ansari SA *et al* (2020) Targeting lysyl oxidase (LOX) overcomes chemotherapy resistance in triple negative breast cancer. *Nat Commun* 11: 2416

Sabari BR, Dall'Agnese A, Boija A, Klein IA, Coffey EL, Shrinivas K, Abraham BJ, Hannett NM, Zamudio AV, Manteiga JC *et al* (2018) Coactivator condensation at super-enhancers links phase separation and gene control. *Science* 361: eaar3958

Sadasivam S, Duan S, DeCaprio JA (2012) The MuvB complex sequentially recruits B-Myb and FoxM1 to promote mitotic gene expression. *Genes Dev* 26: 474 – 489

Salvador F, Martin A, López-Menéndez C, Moreno-Bueno G, Santos V, Vázquez-Naharro A, Santamaria PG, Morales S, Dubus PR, Muinelo-Romay L *et al* (2017) Lysyl oxidase-like protein LOXL2 promotes lung metastasis of breast cancer. *Cancer Res* 77: 5846 – 5859

Schmelzer CEH, Heinz A, Troilo H, Lockhart-Cairns MP, Jowitt TA, Marchand MF, Bidault L, Bignon M, Hedtke T, Barret A *et al* (2019) Lysyl oxidase-like 2 (LOXL2)-mediated cross-linking of tropoelastin. *FASEB J* 33: 5468 – 5481

Sdelci S, Rendeiro AF, Rathert P, You W, Lin JG, Ringler A, Hofstätter G, Moll HP, Gürtl B, Farlik M *et al* (2019) MTHFD1 interaction with BRD4 links folate metabolism to transcriptional regulation. *Nat Genet* 51: 990 – 998

Sengupta S, George RE (2017) Super-enhancer-driven transcriptional dependencies in cancer. *Trends Cancer* 3: 269 – 281

Shi J, Wang Y, Zeng L, Wu Y, Deng J, Zhang Q, Lin Y, Li J, Kang T, Tao M *et al* (2014) Disrupting the interaction of BRD4 with diacetylated twist suppresses tumorigenesis in basal-like breast cancer. *Cancer Cell* 25: 210 – 225

Shorstova T, Foulkes WD, Witcher M (2021) Achieving clinical success with BET inhibitors as anti-cancer agents. *Br J Cancer* 124: 1478 – 1490

Shu S, Lin CY, He HH, Witwicki RM, Tabassum DP, Roberts JM, Janiszewska M, Huh SJ, Liang Y, Ryan J *et al* (2016) Response and resistance to BET bromodomain inhibitors in triple-negative breast cancer. *Nature* 529: 413 – 417

Tanaka N, Yamada S, Sonohara F, Suenaga M, Hayashi M, Takami H, Niwa Y, Hattori N, Iwata N, Kanda M *et al* (2018) Clinical implications of lysyl oxidase-like protein 2 expression in pancreatic cancer. *Sci Rep* 8: 9846

Trott O, Olson AJ (2010) AutoDock Vina: improving the speed and accuracy of docking with a new scoring function, efficient optimization, and multithreading. *J Comput Chem* 31: 455 – 461

Webber LP, Yujra VQ, Vargas PA, Martins MD, Squarize CH, Castilho RM (2019) Interference with the bromodomain epigenome readers drives p21 expression and tumor senescence. *Cancer Lett* 461: 10 – 20

Whitfield ML, Zheng LX, Baldwin A, Ohta T, Hurt MM, Marzluff WF (2000) Stem-loop binding protein, the protein that binds the 3′ end of histone mRNA, is cell cycle regulated by both translational and posttranslational mechanisms. *Mol Cell Biol* 20: 4188 – 4198

Whyte WA, Orlando DA, Hnisz D, Abraham BJ, Lin CY, Kagey MH, Rahl PB, Lee TI, Young RA (2013) Master transcription factors and mediator establish super-enhancers at key cell identity genes. *Cell* 153: 307 – 319

Wu L, Zhu Y (2015) The function and mechanisms of action of LOXL2 in cancer (Review). *Int J Mol Med* 36: 1200 – 1204

Wu SY, Lee CF, Lai HT, Yu CT, Lee JE, Zuo H, Tsai SY, Tsai MJ, Ge K, Wan Y *et al* (2020) Opposing functions of BRD4 isoforms in breast cancer. *Mol Cell* 78: 1114 – 1132

Xu Y, Vakoc CR (2017) Targeting cancer cells with BET bromodomain inhibitors. *Cold Spring Harb Perspect Med* 7: a026674

Yang Z, He N, Zhou Q (2008) Brd4 recruits P-TEFb to chromosomes at late mitosis to promote G1 gene expression and cell cycle progression. *Mol Cell Biol* 28: 967 – 976

Yu G, Wang LG, Han Y, He QY (2012) clusterProfiler: an R package for comparing biological themes among gene clusters. *OMICS* 16: 284 – 287

Zhang J, Dulak AM, Hattersley MM, Willis BS, Nikkilä J, Wang A, Lau A, Reimer C, Zinda M, Fawell SE *et al* (2018) BRD4 facilitates replication stress-induced DNA damage response. *Oncogene* 37: 3763 – 3777

Zhao S, Xu W, Jiang W, Yu W, Lin Y, Zhang T, Yao J, Zhou L, Zeng Y, Li H *et al* (2010) Regulation of cellular metabolism by protein lysine acetylation. *Science* 327: 1000 – 1004

Zuber J, Shi J, Wang E, Rappaport AR, Herrmann H, Sison EA, Magoon D, Qi J, Blatt K, Wunderlich M *et al* (2011) RNAi screen identifies Brd4 as a therapeutic target in acute myeloid leukaemia. *Nature* 478: 524 – 528

License: This is an open access article under the terms of the Creative Commons Attribution License, which permits use, distribution and reproduction in any medium, provided the original work is properly cited.

Expanded View Figures

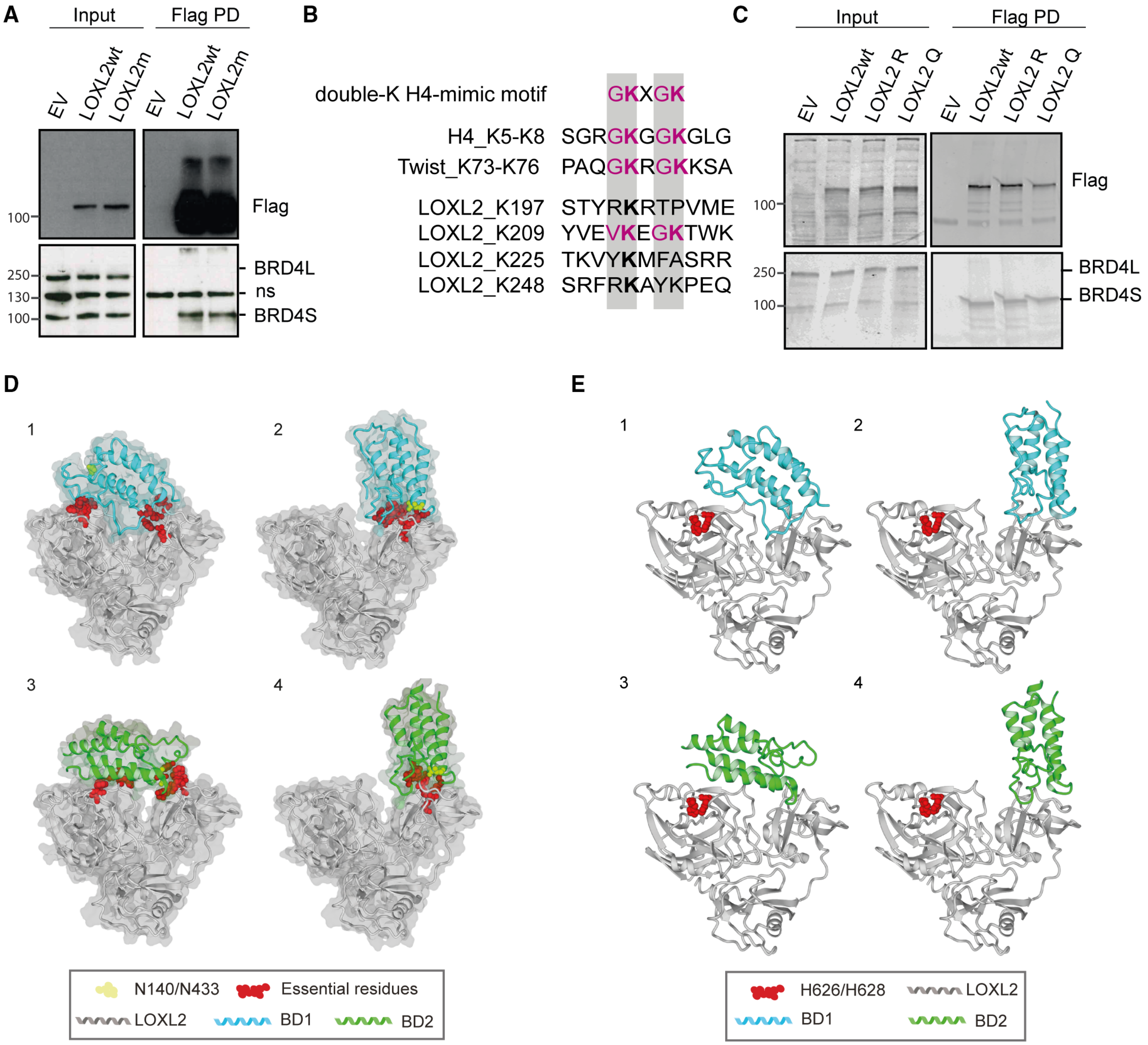

Figure EV1. LOXL2-BRD4S interaction does not involve the activity of the bromodomains.

A Flag pulldown in HEK293T cells overexpressing the empty vector (EV), LOXL2-Flag wild-type (LOXL2wt), or the catalytically dead form of LOXL2-Flag (LOXL2m). Precipitates were analyzed by Western blot with the indicated antibodies. Three biological replicates were performed. ns: non-specific.

B Schematic representation of the double-K H4-mimic motif partially shared between H4, Twist, and LOXL2.

C Flag pulldown of MDA-MB-231 cells overexpressing EV, LOXL2wt, LOXL2 R (the K209 residue mutated to R), or LOXL2 Q (the K209/K212 residues mutated to Q). Precipitates were analyzed by Western blot with the indicated antibodies. Three biological replicates were performed.

D Selected docking models, two for each of the BDs. The docking poses 1 and 2 show the BD1 structure docked on LOXL2, corresponding to models 4uyd_zdock_10 and 4uyd_zdock_3, respectively (Tables EV2 and EV3), while 3 and 4 show the BD2 structures docked into LOXL2, corresponding to models 2ouo_zdock_4 and 2ouo_zdock_5, respectively (Tables EV2 and EV3). Red indicates the atomic representation of residues predicted to be fundamental for the modeled interaction (Table EV3), while yellow indicates the asparagines (N) N140 (BD1), and N433 (BD2). The molecular volumes of the four models are shown in light gray.

E View of the four selected models highlighting LOXL2 histidines H626 and H628 whose mutations to glutamine did not affect the LOXL2 binding to BRD4. Panels 1 to 4 correspond to docking models 4uyd_complex_10, 4uyd_complex_3, 2ouo_complex_4, and 2ouo_complex_5, respectively (Table EV3).

© 2023 The Authors

Figure EV2. ChIP-seq, RNA-seq, and ATAC-seq analyses of LOXL2 downregulation in MDA-MB-231 TNBC cells.

A Representative Western blot analysis of MDA-MB-231 cells infected with C or LOXL2 KD showing LOXL2 levels. Tubulin was used as a loading control. Three biological replicates were performed.

B ATAC-seq normalized coverage in MDA-MB-231 cells transduced with either C or LOXL2 KD, represented as the distance from the center of all peaks (bp = base pair).

C Volcano Plot representation of the differential expression of genes between C and KD conditions. Significance was calculated using the Wald test with Benjamini–Hochberg correction used for multiple testing. Genes with adjusted *P*-values <0.05 and abs (FC) > 1.5 were considered significant.

D RNA-seq logFC for genes associated with ATAC-seq peaks in C and KD conditions which fall in promoter regions. The bottom and top fractions in the boxes represent the first and third quartiles, and the line, the median. Whiskers denote the interval between 1.5 times the interquartile range (IQR) and the median. Data beyond the end of the whiskers are plotted as outliers.

E Gene Set Enrichment Analysis (GSEA) of the genes upregulated upon LOXL2 KD in the RNA-seq dataset. Significance was calculated using a permutation test with Benjamini-Hochberg correction used for multiple testing.

F Number of BRD4 ChIP-seq total or promoter peaks using Ab1 or Ab2 antibodies identified with MACS2.

G Top-10 GO-terms identified either with Ab1 or Ab2 when analyzing promoter peaks with the mSigDB. Gene ratios and adjusted *P*-values are reported on the left side of each GS. GSs shared among the top 10 of Ab1 and Ab2 are indicated in bold.

H Representative Western blot analysis showing BRD4 levels in MDA-MB-231 cells infected with shControl), shBRD4 Long (BRD4L KD), and shBRD4 Short (BRD4S KD) isoforms. Tubulin was used as a loading control. Three biological replicates were performed. ns: non-specific.

I Heatmap of ChIP-seq normalized signal (reads per genomic content) in all peaks in LOXL2 KD or control cells for the antibodies Ab1 and Ab2. The normalized signal is calculated for a region of −1 to 1 kb from the center of the peaks.

J Normalized ATAC-seq signal (reads per genomic content) in the ChIP-seq peaks for Ab1 and Ab2 in LOXL2 KD or control cells. Significance was calculated using a two-sample Kolmogorov–Smirnov test. The bottom and top fractions in the boxes represent the first and third quartiles, and the line, the median. Whiskers denote the interval between 1.5 times the interquartile range (IQR) and the median. Data beyond the end of the whiskers are plotted as outliers.

K RNA-seq logFC for genes associated with the peaks of Ab1 and Ab2, which fall in promoter regions in control or LOXL2 KD cells, respectively. Significance was calculated using a two-sample Kolmogorov–Smirnov test. The bottom and top fractions in the boxes represent the first and third quartiles, and the line, the median. Whiskers denote the interval between 1.5 times the interquartile range (IQR) and the median. Data beyond the end of the whiskers are plotted as outliers.

L Venn diagram showing the number of promoter genes identified with the ChIP-seq with either Ab1 or Ab2 antibodies in control (left) or LOXL2 KD (right) cells. DREAM target genes identified in each condition are depicted in colored dots. The numbers on top of the dots represent the total number of promoters retrieved for each condition. The numbers below the dots are respectively the (upper) total number of DREAM target gene promoters retrieved in each condition and (lower) the condition-relative percentage of DREAM target gene promoters identified (DREAM target gene promoters relative to all promoters).

M Venn diagram showing the overlap between the total number of peaks detected with the ChIP-seq with either Ab1 or Ab2 in control (left) or LOXL2 KD (right) conditions. The overlap of Ab1 and Ab2 peaks is shown as a percentage (intersection relative to Ab1 peaks).

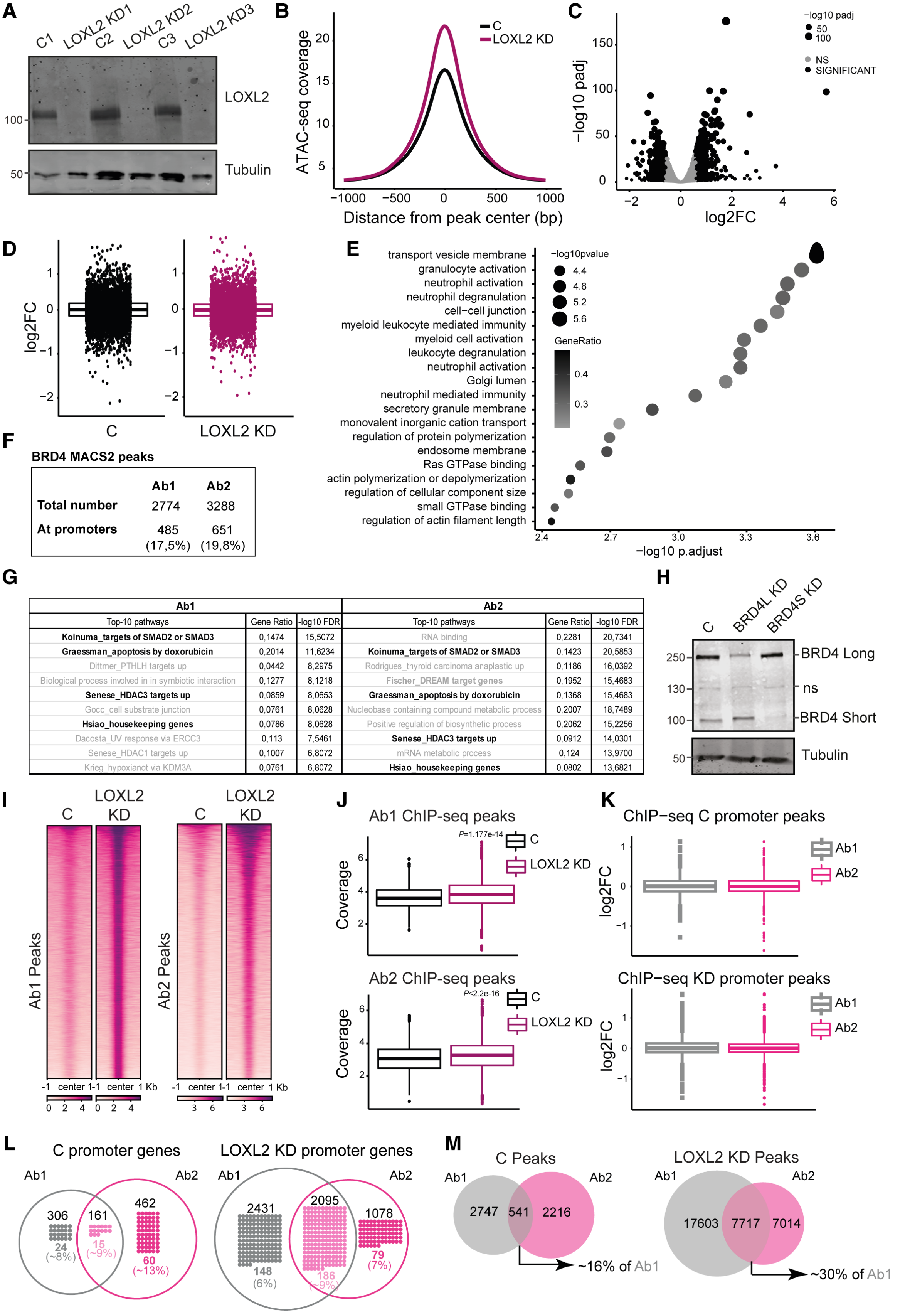

F BRD4 MACS2 peaks

	Ab1	Ab2
Total number	2774	3288
At promoters	485 (17,5%)	651 (19,8%)

G

Ab1			Ab2		
Top-10 pathways	Gene Ratio	-log10 FDR	Top-10 pathways	Gene Ratio	-log10 FDR
**Koinuma_targets of SMAD2 or SMAD3**	0,1474	15,5072	RNA binding	0,2281	20,7341
**Graessman_apoptosis by doxorubicin**	0,2014	11,6234	**Koinuma_targets of SMAD2 or SMAD3**	0,1423	20,5853
Dittmer_PTHLH targets up	0,0442	8,2975	Rodrigues_thyroid carcinoma anaplastic up	0,1186	16,0392
Biological process involved in in symbiotic interaction	0,1277	8,1218	**Fischer_DREAM target genes**	0,1952	15,4683
**Senese_HDAC3 targets up**	0,0859	8,0653	**Graessman_apoptosis by doxorubicin**	0,1368	15,4683
Gocc_cell substrate junction	0,0761	8,0628	Nucleobase containing compound metabolic process	0,2007	18,7489
**Hsiao_housekeeping genes**	0,0786	8,0628	Positive regulation of biosynthetic process	0,2062	15,2256
Dacosta_UV response via ERCC3	0,113	7,5461	**Senese_HDAC3 targets up**	0,0912	14,0301
Senese_HDAC1 targets up	0,1007	6,8072	mRNA metabolic process	0,124	13,9700
Krieg_hypoxianot via KDM3A	0,0761	6,8072	**Hsiao_housekeeping genes**	0,0802	13,6821

Figure EV2.

© 2023 The Authors

Figure EV3. LOXL2 repression affects the transcriptional regulation of DREAM target genes.

A Real-time quantitative PCR (qPCR) showing the changes in mRNA expression of four selected DREAM target genes (*EZH2*, *HMGB2*, *AURKB*, and *PLK4*) in C or LOXL2 KD MDA-MB-231 cells. Gene expression was normalized against an endogenous control (Pumilio homolog 1) and represented as the expression relative to the C condition, which was set as 1. Data are shown as the mean of three independent biological replicates. The standard deviation is shown as error bars. Significance was determined by unpaired Student's *t*-test.

B Representative images of H3S10P high-throughput immunofluorescence in LOXL2 KD or C MDA-MB-231 cells. Scale bar, 100 μm.

C Representative Western blot analysis of H3K4ox in MDA-MB-231 cells treated with DMSO or PXS for 96 h at the indicated concentrations. H3 was used as a loading control. Two biological replicates were performed.

D qPCR showing the changes in mRNA expression of four selected DREAM target genes (*EZH2*, *HMGB2*, *AURKB*, and *PLK4*) in MDA-MB-231 cells treated with DMSO or PXS. Gene expression was normalized against an endogenous control (Pumilio homolog 1) and is represented as the expression relative to the DMSO condition, which was set as 1. Data are shown as the mean of three independent biological replicates. The standard deviation is shown as error bars. Significance was determined by unpaired Student's *t*-test.

E MDA-MB-231 cells expressing SLBP-mTurquoise2 and H1-Maroon1 treated with DMSO or PXS for 96 h. Representative images of the quantification in Fig 4D are shown. Images of big panels show SLBP-mTurquoise2 (top) and H1-Maroon1 (bottom); inset panels show their overlap with brightfield. Scale bar, 100 μm.

F Gene-specific differential gene essentiality between high and low LOXL2-expressing cell lines (CCLE) as calculated by analyzing the Achilles' dataset. $N = 80$ cell lines. Significance was determined using the Student's *t*-test with BH multiple hypothesis correction. The bottom and top fractions in the boxes represent the first and third quartiles, and the line, the median. Whiskers denote the interval between 1.5 times the interquartile range (IQR) and the median. Data beyond the end of the whiskers are plotted as outliers.

© 2023 The Authors

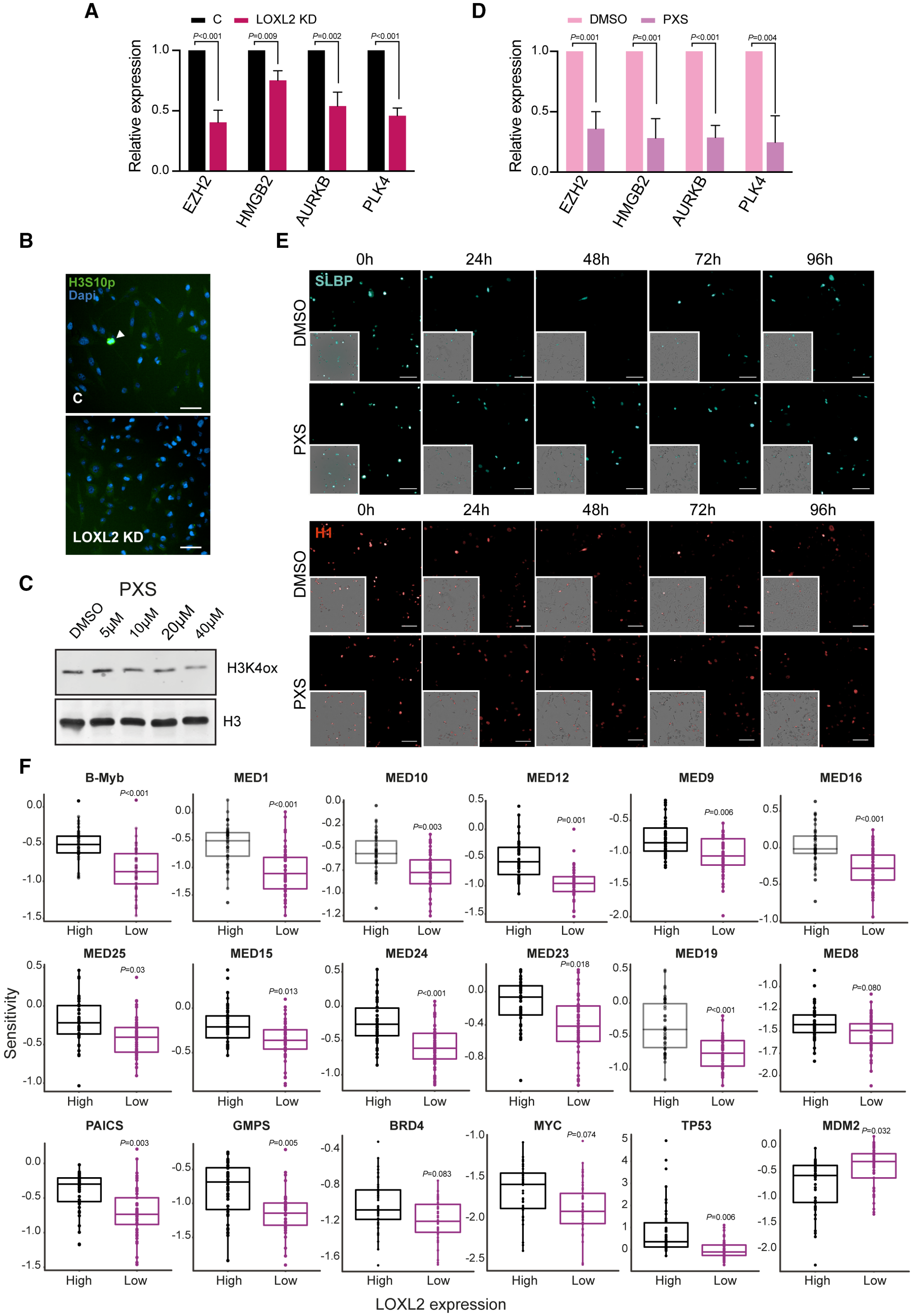

Figure EV3.

 © 2023 The Authors

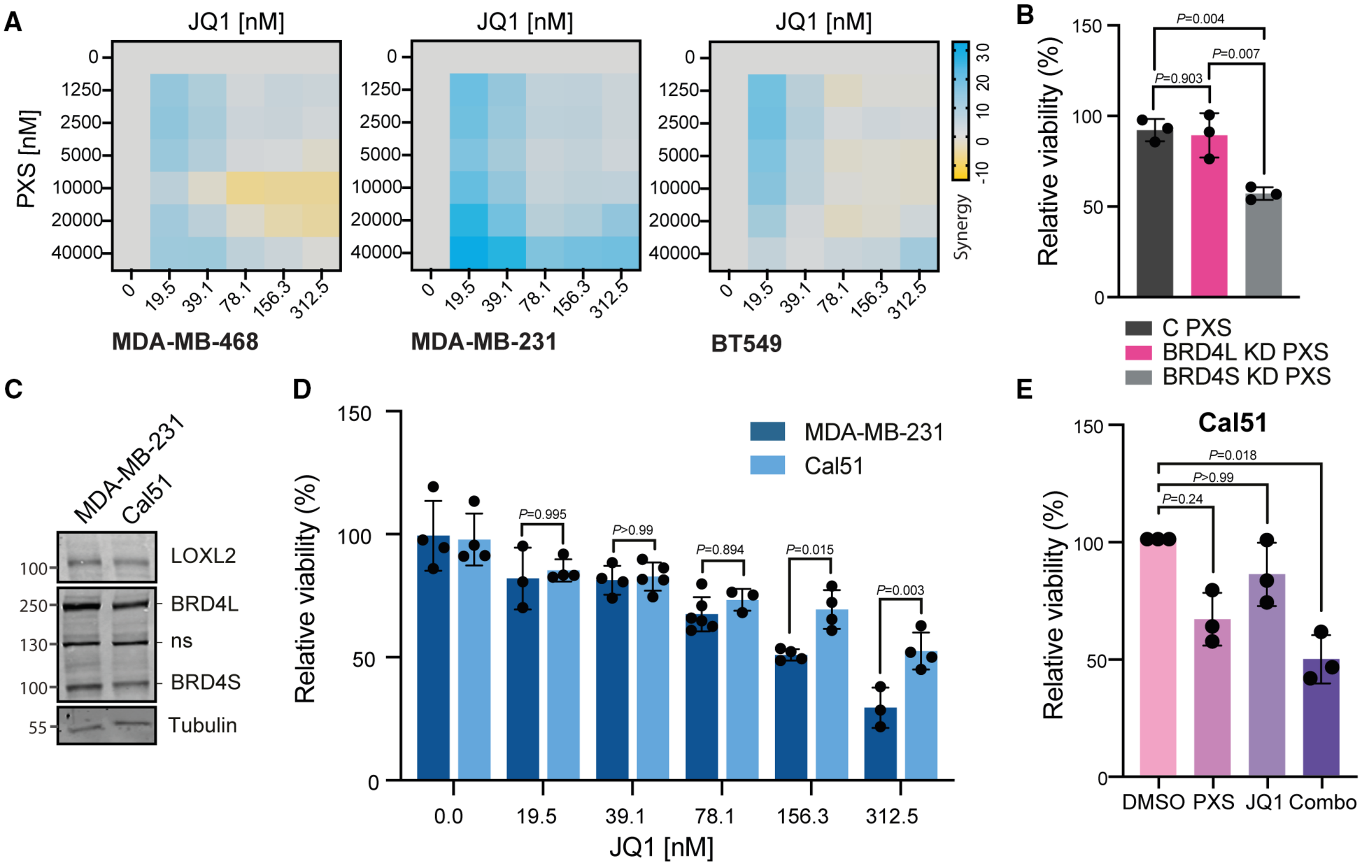

Figure EV4. Effect of LOXL2 and BRD4 inhibition on different TNBC cell lines.

A Representative matrixes showing the synergy score calculated with the cell viability data illustrated in Fig 6A.

B Cell viability assay of MDA-MB-231 cells infected with shControl (C), shBRD4L (BRD4L KD), or shBRD4S (BRD4S KD) and treated with either DMSO or 20 μM of PXS for 96 h. Data were analyzed by DAPI count using the Operetta High Content Screening System and normalized to DMSO. Data are shown as the mean of three independent biological replicates. The standard deviation is shown as error bars. Significance was determined using a one-way ANOVA multiple comparisons with Tukey's correction test.

C Representative Western blot showing BRD4 and LOXL2 levels in MDA-MB-231 and Cal51 cell lines. Tubulin is shown as a loading control. Two biological replicates were performed.

D Cell viability assay of MDA-MB-231 and Cal51 cells treated with the indicated concentrations of JQ1 for 96 h. Data were analyzed with MTT assay and normalized to DMSO. Data are shown as the mean of four independent biological replicates. The standard deviation is shown as error bars. Significance was determined using an unpaired Student's *t*-test.

E Cell viability assay of Cal51 cells treated with either DMSO, 40 μM of PXS, 312.5 nM of JQ1, or the combination of both (Combo) for 96 h. Data were analyzed with MTT assay and normalized to DMSO. Data are shown as the mean of three independent biological replicates. The standard deviation is shown as error bars. Significance was determined using one-way ANOVA multiple comparisons with Dunn's multiple comparisons test.

© 2023 The Authors

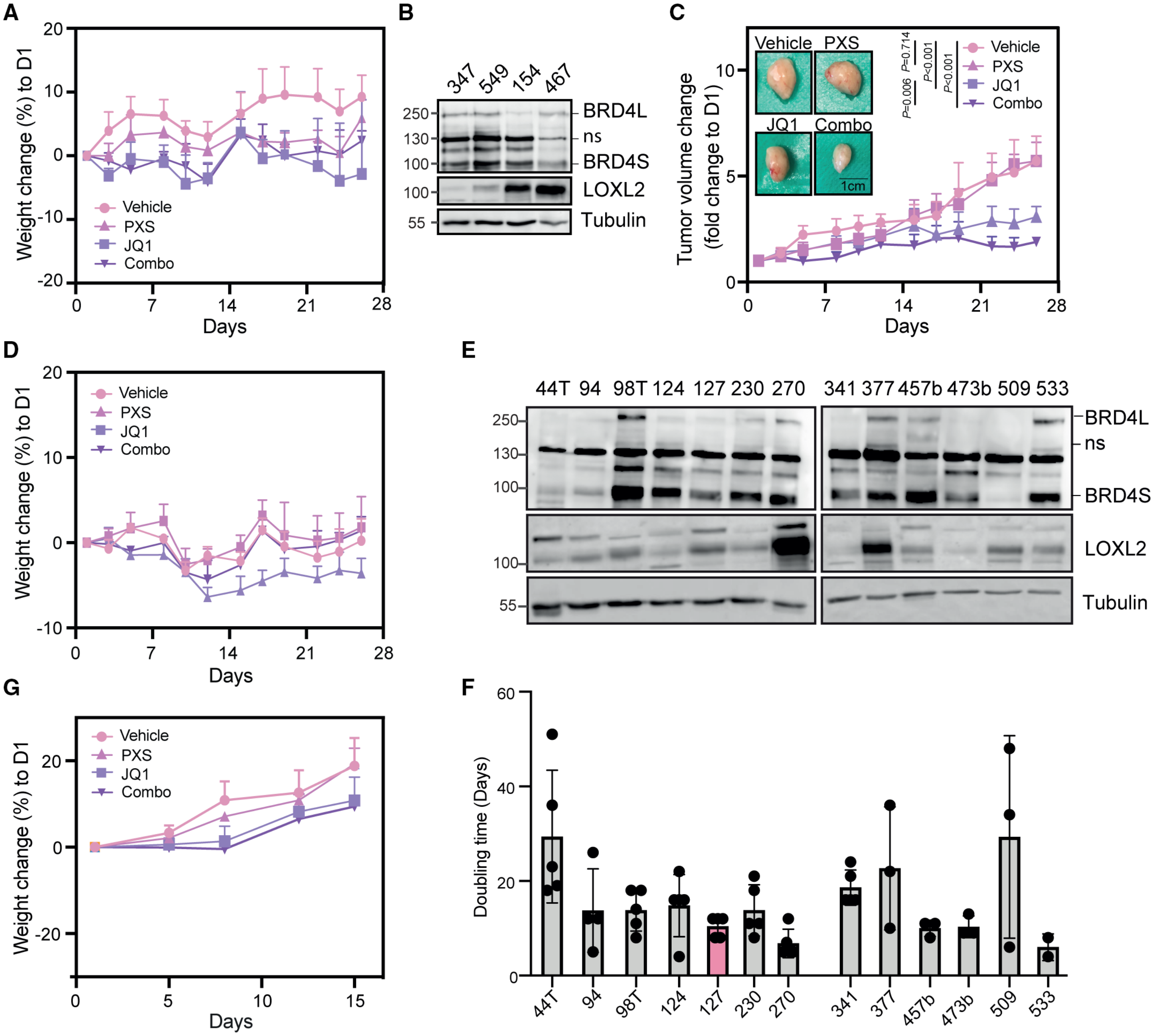

Figure EV5.

© 2023 The Authors

◀ **Figure EV5. Effect of LOXL2 and BRD4 inhibition on *in vivo* TNBC proliferation.**

A Mouse body weight changes from the MDA-MB-231 xenograft mice treated as in Fig 6B at the endpoint (day 26). Weight changes are represented as the percentage with respect to day 1, and standard deviations are shown as error bars. Body weight changes of less than 10% are considered tolerable. A minimum of six tumors per group (with three mice per group, with one tumor on each side) are shown as average mouse body weight. Statistical analysis was performed using two-way ANOVA multiple comparisons with Tukey's correction test for the whole experiment.

B Representative Western blot showing LOXL2 and BRD4 protein levels of four different PDXs. Tubulin is shown as a loading control; ns: non-specific. Two biological replicates were performed.

C Tumor volumes represented as fold change to day 1 (D1) from PDX-549 mice treated five times per week with 715 mg/kg JQ1 and/or 2 mg per pump PXS for 26 days. A minimum of six tumors per group (with 3 mice per group, with one tumor on each side) are shown as the average tumor volume. Standard deviations are shown as error bars. Significance was determined at the endpoint (day 26) using a two-way ANOVA multiple comparisons with Tukey's correction test (graph). Images of the excised tumors at the end of the experiment (day 26) (pictures).

D Mouse body weight changes from the PDX-549 xenograft mice treated as in C at the endpoint (day 26). Weight changes are represented as the percentage with respect to day 1. Standard deviations are shown as error bars. Body weight changes of less than 10% are considered tolerable. A minimum of six tumors per group (with three mice per group, with one tumor on each side) are shown as average mouse body weight. Statistical analysis was performed using two-way ANOVA multiple comparisons with Tukey's correction test.

E Representative Western blot showing LOXL2 and BRD4 protein levels of 13 different PDXs. Tubulin is shown as a loading control; ns: non-specific. Two biological replicates were performed.

F Quantification of tumor doubling time measured as the day it reaches a volume of 2 as compared to day 1. For each PDX, the different number of tumors are shown (a minimum of three different tumors were analyzed). Data are shown as the mean of the replicates. Standard deviations are shown as error bars.

G Mouse body weight changes from the PDX-127 xenograft mice treated as in Fig 6D at the endpoint (day 15). Weight changes are represented as the percentage with respect to day 1. Standard deviations are shown as error bars. Body weight changes of less than 10% are considered tolerable. A minimum of six tumors per group (with three mice per group, with one tumor on each side) are shown as average mouse body weight. Statistical analysis was performed using two-way ANOVA multiple comparisons with Tukey's correction.

© 2023 The Authors

Appendix

Table of contents

- **Appendix Figure S1: LOXL2 protein levels predict response to BETi** …… 1
- **Appendix Figure S2: Modulation of LOXL2 transcription does not induce changes of BRD4 protein levels** ………………………………………….…… 2
- **Appendix Figure S3: LOXL2 and BRD4 levels are not inversely correlated across tumors** ……………………………………………………………….. 5
- **Appendix Figure S4: BRD4 and MED1 foci intensity does not change upon treatment** …..……………………………………………………..………. 6
- **Appendix Figure S5: Characterization of MDA-MB-231 and PDX-127 excised tumors** ……………………………………………………..………...7

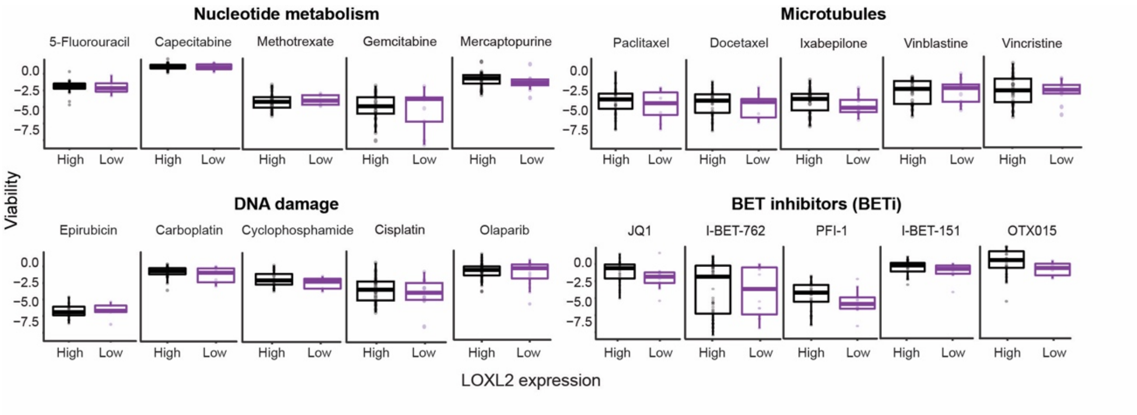

Appendix Figure S1. LOXL2 protein levels predict response to BETi.

Cell viability of high and low LOXL2-expressing CCLE cell lines (protein levels) treated with different chemotherapeutic agents and BETi small molecules at the highest concentration (i.e., 10µM). *N*=80 cell lines. The bottom and top fractions in the boxes represent the first and third quartiles, and the line, the median. Whiskers denote the interval between 1.5 times the interquartile range (IQR) and the median. Data beyond the end of the whiskers are plotted as outliers.

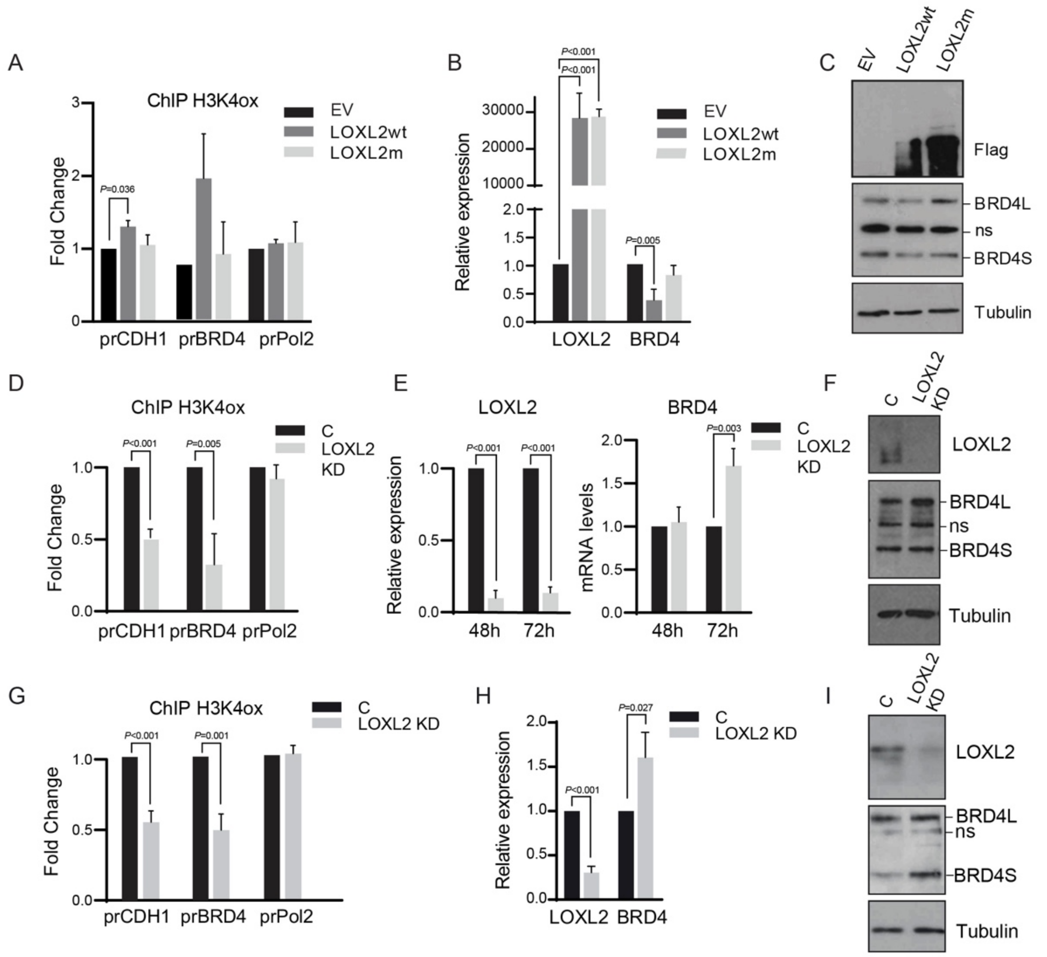

Appendix Figure S2. Modulation of LOXL2 transcription does not induce changes of BRD4 protein levels

A. H3K4ox ChIP-qPCR of the BRD4 promoter (prBRD4) in MDA-MB-468 cells overexpressing either an empty vector (EV), LOXL2-Flag wild-type (LOXL2wt), or the catalytically dead form of LOXL2-Flag (LOXL2m). E-cadherin promoter (prCDH1) and RNA Pol 2 promoter (prPol2) were used as positive and negative controls, respectively. Data from qPCR were normalized to the input and represented as the fold-change relative to the EV condition, which was set as 1. Data are shown as the mean of three independent biological replicates. Standard deviation is shown as error bars. Significance was determined using a one-way ANOVA multiple comparisons with Tukey's correction test.

B. Real-time quantitative PCR (qPCR) showing the changes in mRNA expression of LOXL2 and BRD4 in MDA-MB-468 cells overexpressing EV, LOXL2wt, or LOXL2m. Gene expression was normalized against an endogenous control (Pumilio homolog 1) and represented as the expression relative to the EV condition, which was set as 1. Data are

shown as the mean of three independent biological replicates. Standard deviation is shown as error bars. Significance was determined using a one-way ANOVA multiple comparisons with Tukey's correction test.

C. Representative Western blot of MDA-MB-468 cells overexpressing EV, LOXL2wt, or LOXL2m showing BRD4 and LOXL2 protein levels. Tubulin was used as a loading control, and Flag as a transfection control. ns, non-specific. Three biological replicates were performed.

D. H3K4ox ChIP-qPCR of the BRD4 promoter (prBRD4) in MDA-MB-231 cells infected with C or LOXL2 KD. E-cadherin promoter (prCDH1) and RNA Pol 2 promoter (prPol2) were used as positive and negative controls, respectively. Data from qPCR were normalized to the input and represented as the fold-change relative to the C condition, which was set as 1. Data are shown as the mean of three independent biological replicates. Standard deviation is shown as error bars.

E. Real-time quantitative RT-PCR (qRT-PCR) showing the changes in mRNA expression of LOXL2 and BRD4 in C or LOXL2 KD MDA-MB-231 cells. Gene expression was normalized against an endogenous control (Pumilio homolog 1) and is represented as the expression relative to the EV condition, which was set as 1. Data are shown as the mean of three independent biological replicates. Standard deviation is shown as error bars.

F. Representative Western blot of C and LOXL2 KD MDA-MB-231 cells showing BRD4 and LOXL2 protein levels. Tubulin was used as a loading control. ns, non-specific. Three biological replicates were performed.

G. H3K4ox ChIP-qPCR of the BRD4 promoter (prBRD4) in BT-549 cells infected with C or LOXL2 KD. E-cadherin promoter (prCDH1) and RNA Pol 2 promoter (prPol2) were used as positive and negative controls, respectively. Data from qPCR were normalized to the input and represented as the fold-change relative to the C condition, which was set as 1. Data are shown as the mean of three independent biological replicates. Standard deviation is shown as error bars.

H. Real-time quantitative RT-PCR (qRT-PCR) showing the changes in mRNA expression of LOXL2 and BRD4 in C and LOXL2 KD BT-549 cells. Gene expression was normalized to an endogenous control (Pumilio homolog 1) and represented as the expression relative to EV condition, which was set as 1. Data are shown as the mean of three independent biological replicates. Standard deviation is shown as error bars.

I. Representative Western blot of C and LOXL2 KD MDA-MB-231 cells showing the differences in BRD4 and LOXL2 protein levels. Tubulin was used as a loading control. ns, non-specific. Three biological replicates were performed.

For D, E, G and H, significance was determined by unpaired Student's *t*-test.

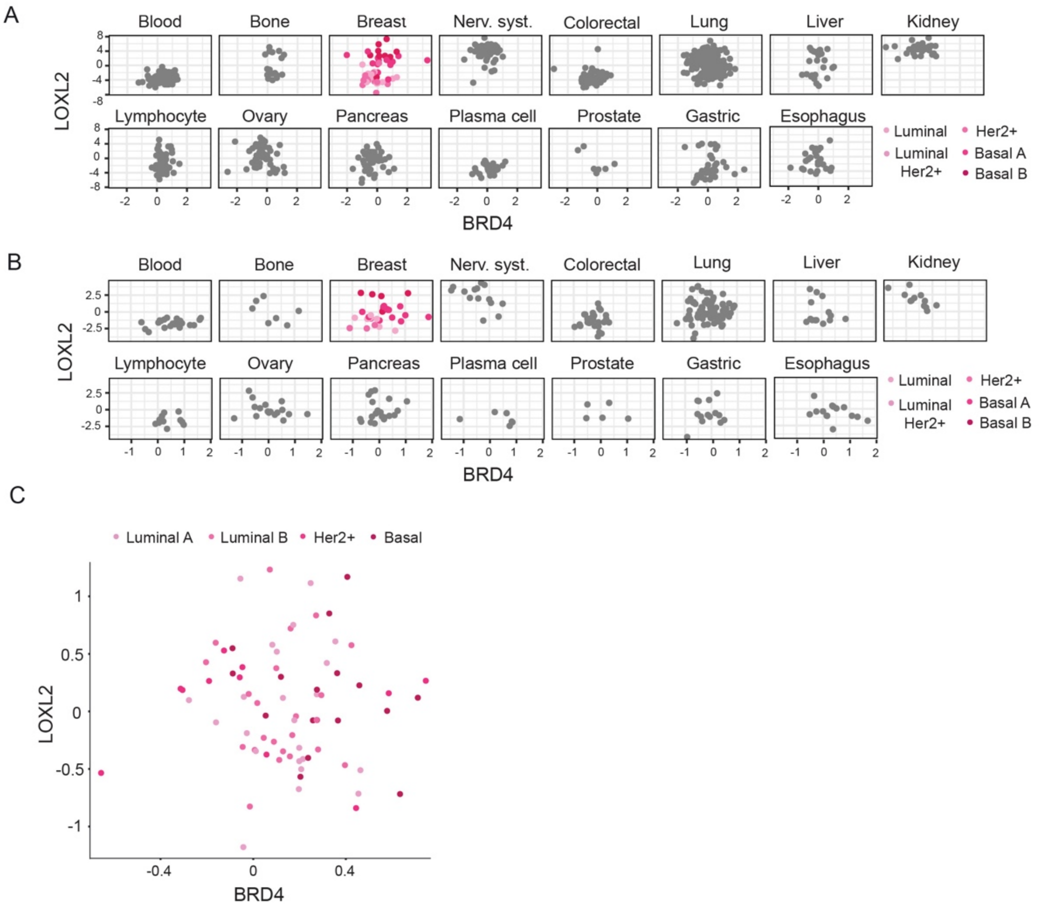

Appendix Figure S3. LOXL2 and BRD4 levels are not inversely correlated across tumors

Correlation between LOXL2 and BRD4 normalized intensities for transcript amounts (**A**) and protein levels (**B**) across CCLE cancer lineages. For breast cancer, different colors indicate different breast cancer subtypes, as indicated in the panel legend.

C. LOXL2-BRD4 protein correlation in TCGA-BRCA mass spectrometry study. Different colors indicate different breast cancer subtypes, as indicated in the panel legend.

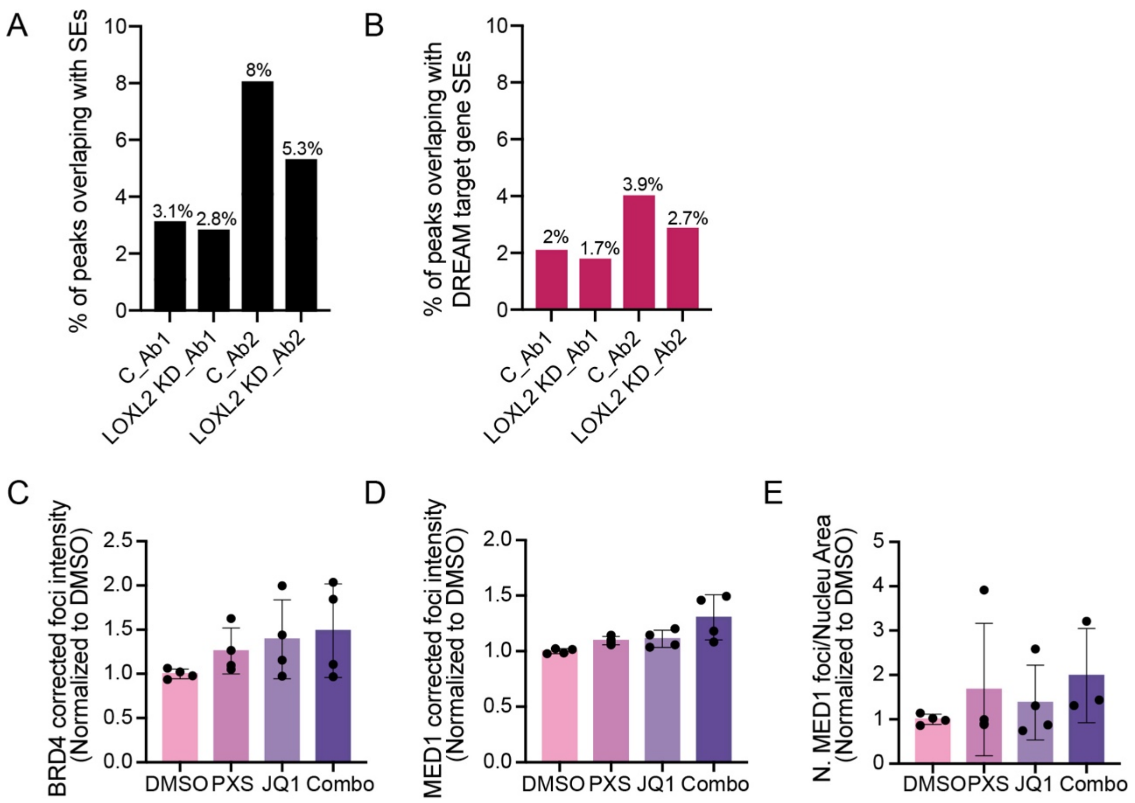

Appendix Figure S4. BRD4 and MED1 foci intensity does not change upon treatment

A. Percentage of Ab1 or Ab2 associated peaks in C or LOXL2 KD conditions overlapping with Super-Enhancers (SE).

B. Percentage of Ab1 or Ab2 associated peaks in C or LOXL2 KD conditions overlapping with Super-Enhancers (SE) located <1Mb of DREAM target genes.

C. Quantification of BRD4 foci intensity from Fig. 5g corrected by the background signal. Results are normalized to DMSO. Data are shown as the mean of four independent biological replicates. Standard deviation is shown as error bars.

D. Quantification of MED1 foci intensity from Fig. 5g corrected by the background signal. Results are normalized to DMSO. Data are shown as the mean of four independent biological replicates. Standard deviation is shown as error bars.

E. Quantification of the number of MED1 foci from Fig. 5g corrected by the nucleus area. Results are normalized to DMSO. Four biological replicates were performed using at least 4000 nuclei/replicate for the analysis. Data are shown as the mean of four independent biological replicates. Standard deviation is shown as error bars. Significance was calculated using a one-way ANOVA multiple comparisons with Tukey's correction test.

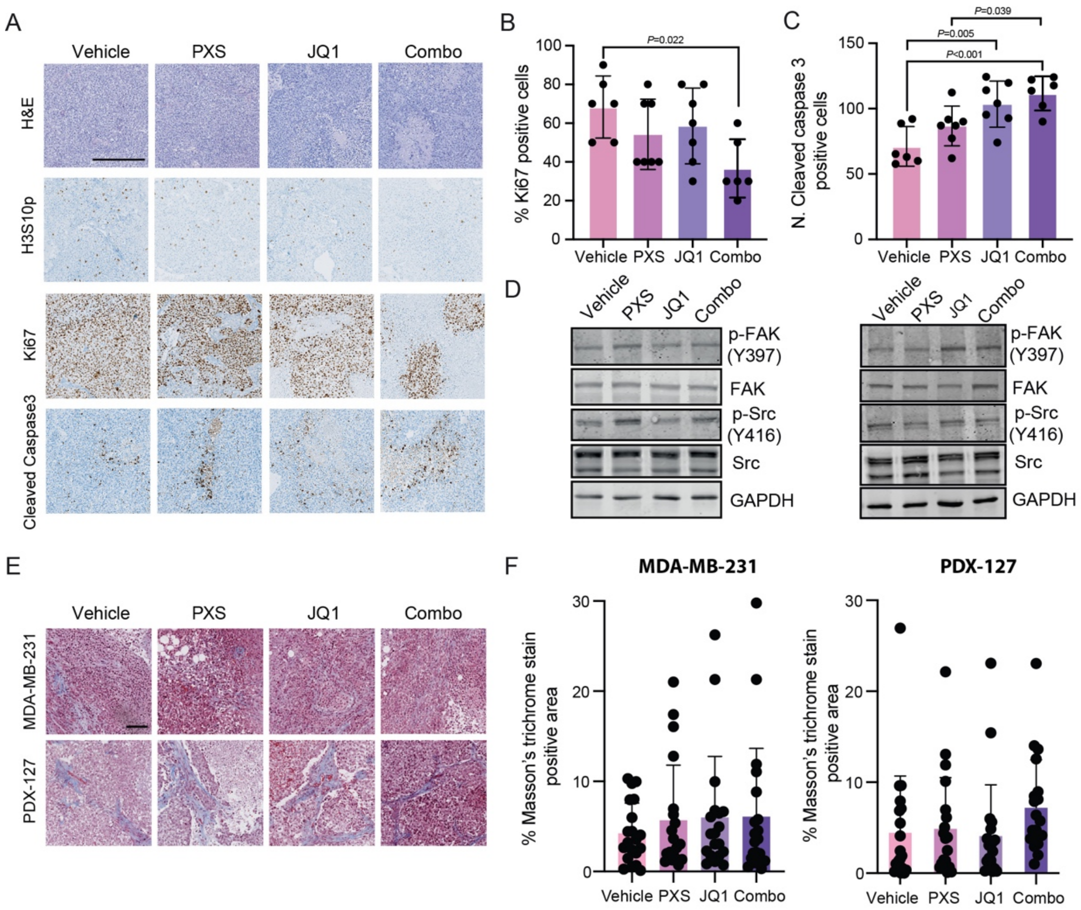

Appendix Figure S5. Characterization of MDA-MB-231 and PDX-127 excised tumors

A. Representative images of hematoxylin-eosin (H&E), H3S10p, Ki67 and cleaved caspase 3 immunohistochemistry of PDX-127 excised tumors at the endpoint (day 15). Scale bar; 100 µm.

B. Quantification of the percentage of Ki67 positive cells in each condition. Six tumors per group were analyzed. Data are shown as the mean of the six tumors. Standard deviations are shown as error bars. Significance was calculated using a one-way ANOVA multiple comparisons with Tukey's correction test.

C. Quantification of the number of cleaved caspase 3 positive cells in each condition. Six tumors per group were analyzed. Data are shown as the mean of the six tumors. Standard deviations are shown as error bars. Significance was calculated using a one-way ANOVA multiple comparisons with Tukey's correction test.

D. Western blots showing phospho FAK (p-FAK), phospho Src (p-Src), as well as FAK and Src total protein levels of PDX-127 excised tumors at the endpoint (day 15). Two

biological replicates were performed and showed (left and right). GAPDH is shown as a loading control.

E. Representative images of Masson's trichrome staining of MDA-MB-231 (top) and PDX-127 (bottom) excised tumors at the endpoint (day 26 and day 15, respectively). Scale bar; 100 μm.

F. Quantification of the percentage of Masson's trichrome stain positive area in each condition from MDA-MB-231 (left) and PDX-127 (right) excised tumors. Ten pictures per tumor and a total of two tumors per group were analyzed. Data are shown as the mean of the replicates. Standard deviations are shown as error bars.
