# Supplementary figures and images for: Interactions between BRD4S, LOXL2, and MED1 drive cell cycle transcription in triple‐negative breast cancer
Source: EMBO Mol Med. 2023 Nov 8;15(12):e18459. doi: 10.15252/emmm.202318459 (PMC10701626; doi:10.15252/emmm.202318459)

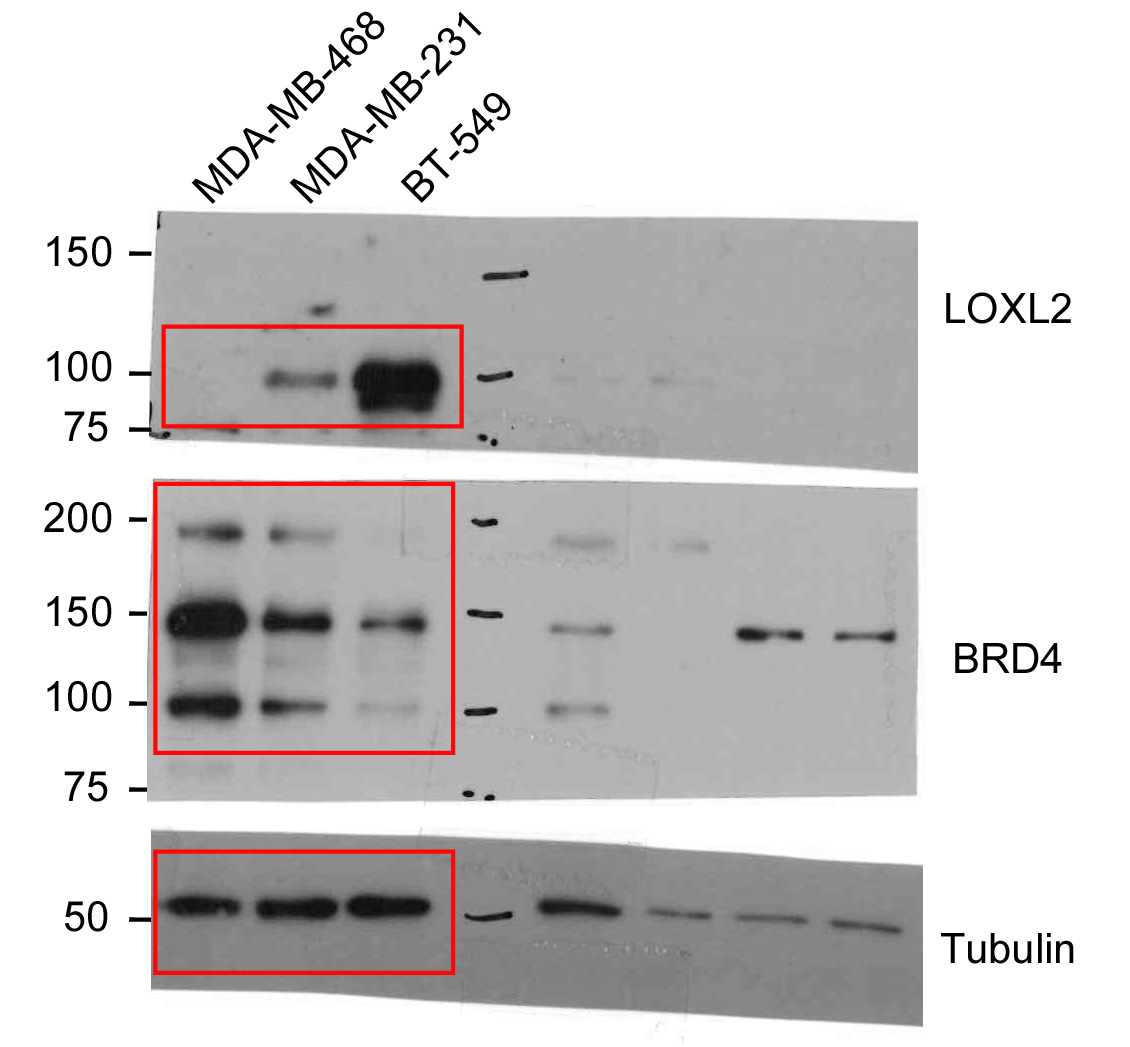

Supplement: Supplementary file 10 — Source Data for Figure 1 [file EMMM-15-e18459-s003.zip › Figure_1/1C/WB_1C.jpg]

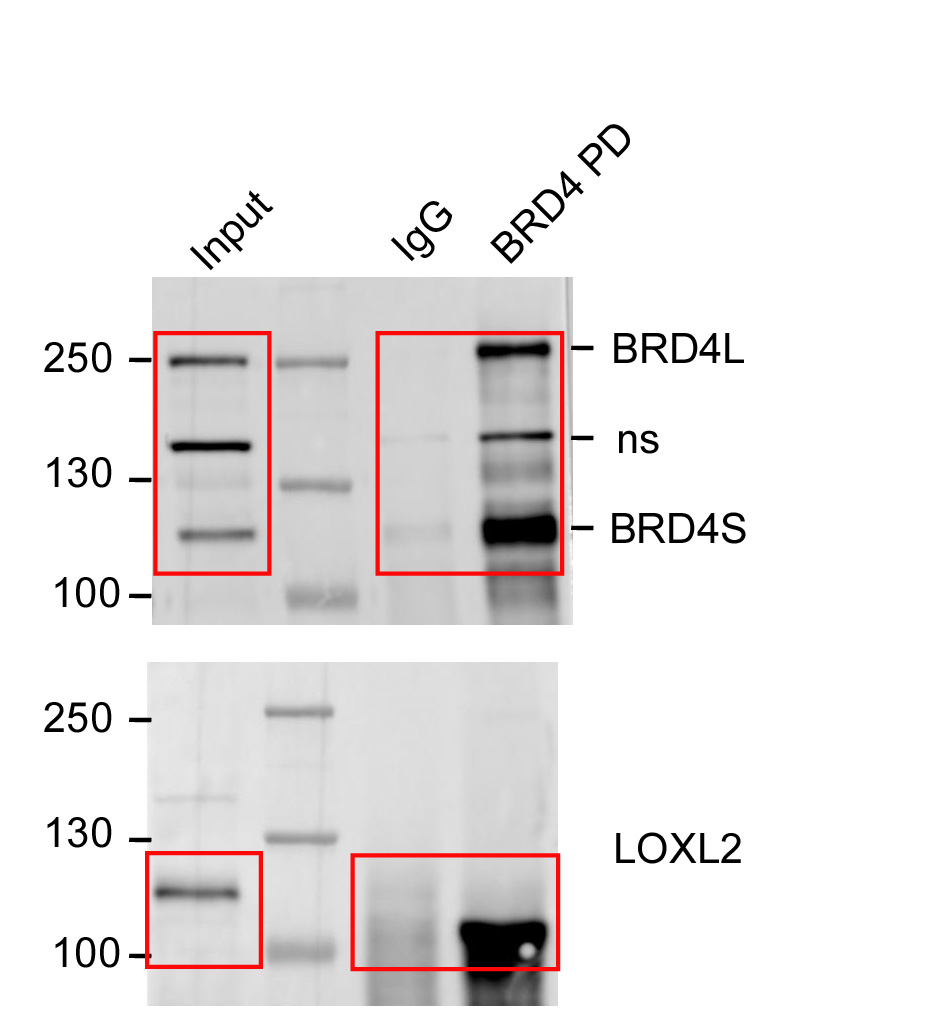

Supplement: Supplementary file 11 — Source Data for Figure 2 [file EMMM-15-e18459-s002.zip › Figure_2/2A/WB_2A.jpg]

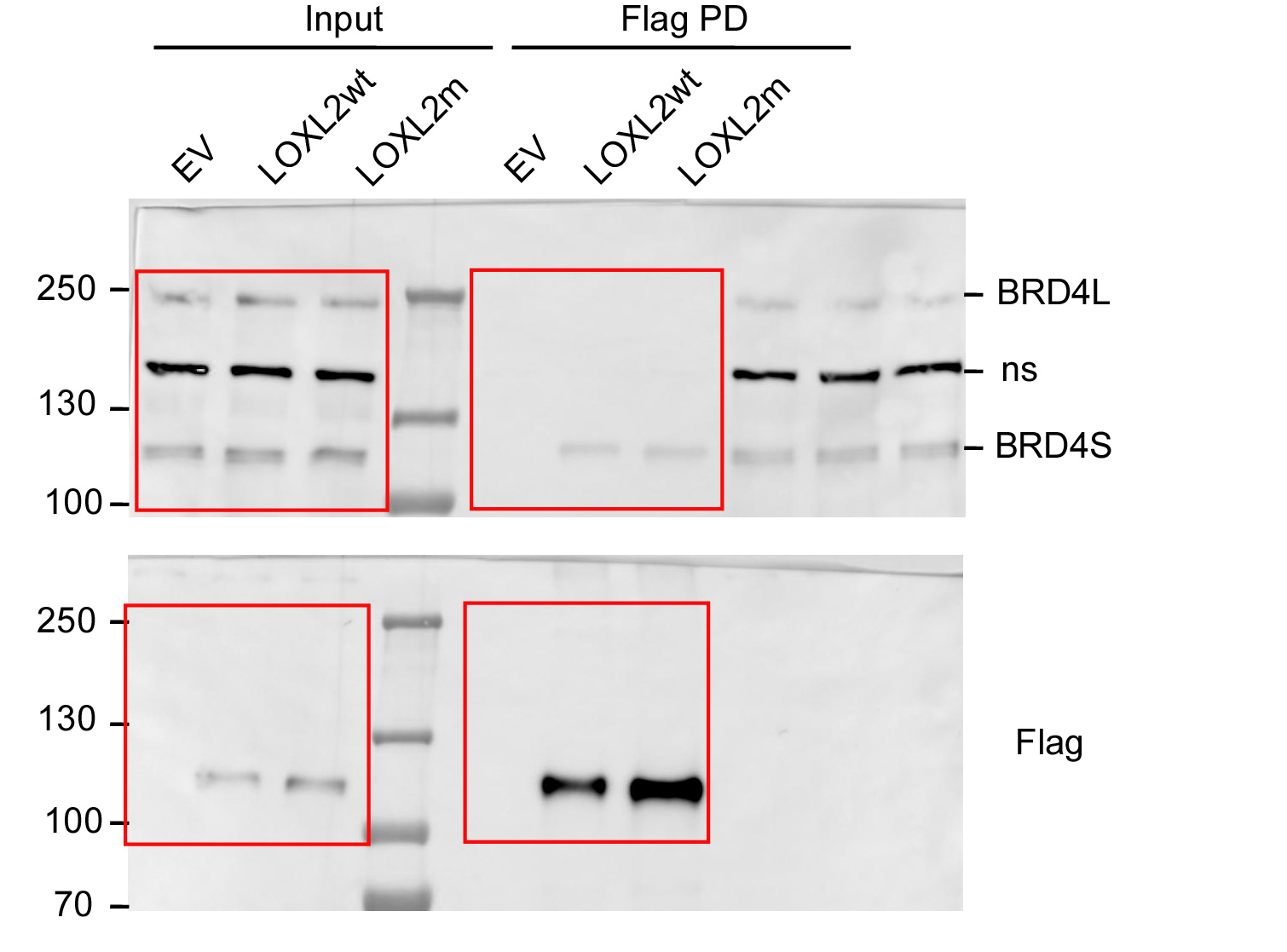

Supplement: Supplementary file 11 — Source Data for Figure 2 [file EMMM-15-e18459-s002.zip › Figure_2/2B/WB_2B.jpg]

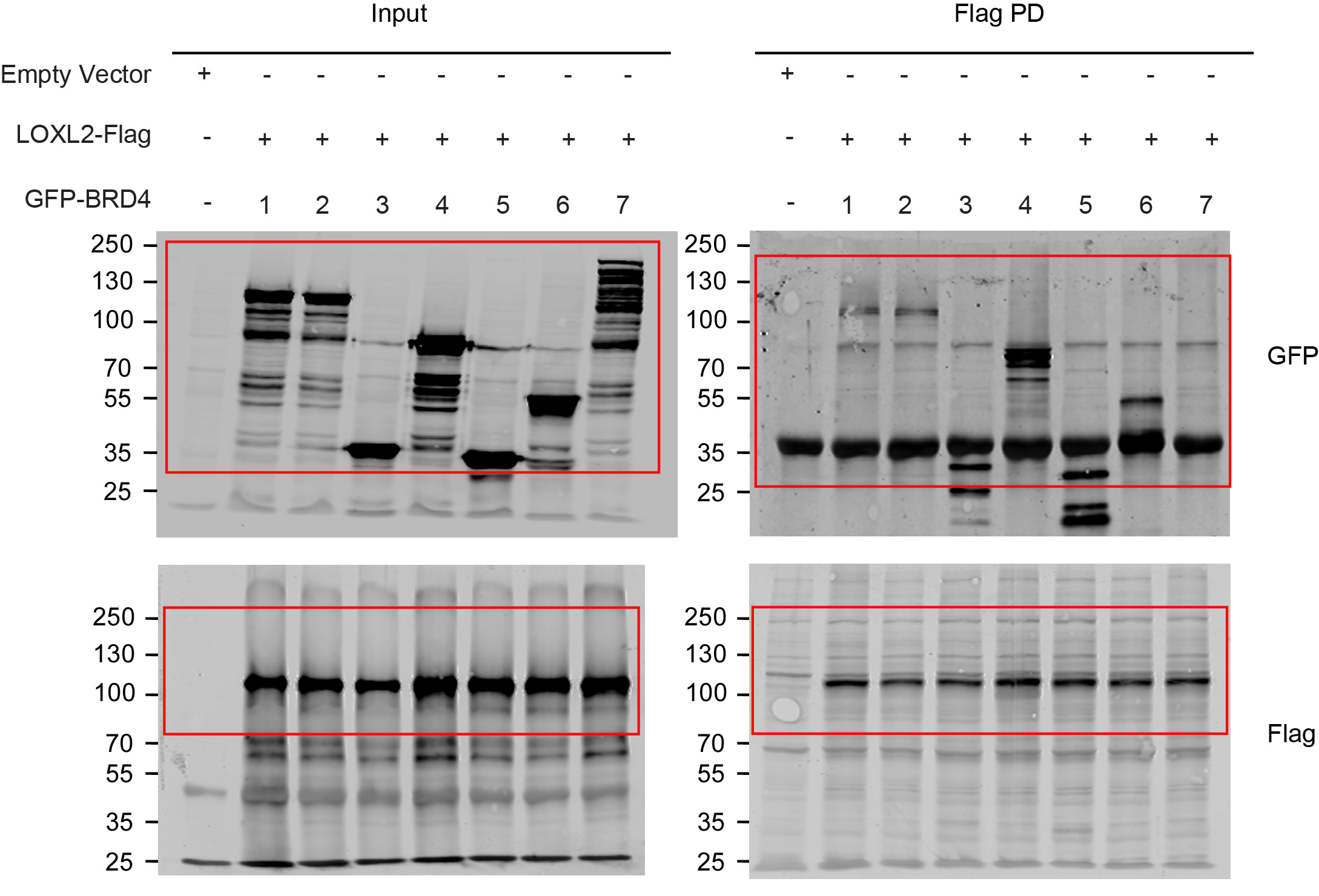

Supplement: Supplementary file 11 — Source Data for Figure 2 [file EMMM-15-e18459-s002.zip › Figure_2/2D/WB_2D.jpg]

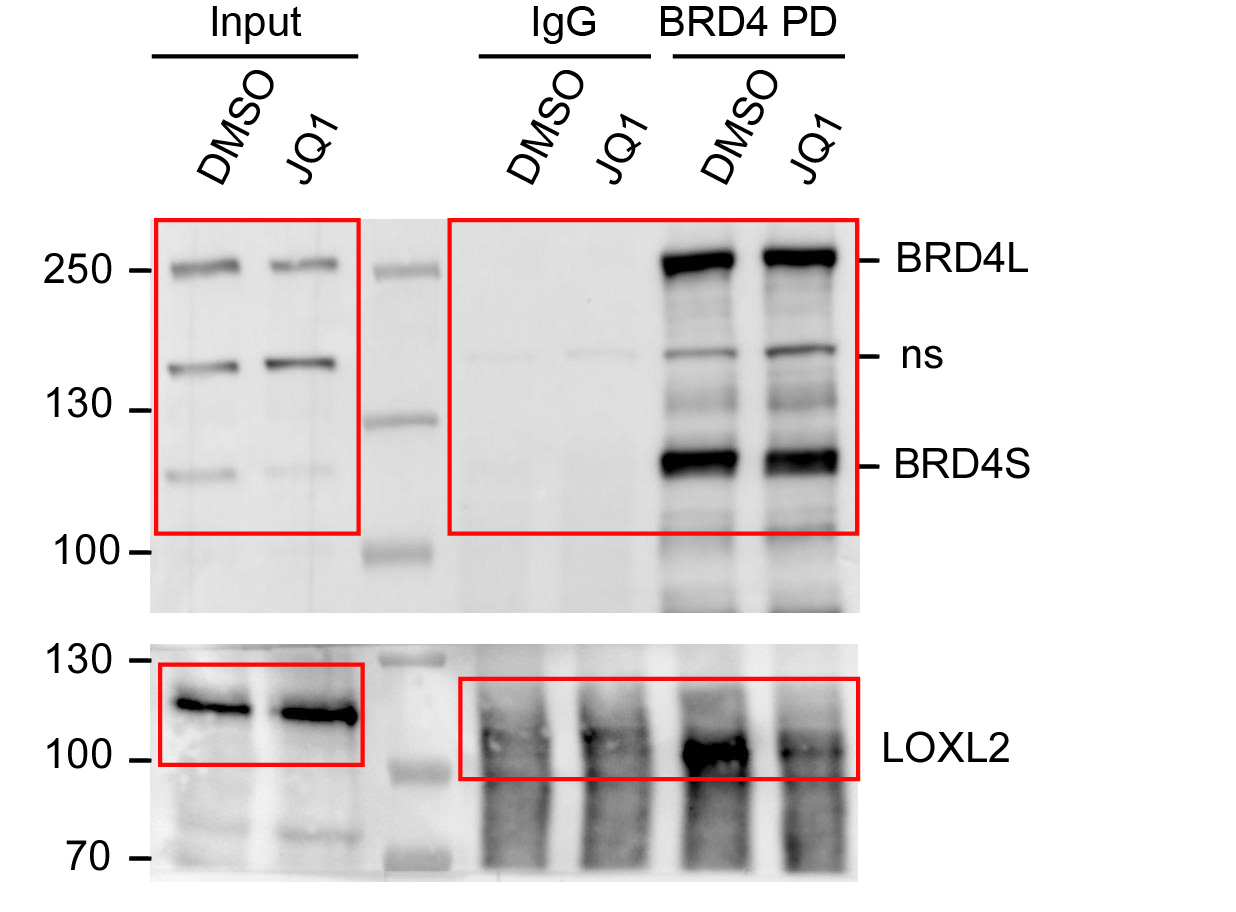

Supplement: Supplementary file 11 — Source Data for Figure 2 [file EMMM-15-e18459-s002.zip › Figure_2/2G/WB_2G.jpg]

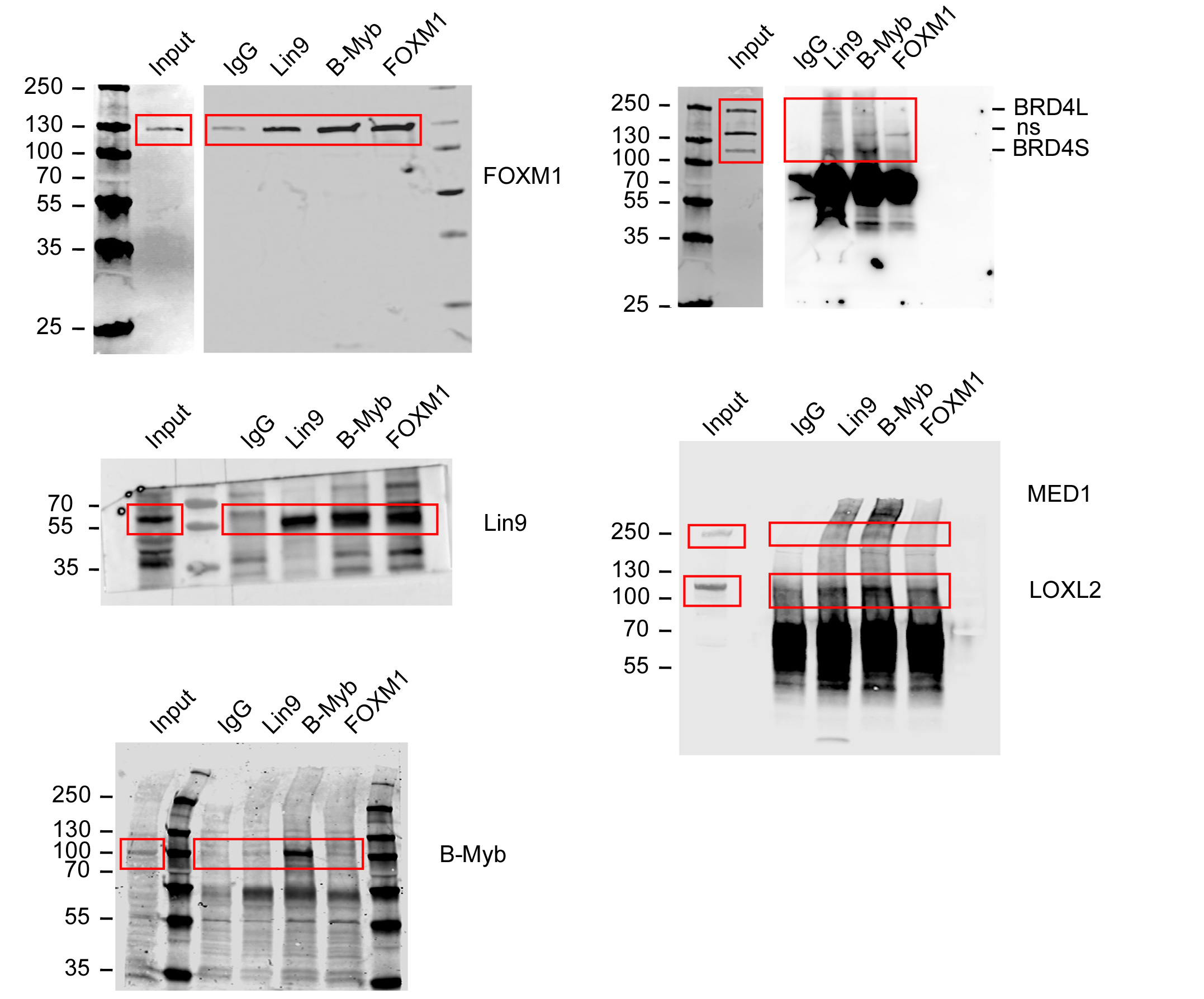

Supplement: Supplementary file 12 — Source Data for Figure 4 [file EMMM-15-e18459-s012.zip › Figure_4/4F/WB_4F.jpg]

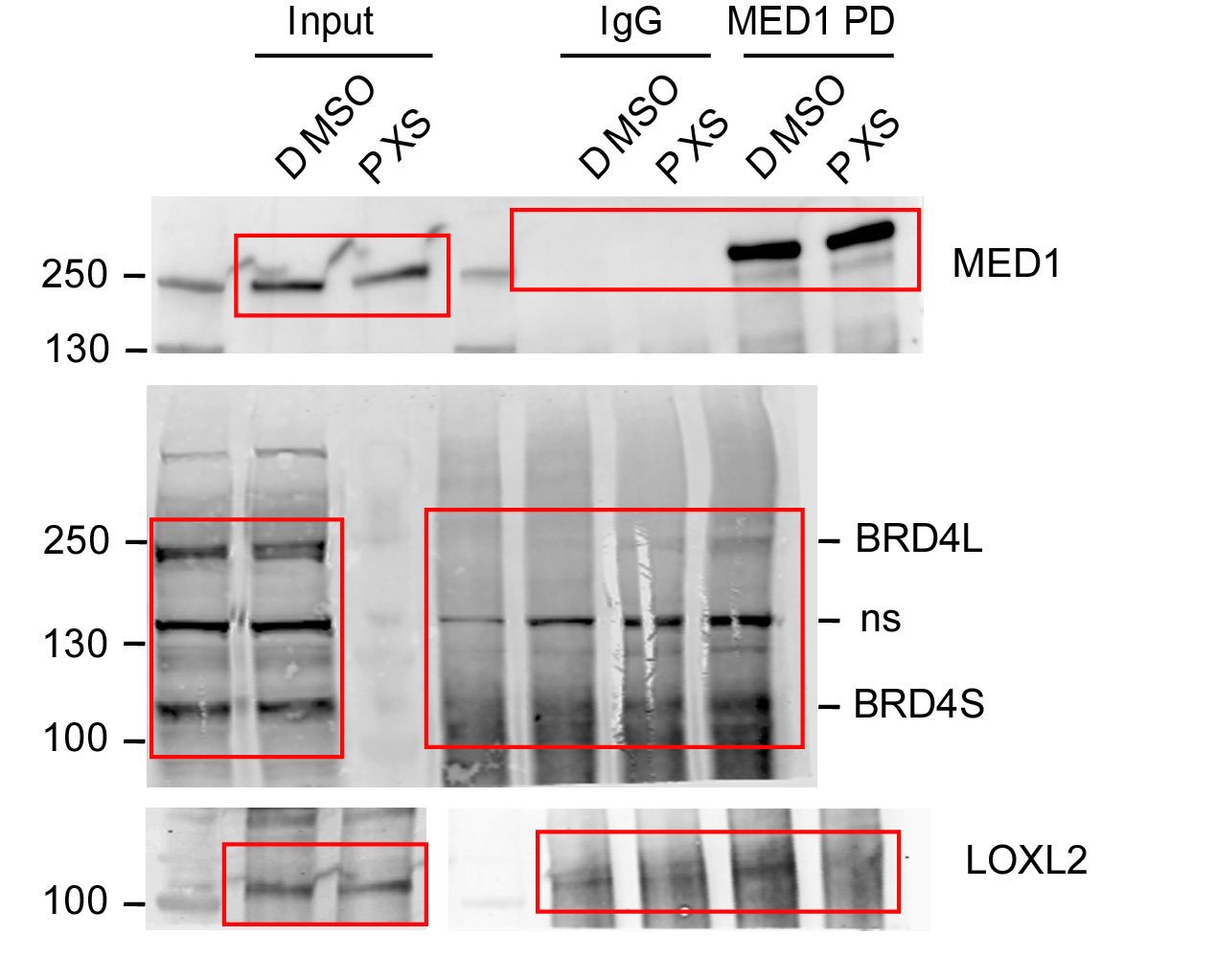

Supplement: Supplementary file 13 — Source Data for Figure 5 [file EMMM-15-e18459-s007.zip › Figure_5/5A/WB_5A.jpg]

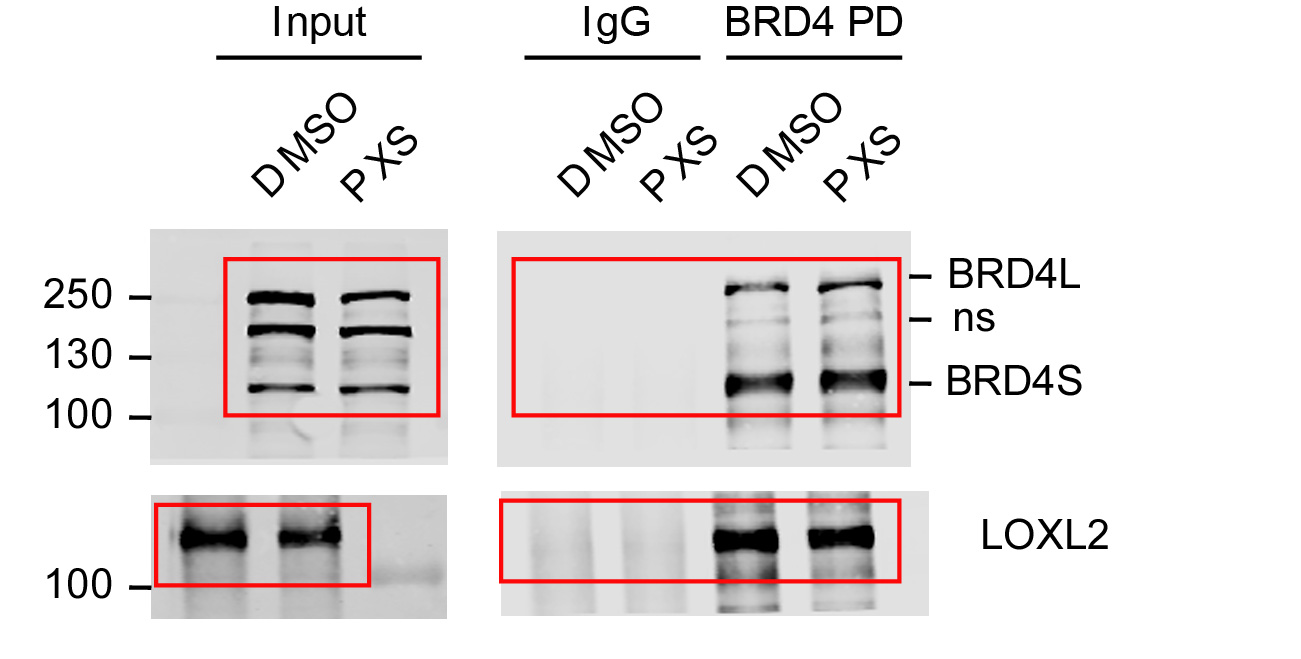

Supplement: Supplementary file 13 — Source Data for Figure 5 [file EMMM-15-e18459-s007.zip › Figure_5/5B/WB_5B.jpg]

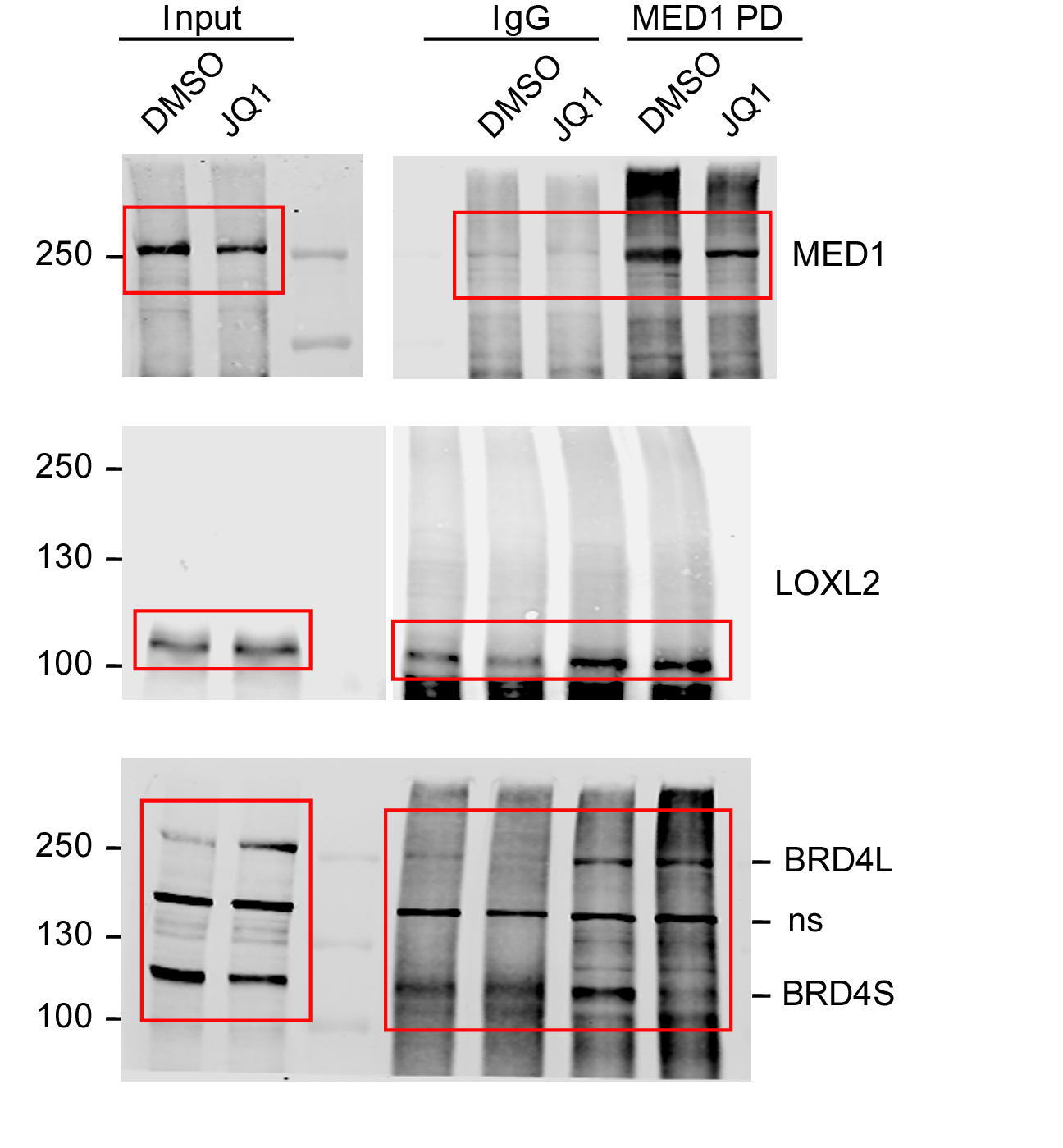

Supplement: Supplementary file 13 — Source Data for Figure 5 [file EMMM-15-e18459-s007.zip › Figure_5/5D/WB_5D.jpg]

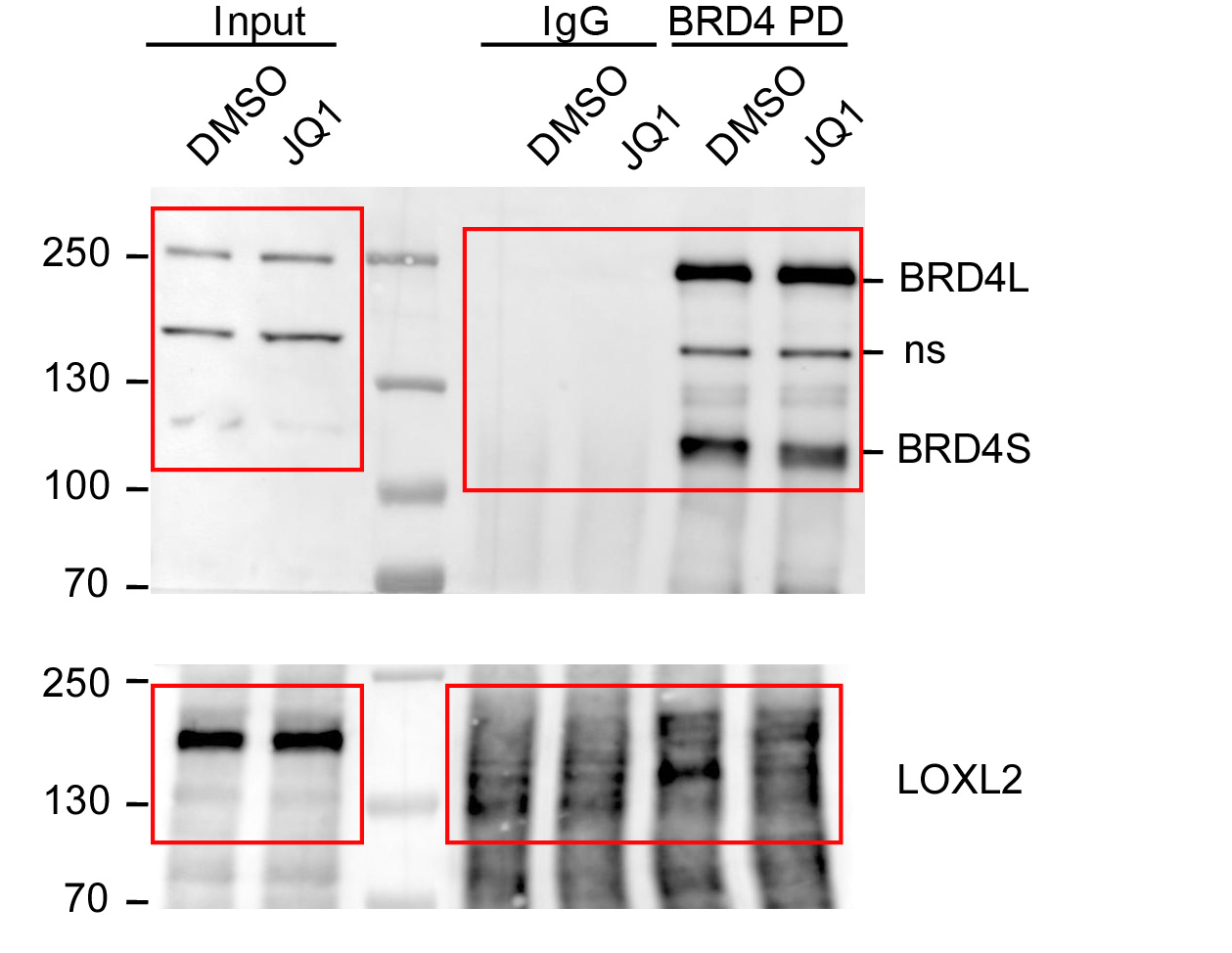

Supplement: Supplementary file 13 — Source Data for Figure 5 [file EMMM-15-e18459-s007.zip › Figure_5/5E/WB_5E.jpg]

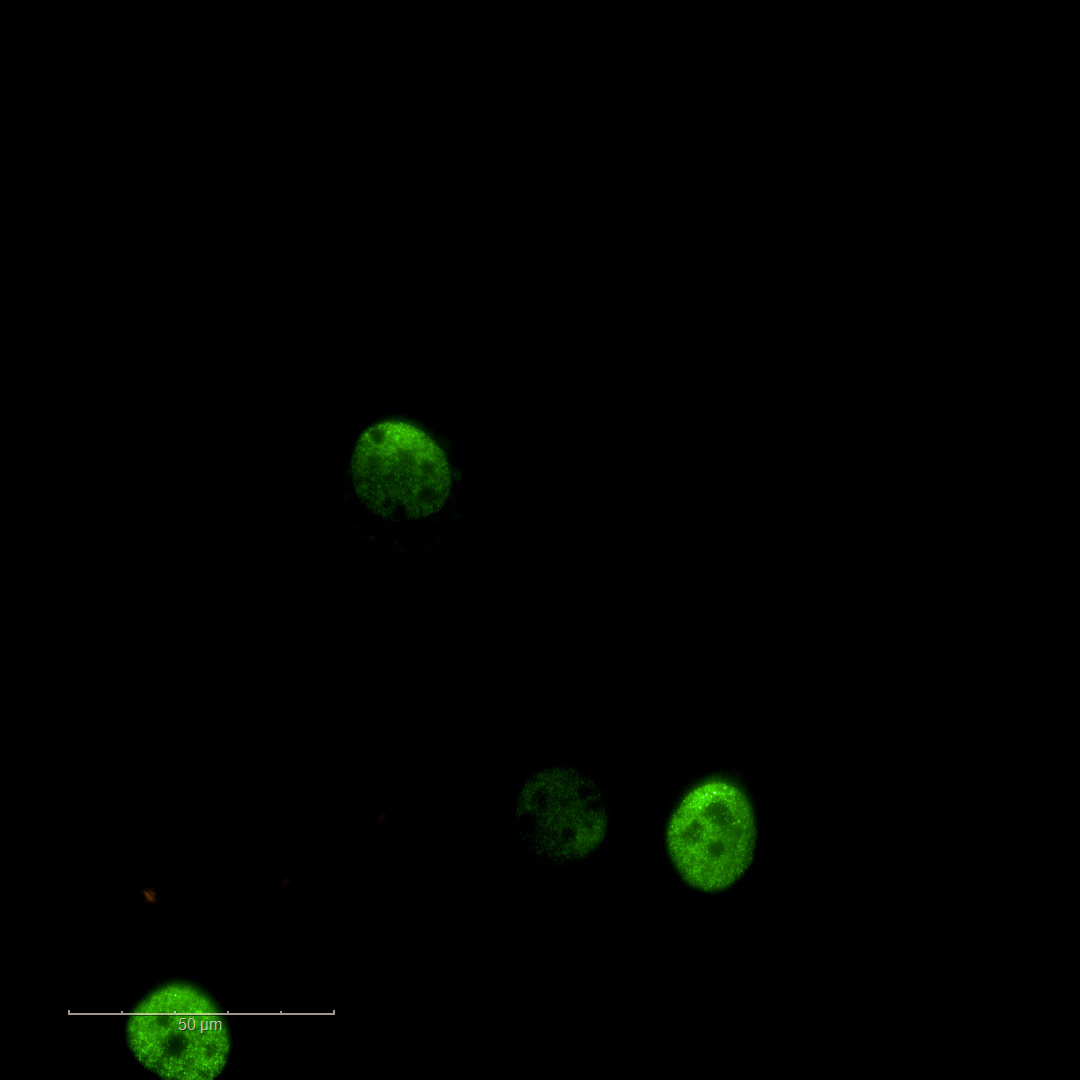

Supplement: Supplementary file 13 — Source Data for Figure 5 [file EMMM-15-e18459-s007.zip › Figure_5/5G/BRD4_Combo.tif]

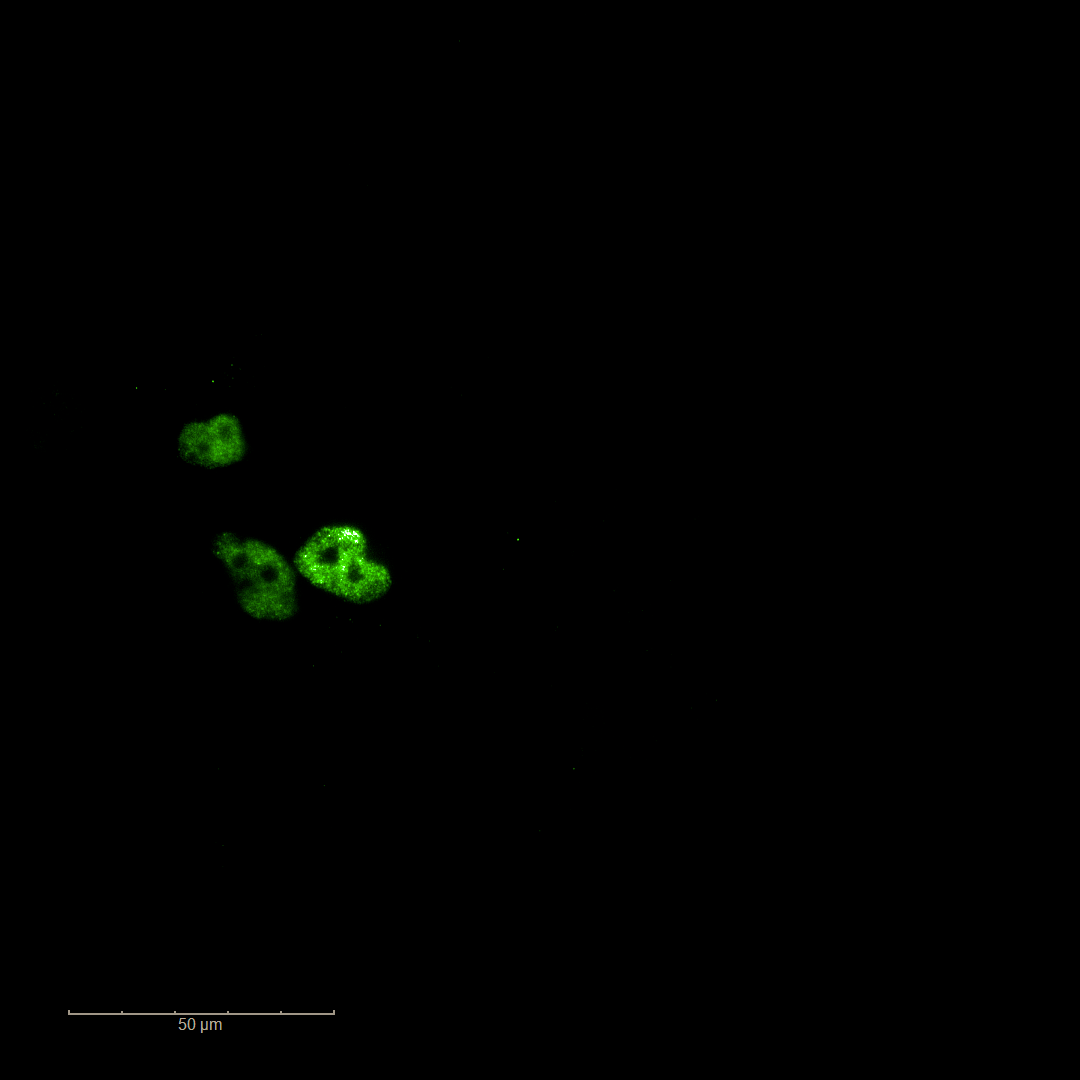

Supplement: Supplementary file 13 — Source Data for Figure 5 [file EMMM-15-e18459-s007.zip › Figure_5/5G/BRD4_DMSO.tif]

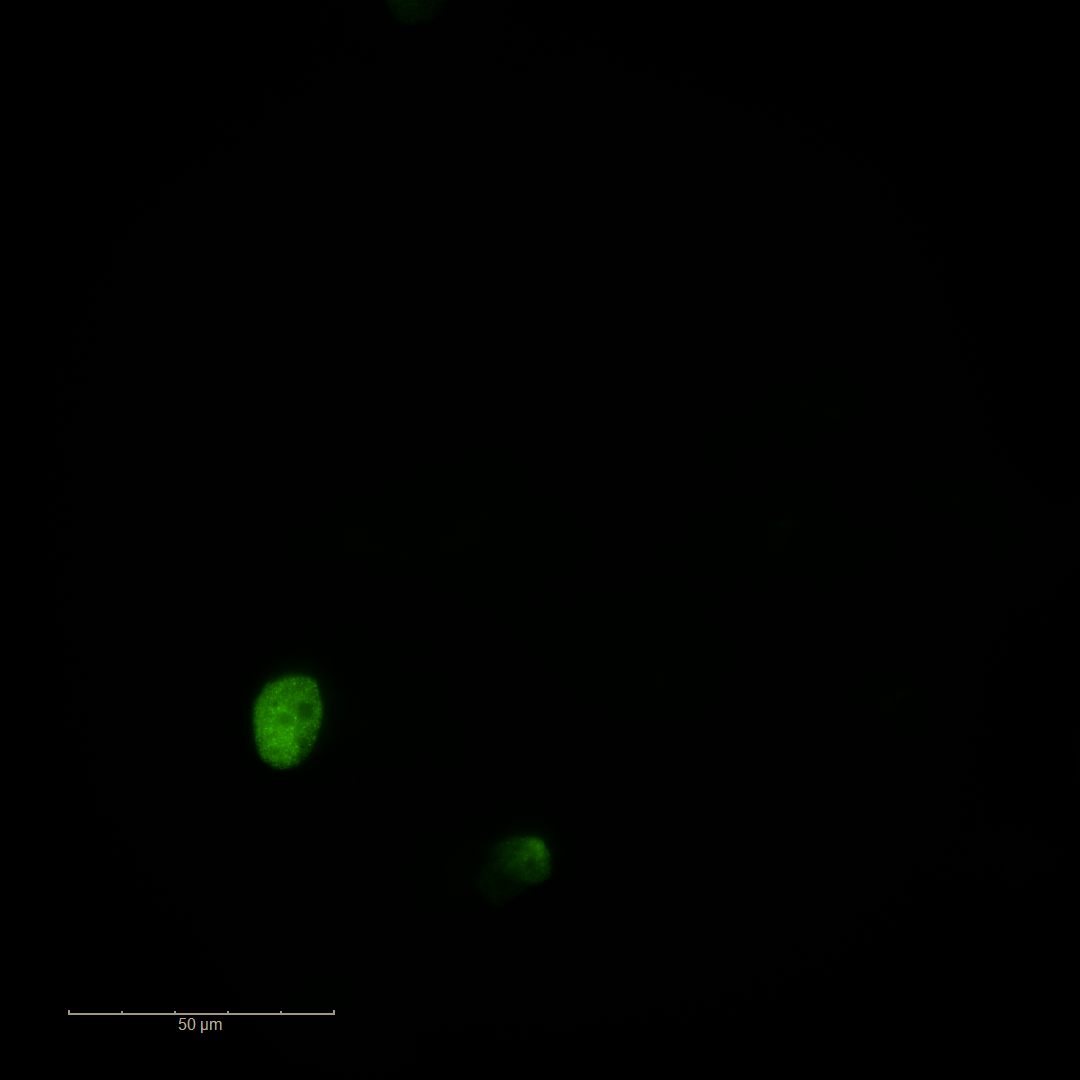

Supplement: Supplementary file 13 — Source Data for Figure 5 [file EMMM-15-e18459-s007.zip › Figure_5/5G/BRD4_JQ1.tif]

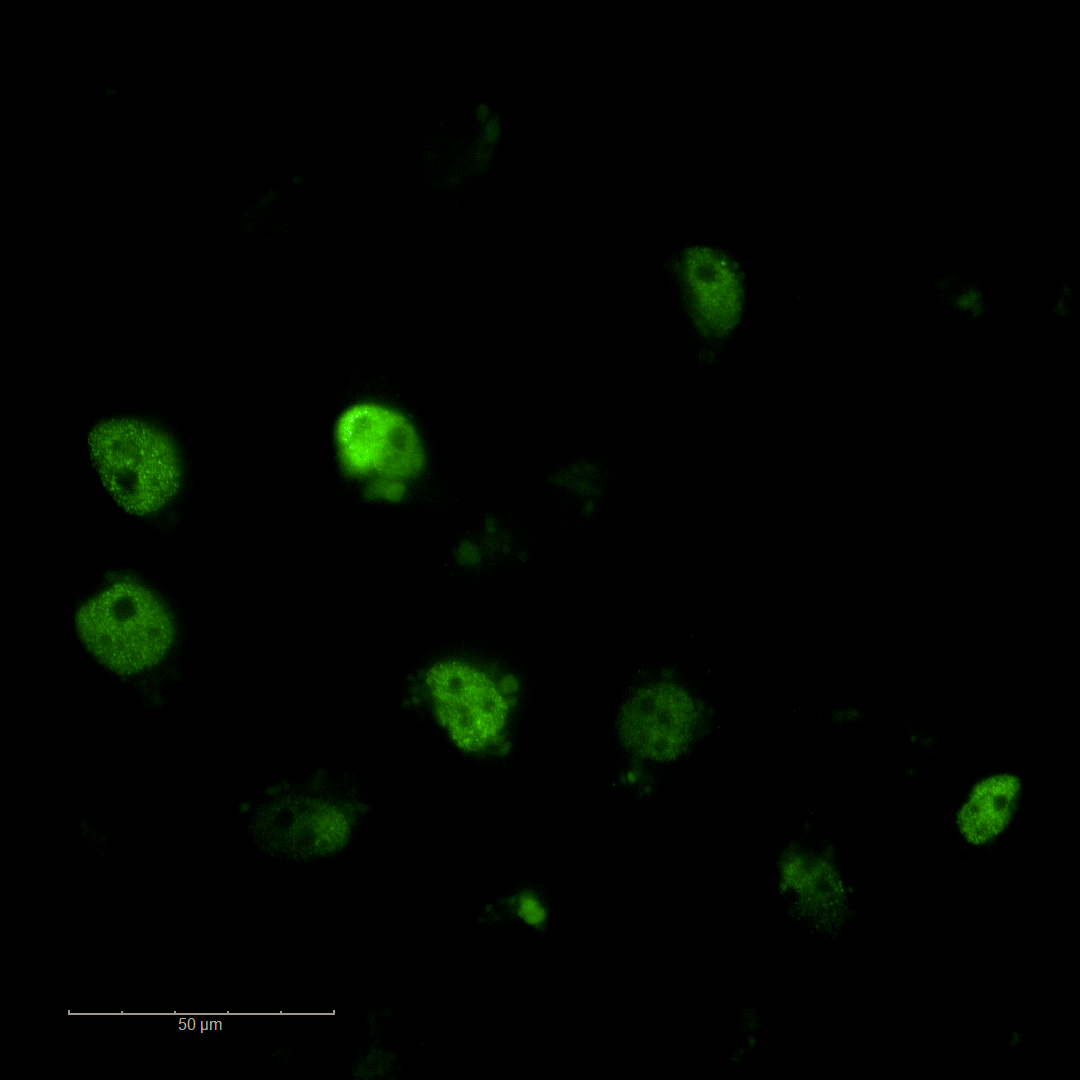

Supplement: Supplementary file 13 — Source Data for Figure 5 [file EMMM-15-e18459-s007.zip › Figure_5/5G/BRD4_PXS.tif]

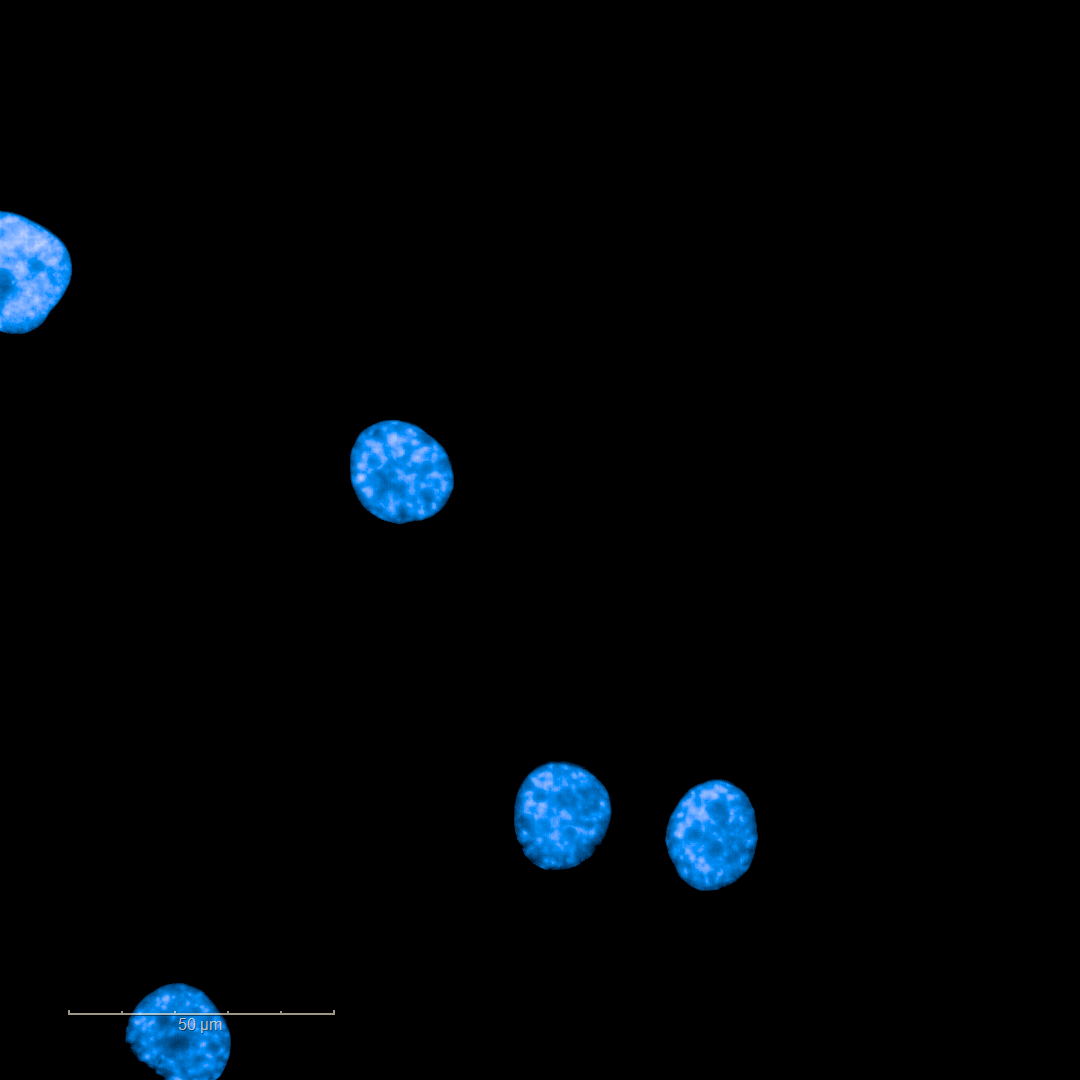

Supplement: Supplementary file 13 — Source Data for Figure 5 [file EMMM-15-e18459-s007.zip › Figure_5/5G/Dapi_Combo.tif]

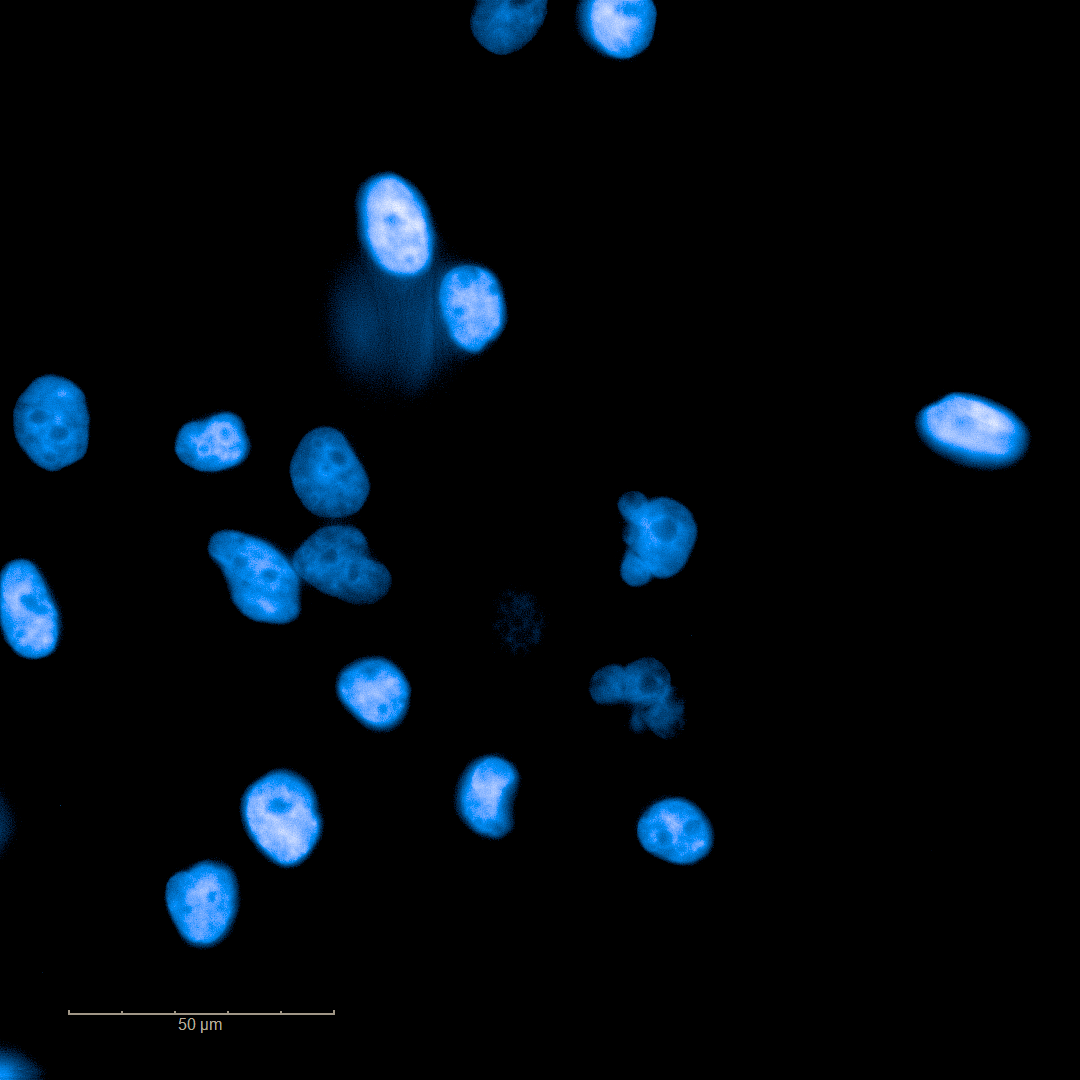

Supplement: Supplementary file 13 — Source Data for Figure 5 [file EMMM-15-e18459-s007.zip › Figure_5/5G/Dapi_DMSO.tif]

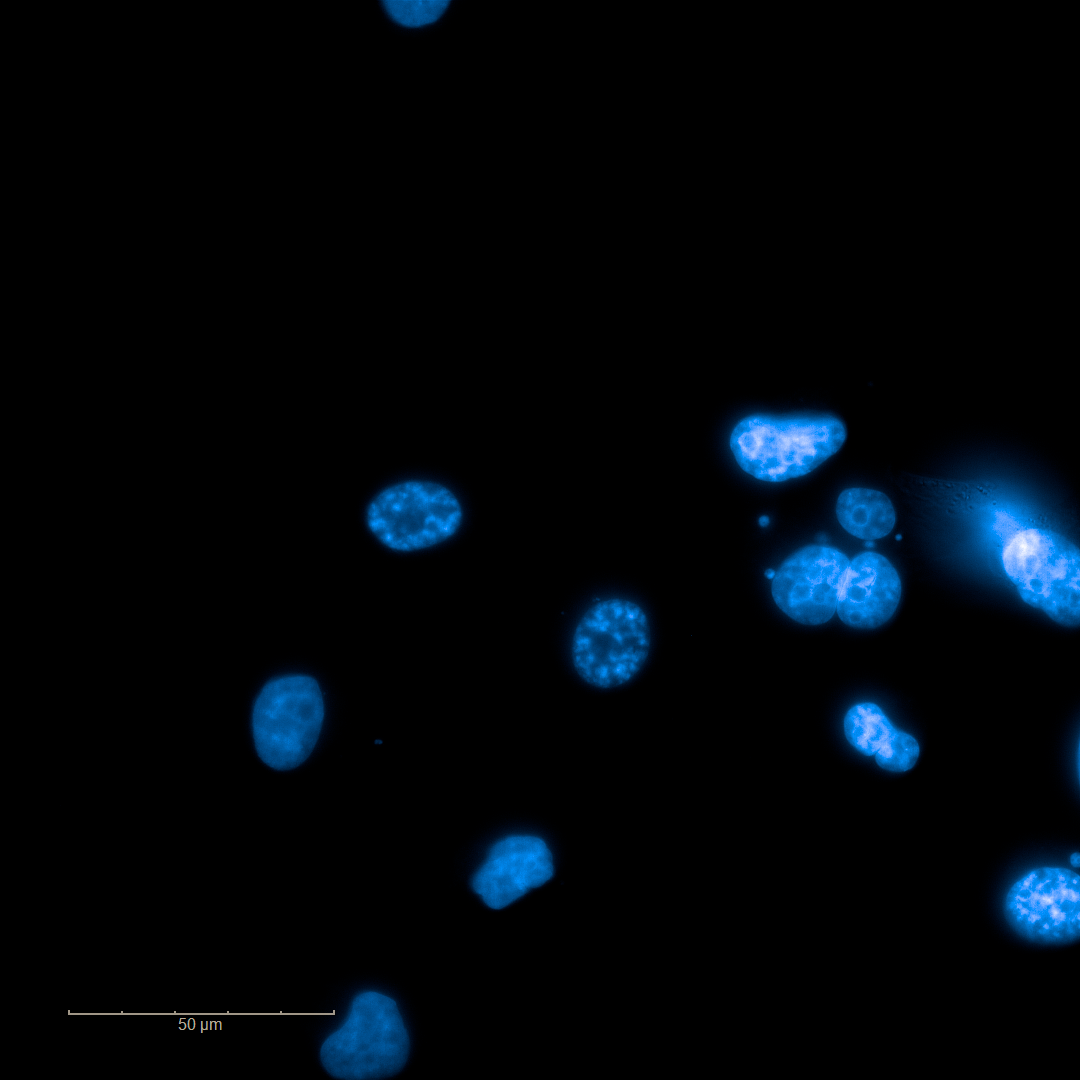

Supplement: Supplementary file 13 — Source Data for Figure 5 [file EMMM-15-e18459-s007.zip › Figure_5/5G/Dapi_JQ1.tif]

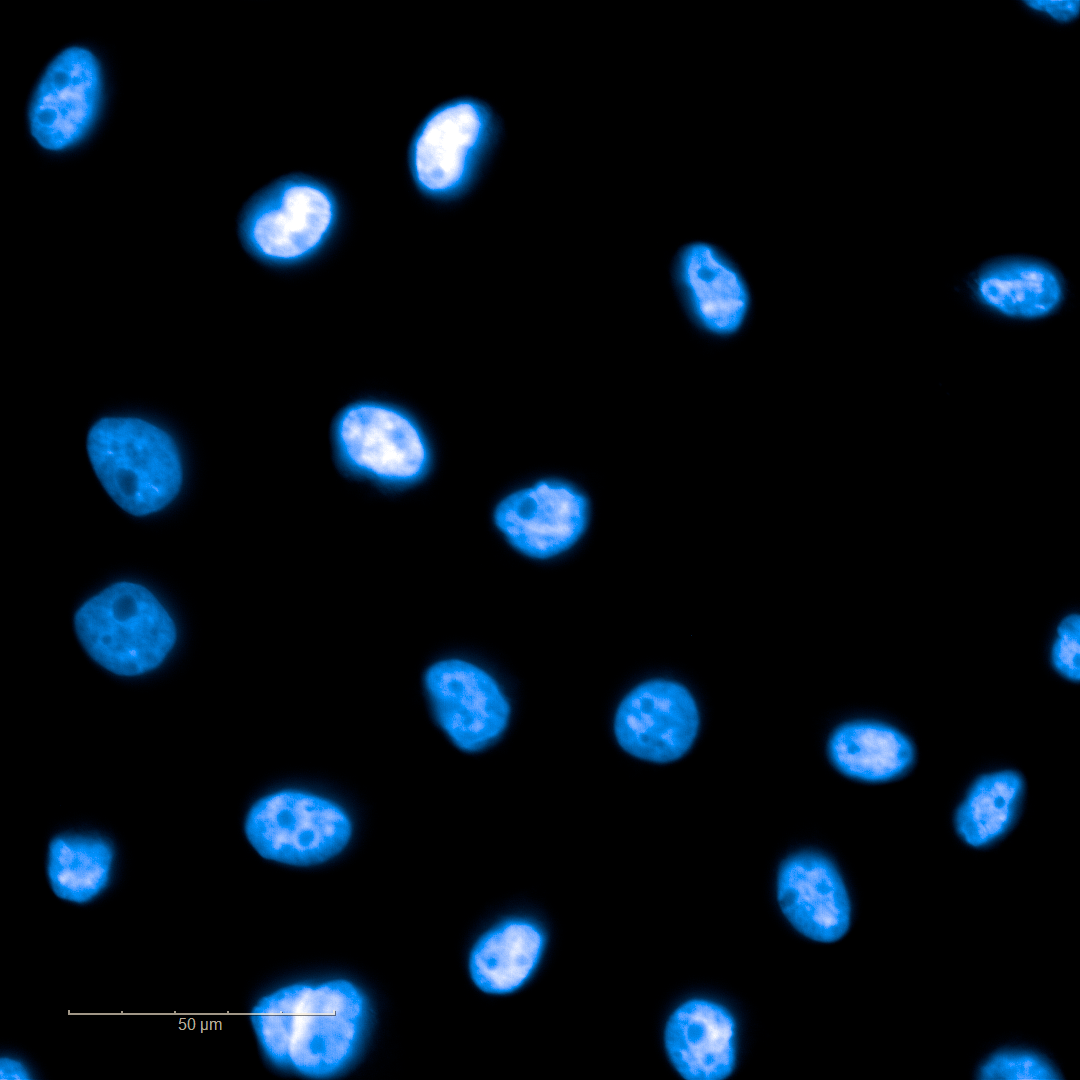

Supplement: Supplementary file 13 — Source Data for Figure 5 [file EMMM-15-e18459-s007.zip › Figure_5/5G/Dapi_PXS.tif]

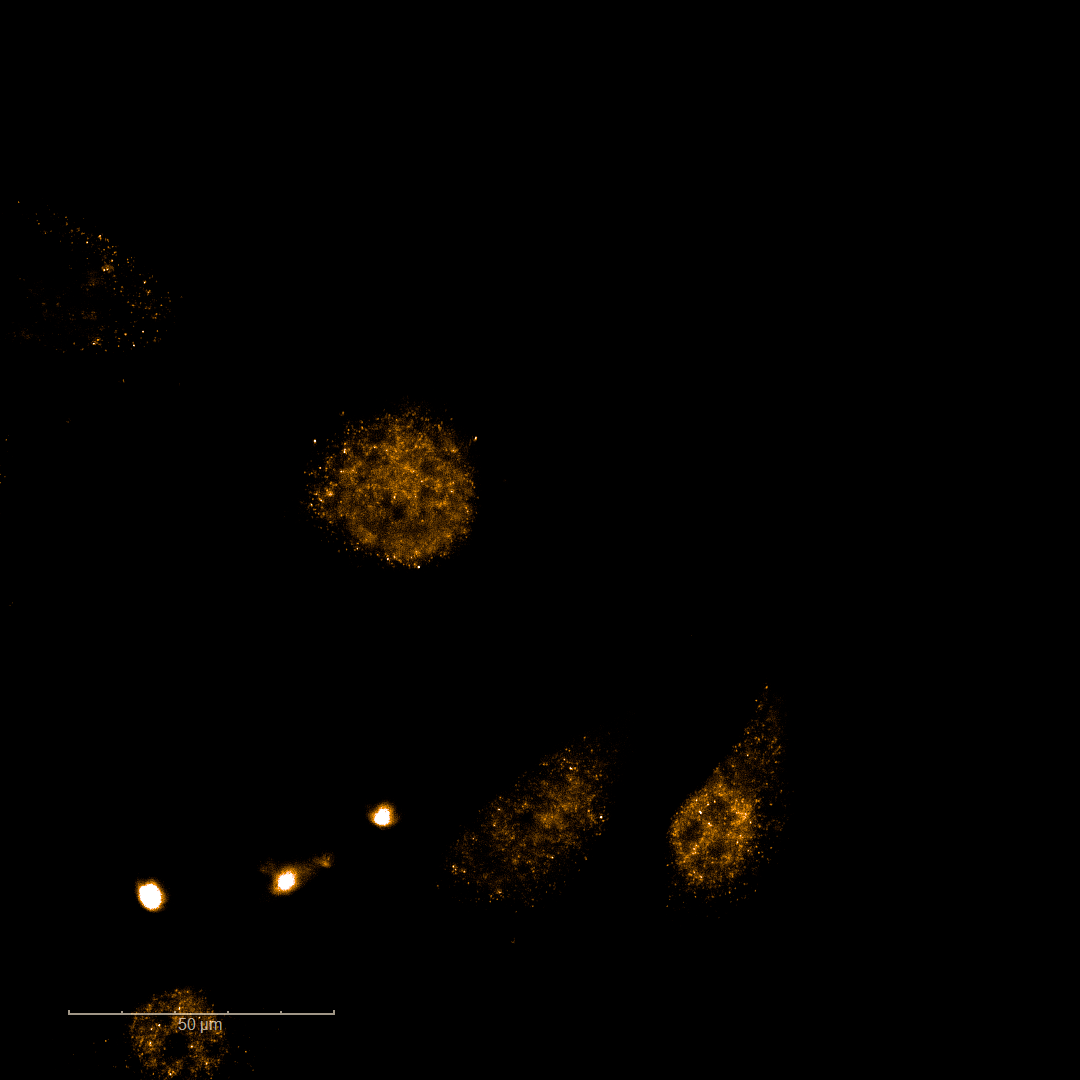

Supplement: Supplementary file 13 — Source Data for Figure 5 [file EMMM-15-e18459-s007.zip › Figure_5/5G/MED1_Combo.tif]

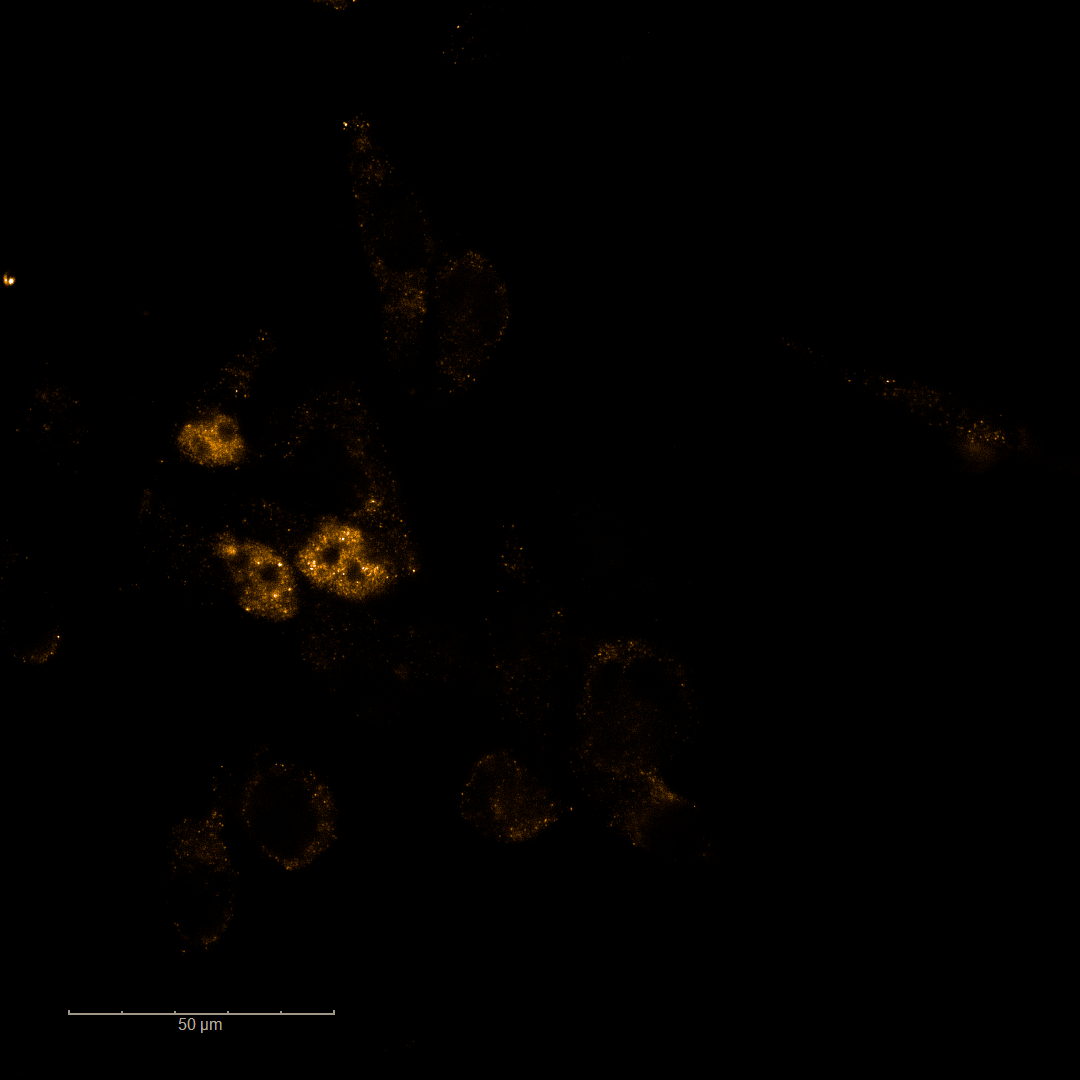

Supplement: Supplementary file 13 — Source Data for Figure 5 [file EMMM-15-e18459-s007.zip › Figure_5/5G/MED1_DMSO.tif]

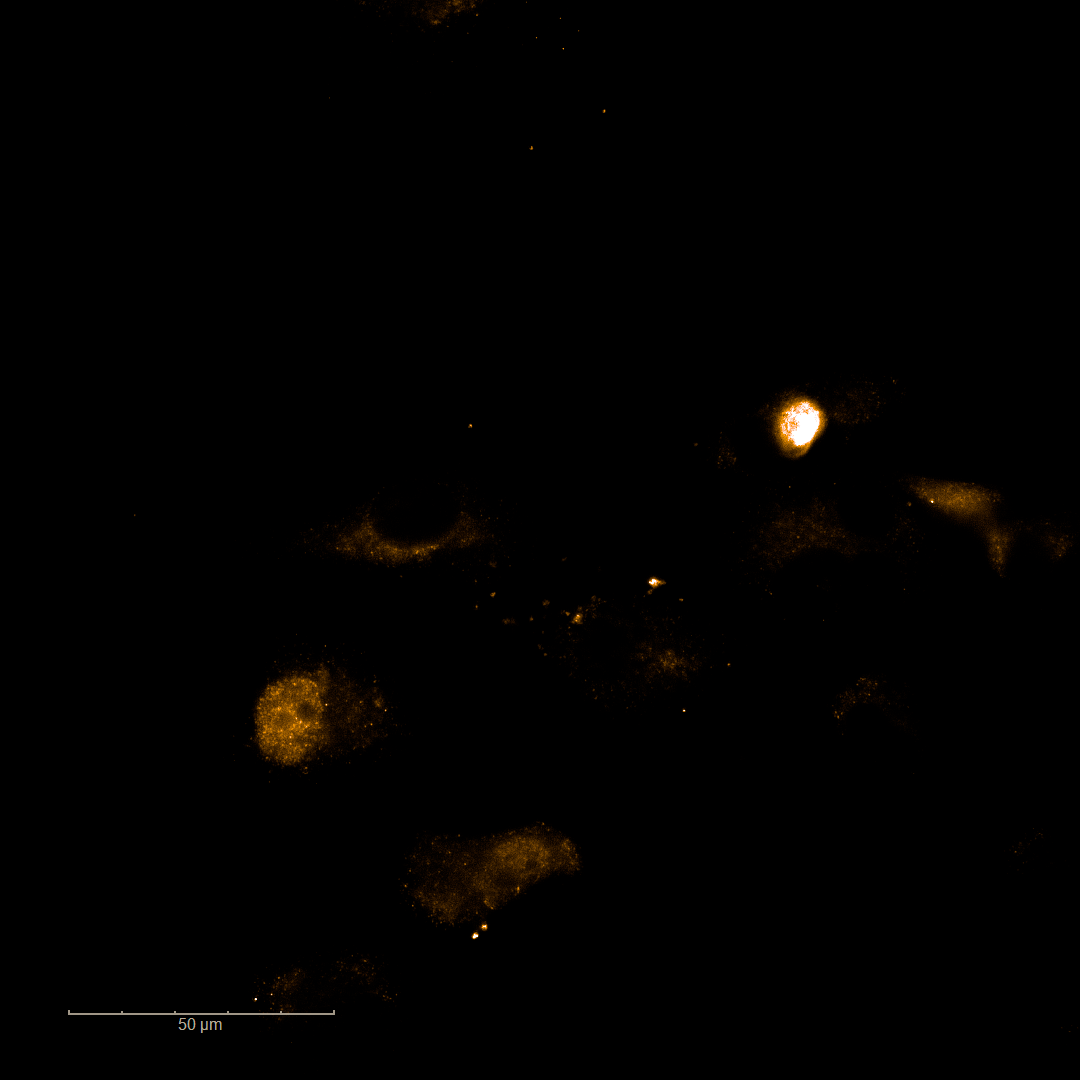

Supplement: Supplementary file 13 — Source Data for Figure 5 [file EMMM-15-e18459-s007.zip › Figure_5/5G/MED1_JQ1.tif]

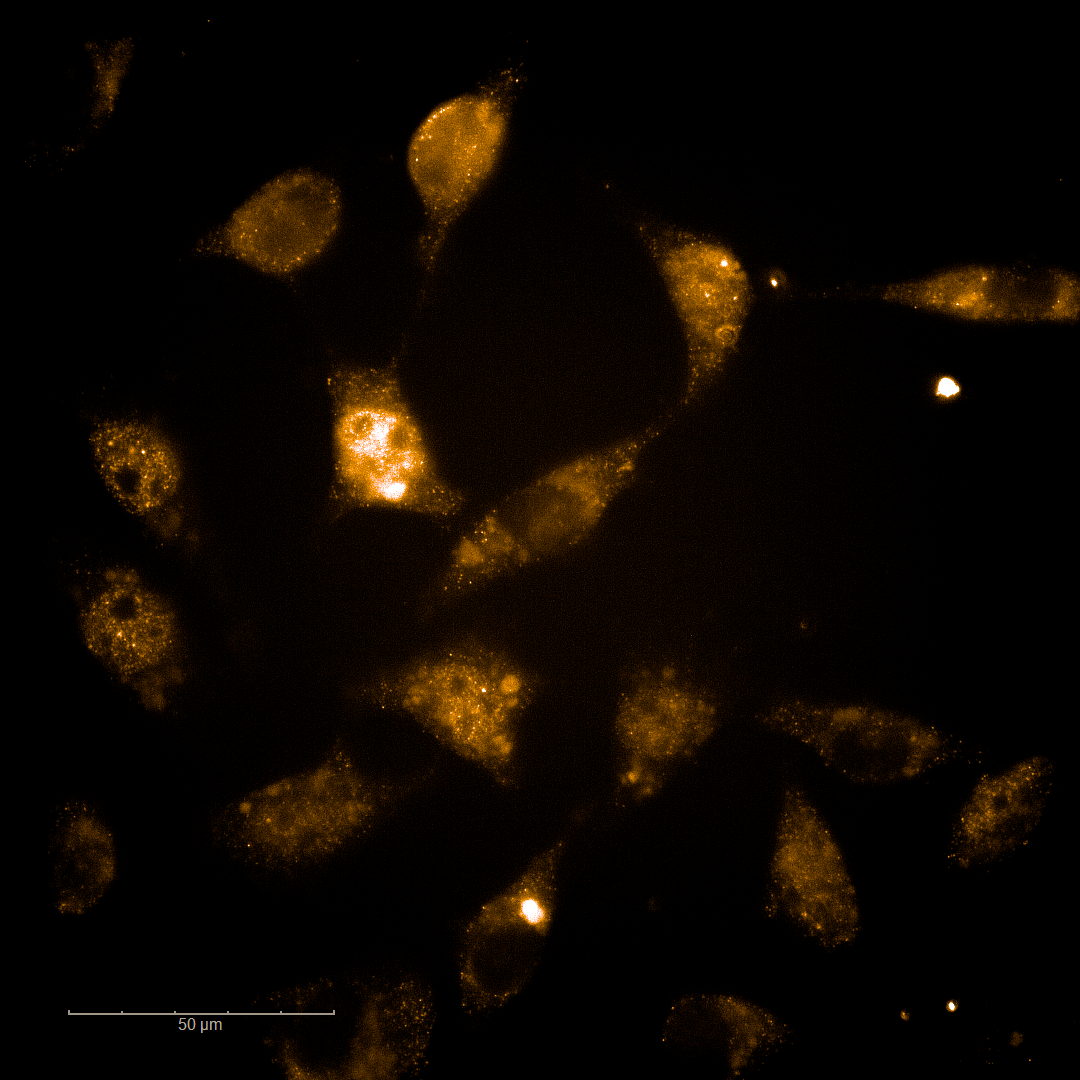

Supplement: Supplementary file 13 — Source Data for Figure 5 [file EMMM-15-e18459-s007.zip › Figure_5/5G/MED1_PXS.tif]

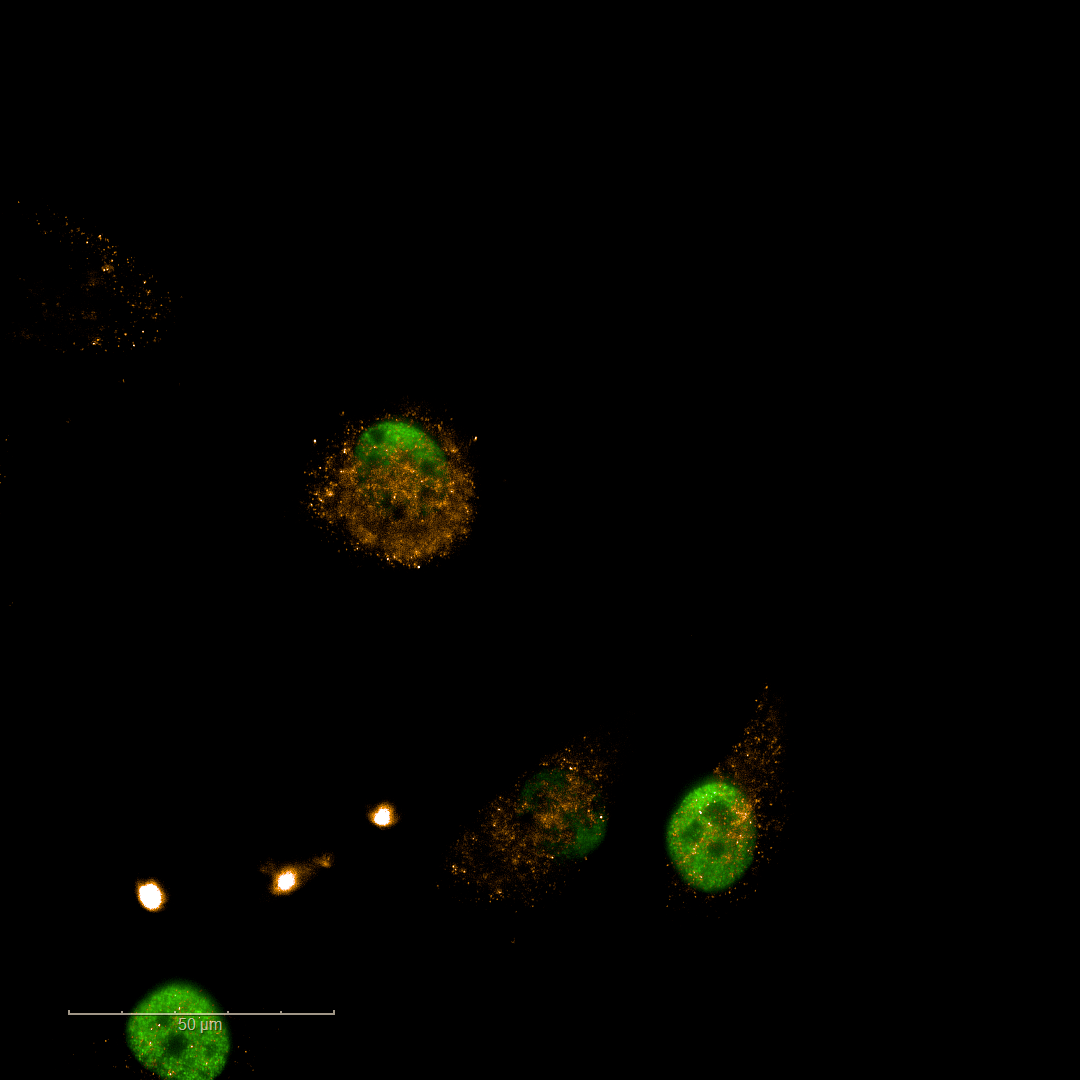

Supplement: Supplementary file 13 — Source Data for Figure 5 [file EMMM-15-e18459-s007.zip › Figure_5/5G/Overlay_Combo.tif]

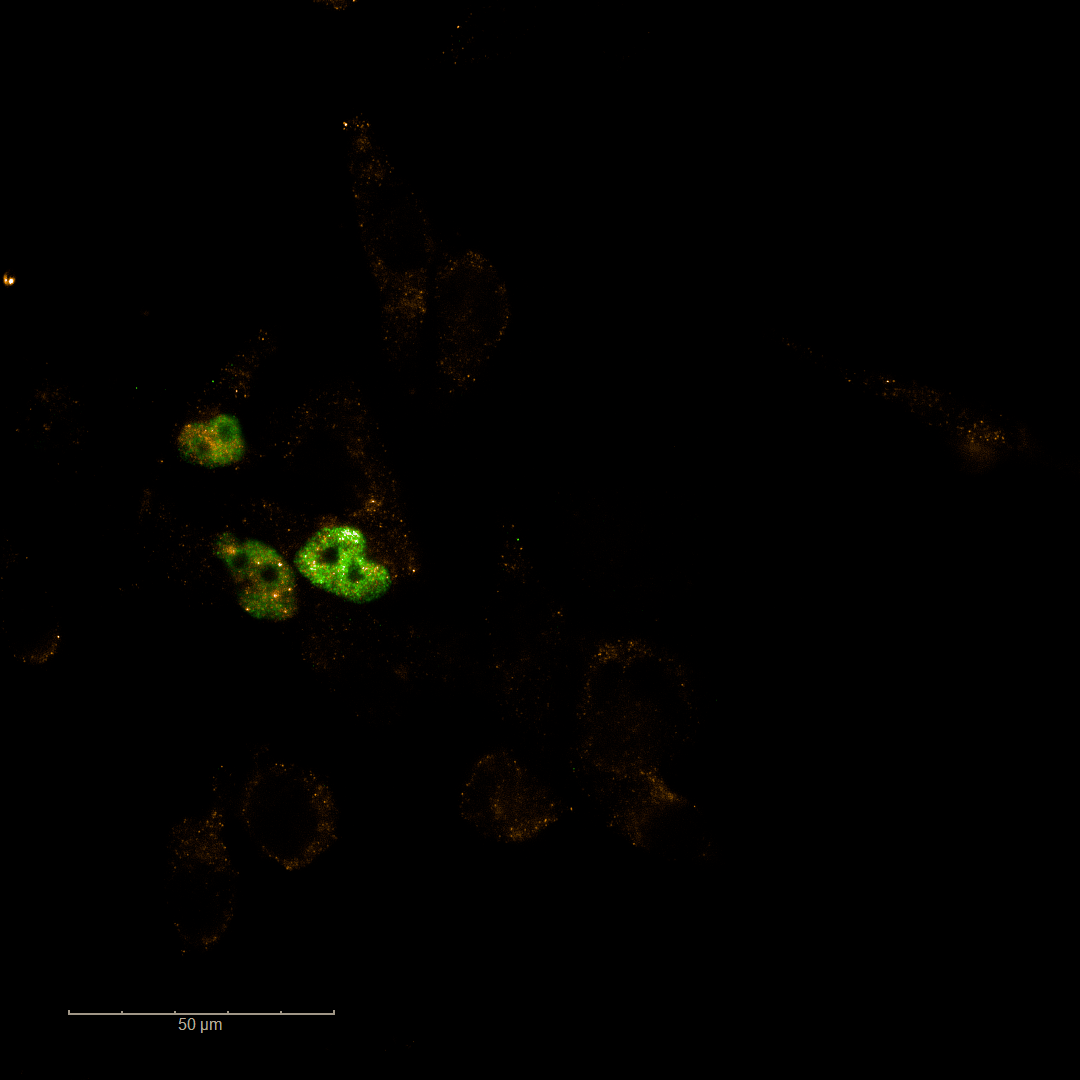

Supplement: Supplementary file 13 — Source Data for Figure 5 [file EMMM-15-e18459-s007.zip › Figure_5/5G/Overlay_DMSO.tif]

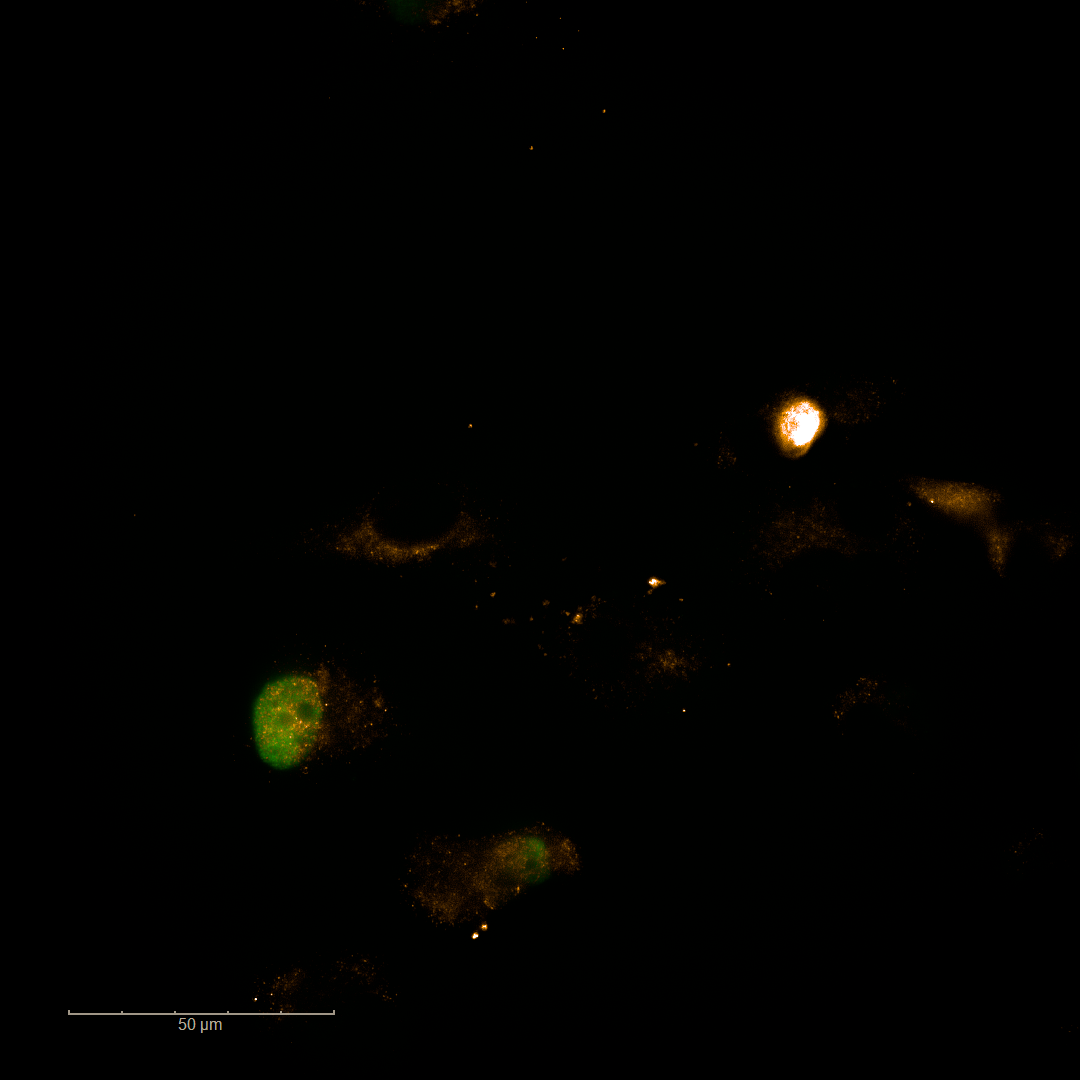

Supplement: Supplementary file 13 — Source Data for Figure 5 [file EMMM-15-e18459-s007.zip › Figure_5/5G/Overlay_JQ1.tif]

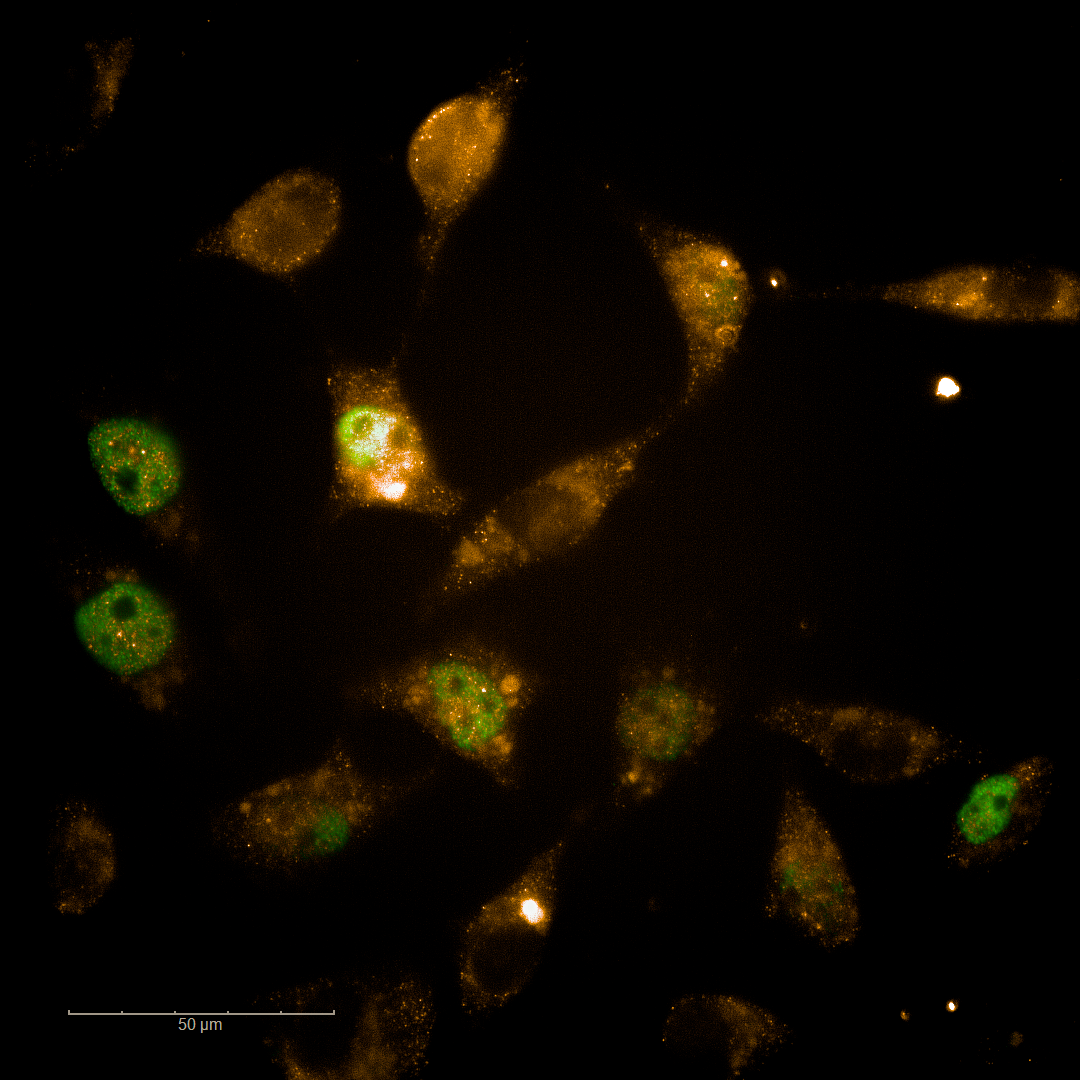

Supplement: Supplementary file 13 — Source Data for Figure 5 [file EMMM-15-e18459-s007.zip › Figure_5/5G/Overlay_PXS.tif]

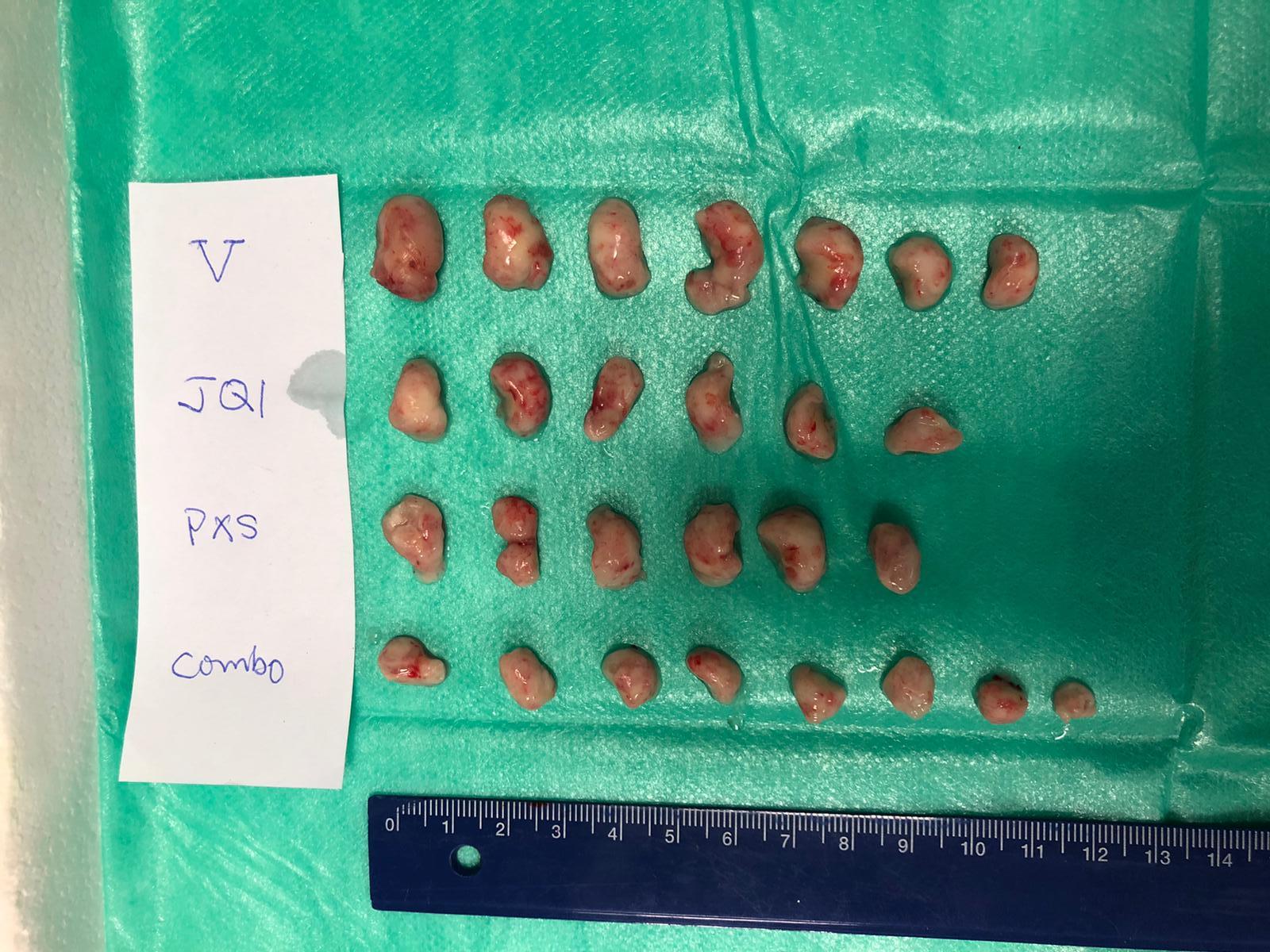

Supplement: Supplementary file 14 — Source Data for Figure 6 [file EMMM-15-e18459-s015.zip › Figure_6/6B/Tumor_pictures.jpg]

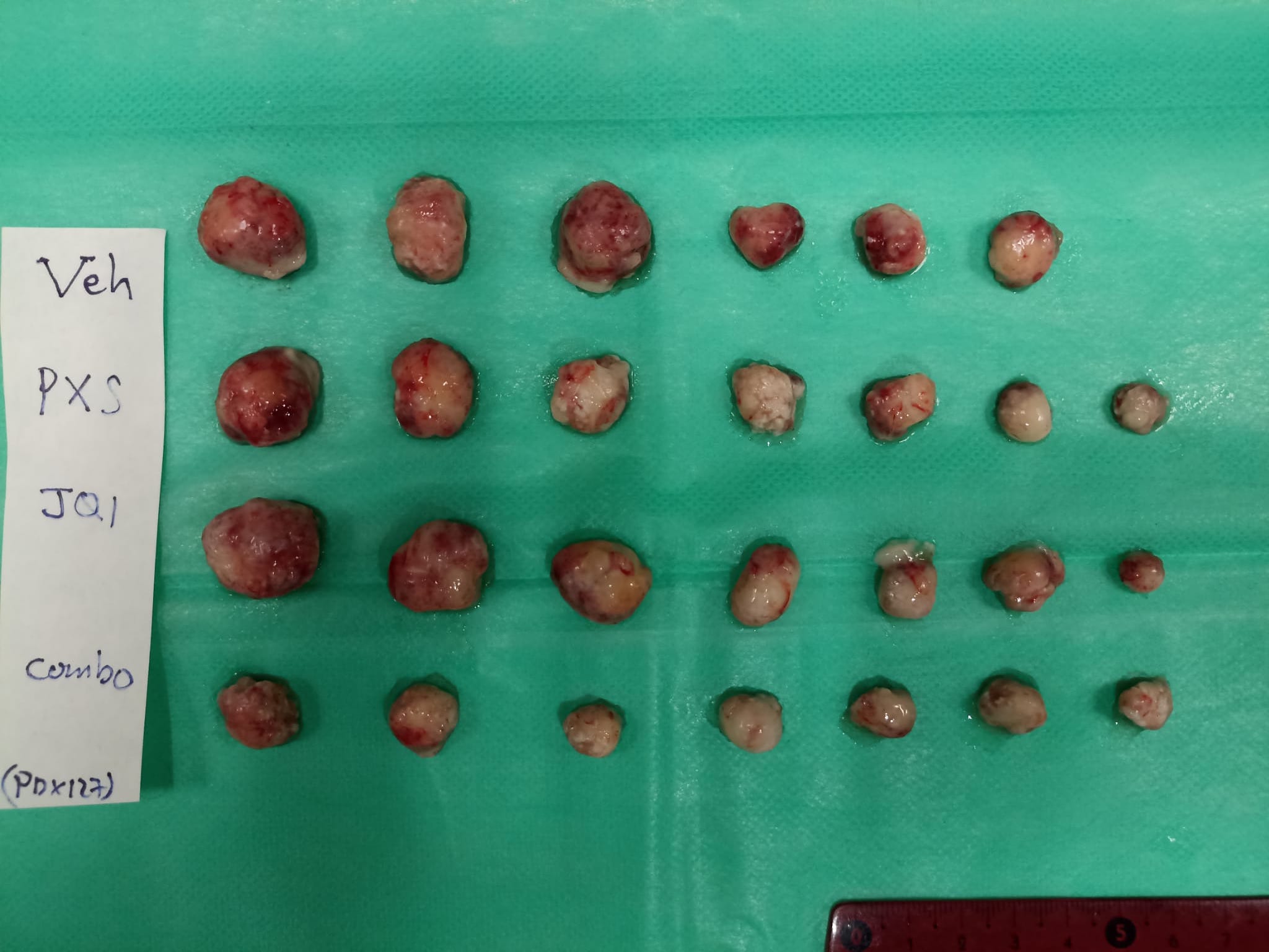

Supplement: Supplementary file 14 — Source Data for Figure 6 [file EMMM-15-e18459-s015.zip › Figure_6/6D/Tumor_pictures.jpeg]
