# Supplementary material for: Interactions between BRD4S, LOXL2, and MED1 drive cell cycle transcription in triple‐negative breast cancer
Source: EMBO Mol Med. 2023 Nov 8;15(12):e18459. doi: 10.15252/emmm.202318459 (PMC10701626; doi:10.15252/emmm.202318459)
Supplement: Supplementary file 15 [file EMMM-15-e18459-s014.pdf]

EMBO Molecular Medicine

Interactions between BRD4S, LOXL2, and MED1 drive cell cycle transcription in triple-negative breast cancer

Laura Pascual-Reguant, Queralt Serra Camprubi, Debayan Datta, Damiano Cianferoni, Savvas Kourtis, Antoni Gañez Zapater, Chiara Cannatá, Lorena Espinar, Jessica Querol, Laura García-López, Sara Musa-Afaneh, Maria Guirola, Anestis Gkanogiannis, Andrea Miro Canturri, Marta Guzmán, Olga Rodríguez, Andrea Herencia-Ropero, Joaquin Arribas, Violeta Serra, Luis Serrano, Tian Tian, Sandra Peiró, and Sara Sdelci
DOI: 10.15252/emmm.202318459

Corresponding author(s): Sara Sdelci (sara.sdelci@crg.eu) , Sandra Peiró (sandra.peiro.sales@gmail.com)

Review Timeline:

Transfer from Review Commons:	3rd Aug 23
Editorial Decision:	19th Sep 23
Revision Received:	11th Oct 23
Editorial Decision:	13th Oct 23
Revision Received:	16th Oct 23
Accepted:	17th Oct 23

Editor: Lise Roth

Review COMMONS

Transaction Report: This manuscript was transferred to EMBO Molecular Medicine following peer review at Review Commons.

Review #1

1. Evidence, reproducibility and clarity:

Evidence, reproducibility and clarity (Required)

The manuscript describes that simultaneous inhibition of LOXL2 and BRD4 reduces proliferation of TNBC in vitro and reduces growth in vivo.
This observation is followed by extensive mechanistic studies that suggest physical interaction between LOXL2 and short isoform of BRD4-MED1. Inferences from Chip-seq analyses suggest that this interaction is involved in regulation of multiple transcriptional programs. Authors focus on differential activation of DREAM complex, to claim that this interaction "is fundamental for proliferation of TNBC". The manuscript is very well written and mechanistic inferences are based on a set of sophisticated epigenetic analyses and bioinformatical inferences. The phenotypic effects from LoxL2 inhibition by itself, or in combination with BRD4 inhibition are relatively modest. These modest effects, as well as many of the reported changes in gene expression are clearly inconsistent with the frequently used adjectives as "dramatic", "fundamental", "deeply affected", "drastically hampered" etc. Given the modest phenotypic effects, many of the key claims and conclusions are not supported by the data.

Specifically:

1. It is unclear why authors generalize their conclusions to TNBC. Figure 1B demonstrates synergy for 1/3 cell lines, which is chosen for the follow up study. Even for MDA231, the synergy is confined to low concentrations of BRD4i (S1c). While MDA231 cell line is frequently used in experimental studies of TNBC, it is quite dissimilar to majority of clinical TNBC, and contains mutant RAS, which is rare in this disease.
2. In vivo, the effect appears to be modest even in the MDA231 model, selected for evidence of synergy in vitro. In vivo, the combination appears to have an additive effect. Tumor growth rates are reduced, but no shrinkage is occurring. In the PDX model, LOXL2i does not have an effect as a monotherapy, while modestly enhancing the impact of BRD4i. These results are at odds with the claim of the interaction being fundamental for proliferation.
3. No analysis of cell proliferation was shown in vivo. Authors should have performed BrdU or KI67 staining to support the claim. For in vitro analyses, authors also used indirect assays for proliferation. PI staining by itself does not have sufficient resolution to clearly capture modest effects that authors demonstrate. BrdU-PI double

staining would have been much more useful.

Minor points:

1. Dose dependent decrease in phosphorylated H3 is not at all obvious from eyeballing the data in S1A; the only effect that I see is a modest reduction at the highest concentration of the inhibitor. Authors need to quantify the results to support the claim.
2. Most of breast cancer cell lines are derived from metastatic disease, including pleural effusion, thus the point that because MDA231 cell line is derived from pleural effusion, it is metastatic does not have sufficient logical foundation.
3. How is loss of cell-cell junction in vitro consistent with LOXL2 role in modulating ECM? There is no evidence of ECM production in MDA231 in vitro. On the other hand, this loss is associated with EMT.

2. Significance:

Significance (Required)

Discovery and characterization of LOXL2-BRD4 interaction is advancing the ever-deepening understanding of molecular mechanisms of regulation of gene expression. The studies and analyses appear to be sufficiently rigorous and reported with clarity, and the claimed discovery of the biological interaction between LOXL2 and BRD4 is well supported. However, given the magnitude of the reported (rather than claimed) effects of this interaction, and concerns about generalizability of authors conclusions, it is not clear how these results are promising for the development of new therapies in TNBC. Moreover, in contrast to luminal BC, there is no clear evidence for utility of cytostatic drugs in constraining TNBC. Therefore, biological and clinical significance of the authors discovery is unclear and claims in this regard appear to be overblown.

3. How much time do you estimate the authors will need to complete the suggested revisions:

Estimated time to Complete Revisions (Required)

(Decision Recommendation)

Cannot tell / Not applicable

4. *Review Commons* values the work of reviewers and encourages them to get credit for their work. Select 'Yes' below to register your reviewing activity at Web of Science Reviewer Recognition Service (formerly Publons); note that the content of your review will not be visible on Web of Science.

Web of Science Reviewer Recognition

Yes

Review #2

1. Evidence, reproducibility and clarity:

Evidence, reproducibility and clarity (Required)

In their study, Pascual-Reguant et al. show that combined inhibition of BRD4 and LOXL2 can synergize to restrict triple-negative breast cancer (TNBC) proliferation. BRD4 and LOXL2 are transcription regulators that can read and write epigenetic information, respectively. The authors employ three distinct breast cancer cell lines and mouse models with cell line-derived xenografts, and they show that combined inhibition of BRD4 and LOXL2 can be superior to single BRD4/LOXL2 inhibition in these model systems. In an attempt to identify a connection between BRD4 and LOXL2, the authors find that the two proteins can bind to each other. The authors performed most of the experiments in the breast cancer cell line MDA-MB-231. To assess the impact of LOXL2-inhibition on transcription, the authors assessed changes of the transcriptome in MDA-MB-231 cells following LOXL2 knockdown. They found that genes related to cell differentiation and morphology were upregulated, while genes related to the cell cycle were downregulated. ChIP-seq data of BRD4 showed that BRD4 can bind to cell cycle gene promoters and that this binding was enhanced upon loss of LOXL2. The authors found that LOXL2 and BRD4 interacted with the transcriptional cell cycle regulators B-MYB, FOXM1, and LIN9, which are components of the MYB-MuvB-FOXM1 (MMB-FOXM1) complex that is known to promote the expression of late cell cycle genes with important functions during mitosis. The authors conclude that LOXL2/BRD4 interact with each other and with

the MMB-FOXM1 complex to drive the expression of cell cycle genes and cell proliferations. Vice versa, they conclude that inhibition of LOXL2/BRD4 reduces cell proliferation through inhibiting the expression of cell cycle genes.

Major:

- The data and methods are presented well. The experiments are adequately replicated and analyzed. However, except for the first section, all experiments were performed using only one cell line. It is important to validate key findings in at least a second cell line.
- There appears to be a misunderstanding of the concept of cell cycle-dependent gene regulation by the DREAM complex and its related factors. Early (G1/S) cell cycle genes contain E2F promoter motifs, while late (G2/M) cell cycle genes contain CHR promoter motifs. The DREAM complex can bind both, while RB-E2F and MuvB recognize only E2F and CHR motifs, respectively. B-MYB and FOXM1 bind to MuvB and regulate late cell cycle genes, but they do not bind to early cell cycle genes. Given this concept, the authors' rationale to connect BRD4/LOXL2 through MuvB/B-MYB/FOXM1 with E2F promoter sequences and early cell cycle genes and the subsequent conclusions must be corrected.
- I felt that the suggested functional connection between LOXL2/BRD4 and DREAM is not strongly supported by the authors' data. Figure S6E: A similarity score of <0.7 is poor support for a 'consensus E2F sequence' and indicates very limited specificity. Figure 4E: IP with BRD4 and LOXL2 is missing as important control. A chromatin-binding control is missing that does not bind to DREAM/LOXL2/BRD4. To test for binding to the actual DREAM complex, the authors should include E2F4 and p130 in their IPs and western blots, perhaps following LOXL2 inhibition/knockdown. Figure 3: The authors' ChIP-seq data indicate that only a fraction of DREAM targets is bound by BRD4. To provide more evidence that LOXL2/BRD4 may be directly involved in regulating DREAM targets, the authors should compare the differential regulation of BRD4-bound DREAM targets upon LOXL2 knockdown with DREAM targets which are not bound by BRD4. If LOXL2/BRD4 acted in a direct manner on those targets, one would expect that loss of LOXL2 affected their transcription more strongly than the other DREAM targets which are affected only indirectly. Such an analysis can be performed readily using the available data.
- The authors state that it is surprising to find that LOXL2 can promote target gene transcription because it is rather known as a transcriptional repressor. To this point, the authors should perform standard analyses using their RNA-seq and ChIP-seq data. Compare differential expression of genes that are bound by BRD4S/L/S+L and genes not bound by BRD4. Perform motif search and enrichment analyses for transcription factor and co-factor binding data (public ChIP-seq repositories). Such analyses may suggest what gene sets are up- and downregulated by LOXL2 through BRD4S/L and

what other factors could be involved in LOXL2-dependent up- and downregulation of gene transcription.

Minor:

- I felt that background information on the BRD4 isoforms was missing. The short and long isoforms of BRD4 should be introduced briefly.
- Given that BRD4 inhibition is known to activate p53 (e.g., PMID 23317504 and 33431824) and p21 (PMID 31265875), the authors should discuss the p53 status of their cell lines (largely mutant). In general, I felt that the authors could better cite and discuss the current literature on BRD4 and LOXL2.
- It was unclear to me why the authors did not actually test experimentally whether their predicted interaction models 2 or 4 are likely true (Figure 2E+G).
- The transcription of cell cycle genes depends on the cell cycle (i.e., reduced cell cycle entry correlates with reduced cell cycle gene expression). Given that the authors showed LOXL2 inhibition reduce MDA-MB-231 cell proliferation, they should note that reduced expression of cell cycle-related genes is expected upon LOXL2 knockdown.
- The authors specify in their discussion that their data show a function of LOXL2/BRD4 in the cell cycle interphase, while there were no experiments that support that specific conclusion. At least it is unclear to me why the authors rule out a function in mitosis?
- I felt that the first part of the manuscript (combination of BRD4 and LOXL2 inhibitors in TNBC) was a bit uncoupled from the functional studies on LOXL2 and its connection to BRD4. The transition between these parts and the final discussion on why the joint control of cell cycle genes by LOXL2/BRD4 may be important for the synergistic effect of LOXL2/BRD4 inhibitors. To this point, the authors' model was not clear to me.

2. Significance:

Significance (Required)

The study by Pascual-Reguant et al. shows that inhibitors of BRD4 and LOXL2 can be combined to achieve better efficacy in reducing proliferation of breast cancer cell lines and breast tumor growth in xenograft models. They provide strong evidence for a functional interaction between LOXL2 and BRD4 and investigate their common transcriptional targets. Intriguingly, some evidence points towards a direct regulation of the DREAM complex and its cell cycle gene targets.

The findings are novel and can be the basis for further research on TNBC combination

therapy using BRD4 and LOXL2 inhibitors. The link to the DREAM complex is preliminary.

The study is of interest for a basic research audience with some translational aspects.

I reviewed this manuscript as a researcher in gene regulatory mechanisms, with cell cycle genes as one focus area. I have no expertise in the computational modeling of protein-protein interactions and I am no expert for breast cancer.

3. How much time do you estimate the authors will need to complete the suggested revisions:

Estimated time to Complete Revisions (Required)

(Decision Recommendation)

Between 1 and 3 months

4. *Review Commons* values the work of reviewers and encourages them to get credit for their work. Select 'Yes' below to register your reviewing activity at Web of Science Reviewer Recognition Service (formerly Publons); note that the content of your review will not be visible on Web of Science.

Web of Science Reviewer Recognition

Yes

Review #3

1. Evidence, reproducibility and clarity:

Evidence, reproducibility and clarity (Required)

Summary:

In this manuscript, Laura Pascual-Reguant et al. identified a novel role of the LOXL2 oxidase in sustaining cell cycle progression through a so far uncharacterized gene-activating function is mediated by the BRD4S epigenetic reader and exerted on key DREAM-target genes in TNBC. Moreover, the authors showed that combinatorial treatment of TNBC with LOXL2- and BRD4-specific inhibitors result in a tremendous anti-tumorigenic effect. For all findings, they leveraged in vitro and in vivo settings as well as high-throughput sequencing approaches. However, the following points should be addressed and explained.

Major points:

- The authors on their working hypothesis propose that dual inhibition of BRD4 and LOXL2 is a novel strategy for curing TNBC. For my taste, just because both targets are quite promising for TNBC, the jump to this combinatorial treatment is kind of abrupt. Knowing the difficulty and time-/financial- investment, authors could optionally perform a mass spectrometry analysis on nuclei lysates with LOXL2 pull down to identify physical interactors. Due to the augmented resources and analysis of raw data, authors may necessitate a generous revision period (approx. 4 months for starters). By that, this can provide a more unbiased approached to look at nucleus-specific gene-regulatory functions and particularly at epigenetic readers. It would be also interesting to see if LOXL2 interacts with other members of the BRD family. Selecting BRD4 and no other members of the bromodomain family cannot be the only choice given that other BRD members can also interact with several of these mediator subunits.
- LOXL enzymes have been shown to promote collagen and fibronectin assembly, thereby sustaining the pro-survival effect of the ITG5A/FN1/FAK/SRC signaling cascade and shielding TNBC cells against chemotherapy treatment (32415208). Did authors observe if LOXL2 loss or inhibition decreased the active status of FAK and SRC, which are well known to promote G1-S transition (25381661)? Probably the cell cycle defects upon LOXL2 loss may also partially arise from the impairment of this cascade.
- Authors exclusively use JQ1 as a BRD4 inhibitor. As JQ1 may have an unspecific effect on BRD2 as well, authors should consider reproducing key experiments with siControl- and siBRD4-treated cells and increasing doses of PSX as well as repeating the JQ1 dose response assay in Figure 1B using siRNA-mediated silencing of LOXL2. Given that both players are part of the same complex, silencing of one and inhibition of the other should sensitize cells compared to their control counterparts.
- Moreover, in Figures 1G and S3D the differential sensitivity of low and high LOXL2 cell lines is unclear. Do authors know if any of these growth kinetic lines

represent one of the tested cell lines in Figure 1A-B? Authors should provide respective legends. In addition, authors should take advantage of their homemade data given that they have already selected a panel of TNBC cell lines with various LOXL2 expression at basal state (Figure 1A) for which dose response assays have been performed (Figure 1B). Therefore, I would perform an IC50 graph for JQ1 (without PSX treatment) using the existing data from Figure 1B.
- In Figure 2D, the pull-down assay is inconclusive, as the molecular weight for each construct is not mentioned. I would probably add this information also in all performed western blots. Also, the overexpression of the BD1/BD2-mutated and especially the BD1/BD2-lacking construct is unclear if it still interacts with LOXL2, probably because of the lack of molecular weight reference of each band. Therefore, the authors should make this pull-down assay more descriptive regarding the size of the bands. Also, BD1 mutagenesis at N140 was shown to dislodge the binding of JQ1 to BRD4 (24497639), which implies that BD1 mutagenesis or overexpression of the BD1-deficient construct should abrogate the interaction of LOXL2 with BRD4, reminiscent to the abrogated interaction of BRD4/LOXL2 upon JQ1 that binds to both BDs (Figure 2F). And, what happens if a BD2-deficient construct is expressed?
- If authors support that BRD4S is the predominant isoform driving the expression of DREAM-targets, this means that DREAM-targets are mainly bound by BRD4S, relying on Figure 3E-F. However, based on the author's ChIPseq tracks in Figure 3H, DREAM targets such as EZH2 and HMGB2 are co-occupied by both BRD4 isoforms at the basal state on their promoter region. Also, especially for EZH2 and PLK4, authors should set to 'group auto-scale' both conditions in a smaller scale range for ChIPseq- and RNAseq tracks, although I do not these two genes as good candidates representing your analysis. Therefore, authors should initially show all genes (e.g in a table format) that enrich the 'DREAM-targets' signature and select for a greater panel of genes (like for AURKB and HMGB2) demonstrating a preferential occupancy of the BRD4S at their promoter region. Finally, authors are recommended to perform a ChIP-qPCR on these genomic regions at basal state (no LOXL2 silencing) to validate the predominant occupancy of BRD4S and the low/absent occupancy of BRD4L at these genomic sites.
- Authors in Figure 3G should select an equal-sized population of randomly chosen non-DREAM-target genes, otherwise, the comparison of log2FC difference between these two gene cohorts is unreliable and difficult to make. Mann-Whitney test should also be performed.
- Authors should repeat the cell cycle analysis (Figure 4A) as the number of cells subjected to flow cytometry is quite discrepant between the conditions. Also, it is not clear if the experiment was performed in at least biological triplicates (although in the respective legend, it is stated so). If performed in biological triplicates, authors should make a new graph where each cell cycle phase cell population differs between the two conditions. Moreover, the difference in cell cycle defects in LOXL2-inhibited cells

(Figure 4C) is indifferent compared to their control counterpart. Therefore, authors should address these inconsistencies.
- Furthermore, authors should explain what was the rational selecting a mediator subunit and specifically MED1 as a possible interacting partner of LOXL2 and BRD4s since MED12 and MED24 were also highly essential (Figure 4F).
- Moreover, do authors also observe this functional relationship of LOXL2 and BRD4S in cell cycle progression in other breast cancer subtypes presenting a high proliferation index e.g HER2+? Presumably, the author's proposed mechanism applies to a wide panel of breast cancer entities, for which, only key experiments could be performed.
- Authors in Figure 5H represent LOXL2 and BRD4s as integral chromatin looping factors together with MED1 at promoter and enhancer regions. However, this illustration is an overrepresentation of their finding because authors did not address the differential occupancy of BRD4S upon LOXL2 loss in DREAM-target-specific enhancer regions. If they wish to do so, they may use the RANK ORDERING OF SUPER-ENHANCERS (ROSE) package to call for super-enhancer regions in the proximity of DREAM-targets and confirm similar results as for their TSS-proximal sites.
- In the current manuscript, authors did not address the translational relevance of their proposed mechanism in the context of conventional therapies. Knowing that several BRD-specific compounds currently undergo clinical trials, authors should address if LOXL2 low (MDAMB468) and high (BT549) cells demonstrate a differential sensitivity to increasing doses of chemotherapy, in the presence or absence of BRD4. By doing that, LOXL2 apart from being a therapeutic target could be also used as a prognostic marker to stratify patients and achieve better response to standard therapies.

Minor points:

- In Figure 1D, the authors should convert the y-axis to a logarithmic scale to better represent the differences between JQ1, PXS, and combo. Also, One-way Anova should be performed between JQ1, PXS and combo.
- In Figure S6F, authors did not show the sensitivity of LOXL2 low and high cell lines for BRD4 KO. If LOXL2-proficient cells are less sensitive to JQ1, based on Figure 1B, authors should consider showing something similar from the gene essentiality database.
- Authors failed to discuss the work from Ozge Saatci et al (PMID: 32415208) regarding LOXL2 in TNBC and ECM reorganization as well as in other cancer entities (PMID: 35428659) in the context of ECM remodeling. Authors should realize that these published works and the current ones are not conflicting but complement each other.

2. Significance:

Significance (Required)

Minor points:

- In Figure 1D, the authors should convert the y-axis to a logarithmic scale to better represent the differences between JQ1, PXS, and combo. Also, One-way Anova should be performed between JQ1, PXS, and combo.
- In Figure S6F, the authors did not show the sensitivity of LOXL2 low and high cell lines for BRD4 KO. If LOXL2-proficient cells are less sensitive to JQ1, based on Figure 1B, authors should consider showing something similar from the gene essentiality database.
- Authors failed to discuss the work from Ozge Saatci et al (PMID: 32415208) regarding LOXL2 in TNBC and ECM reorganization as well as in other cancer entities (PMID: 35428659) in the context of ECM remodeling. Authors should realize that these published works and the current ones are not conflicting but complement each other.

Significance

The conception and findings are of enlightening significance for TNBC therapy, especially given the lack of targeted therapies in this particularly aggressive breast cancer subtype. Hence, I posit this work as highly relevant for the cancer epigenetics research community interested in characterizing unknown factors that facilitate the gene-activating function of epigenetic readers in health and disease.

My field of expertise is to uncover epigenetic vulnerabilities responsible for transcriptional plasticity driving drug tolerance in aggressive forms of breast cancer.

3. How much time do you estimate the authors will need to complete the suggested revisions:

Estimated time to Complete Revisions (Required)

(Decision Recommendation)

Between 3 and 6 months

4. *Review Commons* values the work of reviewers and encourages them to get credit for their work. Select 'Yes' below to register your reviewing activity at Web of Science Reviewer Recognition Service (formerly Publons); note that the content of your review will not be visible on Web of Science.

Web of Science Reviewer Recognition

Yes

Manuscript number: RC-2022-01515
Point-by-point Response to Reviewers

Barcelona, 03/08/2023

We would like to take the opportunity to thank the reviewers for their overall positive evaluation of our manuscript and for the raised criticism. In the last 10 months, we revised our work in an attempt to answer their questions, which, we believe, made our manuscript more solid, consistent, and of high impact.

Reviewer #1 (Evidence, reproducibility and clarity (Required)):

The manuscript describes that simultaneous inhibition of LOXL2 and BRD4 reduces proliferation of TNBC in vitro and reduces growth in vivo.
This observation is followed by extensive mechanistic studies that suggest physical interaction between LOXL2 and short isoform of BRD4-MED1. Inferences from Chip-seq analyses suggest that this interaction is involved in regulation of multiple transcriptional programs. Authors focus on differential activation of DREAM complex, to claim that this interaction "is fundamental for proliferation of TNBC". The manuscript is very well written and mechanistic inferences are based on a set of sophisticated epigenetic analyses and bioinformatical inferences. The phenotypic effects from LoxL2 inhibition by itself, or in combination with BRD4 inhibition are relatively modest. These modest effects, as well as many of the reported changes in gene expression are clearly inconsistent with the frequently used adjectives as "dramatic", "fundamental", "deeply affected", "drastically hampered" etc. Given the modest phenotypic effects, many of the key claims and conclusions are not supported by the data.

We thank the reviewer for appreciating our work, defining the manuscript as well-written, and saying that it comprises extensive mechanistic studies as well as sophisticated epigenetic analysis.

We apologize if some of our statements seemed exaggerated. In this revised version, we revisited some of our conclusion to moderate them.

Moreover, we took the reviewer's criticism as an opportunity to strengthen our findings. In the revised version of the manuscript, we included an additional TNBC PDX (PDX-127), and results from this experiment clearly reinforce our claims (Fig. 6D and Fig. EV9E-F). In this new in vivo experiment, we selected a PDX model in which the expression of BRD4L is not detectable, while BRD4S is clearly expressed. Therefore, the treatment with JQ1 would specifically affect the activity of BRD4S, making the treatment selective. Additionally, we reduced by half the dose of JQ1 administrated to limit the effect of BRD4S inhibition alone on tumor growth. The combinatorial treatment (JQ1+PXS) induced a clear superior effect in this setting as compared with single-agent treatments. In addition to this, we discarded that the observed growth reduction is not the result of the sole inhibition of LOXL2, which could affect FAK/Src activity or extracellular Collagen crosslinking. In conclusion, our data show that the combinatorial inhibition of LOXL2 and BRD4S is effective in reducing tumor proliferation in TNBC in vivo models, independently of the inhibition of BRD4S and of other pathways known to be regulated by LOXL2.

Specifically:

1) It is unclear why authors generalize their conclusions to TNBC. Figure 1B demonstrates synergy for 1/3 cell lines, which is chosen for the follow up study. Even for MDA231, the synergy is confined to low concentrations of BRD4i (S1c). While MDA231 cell line is frequently used in experimental studies of TNBC, it is quite dissimilar to majority of clinical TNBC, and contains mutant RAS, which is rare in this disease.

The synergistic effect is observed in MDA-MB-231 cells because only this cell line expresses both BRD4S and LOXL2. Indeed, in Fig. 1C we show that MDA-MB-468 cells do not express LOXL2, while BT549 only express minimal BRD4 levels.

To corroborate this hypothesis, in the revised version of the manuscript we added:

a. *A new cell line (Cal51) expressing the same LOXL2 and BRD4 levels (Fig. EV8C) but showing greater resistance to JQ1 than MDA-MB-231 (Fig. EV8D). Also, in this cell line, we could show that the combinatorial treatment had a superior effect on cell viability than the single agents' treatment (Fig. EV8E).*

b. *A western blot panel of different TNBC PDXs shows that the majority of them express medium to high levels of both BRD4S and LOXL2 proteins, as is the case of MDA-MB-231 (Fig. EV9E) and Cal51 (Fig. EV8C). This result suggests that the combinatorial treatment could be used in the majority of TNBC patients as they are expected to express both BRD4S and LOXL2.*
c. *Finally, as explained above, we performed another in vivo choosing a PDX that expresses BRD4S (but not BRD4L) and LOXL2 (PDX-127) (Fig. 6D and Fig. EV9E-F). Also, in this new model, we could observe that the combinatorial inhibition had a superior effect than single treatments.*

2) In vivo, the effect appears to be modest even in the MDA231 model, selected for evidence of synergy in vitro. In vivo, the combination appears to have an additive effect. Tumor growth rates are reduced, but no shrinkage is occurring. In the PDX model, LOXL2i does not have an effect as a monotherapy, while modestly enhancing the impact of BRD4i. These results are at odds with the claim of the interaction being fundamental for proliferation.

We agree with the reviewer that the combinatorial inhibition appears to have an additive effect in vivo using the MDA-MB-231 model.

a. *For that reason, we have now performed the in vivo PDX experiment mentioned above (PDX-127; Fig. 6D and Fig. EV9E-F) in which we decreased the dose of JQ1 by half to avoid strong tumor growth effect due to BRD4 inhibition alone. In this new experiment, the synergistic effect is evident. While single-agent treatment showed a very moderate effect (0% or 20% tumor growth reduction for LOXL2 and JQ1, respectively), the combinatorial treatment showed a 50% reduction in tumor volume, further supporting our conclusions.*
b. *We also performed either BRD4 or MED1 pull-down experiments in the presence of PXS and JQ1. We show that upon PXS treatment, the interaction between LOXL2 and BRD4S is maintained while the interaction with MED1 is reduced (Fig. 5A-C). However, in the presence of JQ1, the interaction between LOXL2 and MED1 is maintained while BRD4S-LOXL2 and BRD4S-MED1 interactions are impaired (Fig. 5D-F). These new results explain why monotherapy does not have a sufficient effect in vivo and set the rationale for the use of the combinatorial treatment.*

We believe that these new results corroborate our initial findings and we hope to have been able to satisfy the reviewer comments.

3) No analysis of cell proliferation was shown in vivo. Authors should have performed BrdU or KI67 staining to support the claim. For in vitro analyses, authors also used indirect assays for

proliferation. PI staining by itself does not have sufficient resolution to clearly capture modest effects that authors demonstrate. BrdU-PI double staining would have been much more useful.

We appreciate the reviewer's comment. In the revised manuscript we have added Ki67 and H3S10p staining in the tumor samples for the new in vivo PDX experiment (Fig. 6E and Fig. EV10A-C). We show that the combinatorial treatment significantly induces a reduction of both proliferation markers, which is in agreement with a reduced tumor volume. Regarding the in vitro analysis, we did not only use PI staining to show a reduced proliferation state but also H3S10p staining (Fig. 4B) and an SLBP1 fluorescent reporter MDA-MB-231 cell line (Fig. 4D, Fig. EV6B, E, and Movie EV). In the revised version of the manuscript, we included a new FACS-PI analysis (Fig. 4A, C) to better represent the effects we see on the cell cycle.

Minor points:

1. Dose dependent decrease in phosphorylated H3 is not at all obvious from eyeballing the data in S1A; the only effect that I see is a modest reduction at the highest concentration of the inhibitor. Authors need to quantify the results to support the claim.

We agree with the reviewer and we apologize for the misinterpretation. We have changed the revised manuscript as follows: "The selective LOXL2 inhibitor PXS-538224 (hereafter, PXS) efficiently reduced the levels of oxidized histone H3 (H3K4ox) in MDA-MB-231 cells at 40 μM (Fig. EV6C), indicating an efficient inhibition of LOXL2 catalytic activity in the nucleus."

2. Most of breast cancer cell lines are derived from metastatic disease, including pleural effusion, thus the point that because MDA231 cell line is derived from pleural effusion, it is metastatic does not have sufficient logical foundation.

Many publications have shown the high metastatic capacity of MDA-MB-231 (e.g. https://doi.org/10.1016/j.bbabio.2011.04.015, doi: 10.1038/s41467-017-01829-1), which are therefore used as TNBC metastatic model. The scope of the analysis reported in Fig. 6C was just to show whether any of the used treatments could reduce the metastatic capacity of this cell line. We believe we do not overstate the results but just report them as they are.

3. How is loss of cell-cell junction in vitro consistent with LOXL2 role in modulating ECM? There is no evidence of ECM production in MDA231 in vitro. On the other hand, this loss is associated with EMT.

We thank the reviewer for identifying this mistake. In the revised manuscript we changed the text as follows: "Gene set enrichment analysis (GSEA) revealed that LOXL2 KD induced upregulation of processes involved in cell morphology, secretion, membrane trafficking, and cell differentiation, with cell-cell junction being one of the most significantly affected pathways (Fig. EV5E). These results agree with the role of LOXL2 in regulating epithelial-to-mesenchymal transition, corroborating the high quality of our dataset."

Reviewer #1 (Significance (Required)):

Discovery and characterization of LOXL2-BRD4 interaction is advancing the ever-deepening understanding of molecular mechanisms of regulation of gene expression. The studies and analyses appear to be sufficiently rigorous and reported with clarity, and the claimed discovery of the biological interaction between LOXL2 and BRD4 is well supported. However, given the magnitude of the reported (rather than claimed) effects of this interaction, and concerns about generalizability of authors conclusions, it is not clear how these results are promising for the development of new therapies in TNBC. Moreover, in contrast to luminal BC, there is no clear evidence for utility of cytostatic drugs in constraining TNBC. Therefore, biological and clinical significance of the authors discovery is unclear and claims in this regard appear to be overblown

We thank the reviewer for stating that our analysis is rigorous and reported with clarity. We really took the criticisms as an opportunity to strengthen our findings, as explained above.
For the newly presented in vivo PDX model, we performed immunohistochemistry of Ki67, H3S10p and Cleaved Caspase 3 to check whether the reduction of tumor volume observed in the combinatorial treatment was a result of a cytotoxic and/or a cytostatic effect (Fig. 6E and Fig. EV10A-C). As shown in the figure, the combination of the two inhibitors induced a superior decrease of Ki67, H3S10p, and a clear increase of Cleaved Caspase 3. Therefore, these new data indicate that the combinatorial treatment does not only have a cytostatic effect but also cytotoxic, suggesting a clinical exploitability for the treatment of TNBC patients.

Reviewer #2 (Evidence, reproducibility and clarity (Required)):

In their study, Pascual-Reguant et al. show that combined inhibition of BRD4 and LOXL2 can synergize to restrict triple-negative breast cancer (TNBC) proliferation. BRD4 and LOXL2 are

transcription regulators that can read and write epigenetic information, respectively. The authors employ three distinct breast cancer cell lines and mouse models with cell line-derived xenografts, and they show that combined inhibition of BRD4 and LOXL2 can be superior to single BRD4/LOXL2 inhibition in these model systems. In an attempt to identify a connection between BRD4 and LOXL2, the authors find that the two proteins can bind to each other. The authors performed most of the experiments in the breast cancer cell line MDA-MB-231. To assess the impact of LOXL2-inhibition on transcription, the authors assessed changes of the transcriptome in MDA-MB-231 cells following LOXL2 knockdown. They found that genes related to cell differentiation and morphology were upregulated, while genes related to the cell cycle were downregulated. ChIP-seq data of BRD4 showed that BRD4 can bind to cell cycle gene promoters and that this binding was enhanced upon loss of LOXL2. The authors found that LOXL2 and BRD4 interacted with the transcriptional cell cycle regulators B-MYB, FOXM1, and LIN9, which are components of the MYB-MuvB-FOXM1 (MMB-FOXM1) complex that is known to promote the expression of late cell cycle genes with important functions during mitosis. The authors conclude that LOXL2/BRD4 interact with each other and with the MMB-FOXM1 complex to drive the expression of cell cycle genes and cell proliferations. Vice versa, they conclude that inhibition of LOXL2/BRD4 reduces cell proliferation through inhibiting the expression of cell cycle genes.

Major:

- The data and methods are presented well. The experiments are adequately replicated and analyzed. However, except for the first section, all experiments were performed using only one cell line. It is important to validate key findings in at least a second cell line.

We thank the reviewer for valuing our work.
To address the reviewer's comment, in the revised manuscript we added an additional cell line (Cal-51), that expresses similar levels of LOXL2 and BRD4 as compared to MDA-MB-231 (Fig. EV8C). Even though this cell line is clearly more resistant to JQ1 than the MDA-MB-231 cell line (Fig. EV8D), the combinatorial treatment is significantly more effective as compared with single agents' treatment (Fig. EV8E).
Moreover, we have also performed an additional in vivo experiment using another TNBC PDX (PDX-127) that expresses LOXL2 and BRD4S, but not BRD4L. Given that JQ1 can inhibit both BRD4 isoforms, this in vivo system allowed us to demonstrate that the tumor antiproliferative capacity of the combinatorial treatment is due to the simultaneous inhibition of LOXL2 and BRD4S (rather than BRD4S and L) (Fig. 6D and Fig. EV9E-F).

- There appears to be a misunderstanding of the concept of cell cycle-dependent gene regulation by the DREAM complex and its related factors. Early (G1/S) cell cycle genes contain E2F promoter motifs, while late (G2/M) cell cycle genes contain CHR promoter motifs. The DREAM complex can bind both, while RB-E2F and MuvB recognize only E2F and CHR motifs, respectively. B-MYB and FOXM1 bind to MuvB and regulate late cell cycle genes, but they do not bind to early cell cycle genes. Given this concept, the authors' rationale to connect BRD4/LOXL2 through MuvB/B-MYB/FOXM1 with E2F promoter sequences and early cell cycle genes and the subsequent conclusions must be corrected.

We thank the reviewer for their expert explanation. We corrected our conclusion in the revised version of the manuscript following the reviewer's comment.

- I felt that the suggested functional connection between LOXL2/BRD4 and DREAM is not strongly supported by the authors' data. Figure S6E: A similarity score of <0.7 is poor support for a 'consensus E2F sequence' and indicates very limited specificity. Figure 4E: IP with BRD4 and LOXL2 is missing as important control. A chromatin-binding control is missing that does not bind to DREAM/LOXL2/BRD4. To test for binding to the actual DREAM complex, the authors should include E2F4 and p130 in their IPs and western blots, perhaps following LOXL2 inhibition/knockdown. Figure 3: The authors' ChIP-seq data indicate that only a fraction of DREAM targets is bound by BRD4. To provide more evidence that LOXL2/BRD4 may be directly involved in regulating DREAM targets, the authors should compare the differential regulation of BRD4-bound DREAM targets upon LOXL2 knockdown with DREAM targets which are not bound by BRD4. If LOXL2/BRD4 acted in a direct manner on those targets, one would expect that loss of LOXL2 affected their transcription more strongly than the other DREAM targets which are affected only indirectly. Such an analysis can be performed readily using the available data

Fig. EV6E: We agree with the reviewer that a similarity score of <0.7 does not support specificity. In the revised manuscript, we opted for removing the motif analysis and instead showing a comparative analysis between Ab2-unique (BRD4S) and Ab1-unique (BRD4L) -bound DREAM target gene promoters. This analysis showed that the proportion of early DREAM target genes - identified by Fischer and colleagues (doi: 10.1093/nar/gkw523)- was higher in Ab2-exclusive as compared to all annotated DREAM target genes (Fig. EV6F). These data support our hypothesis of BRD4S regulating predominantly the expression of early cell cycle genes.

Fig. 4E: We thank the reviewer for this comment. The performed pulldown showed that BRD4S, LOXL2, and MED1 interact with Lin9 and B-Myb, but not with FOXM1, thus FOXM1 itself is an internal negative control of the pulldown. Additionally, BRD4L does not show the same interaction pattern as BRD4S, LOXL2, and MED1, again acting as an internal negative control. We, therefore, believe that the pulldown is properly controlled and that the observed interaction is trustful. We furthermore agree with the reviewer that it would be interesting to characterize the interactions between the DREAM complex and BRD4S, LOXL2, and MED1. However, we believe that the dissection of these interactions at the mechanistic levels would require a deeper study, which can be a project in itself that we aim to explore in the future. For example, it would be interesting to investigate whether either the inhibition or the downregulation of LOXL2 and/or BRD4S specifically impairs the formation of the DREAM complex or the recruitment of specific DREAM complex subunits, as well as how these effects impair the DREAM complex chromatin binding. We are afraid that the suggested pulldowns would not be sufficient to answer these questions, which would require extensive cross-interaction studies in either BRD4/LOXL2 and BRD4+LOXL2 inhibition or downregulation followed by ChIP-seq and transcriptomics for all the conditions. We believe that the provided data, together with the functional characterization (both, in vitro and in vivo), of the phenotypes triggered by BRD4S and LOXL2 inhibition make a strong case for our manuscript and leave out of scope the suggested experiments. We hope the reviewer will understand our explanation and will appreciate that we are planning to pursue this further in the future.

Fig. 3: We thank the reviewer for this important comment. The ChIP-seq technique very often does not provide exhaustive results due to sequencing depth limits and antibody performance. We believe that the fraction of DREAM target genes found in our dataset as bound by BRD4S is not exhaustive and that the analysis proposed by the reviewer would not lead to clear conclusive results. However, we understand the importance of verifying that DREAM target genes whose promoter is bound by BRD4 are indeed downregulated when LOXL2 is inhibited. To give an answer to this question, in the revised manuscript we added gene expression analysis of selected DREAM target genes upon treatment with JQ1, PXS their combination. We could successfully show that both JQ1 and PXS treatment impairs the transcription of the selected DREAM target genes, however, the combinatorial treatment almost shut down their expression, in agreement with our hypothesis (Fig. 5J).

- The authors state that it is surprising to find that LOXL2 can promote target gene transcription because it is rather known as a transcriptional repressor. To this point, the authors should perform standard analyses using their RNA-seq and ChIP-seq data. Compare differential

expression of genes that are bound by BRD4S/L/S+L and genes not bound by BRD4. Perform motif search and enrichment analyses for transcription factor and co-factor binding data (public ChIP-seq repositories). Such analyses may suggest what gene sets are up- and downregulated by LOXL2 through BRD4S/L and what other factors could be involved in LOXL2-dependent up- and downregulation of gene transcription.

We thank the reviewer for this valuable comment that certainly provides the rationale for a follow-up project. However, we believe that the proposed study goes beyond the scope of our work at this moment.

Minor:

- I felt that background information on the BRD4 isoforms was missing. The short and long isoforms of BRD4 should be introduced briefly.

We agree with the reviewer. In the revised manuscript, we addressed this by presenting BRD4 isoforms in the introduction part of the manuscript.

- Given that BRD4 inhibition is known to activate p53 (e.g., PMID 23317504 and 33431824) and p21 (PMID 31265875), the authors should discuss the p53 status of their cell lines (largely mutant). In general, I felt that the authors could better cite and discuss the current literature on BRD4 and LOXL2.

We appreciate the comment of the reviewer regarding p53. Given the fact that p53 is mutant in MDA-MB-231, we believe that the proliferation defect observed with the combinatorial treatment may be due to the activation of alternative cytostatic or cytotoxic signaling cascades, independently of P53 activation. We have now briefly mentioned this point in the manuscript discussion.

- It was unclear to me why the authors did not actually test experimentally whether their predicted interaction models 2 or 4 are likely true (Figure 2E+G).

We understand the reviewer's comment. The fact that JQ1 treatment almost abrogates the interaction between LOXL2 and BRD4S strongly suggests that models 1 and 3 are likely wrong, therefore pointing towards models 2 and 4 as the correct ones. To test whether models 2 and 4 are indeed the correct models we are now performing extensive mutagenesis studies, which are producing preliminary results suggesting indeed that models 2 and 4 are correct. The reason why

we did not include this study in the current manuscript, is that we started a parallel line of investigation aimed at identifying residues fundamental for the interaction that can be exploited in compound screening campaigns to identify molecules able to block the described interaction and thus cancer proliferation. Publishing these preliminary results at this stage could jeopardize the drug discovery campaign and we hope that the reviewer will understand our constraints.

- The transcription of cell cycle genes depends on the cell cycle (i.e., reduced cell cycle entry correlates with reduced cell cycle gene expression). Given that the authors showed LOXL2 inhibition reduce MDA-MB-231 cell proliferation, they should note that reduced expression of cell cycle-related genes is expected upon LOXL2 knockdown.

We understand the reviewer's comment. We believe that we provide sufficient data supporting our hypothesis that LOXL2 controls the expression of cell cycle genes at the transcriptional level together with BRD4S. In addition, the sole inhibition of LOXL2 has practically no effect on tumor proliferation in vivo but largely enhances the antiproliferative effect of low-dose JQ1 (Fig. 6D). We hope these clarifications would satisfy the reviewer.

- The authors specify in their discussion that their data show a function of LOXL2/BRD4 in the cell cycle interphase, while there were no experiments that support that specific conclusion. At least it is unclear to me why the authors rule out a function in mitosis?

We thank the reviewer for this comment. We referred to interphase genes because these are the early cell cycle genes, while mitotic genes are the late ones. We do not discard a possible function for BRD4S and LOX2 regulating mitotic progression, however, we believe this would be a consequence of dysregulated G1-S-G2 gene expression, rather than a direct transcriptional effect. This conclusion derives from the fact that while we observe interactions between LOXL2, BRD4S, and MED1 with Lin9 and B-Myb, these are not fully conserved with FOXM1, which is typically required for the transcription of mitotic genes. To avoid confusion, we have now anyway removed the word "interphase" from the text.

- I felt that the first part of the manuscript (combination of BRD4 and LOXL2 inhibitors in TNBC) was a bit uncoupled from the functional studies on LOXL2 and its connection to BRD4. The transition between these parts and the final discussion on why the joint control of cell cycle genes by LOXL2/BRD4 may be important for the synergistic effect of LOXL2/BRD4 inhibitors. To this point, the authors' model was not clear to me.

We really appreciate the reviewer's comment. To better connect the functional studies with the clinical significance of the proposed combinatorial treatment, we restructured the manuscript. In the revised version, the use of the combinatorial treatment is shown in Figure 6. Moreover, to better explain why we focused all the studies on BRD4 and LOXL2, we also included data from the Cancer Cell Line Encyclopedia (CCLE)-associated chemotherapeutics sensitivity (Fig. 1A and Fig. EV1) showing that LOXL2 expression levels can predict the response to BRD4 inhibition, suggesting a functional interaction between BRD4 and LOXL2 and the possibility to exploit it for therapeutical purposes. We believe that these data set the rationale to further explore the connection between LOXL2 and BRD4, both at the mechanistic and functional levels.

Reviewer #2 (Significance (Required)):

The study by Pascual-Reguant et al. shows that inhibitors of BRD4 and LOXL2 can be combined to achieve better efficacy in reducing proliferation of breast cancer cell lines and breast tumor growth in xenograft models. They provide strong evidence for a functional interaction between LOXL2 and BRD4 and investigate their common transcriptional targets. Intriguingly, some evidence points towards a direct regulation of the DREAM complex and its cell cycle gene targets.

The findings are novel and can be the basis for further research on TNBC combination therapy using BRD4 and LOXL2 inhibitors. The link to the DREAM complex is preliminary.

The study is of interest for a basic research audience with some translational aspects.

I reviewed this manuscript as a researcher in gene regulatory mechanisms, with cell cycle genes as one focus area. I have no expertise in the computational modeling of protein-protein interactions and I am no expert for breast cancer.

We thank the reviewer for the positive comments. We also would really like to thank the reviewer for their criticism, which, we believe, contributed to a new and improved manuscript version.

Reviewer #3 (Evidence, reproducibility and clarity (Required)):

Summary:

In this manuscript, Laura Pascual-Reguant et al. identified a novel role of the LOXL2 oxidase in

sustaining cell cycle progression through a so far uncharacterized gene-activating function is mediated by the BRD4S epigenetic reader and exerted on key DREAM-target genes in TNBC. Moreover, the authors showed that combinatorial treatment of TNBC with LOXL2- and BRD4-specific inhibitors result in a tremendous anti-tumorigenic effect. For all findings, they leveraged in vitro and in vivo settings as well as high-throughput sequencing approaches. However, the following points should be addressed and explained.

Major points:

-The authors on their working hypothesis propose that dual inhibition of BRD4 and LOXL2 is a novel strategy for curing TNBC. For my taste, just because both targets are quite promising for TNBC, the jump to this combinatorial treatment is kind of abrupt. Knowing the difficulty and time-/financial- investment, authors could optionally perform a mass spectrometry analysis on nuclei lysates with LOXL2 pull down to identify physical interactors. Due to the augmented resources and analysis of raw data, authors may necessitate a generous revision period (approx. 4 months for starters). By that, this can provide a more unbiased approached to look at nucleus-specific gene-regulatory functions and particularly at epigenetic readers. It would be also interesting to see if LOXL2 interacts with other members of the BRD family. Selecting BRD4 and no other members of the bromodomain family cannot be the only choice given that other BRD members can also interact with several of these mediator subunits.

We thank the reviewer for the suggestion and we agree with the fact that the rationale for combining BRD4 and LOXL2 inhibitors was not sufficiently argued in the first version of the manuscript. For that reason, in the revised manuscript, we added new data to explain why we explored this topic. In particular, to better explain why we focused all the studies on BRD4 and LOXL2, we included data from the Cancer Cell Line Encyclopedia (CCLE)-associated chemotherapeutics sensitivity (Fig. 1A and Fig. EV1) showing that LOXL2 expression levels can predict the response to BRD4 inhibition (but not to other approved chemotherapeutic drug), suggesting a functional interaction between BRD4 and LOXL2 and the possibility to exploit it for therapeutical purposes. Moreover, we restructured the manuscript to make the story more linear, explaining first the functionality of BRD4S-LOXL2 interaction at the molecular and cellular levels, and then presenting the in vivo systems in the last part of the manuscript.

We agree with the reviewer that it may be interesting to explore whether LOXL2 interacts with other BRD family members. However, given the prominent role of BRD4 in promoting cancer proliferation, we believe that understanding the relevance of BRD4S-LOXL2 interaction in TNBC

is, per se, of great interest and provide a novel mechanistic understanding of how TNBC proliferation is controlled at the transcription level. In the specific case of TNBC, it has been shown that BRD4S has an oncogenic effect, while BRD4L is an oncosuppressor. In the manuscript, we now showed that LOXL2 downregulation sensitizes cells to JQ1 treatment (Fig. 1D). Additionally, while the downregulation of BRD4L does not have any additional effect on cell treated with PXS, the downregulation of BRD4S sensitize them to LOXL2 inhibition (Fig. EV8B). These results, once again, indicate the relevance of studying the functional interaction between BRD4S and LOXL2.

-LOXL enzymes have been shown to promote collagen and fibronectin assembly, thereby sustaining the pro-survival effect of the ITG5A/FN1/FAK/SRC signaling cascade and shielding TNBC cells against chemotherapy treatment (32415208). Did authors observe if LOXL2 loss or inhibition decreased the active status of FAK and SRC, which are well known to promote G1-S transition (25381661)?
Probably the cell cycle defects upon LOXL2 loss may also partially arise from the impairment of this cascade.

We really appreciate the reviewer's suggestions. In the revised version of the manuscript, we checked FAK and Src activation status in tumor samples from one of our in vivo experiments (Fig. EV10D). We did not observe any difference in phospho-FAK or phospho-Src upon treatment either with PXS, JQ1, or their combinations, suggesting that alterations in the activity of these factors were not driving the observed proliferation defects.

-Authors exclusively use JQ1 as a BRD4 inhibitor. As JQ1 may have an unspecific effect on BRD2 as well, authors should consider reproducing key experiments with siControl- and siBRD4-treated cells and increasing doses of PSX as well as repeating the JQ1 dose response assay in Figure 1B using siRNA-mediated silencing of LOXL2. Given that both players are part of the same complex, silencing of one and inhibition of the other should sensitize cells compared to their control counterparts.

We agree with the reviewer and we addressed this comment in the revised manuscript. In particular, we have added two additional experiments:

- *We transduced MDA-MB-231 cells with isoform-specific shBRD4s (shBRD4L and shBRD4S) (Fig. EV5H) and checked cell sensitivity to PXS treatment (Fig. EV8B). As explained also above, we observed that only when the short isoform of BRD4 was downregulated cells displayed higher sensitivity to PXS treatment. This result corroborates that BRD4S and LOXL2 are required for TNBC proliferation.*

- *We transduced MDA-MB-231 cells with shLOXL2 and assessed JQ1 sensitivity (Fig. 1D). We showed that upon LOXL2 downregulation, cells became more sensitive to JQ1 treatment, again corroborating the fact that TNBC proliferation requires BRD4S and LOXL2.*

-Moreover, in Figures 1G and S3D the differential sensitivity of low and high LOXL2 cell lines is unclear. Do authors know if any of these growth kinetic lines represent one of the tested cell lines in Figure 1A-B? Authors should provide respective legends. In addition, authors should take advantage of their homemade data given that they have already selected a panel of TNBC cell lines with various LOXL2 expression at basal state (Figure 1A) for which dose response assays have been performed (Figure 1B). Therefore, I would perform an IC50 graph for JQ1 (without PSX treatment) using the existing data from Figure 1B.

We apologize if our representation was confusing. In the revised manuscript we have changed the sensitivity plots (Fig. 1A and Fig. EV1) to make them easier to grasp. Additionally, in Figure 1A we included the analysis of CCLE cell lines stratified based on their LOXL2 expression levels. This analysis showed that LOXL2 expression levels could overall predict the response to BETi treatment. As suggested by the reviewer, we also plotted the IC50 of the 3 cell lines tested. However, their JQ1 sensitivity curves did not show any difference that could be attributed to their different LOXL2 levels (figure below). Our speculation is that only 3 cell lines do not provide a sufficient size to reach a meaningful conclusion, which, in contrast, can be achieved by comparing the CCLE BETi sensitivity.

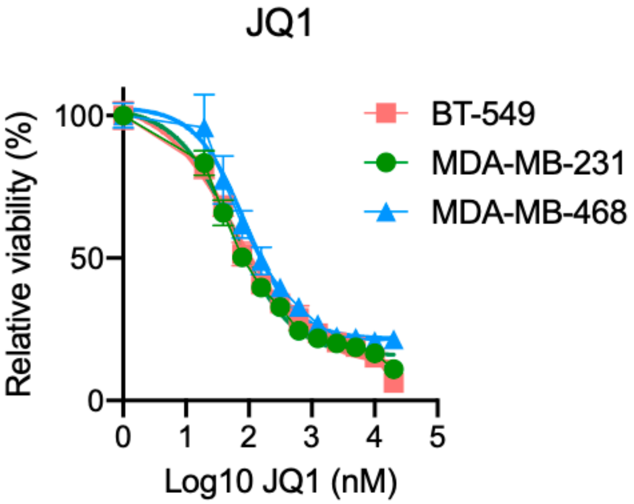

-In Figure 2D, the pull-down assay is inconclusive, as the molecular weight for each construct is not mentioned. I would probably add this information also in all performed western blots. Also,

the overexpression of the BD1/BD2-mutated and especially the BD1/BD2-lacking construct is unclear if it still interacts with LOXL2, probably because of the lack of molecular weight reference of each band. Therefore, the authors should make this pull-down assay more descriptive regarding the size of the bands. Also, BD1 mutagenesis at N140 was shown to dislodge the binding of JQ1 to BRD4 (24497639), which implies that BD1 mutagenesis or overexpression of the BD1-deficient construct should abrogate the interaction of LOXL2 with BRD4, reminiscent to the abrogated interaction of BRD4/LOXL2 upon JQ1 that binds to both BDs (Figure 2F). And, what happens if a BD2-deficient construct is expressed?

We thank the reviewer for spotting this distraction. We apologize for this and in the revised version of the manuscript we included molecular weights for all western blots.

We acknowledge that BD1 mutagenesis displaces JQ1 binding, however, we respectfully disagree that because of this BD1-N140 mutant should not bind to LOXL2. Our docking analysis indeed showed that none of the poses is impaired either by BD1 or BD2 mutagenesis (Fig. EV4D). The fact that JQ1 disrupts the interaction between BRD4S and LOXL2 (Fig. 2F, G) is not due to the fact that they compete for the same binding residue, but rather for the space occupied by JQ1 inside the AcK binding pocket of either BD1 or BD2, which impedes proper binding to LOXL2. Our pulldown data indeed showed that mutant BD1 and BD2 retain the ability to bind to LOXL2 (Fig. 2C), as predicted by the docking.

We did not try to express constructs either lacking BD1 or BD2 and we cannot speculate what could happen to the BRD4S-LOXL2 interaction in this scenario. Even though this experiment could help dissect the interaction between LOXL2 and BRD4S, we decided to rather perform mutagenesis of specific residues that have been predicted to be important for the interaction. The reason why we did not include this study in the current manuscript, is that we started a parallel line of investigation aimed at identifying residues fundamental for the interaction that can be exploited in compound screening campaigns to identify molecules able to block the described interaction and thus cancer proliferation. Publishing these preliminary results at this stage could jeopardize the drug discovery campaign and we hope that the reviewer will understand our constraints.

-If authors support that BRD4S is the predominant isoform driving the expression of DREAM-targets, this means that DREAM-targets are mainly bound by BRD4S, relying on Figure 3E-F. However, based on the author's ChIPseq tracks in Figure 3H, DREAM targets such as EZH2 and HMGB2 are co-occupied by both BRD4 isoforms at the basal state on their promoter region. Also,

especially for EZH2 and PLK4, authors should set to 'group auto-scale' both conditions in a smaller scale range for ChIPseq- and RNAseq tracks, although I do not these two genes as good candidates representing your analysis. Therefore, authors should initially show all genes (e.g in a table format) that enrich the 'DREAM-targets' signature and select for a greater panel of genes (like for AURKB and HMGB2) demonstrating a preferential occupancy of the BRD4S at their promoter region. Finally, authors are recommended to perform a ChIP-qPCR on these genomic regions at basal state (no LOXL2 silencing) to validate the predominant occupancy of BRD4S and the low/absent occupancy of BRD4L at these genomic sites.

We apologize for the confusion. To make the figure more understandable, we now scaled all the panels to the same scale and highlighted in grey the promoter region of each selected DREAM target gene. As the reviewer can appreciate, none of these genes is bound by BRD4L in basal conditions (Fig. 3F).
To better characterize the differential binding, following the reviewer's suggestion, we performed ChIP-qPCR using Ab2 (which recognizes both BRD4 isoforms), in cells either downregulated for BRD4L or BRD4S with isoform-specific shRNAs (Fig. EV5H). Results showed that only the downregulation of BRD4S reduced the binding of Ab2 to the promoter of the selected DREAM target genes (Fig. 3D), corroborating our hypothesis and validating our ChIPseq strategy.

-Authors in Figure 3G should select an equal-sized population of randomly chosen non-DREAM-target genes, otherwise, the comparison of log2FC difference between these two gene cohorts is unreliable and difficult to make. Mann-Whitney test should also be performed.

We thank the reviewer for this suggestion, which was added to the revised version of the manuscript (Fig. 3E, lower panel).

-Authors should repeat the cell cycle analysis (Figure 4A) as the number of cells subjected to flow cytometry is quite discrepant between the conditions. Also, it is not clear if the experiment was performed in at least biological triplicates (although in the respective legend, it is stated so). If performed in biological triplicates, authors should make a new graph where each cell cycle phase cell population differs between the two conditions. Moreover, the difference in cell cycle defects in LOXL2-inhibited cells (Figure 4C) is indifferent compared to their control counterpart. Therefore, authors should address these inconsistencies.

We thank the reviewer for the suggestion. In the revised version of the manuscript, we represent the cell cycle also as a bar plot with statistical analysis (Fig. 4A, C). Even though the number of

cells was the same across conditions, the sub-G1 population of the LOXL2 KD cells may have distorted the profile of the cell cycle. To avoid misinterpretations, we repeated the analysis in the revised version of the manuscript. Statistical analysis supports that LOXL2 inhibition or downregulation has a significant effect on cell cycle progression (Fig. 4A, C, right panel).

-Furthermore, authors should explain what was the rational selecting a mediator subunit and specifically MED1 as a possible interacting partner of LOXL2 and BRD4s since MED12 and MED24 were also highly essential (Figure 4F).

We selected MED1 as a Mediator Complex proxy. In our essentiality analysis MED 1, 9, 10, 12, 15, 16, 19, 23, 24, 25 score as significant, suggesting a functional interaction between LOXL2 and the Mediator Complex, rather than a specific subunit. MED1 has been previously described as a BRD4 partner and it is often used in immunofluorescence to visualize transcriptional foci, which made it the best candidate for follow-up study in our project.

-Moreover, do authors also observe this functional relationship of LOXL2 and BRD4S in cell cycle progression in other breast cancer subtypes presenting a high proliferation index e.g HER2+? Presumably, the author's proposed mechanism applies to a wide panel of breast cancer entities, for which, only key experiments could be performed.

We thank the reviewer for the suggestion. We hypothesized that other cancer types expressing LOXL2 and BRD4S could also benefit from the combinatorial treatment. Indeed, the CCLE drug sensitivity panel in Fig. 1A comprises cancer cell lines of different origins, not just TNBC, and corroborates that the relationship between LOXL2 expression levels and BRD4 sensitivity exist also beyond TNBC. Even though it is important to experimentally verify this hypothesis, we decided to pursue it in the future to broaden the applicability of the proposed strategy in preclinical settings.

-Authors in Figure 5H represent LOXL2 and BRD4s as integral chromatin looping factors together with MED1 at promoter and enhancer regions. However, this illustration is an overrepresentation of their finding because authors did not address the differential occupancy of BRD4S upon LOXL2 loss in DREAM-target-specific enhancer regions. If they wish to do so, they may use the RANK ORDERING OF SUPER-ENHANCERS (ROSE) package to call for super-enhancer regions in the proximity of DREAM-targets and confirm similar results as for their TSS-proximal sites.

We thank the reviewer for the useful suggestion. In the new version of the manuscript, we have simplified the representation, which now does not show super-enhancers. However, following the

reviewer's suggestion, we performed super enhancer analysis using ROSE. Results showed that BRD4S binds to super-enhancers more than BRD4L, including DREAM target gene super-enhancers. Additionally, while LOXL2 KD did not alter the binding of LOXL2 to DREAM target gene super-enhancers, it decreased the binding of BRD4S to them (Fig. EV7D, E). Overall, these data are in agreement with our hypothesis that BRD4S together with LOXL2 controls the expression of DREAM target genes.

-In the current manuscript, authors did not address the translational relevance of their proposed mechanism in the context of conventional therapies. Knowing that several BRD-specific compounds currently undergo clinical trials, authors should address if LOXL2 low (MDAMB468) and high (BT549) cells demonstrate a differential sensitivity to increasing doses of chemotherapy, in the presence or absence of BRD4. By doing that, LOXL2 apart from being a therapeutic target could be also used as a prognostic marker to stratify patients and achieve better response to standard therapies.

We really appreciate the reviewer's suggestion and we think this is a fundamental point. In the new version of the manuscript, we have performed further analysis using a greater panel of chemotherapeutic agents from the CCLE sensitivity database. We now show that LOXL2 low-expressing cells show significantly more sensitivity to BETi treatments, but not to conventional chemotherapeutic agents (e.g. doxorubicin, Olaparib, 5-fluorouracil, paclitaxel, etc.) (Fig. 1A and Fig. EV1), which set the rationale to further explore the functional relationship between BRD4 and LOXL2.

Minor points:

-In Figure 1D, the authors should convert the y-axis to a logarithmic scale to better represent the differences between JQ1, PXS, and combo. Also, One-way Anova should be performed between JQ1, PXS and combo.

We don't understand the reviewer's suggestion since Fig. 1D (Fig. 6B, right panel in the revised version) is a tumor picture for which the y-axis cannot be converted to a logarithmic scale.

-In Figure S6F, authors did not show the sensitivity of LOXL2 low and high cell lines for BRD4 KO. If LOXL2-proficient cells are less sensitive to JQ1, based on Figure 1B, authors should consider showing something similar from the gene essentiality database.

We agree with the reviewer and we apologize for this mistake. We have included the sensitivity of LOXL2 low and high cell lines for BRD4 KO and also for MYC KO (Fig. EV6G).

-Authors failed to discuss the work from Ozge Saatci et al (PMID: 32415208) regarding LOXL2 in TNBC and ECM reorganization as well as in other cancer entities (PMID: 35428659) in the context of ECM remodeling. Authors should realize that these published works and the current ones are not conflicting but complement each other.

We thank the reviewer for the suggestion. In the revised version of the manuscript, we discussed this work.

Reviewer #3 (Significance (Required)):

SIGNIFICANCE
The conception and findings are of enlightening significance for TNBC therapy, especially given the lack of targeted therapies in this particularly aggressive breast cancer subtype. Hence, I posit this work as highly relevant for the cancer epigenetics research community interested in characterizing unknown factors that facilitate the gene-activating function of epigenetic readers in health and disease.
My field of expertise is to uncover epigenetic vulnerabilities responsible for transcriptional plasticity driving drug tolerance in aggressive forms of breast cancer.

We would like to take the opportunity to thank the reviewer for the relevant suggestions. We strongly believe the revised version of the manuscript has been substantially improved by addressing the comments the reviewer made.

19th Sep 2023

Dear Dr. Sdelci,

Thank you for the submission of your revised manuscript to EMBO Molecular Medicine, and please accept my apologies for the delay in getting back to you in this busy time of the year. We have now received the reports from the referees who had originally reviewed your manuscript for Review Commons. As you will see below, they are supportive of publication pending minor revisions, and I am therefore pleased to inform you that we will be able to accept your manuscript once the following points will be addressed:

1/ Referees comments:
- Please address in writing the remaining concerns from referee #1 and #2.

2/ Manuscript text:
- Please provide a .docx formatted version of the manuscript text without figures (including legends for main figures, EV figures and tables). We will send you shortly queries from our data editors, please address them in the manuscript text in track changes mode. Make sure that new changes are highlighted to be clearly visible.
- Please provide up to 5 keywords.
- Material and methods:
o Cell culture: please add a statement about cell authentication.
o Animal studies: please include information on housing and husbandry conditions.
o Antibodies: please provide dilutions/concentrations.
o Statistics: please provide information on randomization, blinding, sample size and exclusion/inclusion criteria.
o The tables (plasmids and primers) should have a title (Table 1 and Table 2) and should be moved after the main figure legends. Both tables need to be referenced in the manuscript text.
o Ethics should be part of the Materials and Methods section.
- Thank you for providing a 'Data Availability' section, please merge 'Data and Code Availability' and provide a link to access the data.
- Author contributions: CRediT has replaced the traditional author contributions section because it offers a systematic machine-readable author contributions format that allows for more effective research assessment. Please remove the Authors Contributions from the manuscript and use the free text boxes beneath each contributing author's name in our system to add specific details on the author's contribution. More information is available in our guide to authors.
- Acknowledgements: the complete funding information should be provided both in the acknowledgements and in the submission system (currently, Juan de la Cierva-Formación fellowship (FJC2019-040598-I), AECC (INVES20036TIAN), Ramón y Cajal investigator contract (RYC2020-029098-I), la Caixa Foundation PhD fellowship (ID 100010434; fellowship code LCF/BQ/DI19/11730061) are missing in the submission system).
- Please move your COI after the Acknowledgements and rename "Disclosure statement and competing interests": We updated our journal's competing interests policy in January 2022 and request authors to consider both actual and perceived competing interests. Please review the policy https://www.embopress.org/competing-interests and update your competing interests if necessary.
- Please reformat the references in alphabetical order, and with 10 authors listed before et al.

3/ Figures:
- The figures should be removed from the manuscript text and uploaded as individual, high resolution figure files as .eps, .tif, .jpg (one file per figure). For guidance, download the 'Figure Guide PDF' (https://www.embopress.org/page/journal/17574684/authorguide#figureformat). Main figure legends should be after References, EV figure legends after Tables. We can accommodate up to 5 EV figures; the remaining EV figures should be compiled, with their legends, in a PDF and renamed Appendix Figure S1 etc. The appendix needs a ToC with page numbers.
- Kindly provide exact p values (including for ns, non significant) in the figures or their legends (including EV figures and Appendix).
- Movie file: the legend needs to be removed from the manuscript and zipped to the movie file.
- Tables EV1, EV2 and EV3 should be uploaded as three separate files, with their legends included.
- During a standard figure check, we noted similarities between different stainings in Figure EV6E, please check the composition of the figure, provide source data (see below point 4/) and clarify.

4/ At EMBO Press we ask authors to provide source data for the main figures. Our source data coordinator will contact you to discuss which figure panels we would need source data for and will also provide you with helpful tips on how to upload and organize the files.

5/ Checklist: Please correct to reflect the materials and methods.

6/ Please note that all corresponding authors are required to supply an ORCID ID for their name upon submission of a revised manuscript. An ORCID identifier is currently missing for S. Peiró.

7/ The paper explained: EMBO Molecular Medicine articles are accompanied by a summary of the articles to emphasize the major findings in the paper and their medical implications for the non-specialist reader. Please provide a draft summary of your article highlighting
- the medical issue you are addressing,
- the results obtained and
- their clinical impact.
This may be edited to ensure that readers understand the significance and context of the research. Please refer to any of our published articles for an example.

8/ Every published paper now includes a 'Synopsis' to further enhance discoverability. Synopses are displayed on the journal webpage and are freely accessible to all readers. They include a short stand first (maximum of 300 characters, including space) as well as 2-5 one-sentences bullet points that summarizes the paper. Please write the bullet points to summarize the key NEW findings. They should be designed to be complementary to the abstract - i.e. not repeat the same text. We encourage inclusion of key acronyms and quantitative information (maximum of 30 words / bullet point). Please use the passive voice. Please attach these in a separate file or send them by email, we will incorporate them accordingly.

Please also suggest a striking image or visual abstract to illustrate your article as a PNG file 550 px wide x 300-600 px high.

9/ As part of the EMBO Publications transparent editorial process initiative (see our Editorial at http://embomolmed.embopress.org/content/2/9/329), EMBO Molecular Medicine will publish online a Review Process File (RPF) to accompany accepted manuscripts.
This file will be published in conjunction with your paper and will include the anonymous referee reports, your point-by-point response and all pertinent correspondence relating to the manuscript. Let us know whether you agree with the publication of the RPF and as here, if you want to remove or not any figures from it prior to publication.
Please note that the Authors checklist will be published at the end of the RPF.

I look forward to receiving your revised manuscript.

Yours sincerely,

Lise Roth

Lise Roth, PhD
Senior Editor
EMBO Molecular Medicine

***** Reviewer's comments *****

Referee #1 (Remarks for Author):

My main issue with the initial submission was that mismatch between the data and the claims, rather than with the study itself. The review has addressed most of these concerns by toning down some of the exaggerated claims. This is a fairly massive mechanistic study that supports a reasonably solid claim on the LoxL2/BRD4-MED1 interaction, and its involvement on the regulation of multiple transcriptional programs, including G1/S entry. I think this finding as well as mechanistic sophistication of the elucidation of molecular interactions warrant a publication in EMBO Molecular Medcine journal. I think authors should have emphasize novelty of these mechanistic aspects rather than making questionable claims about the magnitude of the effect (arrest of proliferation) and therapeutic implications of their findings.

I do not think the term "arrest" in describing the observed cell cycle effect of the combination treatment is warranted; proliferation is inhibited, but not stopped, which is to be expected based on the aggregate of the current knowledge on the cell cycle

regulation. "Suppression" would be a more reasonable descriptor.

The claim of a therapeutic potential is fairly weak. The effect of dual inhibition of LoxL2 an BRD4 is limited to a subset of the models, and even with the selected models the combined therapy suppresses tumor growth without inducing remissions. Within a clinical trial, this would have been registered as either no response or, at best, disease stabilization. Therefore, I suggest deemphasizing the clinical utility claim.

The cell cycle analyses do not justify the conclusion that LoxL2 suppression impedes S/G2-M progression. G1/S arrest appears to be more likely from the data that is shown. S/G2 arrest would lead to an accumulation in the cells at late S phase, which would show up in the G2 peak in Figures 4A and 4C; this would lead to an expansion rather than a reduction of the G2 peak. I am also surprised that the 4C analyses indicate a compatible % of cells in the S phase of the cell cycle between DMSO control and PXS treated groups. Unless there is a S phase arrest (which is unlikely, but can be analyzed explicitly), the results do not quite make sense, as, given the relatively constant duration of S phase, the equal proportion of cell in S cycle would lead to the equal proportion of net proliferation (given the same death rates. I suspect a substantial proportion of the S phase cycle cells are actually G1/S arrested, as PI-only based analyses do not offer sufficient quantitative power. SLBP1 reporter assays do support the notion of cell cycle progression, but the SLBP-mTurquoise2 do not discriminate between G1/S and S/G2 arrest. A PI-BrdU combination, or the inclusion of a G1 specific cell cycle reporter would have allowed the discrimination. However, I do not think the discrimination between G1/S and S/G2 arrest is essential for the main conclusions of this work. Unless authors want to specifically claim the S phase or S/G2 arrest, the issue could be resolved by a slight modification of the verbiage, to make inferences consistent with the data.

Referee #2 (Remarks for Author):

The authors addressed my concerns through the revised version of their manuscript. However, a few critical points were not fully addressed and it is important that they are resolved before publication.

Major:

A critical misinterpretation remains with regard to the function of MuvB complexes. The authors correctly note that the repressive DREAM complex disassembles during G1 phase. However, they falsely claim that "Transcription of early cell cycle genes requires the association of the MuvB complex either with E2F transcription factors (G1-S) or the MYB Proto-Oncogene Like 2 (B-Myb) (S-G2)." And similarly in the discussion: "B-Myb, which controls the expression of early cell cycle genes". It is important to note that the MuvB complex does not associate with the activating E2F transcription factors and is not required for the transcription of early cell cycle genes that display expression maxima in S phase. While the MuvB complex associates with B-MYB during S/G2 and with FOXM1 during G2/M, it binds and regulates only late cell cycle genes that display expression maxima in G2/M. Importantly, the general absence of trans-activating MuvB complexes from early cell cycle genes stands in contrast to the authors' conclusion that their "results overall suggest that BRD4S, LOXL2, and MED1 interact with the MuvB complex and B-Myb to promote the transcription of early cell cycle genes". LOXL2 and BRD4S binding to LIN9 and B-MYB does not support a role of LOXL2/BRD4S in regulating the transcription of early cell cycle genes.
Unless the authors can show that BRD4S/LOXL2-bound cell cycle genes are affected differently than cell cycle genes that are not bound by those factors, their observations are more likely caused by indirect effects, i.e., reduced proliferation or partial cell cycle arrest which leads to the downregulation of all cell cycle genes and not the other way around.

Minor:

Full DESeq2 results table should be provided as Supplementary Table (LOXL2 knock-down).

BRD4 peaks should be provided through a Supplementary Table.

Some Figure referrals need to be corrected/updated, e.g., to Fig. 3D and E.

The headline of Figure 3 reads "The interaction between LOXL2 and BRD4S regulates DREAM target gene expression", but the functional relevance of the interaction for regulating DREAM targets actually has not been assessed.

Referee #3 (Remarks for Author):

The authors sufficiently answered all reviewer's comments and the revised work is significantly more solid and certainly novel.

***
Rev_Com_number: RC-2022-01515
New_manu_number: EMM-2023-18459
Corr_author: Sdelci
Title: Interactions between BRD4S, LOXL2, and MED1 drive cell cycle transcription in triple-negative breast cancer

Response to reviewers: EMM-2023-18459

Referee #1 (Remarks for Author):

My main issue with the initial submission was that mismatch between the data and the claims, rather than with the study itself. The review has addressed most of these concerns by toning down some of the exaggerated claims. This is a fairly massive mechanistic study that supports a reasonably solid claim on the LoxL2/BRD4-MED1 interaction, and its involvement on the regulation of multiple transcriptional programs, including G1/S entry. I think this finding as well as mechanistic sophistication of the elucidation of molecular interactions warrant a publication in EMBO Molecular Medcine journal. I think authors should have emphasize novelty of these mechanistic aspects rather than making questionable claims about the magnitude of the effect (arrest of proliferation) and therapeutic implications of their findings.

I do not think the term "arrest" in describing the observed cell cycle effect of the combination treatment is warranted; proliferation is inhibited, but not stopped, which is to be expected based on the aggregate of the current knowledge on the cell cycle regulation. "Suppression" would be a more reasonable descriptor.

The claim of a therapeutic potential is fairly weak. The effect of dual inhibition of LoxL2 an BRD4 is limited to a subset of the models, and even with the selected models the combined therapy suppresses tumor growth without inducing remissions. Within a clinical trial, this would have been registered as either no response or, at best, disease stabilization. Therefore, I suggest deemphasizing the clinical utility claim.

The cell cycle analyses do not justify the conclusion that LoxL2 suppression impedes S/G2-M progression. G1/S arrest appears to be more likely from the data that is shown. S/G2 arrest would lead to an accumulation in the cells at late S phase, which would show up in the G2 peak in Figures 4A and 4C; this would lead to an expansion rather than a reduction of the G2 peak. I am also surprised that the 4C analyses indicate a compatible % of cells in the S phase of the cell cycle between DMSO control and PXS treated groups. Unless there is a S phase arrest (which is unlikely, but can be analyzed explicitly), the results do not quite make sense, as, given the relatively constant duration of S phase, the equal proportion of cell in S cycle would lead to the equal proportion of net proliferation (given the same death rates. I suspect a substantial proportion of the S phase cycle cells are actually G1/S arrested, as PI-only based analyses do not offer sufficient quantitative power. SLBP1 reporter assays do support the notion of cell cycle progression, but the SLBP-mTurquoise2 do not discriminate between G1/S and S/G2

C/ Dr. Aiguader, 88
08003 Barcelona
T +34 316 01 00
www.crg.es

arrest. A PI-BrdU combination, or the inclusion of a G1 specific cell cycle reporter would have allowed the discrimination. However, I do not think the discrimination between G1/S and S/G2 arrest is essential for the main conclusions of this work. Unless authors want to specifically claim the S phase or S/G2 arrest, the issue could be resolved by a slight modification of the verbiage, to make inferences consistent with the data.

We thank the reviewer for their support and appreciation of our work. We have now further toned down some of our statements.

Regarding the cell cycle analysis, we say:

LOXL2 KD significantly reduced the number of cells in the G2-M phase of the cell cycle, indicating a cell cycle progression defect (Fig. 4A).

Cell cycle analysis confirmed that PXS treatment of MDA-MB-231 cells significantly decreased the G2-M population, thereby impairing cell cycle progression (Fig. 4C).

We do not actually refer to a particular effect of the KD or PXS treatment arresting cells in G2/M, but rather state that we observed a significant decrease of cells in G2/M, which is consistent with a cell cycle defect.

Regarding the experiment with SLBP-Torquoise2, we respectfully disagree with the reviewer that discrimination between G1/S and G2/M is not possible. The advantage of the time-lapse is that it allows identifying when a cell starts accumulating the SLBP-Torquoise2 fluorescence (G1), reaches the maximum level (G1/S), and starts losing it (G2/M). However, we agreed on a rewording given the fact that this is not central to the discovery. The text is not reading as follows:

As expected, DMSO-treated MDA-MB-231 cells progressed into the cell cycle, first acquiring and then progressively losing the accumulation of nuclear SLBP-mTurquoise2. In contrast, PXS-treated cells showed a clear retention of mTurquoise2 nuclear fluorescence (from SLBP), **confirming that the treatment impairs cell cycle progression**.

Referee #2 (Remarks for Author):

The authors addressed my concerns through the revised version of their manuscript. However, a few critical points were not fully addressed and it is important that they are resolved before publication.

Major:

A critical misinterpretation remains with regard to the function of MuvB complexes. The authors correctly note that the repressive DREAM complex disassembles during G1 phase. However, they falsely claim that "Transcription of early cell cycle genes requires the association of the MuvB complex either with E2F transcription factors (G1-S) or the MYB Proto-Oncogene Like 2 (B-Myb) (S-G2)." And similarly in the discussion: "B-Myb, which controls the expression of early cell cycle genes". It is important to note that the MuvB complex does not associate with the activating E2F transcription factors and is not required for the transcription of early cell cycle genes that display expression maxima in S phase. While the MuvB complex associates with B-MYB during S/G2 and with FOXM1 during G2/M, it binds and regulates only late cell cycle genes that display expression maxima in G2/M. Importantly, the general absence of trans-activating MuvB complexes from early cell cycle genes stands in contrast to the authors' conclusion that their "results overall suggest that BRD4S, LOXL2, and MED1 interact with the MuvB complex and B-Myb to promote the transcription of early cell cycle genes". LOXL2 and BRD4S binding to LIN9 and B-MYB does not support a role of LOXL2/BRD4S in regulating the transcription of early cell cycle genes.
Unless the authors can show that BRD4S/LOXL2-bound cell cycle genes are affected differently than cell cycle genes that are not bound by those factors, their observations are more likely caused by indirect effects, i.e., reduced proliferation or partial cell cycle arrest which leads to the downregulation of all cell cycle genes and not the other way around.

Minor:

Full DESeq2 results table should be provided as Supplementary Table (LOXL2 knock-down).

BRD4 peaks should be provided through a Supplementary Table.

Some Figure referrals need to be corrected/updated, e.g., to Fig. 3D and E.

The headline of Figure 3 reads "The interaction between LOXL2 and BRD4S regulates DREAM target gene expression", but the functional relevance of the interaction for regulating DREAM targets actually has not been assessed.

We thank the reviewer for their positive evaluation of our work. We are also very grateful to have them as reviewers because they clarified for us the dynamics of the DREAM complex. We now removed from the text the word **early** when referring to the cell cycle effect observed when perturbing BRD4S and LOXL2. The description of the MuvB function has also changed as follows:

C/ Dr. Aiguader, 88
08003 Barcelona
T +34 316 01 00
www.crg.es

Cell cycle progression relies on the finely tuned transcriptional control of DREAM target genes. The DREAM complex comprises multiple subunits, including the MuvB complex (LIN9, LIN37, LIN52, LIN54, and RBBP), the Rb-like proteins p130 and E2F4, and DP1, and acts as a transcriptional repressor of cell cycle genes. When cells start cycling the DREAM complex disassembles, leaving only the MuvB complex bound to the promoters of cell cycle genes. **MuvB coordinates gene expression in the G2/M phases via interacting with the Proto-Oncogene Like 2 (B-Myb) and the Forkhead Box M1 (FOXM1) transcription factors**.

Additionally, we have addressed all the minor points, including tables with Full DESeq2 (Dataset EV1) and BRD4 peaks (Dataset EV2), corrected mislabeled figures, and changed the title of Figure 3, which now reads as follows:

LOXL2 and BRD4S regulate DREAM target gene expression

C/ Dr. Aiguader, 88
08003 Barcelona
T +34 316 01 00
www.crg.es

13th Oct 2023

Dear Dr. Sdelci,

Thank you for submitting your revised files. A few minor points still need to be addressed before I can proceed with acceptance:

1/ Please address the following remaining query from the data editors:
"Please note that a separate 'Data Information' section is required in the legend of Figure EV2."

2/ Checklist:
- Please correct the checklist to reflect the information provided in the manuscript (i.e. housing and husbandry conditions).
- Please make sure you need to fill the section "microbes", as I don't think it applies to your study.

3/ An ORCID identifier is missing for S. Peiró. Unfortunately, we cannot do it on Dr. Peiró's behalf.

4/ The paper explained: I introduced minor modifications in your text, please let me know if you agree or amend as you see fit:
Problem
Among the different subtypes of breast cancer, triple-negative breast cancer (TNBC) accounts for 15% of all diagnosed cases. Due to the lack of effective treatment options and to the highly heterogeneous nature of this cancer subtype, patients with TNBC have a poor prognosis and are at a higher risk of developing drug resistance. Therefore, there is an urgent need to develop new therapeutic approaches to address this unmet medical need.
Results
We found that LOXL2 expression can predict the response to BET inhibitors (BETi) in cancer cells. In TNBC, LOXL2 interacts with the short isoform of BRD4 (BRD4S), the functional partner MED1, and the transcriptional regulator B-Myb in the nucleus. These interactions play a crucial role in the formation of BRD4-MED1 transcriptional foci, which regulate the transcription of cell cycle genes, and ultimately, TNBC proliferation. When LOXL2 and BRD4 are inhibited simultaneously, LOXL2-BRD4S-MED1 interactions are dismantled, leading to the suppression of TNBC progression both in vitro and in vivo through synergistic cooperation.
Impact
Inhibiting both BRD4 and LOXL2 shows promise as a novel therapeutic approach for the treatment of TNBC.

5/ Synopsis:
I slightly modified your text to accommodate our style and format, please let me know if you agree with the following or amend as you see fit:

Investigation of the molecular mechanisms underlying triple-negative breast cancer (TNBC) proliferation led to the identification of new therapeutic vulnerabilities.

• LOXL2 expression predicted cancer cells response to BET inhibitor (BETi).
• LOXL2 interacted with the short isoform of BRD4 (BRD4S) and MED1 in the nucleus of TNBC cells.
• LOXL2-BRD4S-MED1 interactions were responsible for regulating cell cycle genes expression.
• Combined inhibition of LOXL2 and BRD4 disrupted LOXL2-BRD4S-MED1 interaction, MED1-BRD4 colocalization, and the formation of BRD4 transcriptional foci.
• Combined inhibition of LOXL2 and BRD4 suppressed TNBC proliferation in vitro and in vivo.

Please accept all previous changes and only keep in track changes mode any new modification.

I look forward to receiving a new revised version of your manuscript as soon as possible.

With kind regards,

Lise Roth

Lise Roth, PhD
Senior Editor
EMBO Molecular Medicine

All editorial and formatting issues were resolved by the authors.

17th Oct 2023

Dear Dr. Sdelci,

Thank you for bearing with the last editorial revisions. I am pleased to inform you that your manuscript is now accepted for publication and will be sent to our publisher to be included in the next available issue of EMBO Molecular Medicine!

Please note that in the checklist, I have removed the text in the right column - Microbes. Let us know immediately if you do not agree with this modification.

Please read below for additional IMPORTANT information regarding your article, its publication and the production process.

Congratulations on your interesting work!

With kind regards,

Lise Roth

Lise Roth, Ph.D
Senior Editor
EMBO Molecular Medicine

Follow us on Twitter @EmboMolMed
Sign up for eTOCs at embopress.org/alertsfeeds

*** *** *** IMPORTANT INFORMATION *** *** ***

SPEED OF PUBLICATION
The journal aims for rapid publication of papers, using using the advance online publication "Early View" to expedite the process: A properly copy-edited and formatted version will be published as "Early View" after the proofs have been corrected. Please help the Editors and publisher avoid delays by providing e-mail address(es), telephone and fax numbers at which author(s) can be contacted.

Should you be planning a Press Release on your article, please get in contact with embomolmed@wiley.com as early as possible, in order to coordinate publication and release dates.

LICENSE AND PAYMENT:

All articles published in EMBO Molecular Medicine are fully open access: immediately and freely available to read, download and share.

EMBO Molecular Medicine charges an article processing charge (APC) to cover the publication costs. You, as the corresponding author for this manuscript, should have already received a quote with the article processing fee separately. Please let us know in case this quote has not been received.

Once your article is at Wiley for editorial production you will receive an email from Wiley's Author Services system, which will ask you to log in and will present you with the publication license form for completion. Within the same system the publication fee can be paid by credit card, an invoice, pro forma invoice or purchase order can be requested.

Payment of the publication charge and the signed Open Access Agreement form must be received before the article can be published online.

PROOFS

You will receive the proofs by e-mail approximately 2 weeks after all relevant files have been sent o our Production Office. Please return them within 48 hours and if there should be any problems, please contact the production office at embopressproduction@wiley.com.

Please inform us if there is likely to be any difficulty in reaching you at the above address at that time. Failure to meet our deadlines may result in a delay of publication.

All further communications concerning your paper proofs should quote reference number EMM-2023-18459-V3 and be directed to the production office at embopressproduction@wiley.com.

***

Rev_Com_number: RC-2022-01515
New_manu_number: EMM-2023-18459-V3
Corr_author: Sdelci
Title: Interactions between BRD4S, LOXL2, and MED1 drive cell cycle transcription in triple-negative breast cancer

EMBO Press Author Checklist

Corresponding Author Name: Sara Sdelci	
Journal Submitted to: EMBO Molecular Medicine	
Manuscript Number: EMM-2023-18459	

USEFUL LINKS FOR COMPLETING THIS FORM
The EMBO Journal - Author Guidelines
EMBO Reports - Author Guidelines
Molecular Systems Biology - Author Guidelines
EMBO Molecular Medicine - Author Guidelines

Reporting Checklist for Life Science Articles (updated January
This checklist is adapted from Materials Design Analysis Reporting (MDAR) Checklist for Authors. MDAR establishes a minimum set of requirements in transparent reporting in the life sciences (see Statement of Task: 10.31222/osf.io/9sm4x). Please follow the journal's guidelines in preparing your
Please note that a copy of this checklist will be published alongside your article.

Abridged guidelines for figures
1. Data
The data shown in figures should satisfy the following conditions:
- the data were obtained and processed according to the field's best practice and are presented to reflect the results of the experiments in an accurate and unbiased manner.
- ideally, figure panels should include only measurements that are directly comparable to each other and obtained with the same assay.
- plots include clearly labeled error bars for independent experiments and sample sizes. Unless justified, error bars should not be shown for technical
- if n<5, the individual data points from each experiment should be plotted. Any statistical test employed should be justified.
- Source Data should be included to report the data underlying figures according to the guidelines set out in the authorship guidelines on Data

2. Captions
Each figure caption should contain the following information, for each panel where they are relevant:
- a specification of the experimental system investigated (eg cell line, species name).
- the assay(s) and method(s) used to carry out the reported observations and measurements.
- an explicit mention of the biological and chemical entity(ies) that are being measured.
- an explicit mention of the biological and chemical entity(ies) that are altered/varied/perturbed in a controlled manner.
- the exact sample size (n) for each experimental group/condition, given as a number, not a range;
- a description of the sample collection allowing the reader to understand whether the samples represent technical or biological replicates (including how many animals, litters, cultures, etc.).
- a statement of how many times the experiment shown was independently replicated in the laboratory.
- definitions of statistical methods and measures:
  - common tests, such as t-test (please specify whether paired vs. unpaired), simple χ2 tests, Wilcoxon and Mann-Whitney tests, can be unambiguously identified by name only, but more complex techniques should be described in the methods section;
  - are tests one-sided or two-sided?
  - are there adjustments for multiple comparisons?
  - exact statistical test results, e.g., P values = x but not P values < x;
  - definition of 'center values' as median or average;
  - definition of error bars as s.d. or s.e.m.

Please complete ALL of the questions below.
Select "Not Applicable" only when the requested information is not relevant for your study.

Materials

Newly Created Materials	Information included in the manuscript?	In which section is the information available? (Reagents and Tools Table, Materials and Methods, Figures, Data Availability Section)
New materials and reagents need to be available; do any restrictions apply?	Yes	Materials and Methods (new materials, such as plasmids, are available upon request by contacting the corresponding author)

Antibodies	Information included in the manuscript?	In which section is the information available? (Reagents and Tools Table, Materials and Methods, Figures, Data Availability Section)
For **antibodies** provide the following information:<br>- Commercial antibodies: RRID (if possible) or supplier name, catalogue number and or/clone number<br>- Non-commercial: RRID or citation	Yes	Materials and Methods

DNA and RNA sequences	Information included in the manuscript?	In which section is the information available? (Reagents and Tools Table, Materials and Methods, Figures, Data Availability Section)
**Short novel DNA or RNA including primers, probes**: provide the sequences.	Yes	Table 2

Cell materials	Information included in the manuscript?	In which section is the information available? (Reagents and Tools Table, Materials and Methods, Figures, Data Availability Section)
**Cell lines:** Provide species information, strain. Provide accession number in repository **OR** supplier name, catalog number, clone number, and/**OR** RRID.	Yes	Materials and Methods
**Primary cultures:** Provide species, strain, sex of origin, genetic modification status.	Not Applicable	
Report if the cell lines were recently **authenticated** (e.g., by STR profiling) and tested for mycoplasma contamination.	Yes	Materials and Methods

Experimental animals	Information included in the manuscript?	In which section is the information available? (Reagents and Tools Table, Materials and Methods, Figures, Data Availability Section)
**Laboratory animals or Model organisms:** Provide species, strain, sex, age, genetic modification status. Provide accession number in repository **OR** supplier name, catalog number, clone number, **OR** RRID.	Yes	Materials and Methods
**Animal observed in or captured from the field:** Provide species, sex, and age where possible.	Not Applicable	
Please detail **housing and husbandry conditions**.	Yes	Materials and Methods

Plants and microbes	Information included in the manuscript?	In which section is the information available? (Reagents and Tools Table, Materials and Methods, Figures, Data Availability Section)
**Plants:** provide species and strain, ecotype and cultivar where relevant, unique accession number if available, and source (including location for collected wild specimens).	Not Applicable	
**Microbes:** provide species and strain, unique accession number if available, and source.	Not Applicable	

Human research participants	Information included in the manuscript?	In which section is the information available? (Reagents and Tools Table, Materials and Methods, Figures, Data Availability Section)
If collected and within the bounds of privacy constraints report on age, sex and gender or ethnicity for all study participants.	Not Applicable	

Core facilities	Information included in the manuscript?	In which section is the information available? (Reagents and Tools Table, Materials and Methods, Figures, Data Availability Section)
If your work benefited from core facilities, was their service mentioned in the acknowledgments section?	Yes	Acknowledgment

Design

Study protocol	Information included in the manuscript?	In which section is the information available? (Reagents and Tools Table, Materials and Methods, Figures, Data Availability Section)

If study protocol has been **pre-registered, provide DOI in the manuscript**. For clinical trials, provide the trial registration number **OR** cite DOI.	Not Applicable	
Report the **clinical trial registration number** (at ClinicalTrials.gov or equivalent), where applicable.	Not Applicable	

Laboratory protocol	Information included in the manuscript?	In which section is the information available? (Reagents and Tools Table, Materials and Methods, Figures, Data Availability Section)
Provide DOI OR other citation details if **external detailed step-by-step protocols** are available.	Not Applicable	

Experimental study design and statistics	Information included in the manuscript?	In which section is the information available? (Reagents and Tools Table, Materials and Methods, Figures, Data Availability Section)
Include a statement about **sample size** estimate even if no statistical methods were used.	Yes	Figure captions
Were any steps taken to minimize the effects of subjective bias when allocating animals/samples to treatment (e.g. **randomization procedure**)? If yes, have they been described?	Yes	Statistics section
Include a statement about **blinding** even if no blinding was done.	Yes	Statistics section
Describe **inclusion/exclusion criteria** if samples or animals were excluded from the analysis. Were the criteria pre-established?<br><br>If sample or data points were omitted from analysis, report if this was due to attrition or intentional exclusion and provide justification.	Yes	statistics section
For every figure, are **statistical tests** justified as appropriate? Do the data meet the assumptions of the tests (e.g., normal distribution)? Describe any methods used to assess it. Is there an estimate of variation within each group of data? Is the variance similar between the groups that are being statistically compared?	Yes	Figure captions and Materials and Methods

Sample definition and in-laboratory replication	Information included in the manuscript?	In which section is the information available? (Reagents and Tools Table, Materials and Methods, Figures, Data Availability Section)
In the figure legends: state number of times the experiment was **replicated** in laboratory.	Yes	Figure captions
In the figure legends: define whether data describe **technical or biological replicates**.	Yes	Figure captions

Ethics

Ethics	Information included in the manuscript?	In which section is the information available? (Reagents and Tools Table, Materials and Methods, Figures, Data Availability Section)
Studies involving **human participants**: State details of **authority granting ethics approval** (IRB or equivalent committee(s), provide reference number for approval.	Not Applicable	
Studies involving **human participants**: Include a statement confirming that **informed consent** was obtained from all subjects and that the experiments conformed to the principles set out in the WMA Declaration of Helsinki and the Department of Health and Human Services Belmont Report.	Not Applicable	
Studies involving **human participants:** For publication of **patient photos**, include a statement confirming that consent to publish was obtained.	Not Applicable	
Studies involving experimental **animals**: State details of **authority granting ethics approval** (IRB or equivalent committee(s), provide reference number for approval. Include a statement of compliance with ethical regulations.	Yes	Ethics
Studies involving **specimen and field samples:** State if relevant **permits** obtained, provide details of authority approving study; if none were required, explain why.	Not Applicable	

Dual Use Research of Concern (DURC)	Information included in the manuscript?	In which section is the information available? (Reagents and Tools Table, Materials and Methods, Figures, Data Availability Section)
Could your study fall under dual use research restrictions? Please check biosecurity documents and list of **select agents and toxins** (CDC): https://www.selectagents.gov/sat/list.htm	Not Applicable	
If you used a select agent, is the security level of the lab appropriate and reported in the manuscript?	Not Applicable	
If a study is subject to dual use research of concern regulations, is the name of the **authority granting approval and reference number** for the regulatory approval provided in the manuscript?	Not Applicable	

Reporting

The MDAR framework recommends adoption of discipline-specific guidelines, established and endorsed through community initiatives. Journals have their own policy about requiring specific guidelines and recommendations to complement MDAR.

Adherence to community standards	Information included in the manuscript?	In which section is the information available? (Reagents and Tools Table, Materials and Methods, Figures, Data Availability Section)
State if relevant guidelines or checklists (e.g., **ICMJE, MIBBI, ARRIVE, PRISMA**) have been followed or provided.	Not Applicable	
For **tumor marker prognostic studies,** we recommend that you follow the **REMARK** reporting guidelines (see link list at top right). See author guidelines, under 'Reporting Guidelines'. Please confirm you have followed these guidelines.	Not Applicable	
For **phase II and III randomized controlled trials**, please refer to the **CONSORT** flow diagram (see link list at top right) and submit the CONSORT checklist (see link list at top right) with your submission. See author guidelines, under 'Reporting Guidelines'. Please confirm you have submitted this list.	Not Applicable	

Data Availability

Data availability	Information included in the manuscript?	In which section is the information available? (Reagents and Tools Table, Materials and Methods, Figures, Data Availability Section)
Have **primary datasets** been deposited according to the journal's guidelines (see 'Data Deposition' section) and the respective accession numbers provided in the Data Availability Section?	Yes	Data and code availability
Were **human clinical and genomic datasets** deposited in a public access-controlled repository in accordance to ethical obligations to the patients and to the applicable consent agreement?	Not Applicable	
Are **computational models** that are central and integral to a study available without restrictions in a machine-readable form? Were the relevant accession numbers or links provided?	Yes	Data and code availability
If publicly available data were reused, provide the respective **data citations in the reference list**.	Yes	Reference
